# Supplementary material for: Afterglow ice formed by phosphorescent luminophore-protein conjugates and complexes in aqueous solution at freezing temperature
Source: Nat Commun. 2025 Dec 22;17:226. doi: 10.1038/s41467-025-67670-z (PMC12779962; doi:10.1038/s41467-025-67670-z)
Supplement: Supplementary file 1 — Supplementary Information [file 41467_2025_67670_MOESM1_ESM.pdf]

Supplementary Information for

Afterglow ice formed by phosphorescent luminophore-protein conjugates and complexes in aqueous solution at freezing temperature

Xun Li *et al.*

*Corresponding author: Kaka Zhang, Email: zhangkaka@sioc.ac.cn

This PDF file includes:

Photophysical measurements, photographs of afterglow samples and others (Supplementary Figs. S1 to S60)
Structural characterization results and sample purity information (Supplementary Figs. S61 to S165)
Materials
Molecular synthesis
Physical measurements and instrumentation
TD-DFT calculations
Supporting Text

Photophysical measurements, photographs of afterglow samples and others

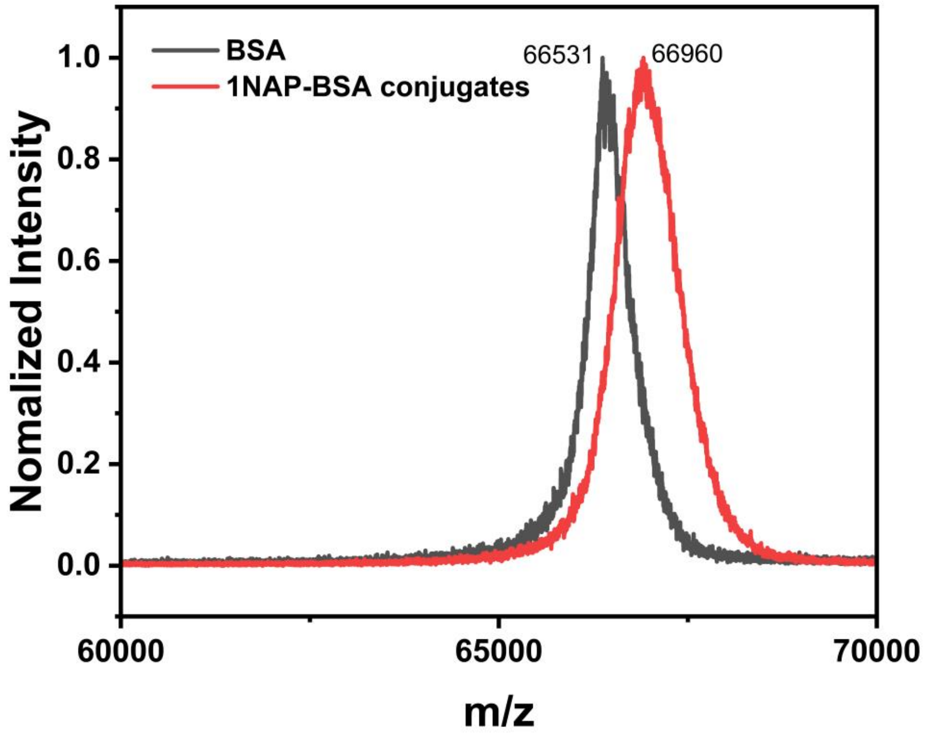

Supplementary Fig. 1. MALDI-TOF mass spectra of pristine **BSA** and **1NAP-BSA** conjugates.

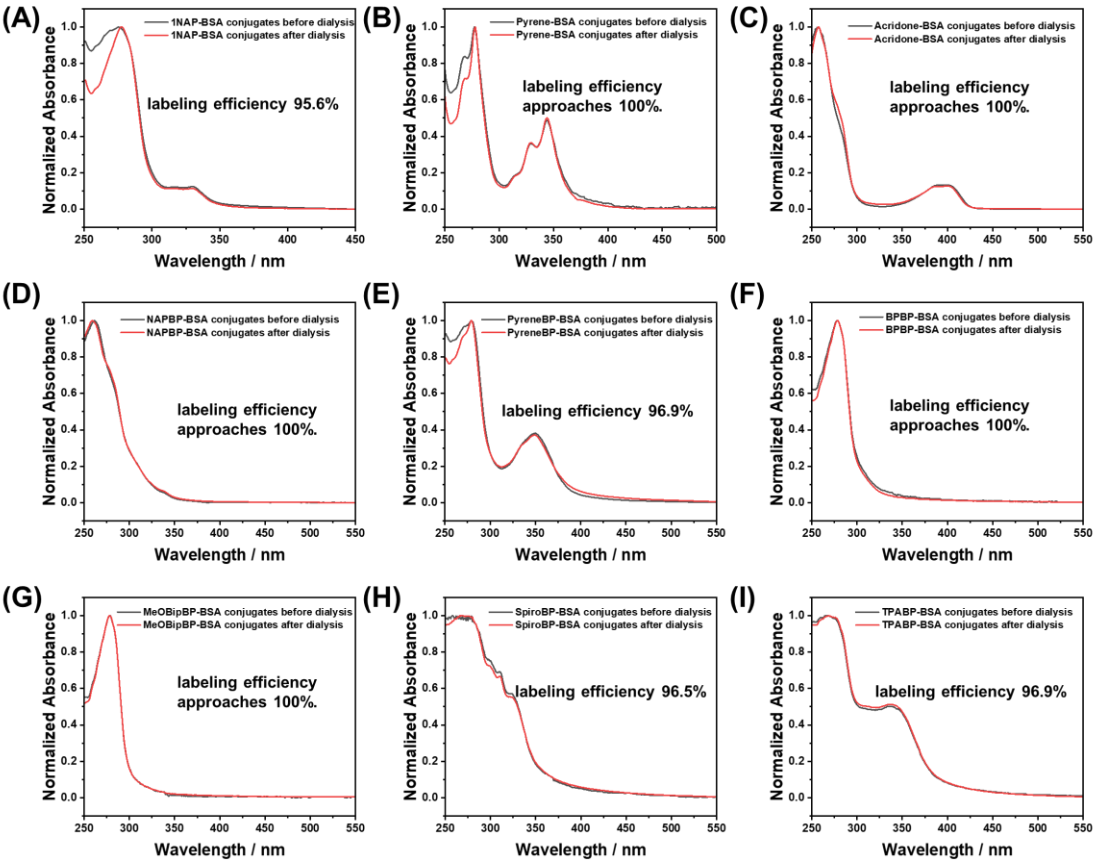

Supplementary Fig. 2. UV-vis absorption spectra of luminophore-**BSA** conjugates. UV-vis absorption spectra of the (A) **1NAP-BSA** conjugates, (B) **Pyrene-BSA** conjugates, (C) **Acridone-BSA** conjugates, (D) **NAPBP-BSA** conjugates, (E) **PyreneBP-BSA** conjugates, (F) **BPBP-BSA** conjugates, (G) **MeOBipBP-BSA** conjugates, (H) **SpiroBP-BSA** conjugates and (I) **TPABP-BSA** conjugates before and after dialysis. Before dialysis, the UV-vis absorption band in the lower-energy region corresponds to the combined signals of free luminophore and conjugated luminophore, while after dialysis, the UV-vis absorption band corresponds to the conjugated luminophore. Here we estimate the labeling efficiency from the ratio of absorbance (after dialysis)/absorbance (before dialysis) in the lower-energy region.

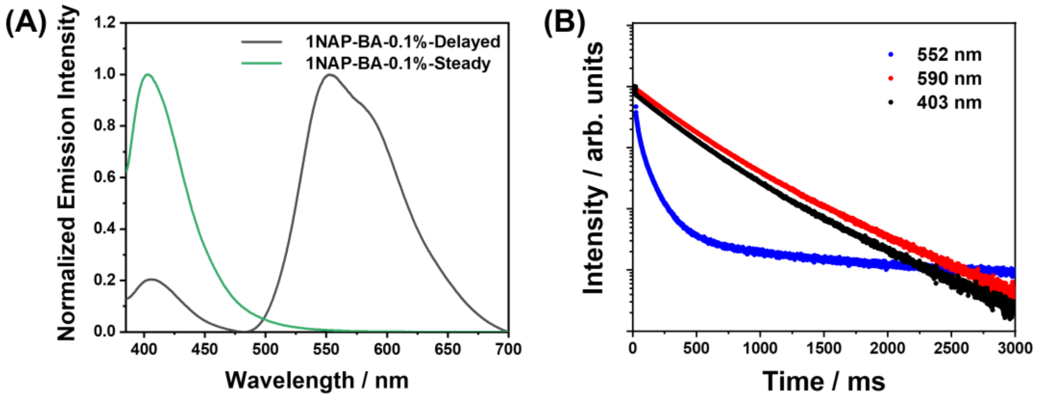

Supplementary Fig. 3. Photophysical property of **1NAP**-benzoic acid (BA)-0.1% sample. (A) Room-temperature steady-state and delayed emission spectra of the **1NAP**-benzoic acid (BA)-0.1% and (B) the corresponding emission decay curve under 365 nm excitation.

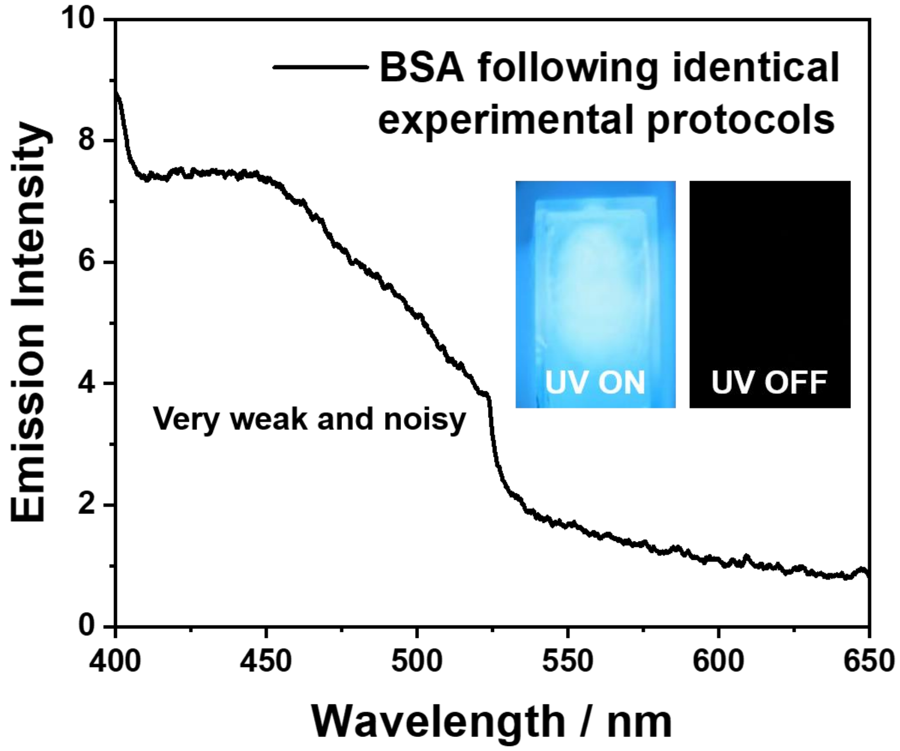

Supplementary Fig. 4. Delayed emission spectra of the frozen BSA solution (about 10 mg/mL) prepared following identical experimental protocols under 365 nm excitation.

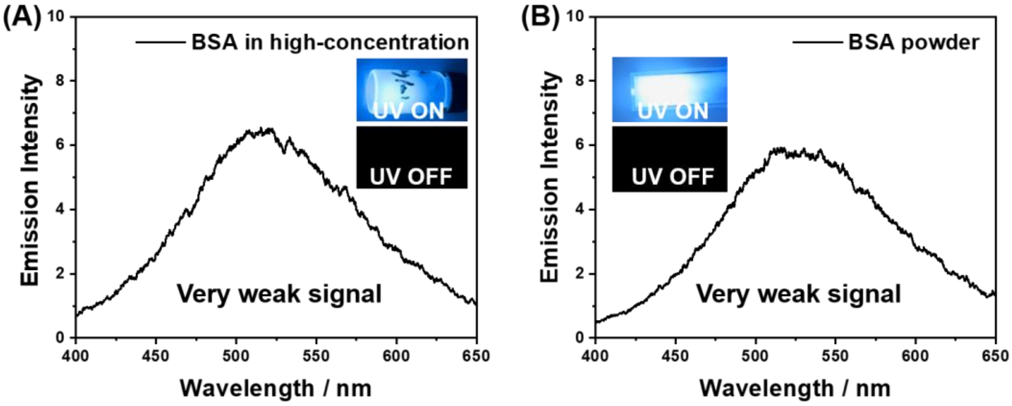

Supplementary Fig. 5. Emission spectra of BSA solution and powder. Delayed emission spectra of the high-concentration aqueous BSA solution (15 mg/mL) at -20 °C (A) and BSA powder at -20 °C (B) under 365 nm excitation.

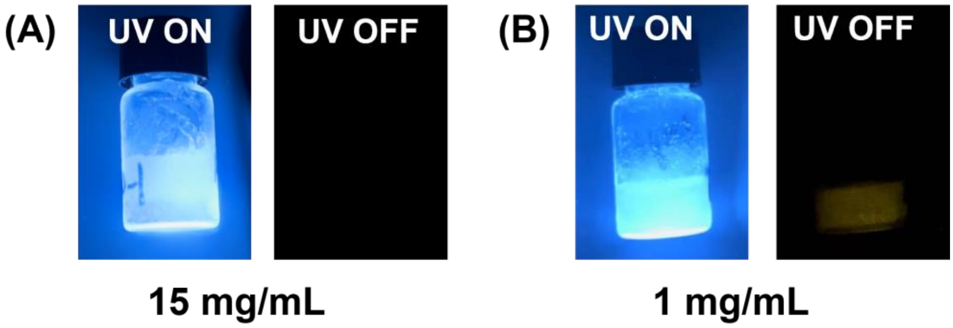

Supplementary Fig. 6. Photographs of **1NAP-PEG** aqueous solution and **1NAP-BSA** conjugates. Photograph of the frozen 15 mg/mL **1NAP-PEG** aqueous solution (A) and frozen 1 mg/mL **1NAP-BSA** conjugates (B) under UV and after switching off UV.

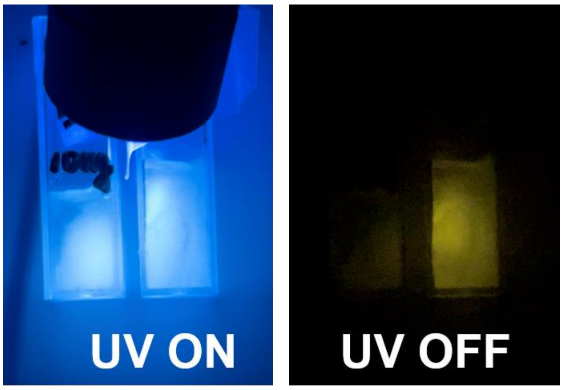

Supplementary Fig. 7. Photographs of **1NAP** and BSA (without covalent linkage) and **1NAP-BSA** conjugates. (left cell) Photographs of the frozen solution of **1NAP** and BSA (without covalent linkage) under UV and after switching off UV excitation; (right cell) photographs of the afterglow ice of **1NAP-BSA** conjugates for comparison.

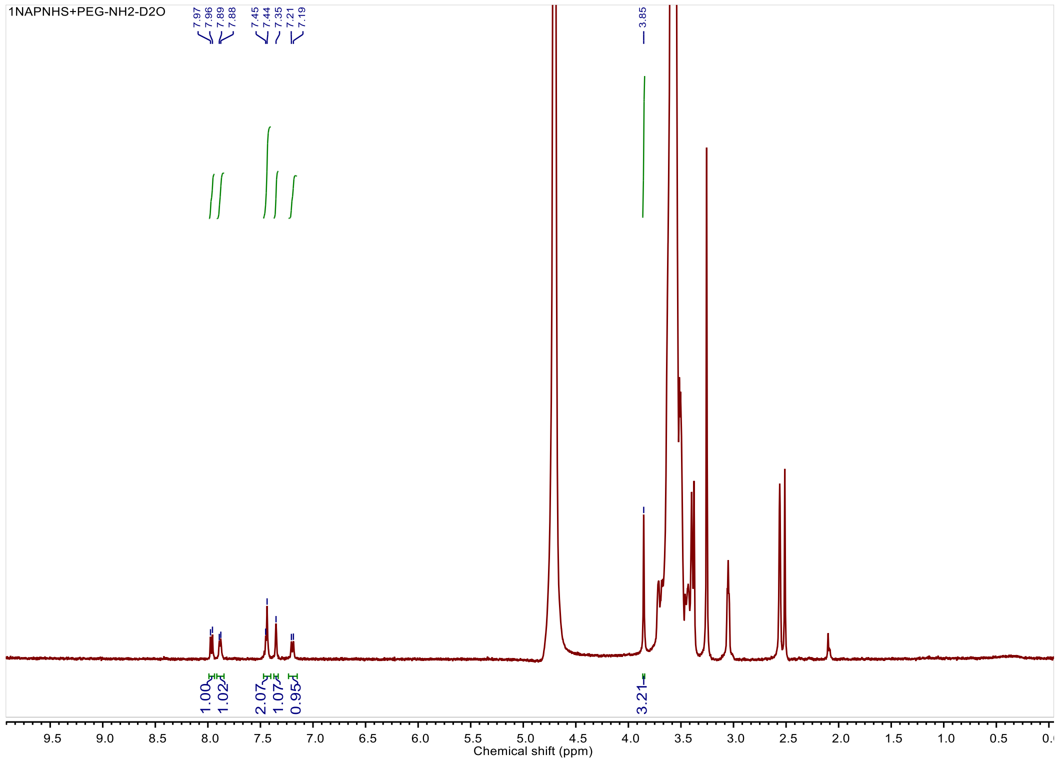

Supplementary Fig. 8. ^{1}H NMR (500 MHz, D_2O) result of **1NAP-PEG** sample in D_2O at room temperature.

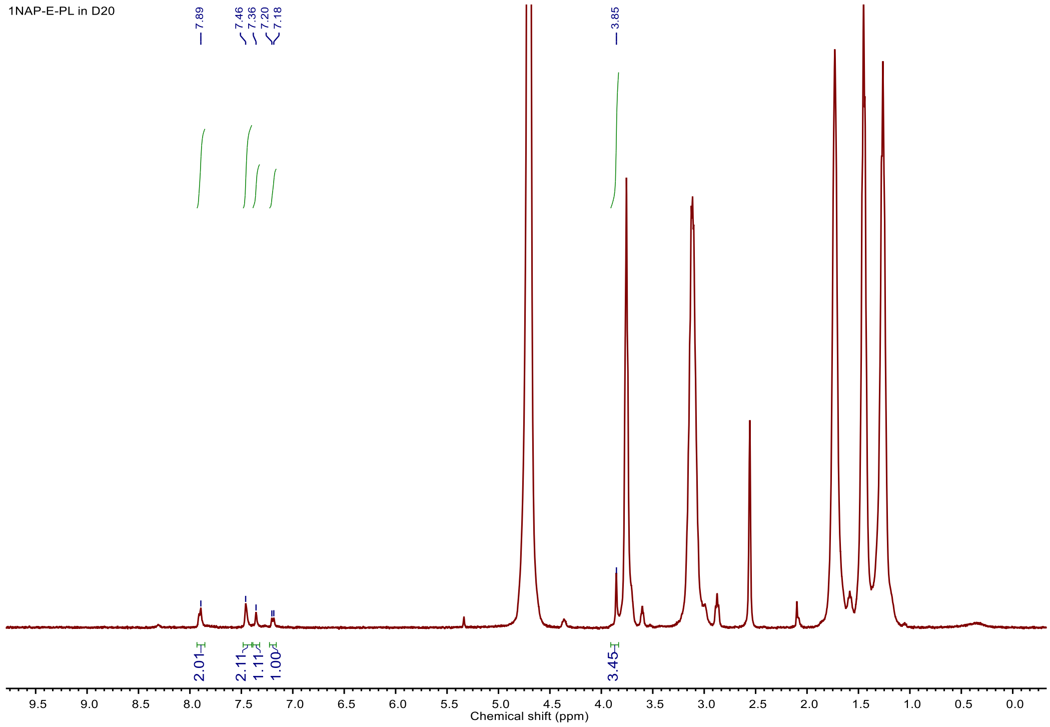

Supplementary Fig. 9. ^{1}H NMR (500 MHz, D_2O) result of **1NAP-EPL** sample in D_2O at room temperature.

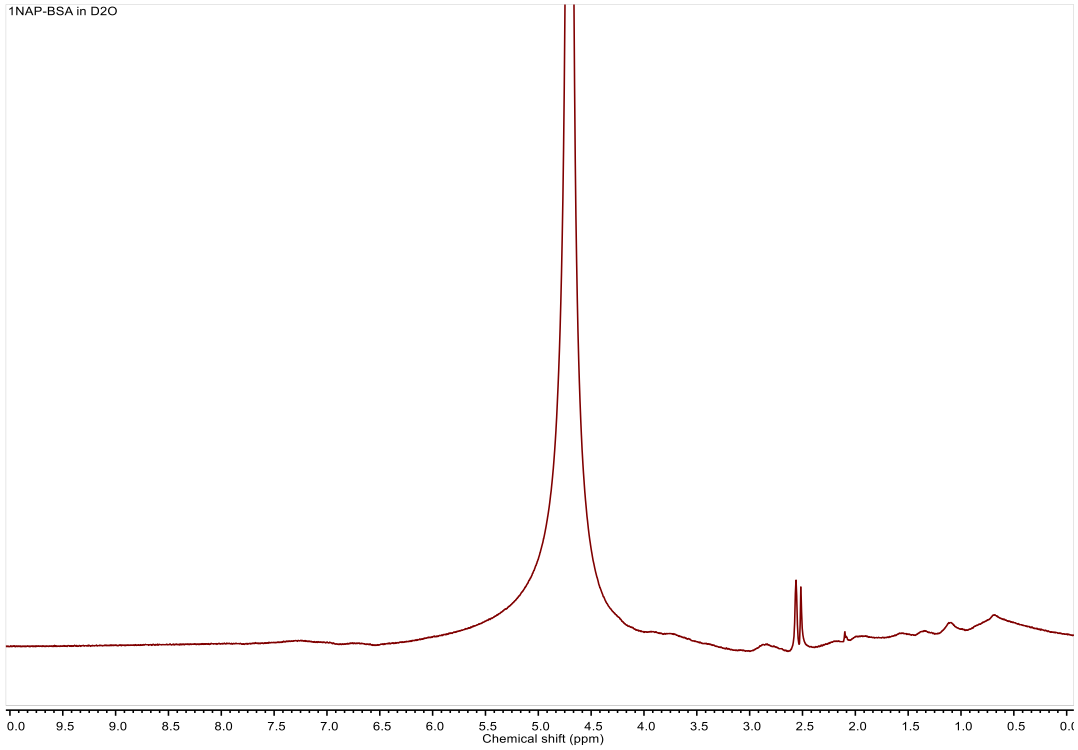

Supplementary Fig. 10. ^{1}H NMR (500 MHz, D_2O) result of **1NAP-BSA conjugates** in D_2O at room temperature.

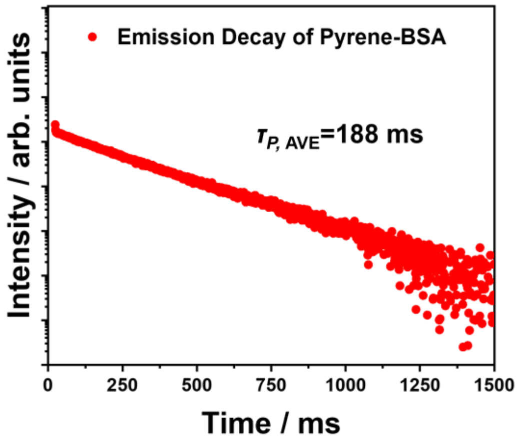

Supplementary Fig. 11. Emission decay curve of the frozen **Pyrene-BSA** conjugates under 365 nm excitation.

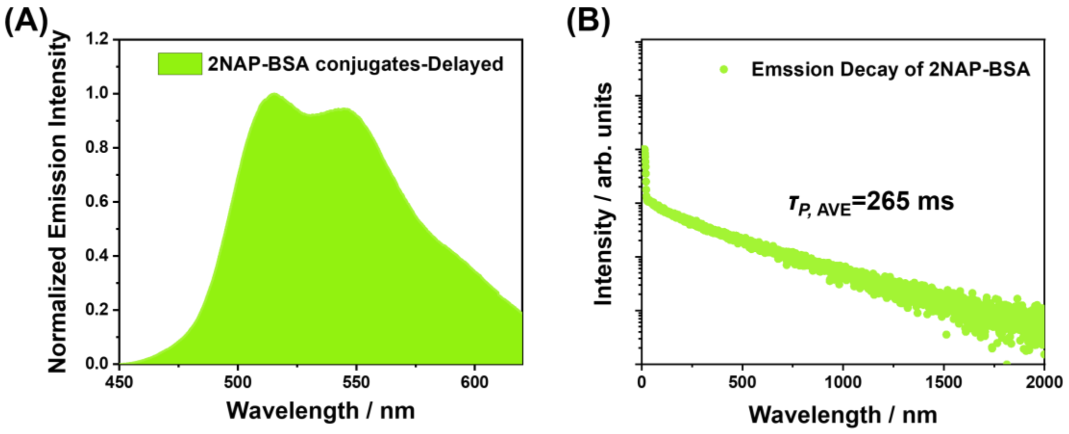

Supplementary Fig. 12. Photophysical property of **2NAP-BSA** conjugates. (A) Delayed emission spectra of the frozen **2NAP-BSA** conjugates and (B) the corresponding emission decay curve under 320 nm excitation.

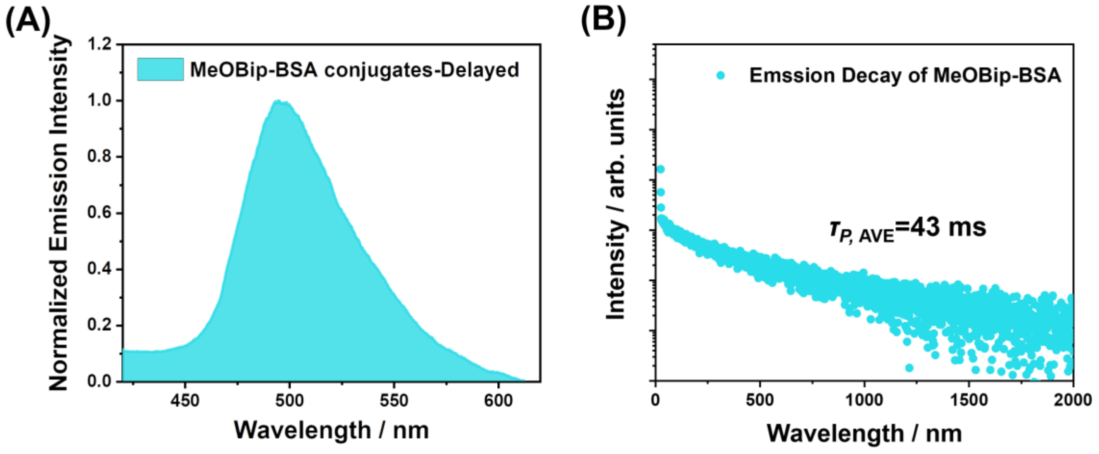

Supplementary Fig. 13. Photophysical property of **MeOBip-BSA** conjugates. (A) Delayed emission spectra of the frozen **MeOBip-BSA** conjugates and (B) the corresponding emission decay curve under 365 nm excitation.

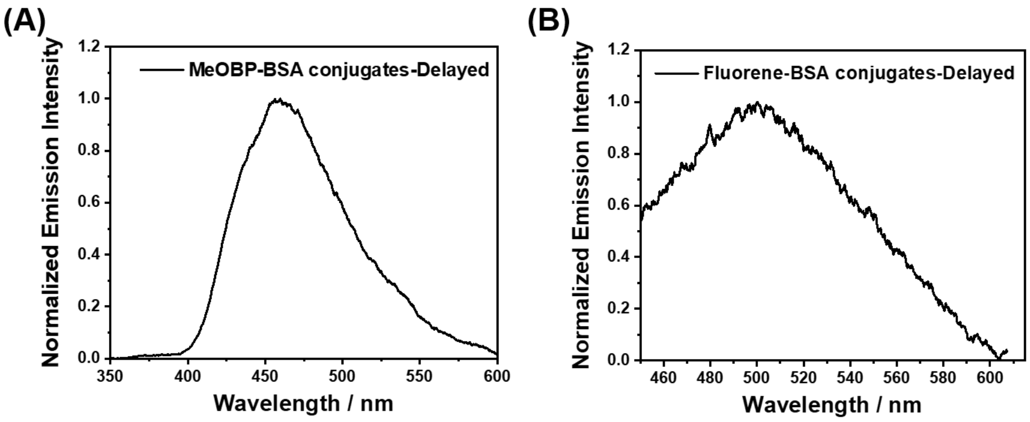

Supplementary Fig. 14. Emission spectra of **MeOBP-BSA** and **Fluorene-BSA** conjugates. Delayed emission spectra of the frozen **MeOBP-BSA** conjugates (A) and the frozen **Fluorene-BSA** conjugates (B) under 320 nm excitation.

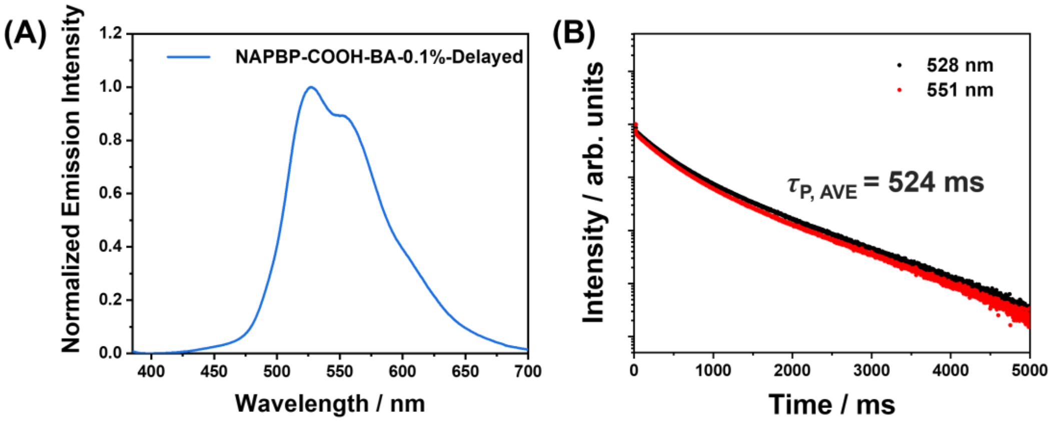

Supplementary Fig. 15. Photophysical property of **NAPBP-COOH**-BA-0.1% sample. (A) Room-temperature delayed emission spectra of the **NAPBP-COOH**-BA-0.1% powder and (B) the corresponding emission decay curve under 365 nm excitation.

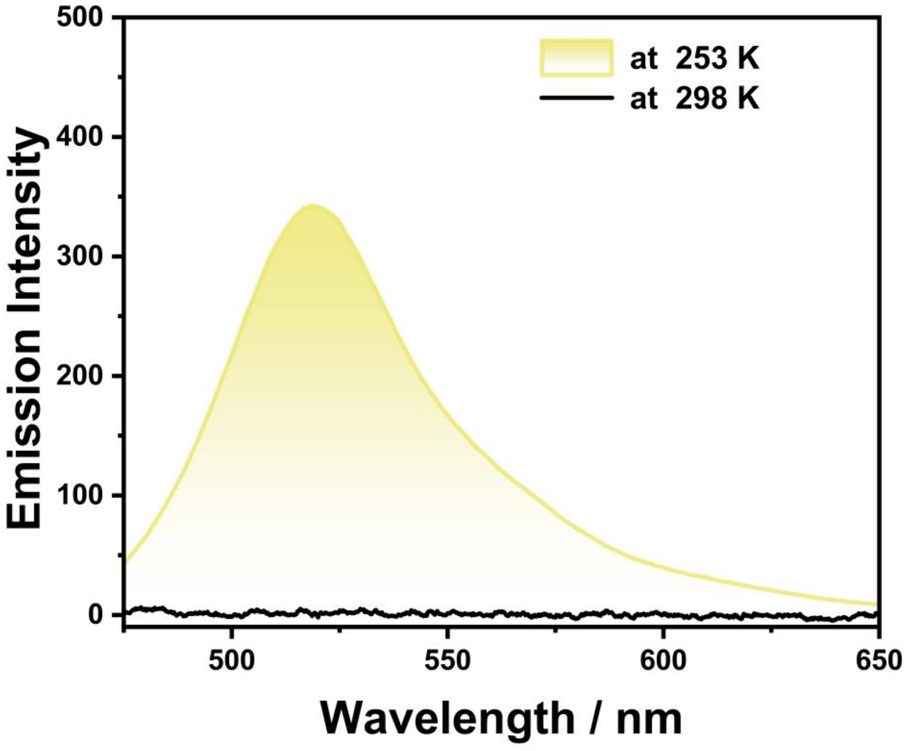

Supplementary Fig. 16. Delayed emission spectra (1 ms delay) of **NAPBP-BSA** conjugates at room temperature and frozen temperature.

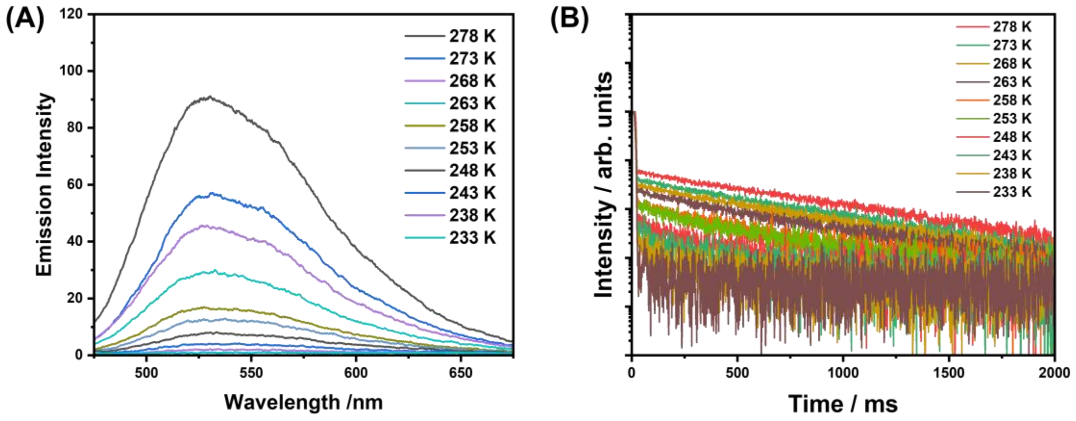

Supplementary Fig. 17. Temperature-dependent photophysical property of **NAPBP-BSA** conjugates. (A) Temperature-dependent delayed emission spectra (1 ms delay) of **NAPBP-BSA** conjugates. (B) Emission decay profile of **NAPBP-BSA** conjugates under 365 nm excitation monitored at 525 nm at different temperature.

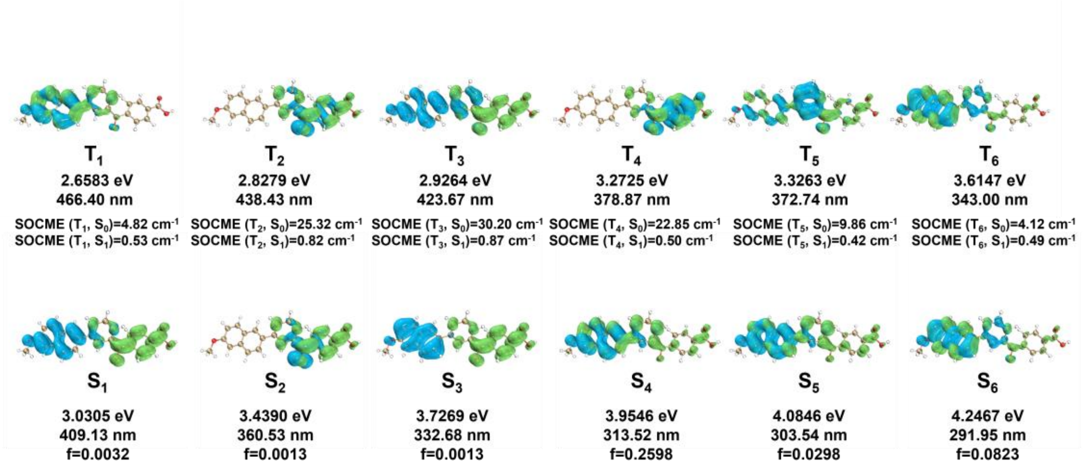

Supplementary Fig. 18. Iso-surface maps of electron-hole density difference of the S_n and T_n excited states of **NAPBP-COOH** molecule, where blue and green iso-surfaces correspond to hole and electron distributions, respectively, and the SOCME values of T_n-S_1 and T_n-S_0 transitions obtained by TD-DFT calculations using optimized ground-state geometry. The ground-state geometry was optimized by a DFT calculation using B3LYP functional and 6-31G (d, p) basis set; the singlet excited states and triplet excited states were calculated on Gaussian 16 program (Revision A.03) with B3LYP functional and 6-31G (d, p) basis set; spin-orbit coupling (SOC) matrix elements between the singlet excited states and triplet excited states were calculated with spin-orbit mean-field (SOMF) methods on ORCA 4.2.1 program with B3LYP functional and def2-TZVP(-f) basis set.

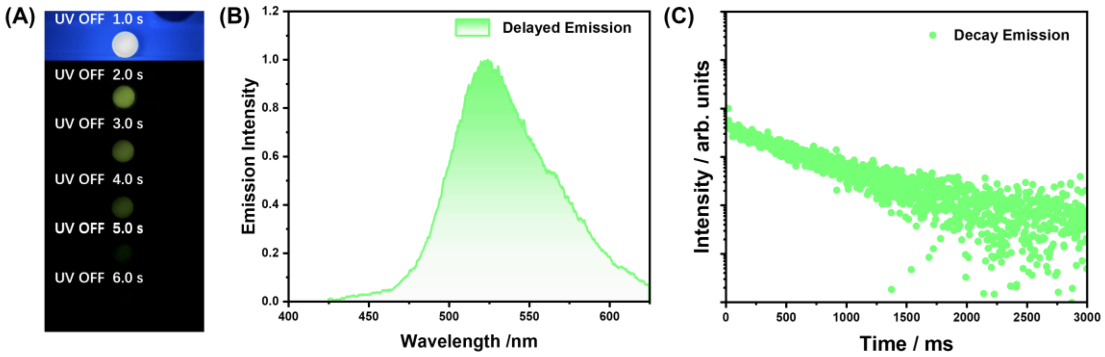

Supplementary Fig. 19. Photophysical property of **NAPBP-BSA** conjugates. (A) Photograph of the frozen 10 mg/mL **NAPBP-BSA** conjugates under UV and after switching off UV excitation. (B) Delayed emission spectra of the frozen **NAPBP-BSA** conjugates and (C) the corresponding emission decay curve under 365 nm excitation. Preparation of this sample: aqueous solution of **NAPBP-BSA** conjugates are immediately frozen by liquid nitrogen and then placed in -20 °C refrigerator for one day. The sample shows homogenous afterglow upon removing excitation source.

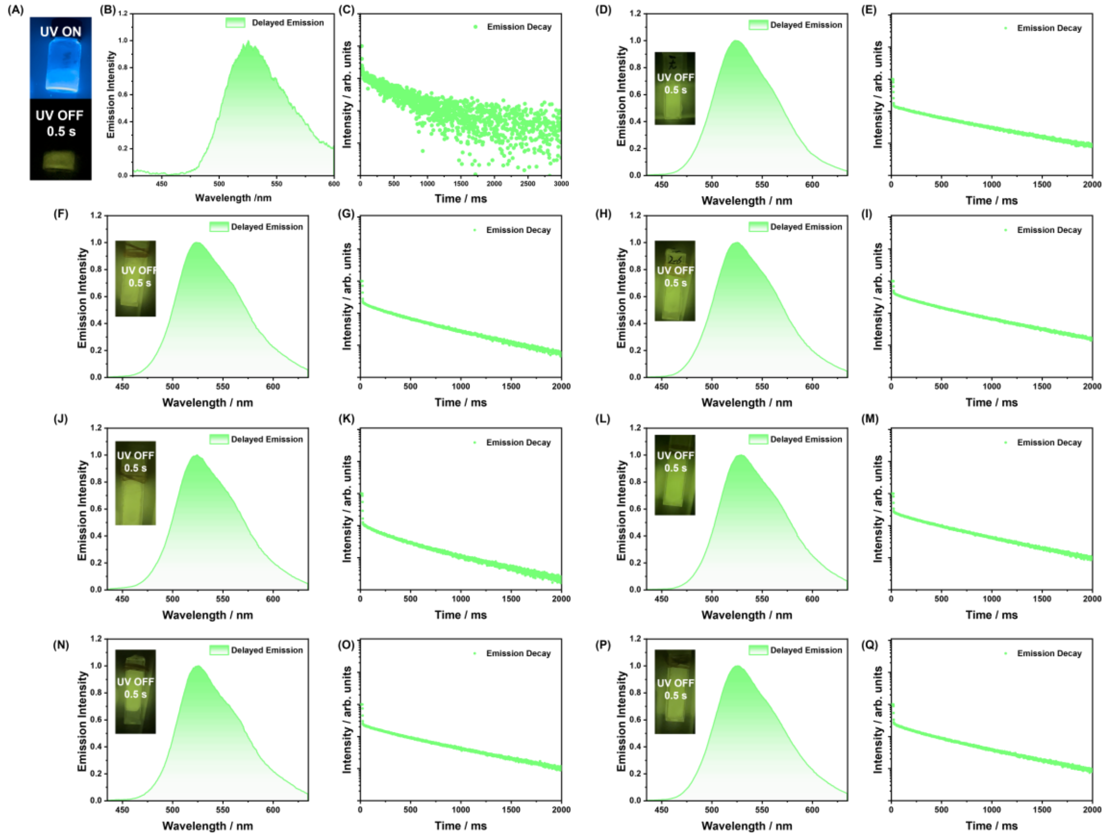

Supplementary Fig. 20. Photophysical property of **NAPBP-BSA** conjugates and control experiments. (A) Photograph of the 1 mg/mL frozen **NAPBP-BSA** conjugates under UV and after switching off UV. (B) Delayed emission spectra of the 1 mg/mL frozen **NAPBP-BSA** conjugates and (C) corresponding emission decay under 365 nm excitation. (D) Delayed emission spectra of the frozen **NAPBP-BSA** conjugates (10 mg/mL for BSA moiety) and (E) corresponding emission decay curve under 365 nm excitation. (F-K) Under 365 nm excitation, delayed emission spectra and corresponding emission decay curve of the frozen PEG-decorated **NAPBP-BSA** conjugates (10 mg/mL for BSA moiety); such conjugates are prepared through two steps (1) labeling BSA by **NAPBP-NHS** and (2) decorating the as-formed **NAPBP-BSA** conjugates by PEG-NHS (molecular weight 2000), followed by dialysis against deionized water. The molar feed ratios of BSA/**NAPBP-NHS**/PEG-NHS are 1/2/2 for (F, G), 1/2/6 for (H, I), and 1/2/18 for (J, K), respectively. Insets show afterglow photographs of the frozen samples. (L-Q) Under 365 nm excitation, delayed emission spectra and corresponding emission decay of the frozen PEG-decorated **NAPBP-BSA** conjugates (10 mg/mL for BSA moiety); such conjugates are prepared through two steps (1) labeling BSA by **NAPBP-NHS** and (2) decorating the as-formed **NAPBP-BSA** conjugates by PEG-NHS (molecular weight 5000), followed by dialysis against deionized water. The molar feed ratios of BSA/**NAPBP-NHS**/PEG-NHS are 1/2/2 for (L, M), 1/2/6 for (N, O), and 1/2/18 for (P, Q), respectively. Insets show afterglow photographs of the frozen samples.

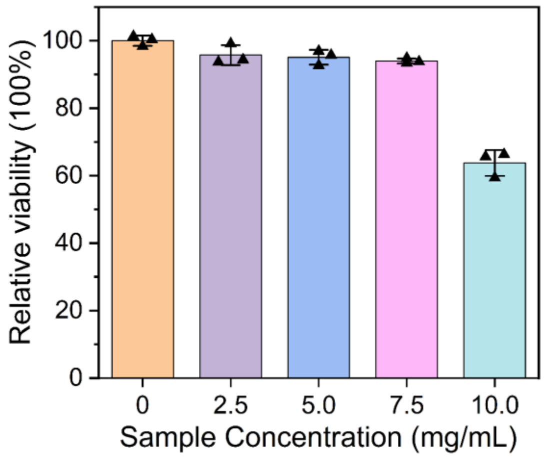

Supplementary Fig. 21. Cell viability assay of **NAPBP-BSA** conjugates. No data were excluded from the analyses.

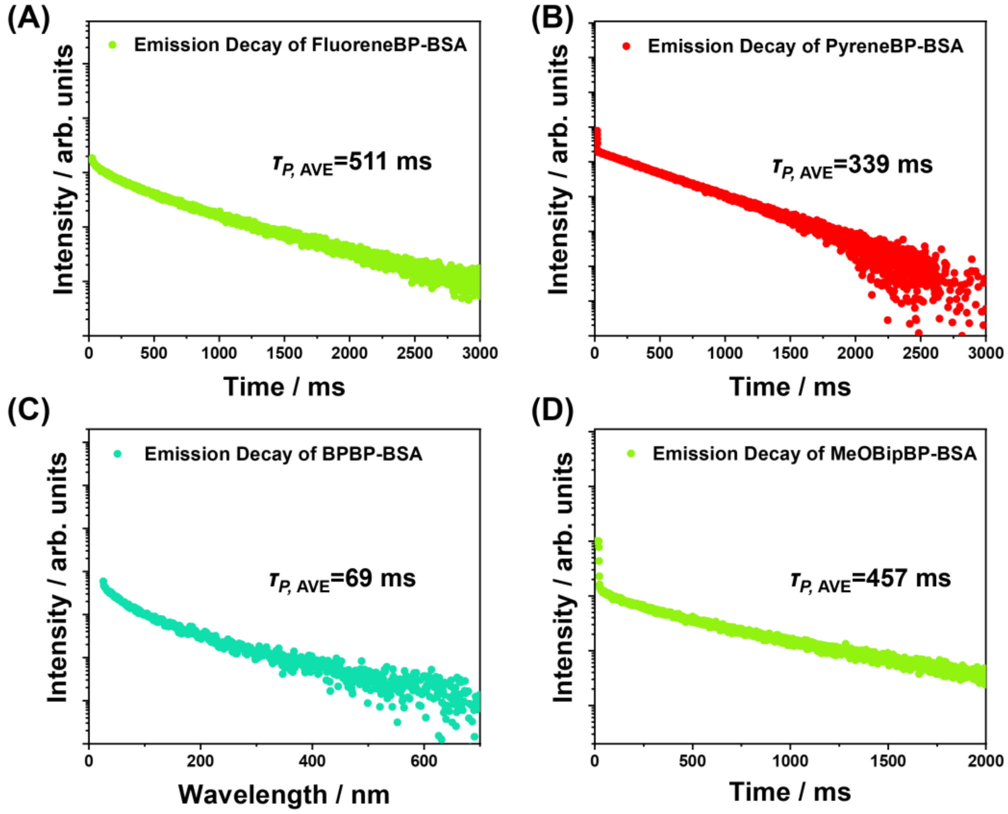

Supplementary Fig. 22. Photophysical property of luminophore-**BSA** conjugates. The emission decay curve of the frozen **FluoreneBP-BSA** conjugates (A), the frozen **PyreneBP-BSA** conjugates (B), the frozen **BPBP-BSA** conjugates (C), and the frozen **MeOBipBP-BSA** conjugates (D) under 365 nm excitation.

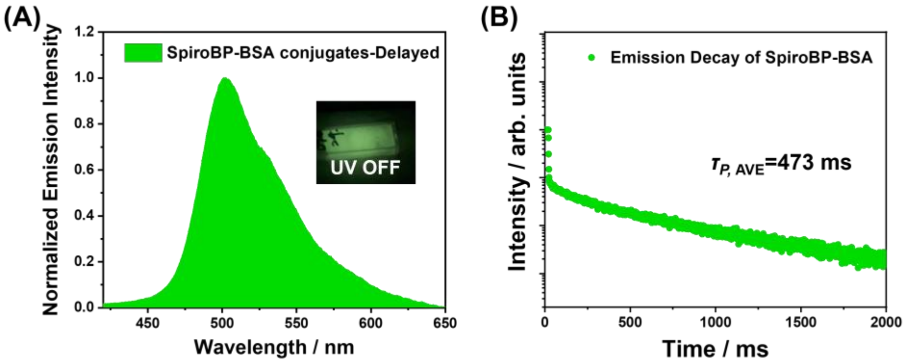

Supplementary Fig. 23. Photophysical property of **SpiroBP-BSA** conjugates. (A) Delayed emission spectra of the frozen **SpiroBP-BSA** conjugates and (B) the corresponding emission decay curve under 365 nm excitation.

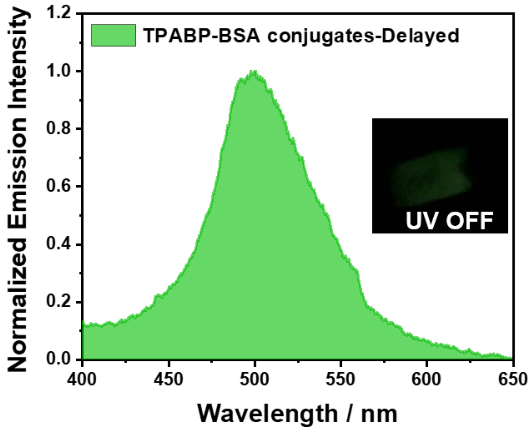

Supplementary Fig. 24. Delayed emission spectra of the frozen **TPABP-BSA** conjugate under 365 nm excitation.

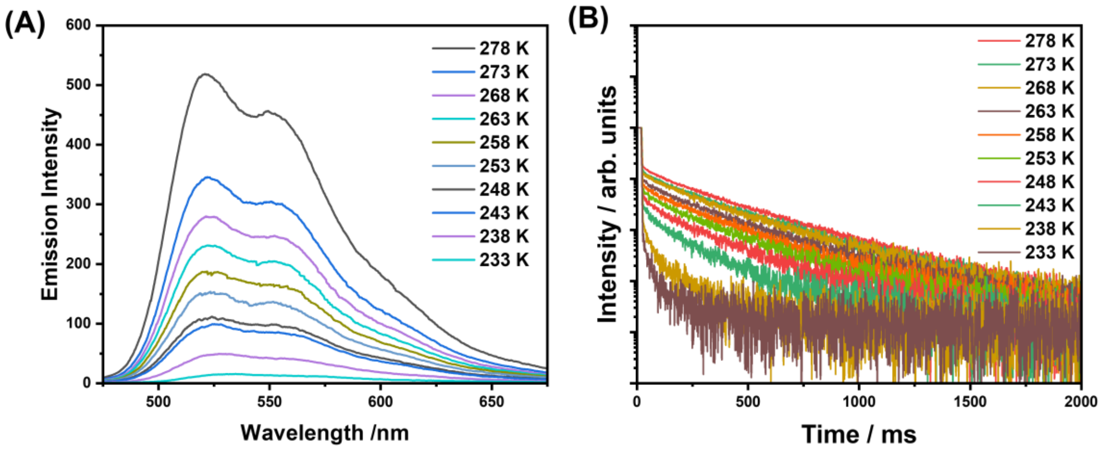

Supplementary Fig. 25. Temperature-dependent photophysical property of **2COOHNAP-diketone-BSA** conjugates. (A) Temperature-dependent delayed emission spectra (1 ms delay) of **2COOHNAP-diketone-BSA** conjugates. (B) Emission decay profile of **2COOHNAP-diketone-BSA** conjugates under 365 nm excitation monitored at 525 nm at different temperature.

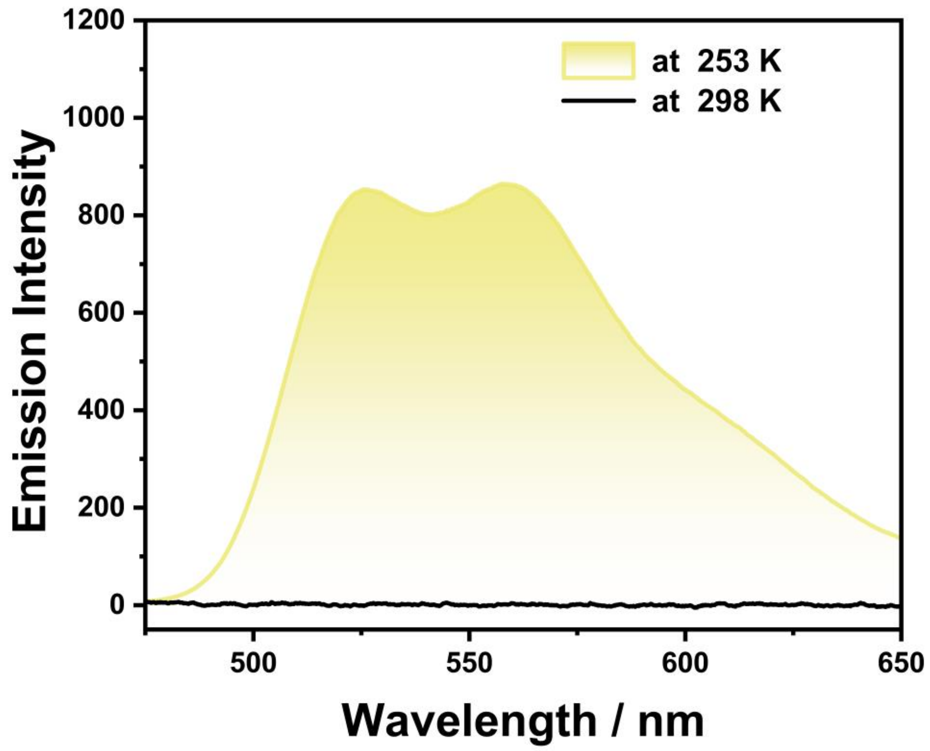

Supplementary Fig. 26. Delayed emission spectra (1 ms delay) of **2COOHNAP-diketone-BSA** conjugates at room temperature and frozen temperature.

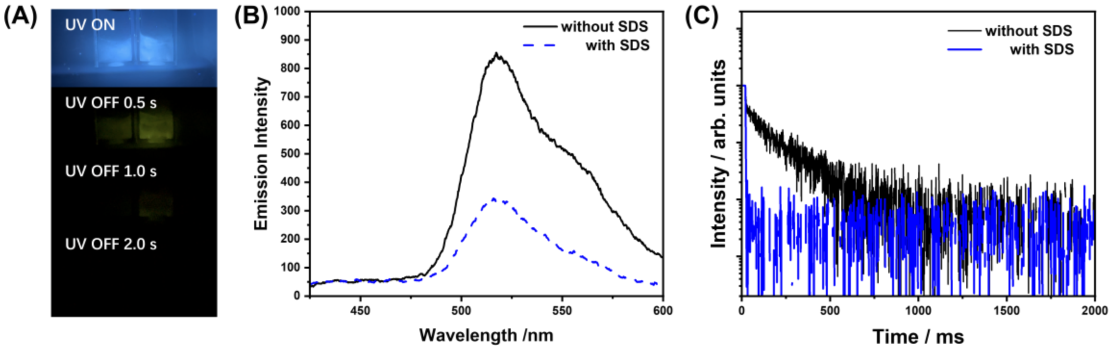

Supplementary Fig. 27. Photophysical property of **2COOHNAP-diketone-BSA** conjugate. (A) Photographs of the frozen solution of **2COOHNAP-diketone-BSA** conjugate with SDS (left cell) under UV and after switching off UV excitation; photographs of the **2COOHNAP-diketone-BSA** conjugates without SDS (right cell) for comparison. (B) Delayed emission spectra of the frozen samples with SDS and without SDS, and (C) the corresponding emission decay curve under 365 nm excitation.

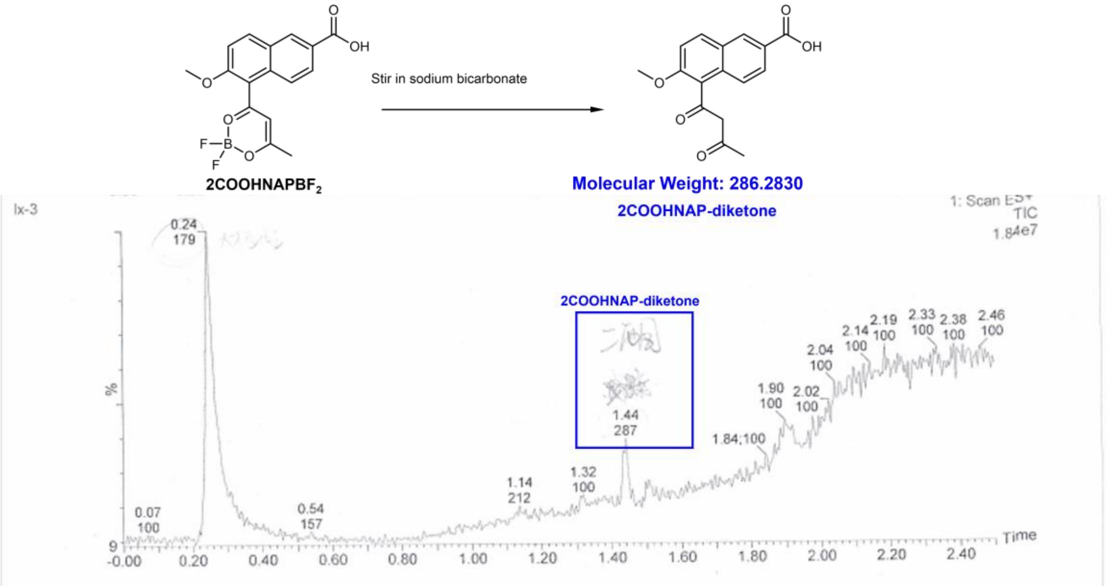

Supplementary Fig. 28. HPLC-MS profile of **$2COOHNAPBF_2$** in sodium bicarbonate solution.

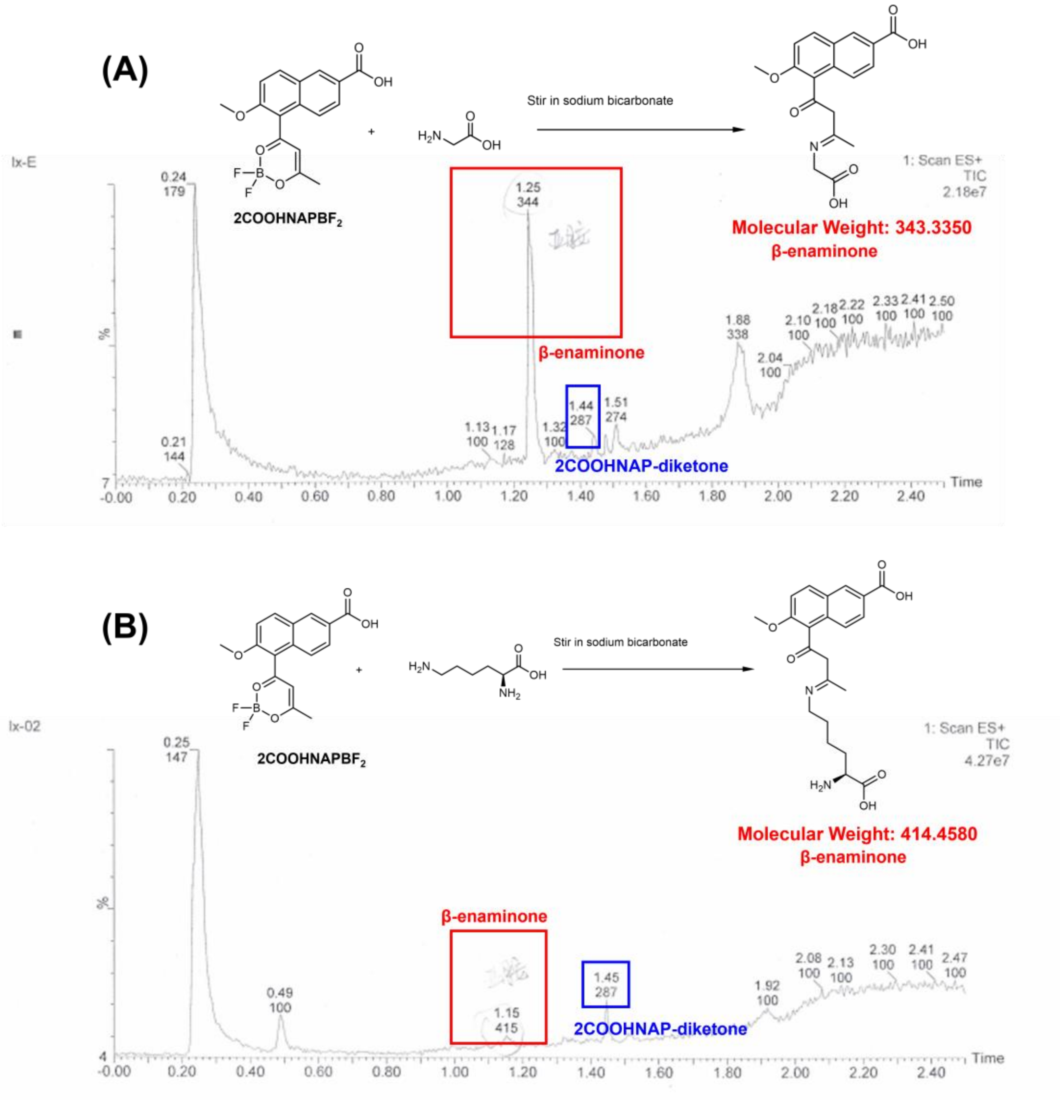

Supplementary Fig. 29. HPLC-MS profiles of (A) the reaction mixture of **2COOHNAPBF2** and Gly in sodium bicarbonate solution, (B) the reaction mixture of **2COOHNAPBF2** and Lys in sodium bicarbonate solution.

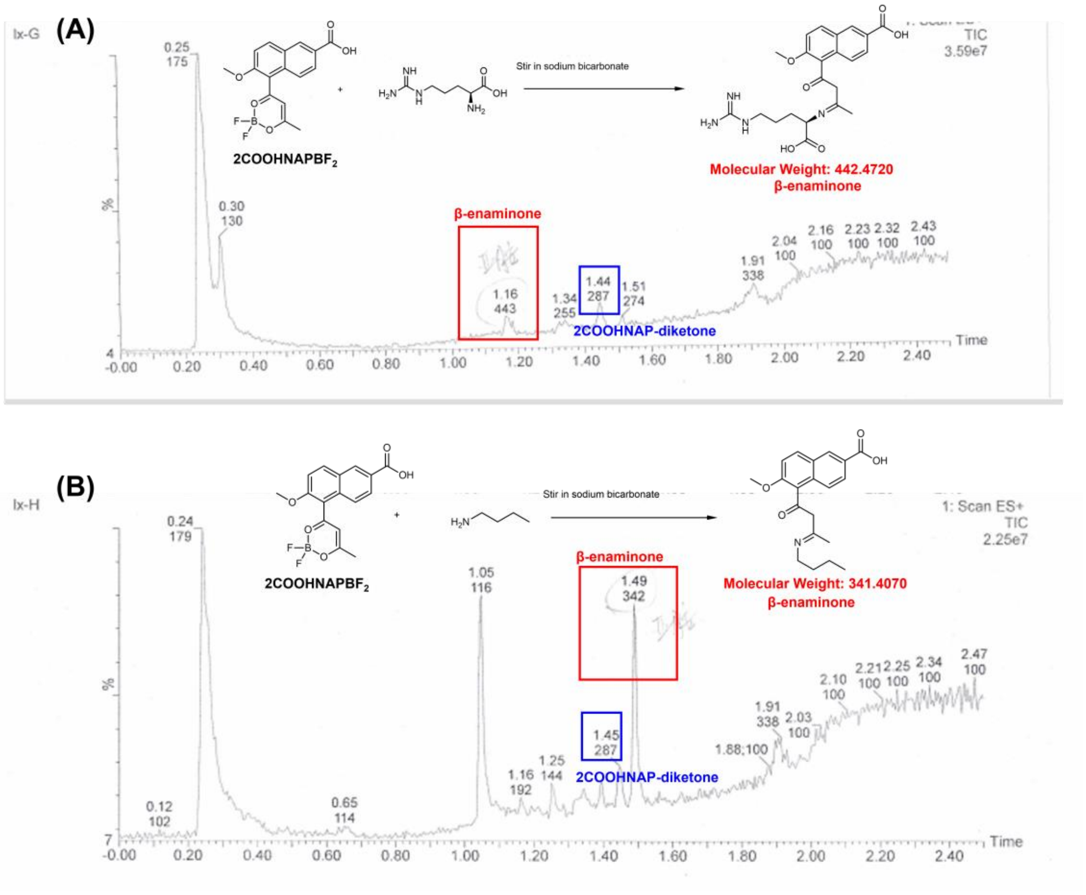

Supplementary Fig. 30. HPLC-MS profiles of (A) the reaction mixture of **2COOHNAPBF₂** and L-Arg in sodium bicarbonate solution, (B) the reaction mixture of **2COOHNAPBF₂** and n-butylamine in sodium bicarbonate solution.

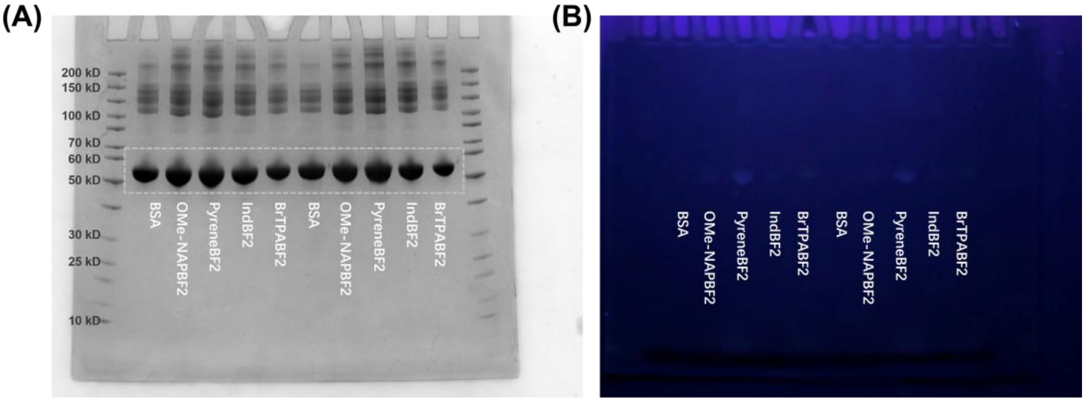

Supplementary Fig. 31. SDS-PAGE analysis of BSA and **BF$_2$bdk**-BSA conjugates. The results of SDS-PAGE gel have also been shown in Figure 4d in the main text, from which we can see the fluorescence from **BF$_2$bdk**-BSA conjugates and some background fluorescence. In order to remove the background of the SDS-PAGE gel, we use low-power UV excitation source, so that the fluorescence signals of **BF$_2$bdk**-BSA conjugates are weak in this figure. To give a clear view of the fluorescence from **BF$_2$bdk**-BSA conjugates, we still use the original Figure 4d in the main text. Actually, we have tried for many times to capture the photographs of SDS-PAGE gel under UV lamp. The photographs in the main text and supplementary information are the best results at the current stage.

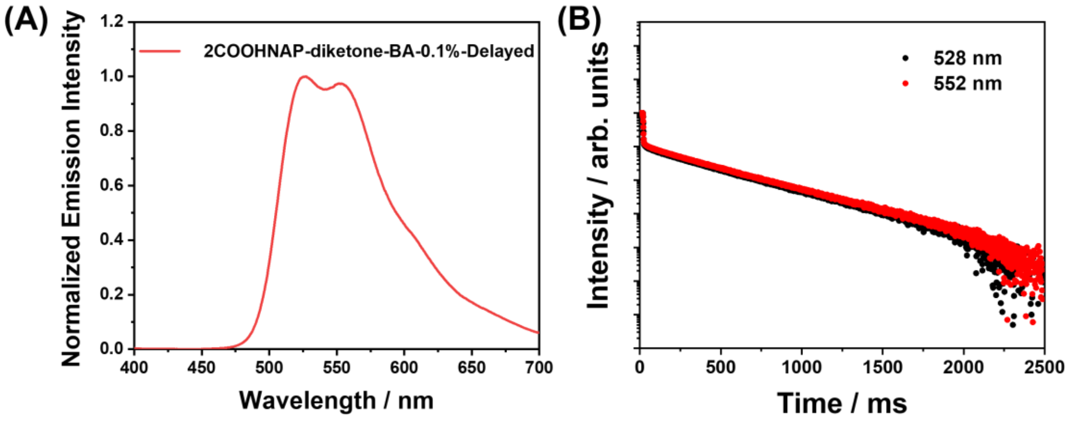

Supplementary Fig. 32. Photophysical property of **2COOHNAP-diketone**-BA-0.1% sample. (A) Room-temperature delayed emission spectra of the **2COOHNAP-diketone**-BA-0.1% powder and (B) the corresponding emission decay curve under 365 nm excitation.

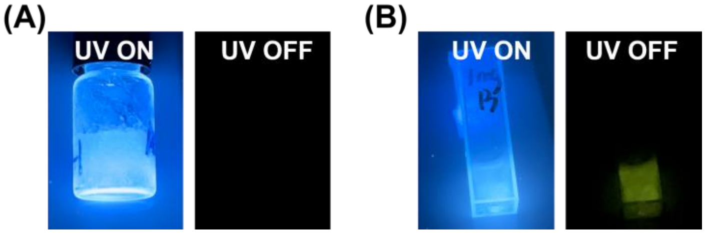

Supplementary Fig. 33. Photographs of **2COOHNAP-diketone-PEG** and **2COOHNAP-diketone-BSA** conjugates. (A) Photographs of the frozen **2COOHNAP-diketone-PEG** aqueous solution at 13 mg/mL under UV and after switching off UV excitation; (B) Photographs of the afterglow ice of **2COOHNAP-diketone-BSA** conjugates at 1 mg/mL for comparison.

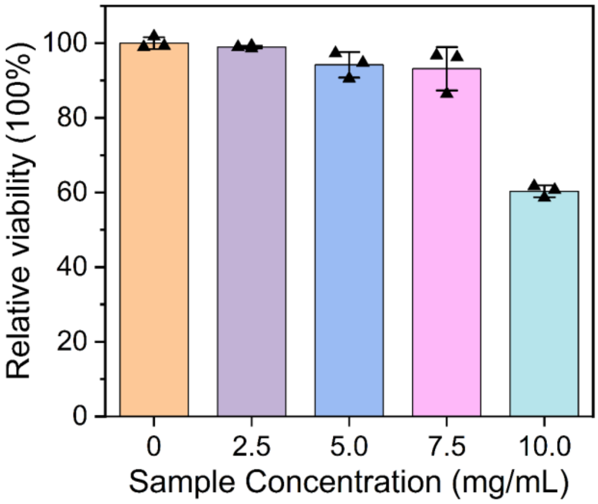

Supplementary Fig. 34. Cell viability assay of **2COOHNAP-diketone-BSA** conjugates. No data were excluded from the analyses.

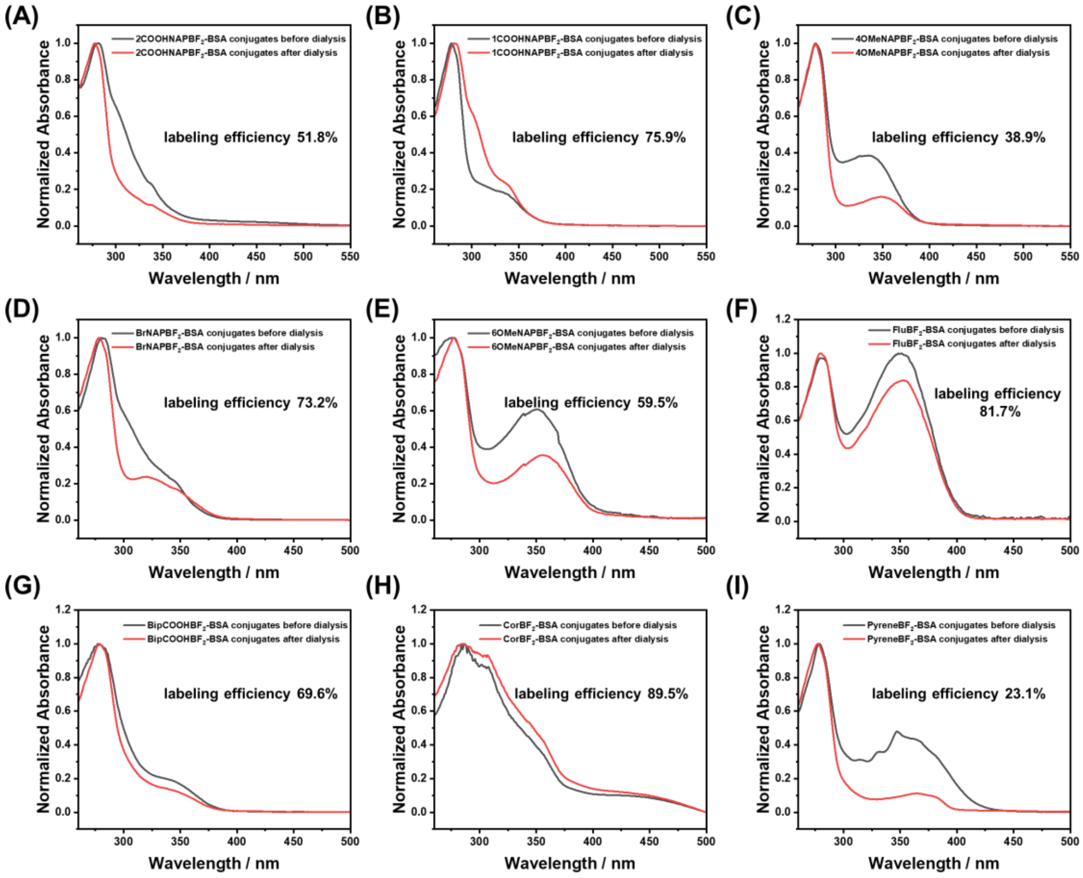

Supplementary Fig. 35. UV-vis absorption spectra of **BF_2bdk-BSA** conjugates. UV-vis absorption spectra of the (A) **2COOHNAPBF_2-BSA** conjugates, (B) **1COOHNAPBF_2-BSA** conjugates, (C) **4OMeNAPBF_2-BSA** conjugates, (D) **BrNAPBF_2-BSA** conjugates, (E) **6OMeNAPBF_2-BSA** conjugates, (F) **FluBF_2-BSA** conjugates, (G) **BipCOOHBF_2-BSA** conjugates, (H) **CorBF_2-BSA** conjugates and (I) **PyreneBF_2-BSA** conjugates before and after dialysis. Before dialysis, the UV-vis absorption band in the lower-energy region corresponds to the combined signals of free luminophore and conjugated luminophore, while after dialysis, the UV-vis absorption band corresponds to the conjugated luminophore. Here we estimate the labeling efficiency from the ratio of absorbance (after dialysis)/absorbance (before dialysis) in the lower-energy region.

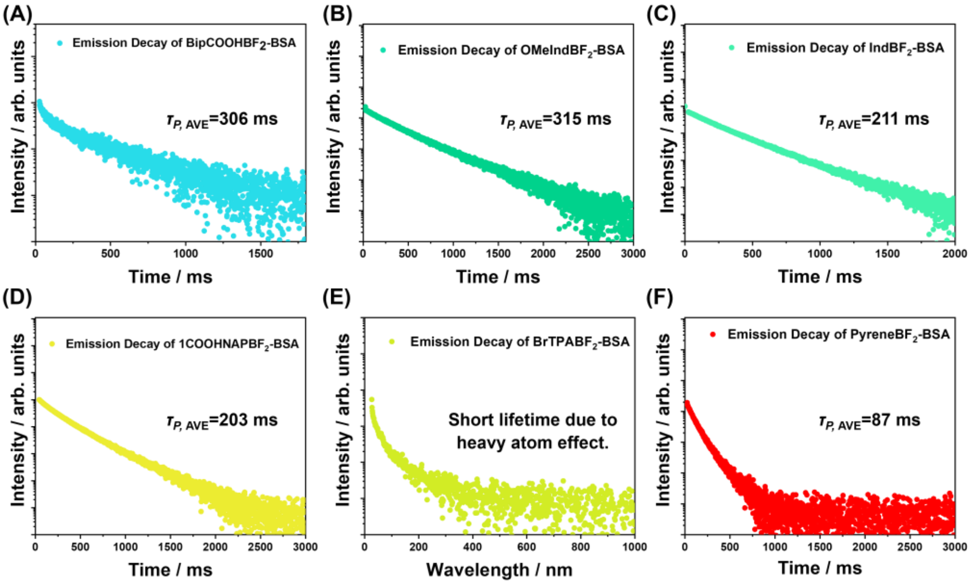

Supplementary Fig. 36. Photophysical property of **BF2bdk-BSA** conjugates. Emission decay curve of the frozen **BipCOOHBF2-BSA** conjugates (A), the frozen **OMeIndBF2-BSA** conjugates (B), the frozen **IndBF2-BSA** conjugates (C), the frozen **1COOHNAPBF2-BSA** conjugates (D), the frozen **BrTPABF2-BSA** conjugates (E), and the frozen **PyreneBF2-BSA** conjugates (F) under 365 nm excitation.

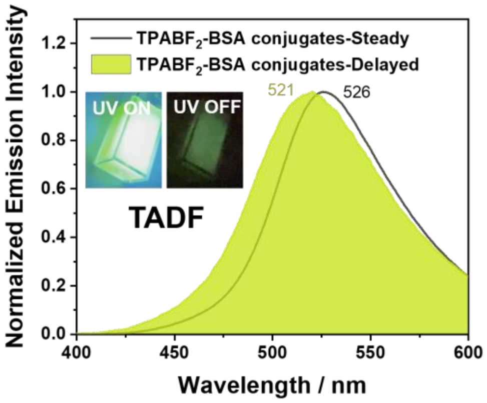

Supplementary Fig. 37. Steady-state and delayed emission spectra of the frozen **TPABF2-BSA** conjugates under 365 nm excitation.

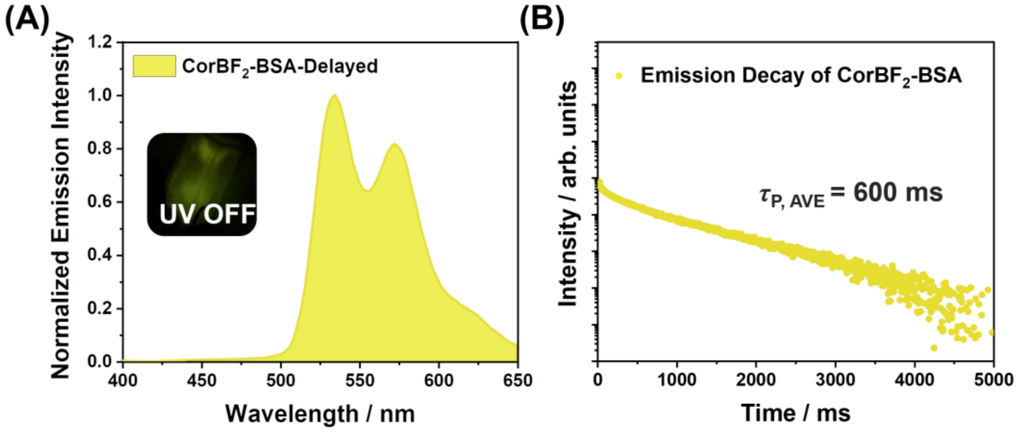

Supplementary Fig. 38. (A) Delayed emission spectra of the frozen **$CorBF_2$-BSA** conjugates and (B) the corresponding emission decay curve under 365 nm excitation.

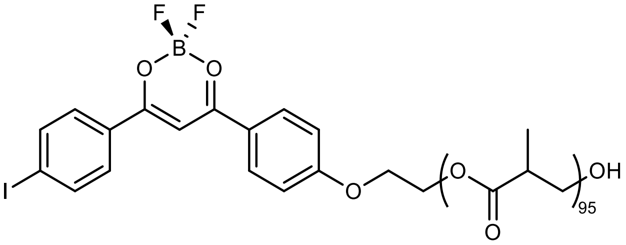

τ=4.82 ms, nanospheres in aqueous medium

Nat. Mater. **2009**, 8, 747–751.

DPhCzT

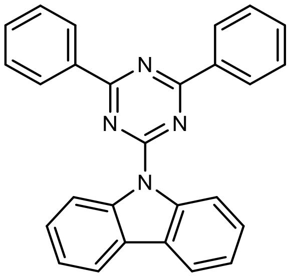

τ=0.46 s, microcrystals in aqueous medium

Nat. Mater. **2015**, 14, 685–690.

CPhCz

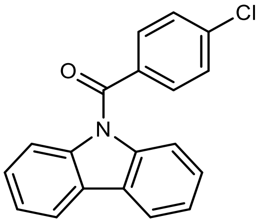

F127

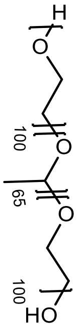

τ=0.65 s, nanospheres in aqueous medium

Adv. Mater. **2017**, 29, 1701244.

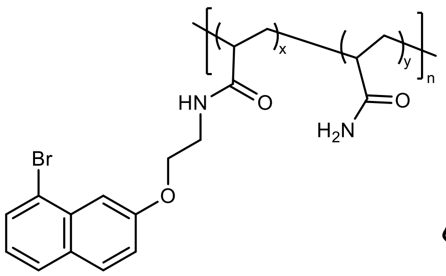

Poly-BrNP

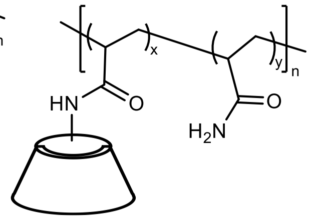

Poly-β-CD

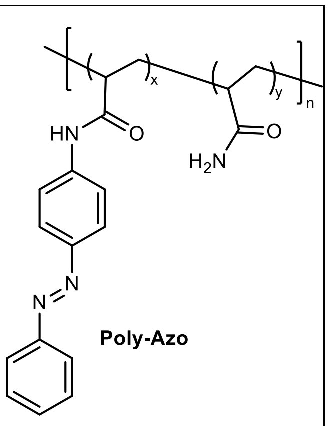

Poly-Azo

τ=0.56 ms in aqueous medium

Angew. Chem. Int. Ed. **2014**, 53, 14149–14152.

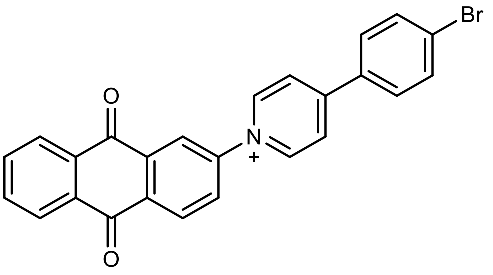

AQPY

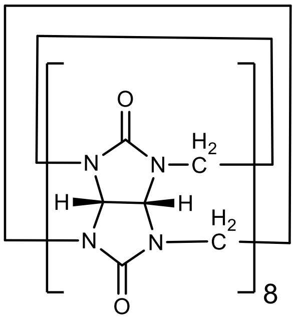

cucurbit[8]uril (CB[8])

τ=376.53 μs in aqueous medium

J. Am.Chem. Soc. **2021**, 143, 13887-13894.

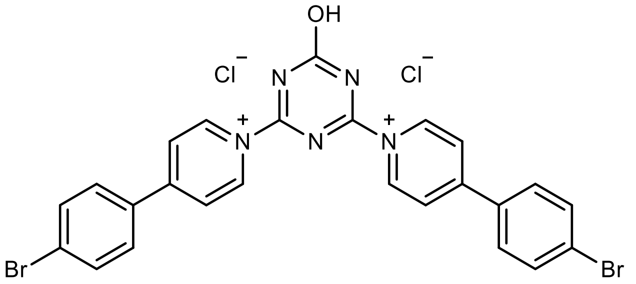

TBP

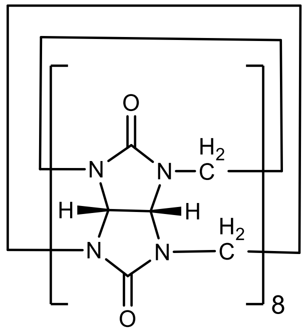

cucurbit[8]uril (CB[8])

τ=0.19 ms in aqueous medium

Angew. Chem. Int. Ed. **2020**, 59, 9928-9933.

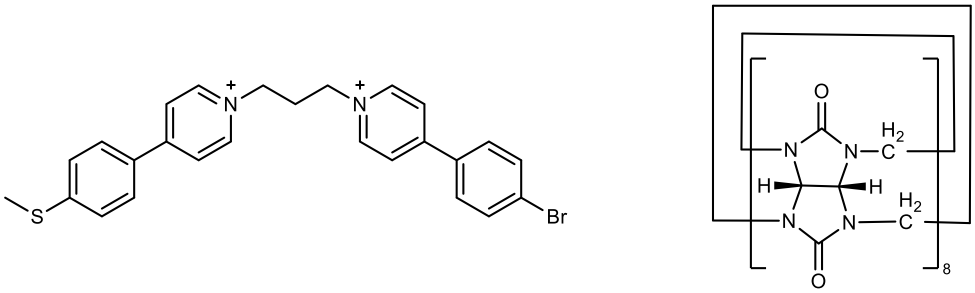

Compound 1 **cucurbit[8]uril (CB[8])**

τ=133 μs in aqueous medium
Adv. Mater. **2021**, 2007476.

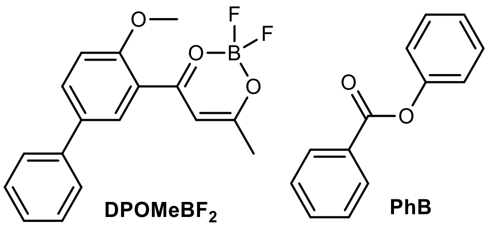

DPOMeBF$_2$ **PhB**

τ=250 ms
microcrystals in aqueous suspension
Angew. Chem. Int. Ed. **2021**, 60, 17138–17147.

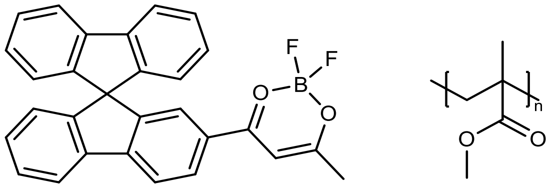

SpiroBF$_2$ **PMMA**

τ=1.49 s, emulsion in aqueous medium
Adv. Opt. Mater. **2022**, 10, 2201502.

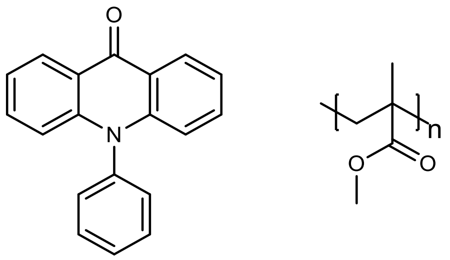

Compound 1 **PMMA**

τ=336 ms, emulsion in aqueous medium
Chem. Commun., **2023**, 59, 12302–12305.

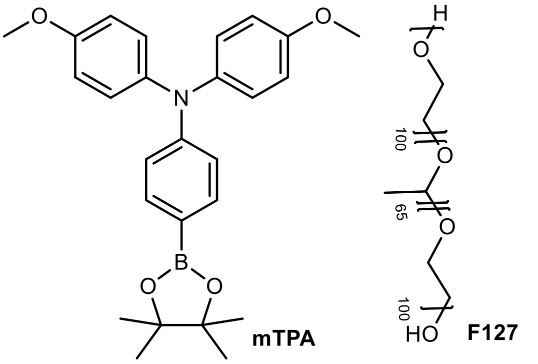

mTPA **F127**

τ=9.0 μs, nanospheres in aqueous medium
Adv. Mater. **2020**, 32, 2006752.

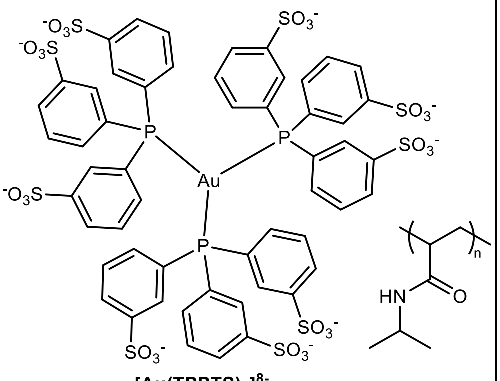

[Au(TPPTS)$_3$]$^{8-}$ **PNIPAM**

τ=2.92 ms in hydrogel
Langmuir **2010**, 26, 15523–15531.

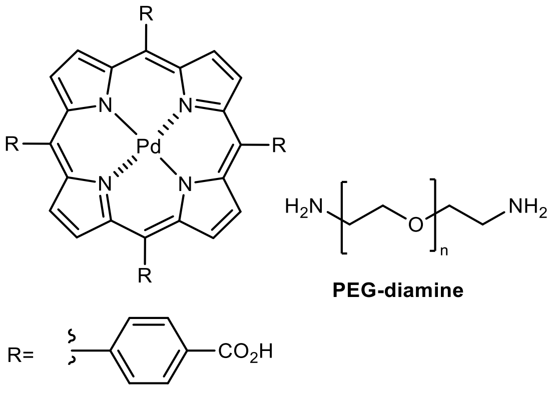

PEG-diamine

Pd-mTCPP τ=55 ms in hydrogel
Adv. Healthc Mater. **2014**, 3, 890–890.

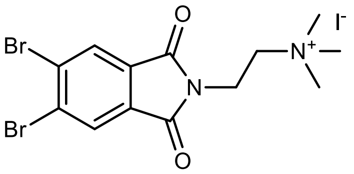

cationic phthalimide derivative (CPthBr)

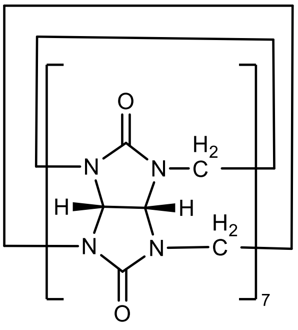

cucurbit[7]uril (CB[7])

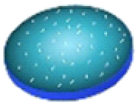

laponite (LP)

τ=1.62 ms in hydrogel

Angew. Chem. Int. Ed. **2021**, 60, 19720–19724.

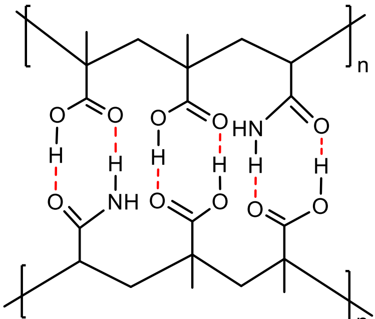

τ=50 ms in hydrogel

Adv. Mater. **2023**, 35, 2300244.

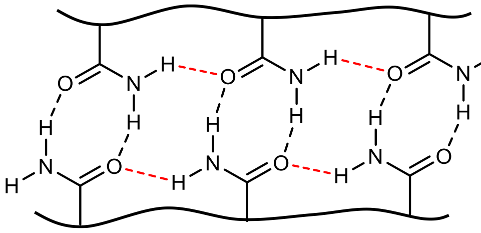

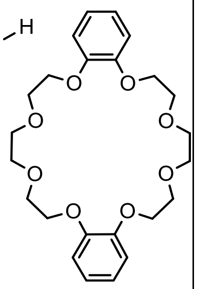

τ=342 ms in hydrogel

J. Am. Chem. Soc. **2023**, 145, 3763–3773.

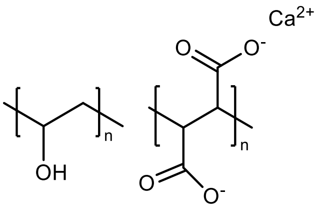

PVA **PMACa**

τ=13.42 ms in hydrogel

Adv. Funct. Mater. **2023**, 2308420.

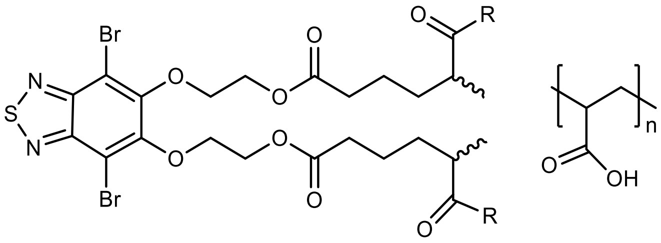

τ=1.71 ms in hydrogel

Angew. Chem. Int. Ed. **2024**, 63, e202401331.

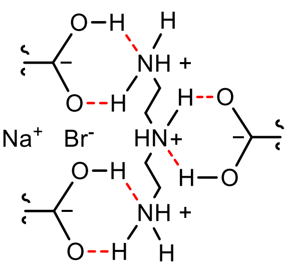

τ=73.44 ms in hydrogel

Adv. Opt. Mater. **2024**, 2303330.

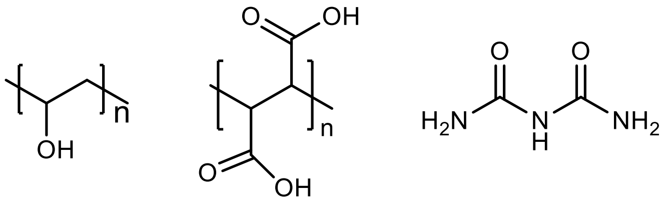

PVA **PMAc** **Biuret**

Hydrogen bonds and synergy induce rigid conformation and long RTP, τ=782.8 ms

Adv. Funct. Mater. **2024**, 2408821.

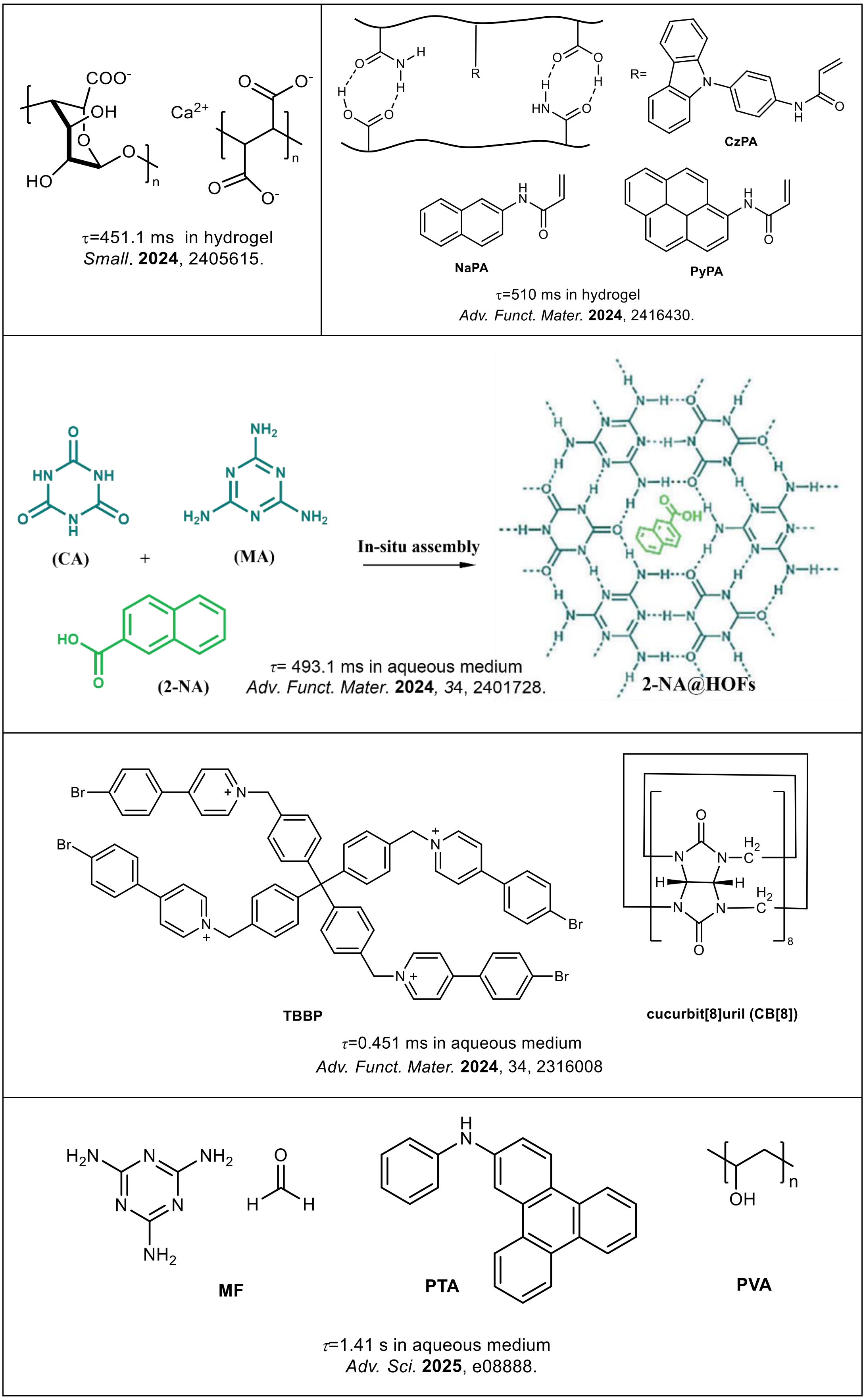

Supplementary Fig. 39. Selected examples of afterglow materials in aqueous systems including supramolecular assemblies, suspension, emulsion and hydrogels.

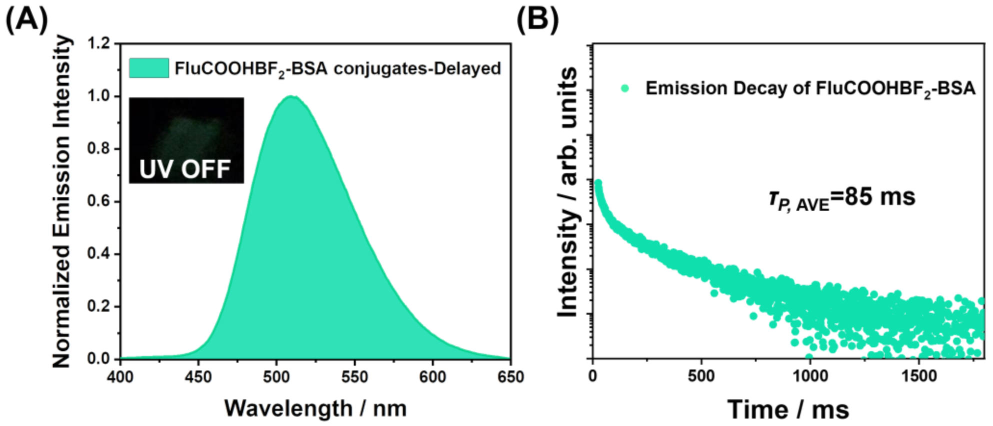

Supplementary Fig. 40. Photophysical property of **FluCOOHBF2-BSA** conjugates. (A) Delayed emission spectra of the frozen **FluCOOHBF2-BSA** conjugates and (B) the corresponding emission decay curve under 365 nm excitation.

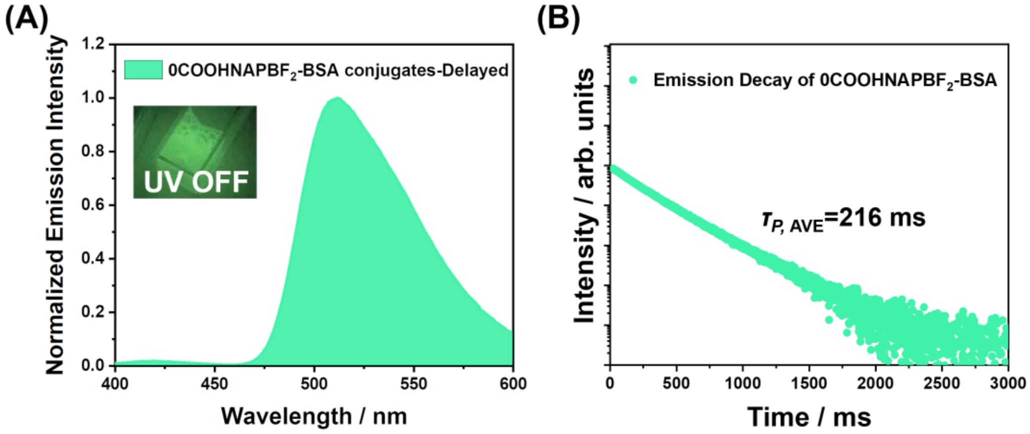

Supplementary Fig. 41. Photophysical property of **0COOHNAPBF2-BSA** conjugates. (A) Delayed emission spectra of the frozen **0COOHNAPBF2-BSA** conjugates and (B) the corresponding emission decay curve under 365 nm excitation.

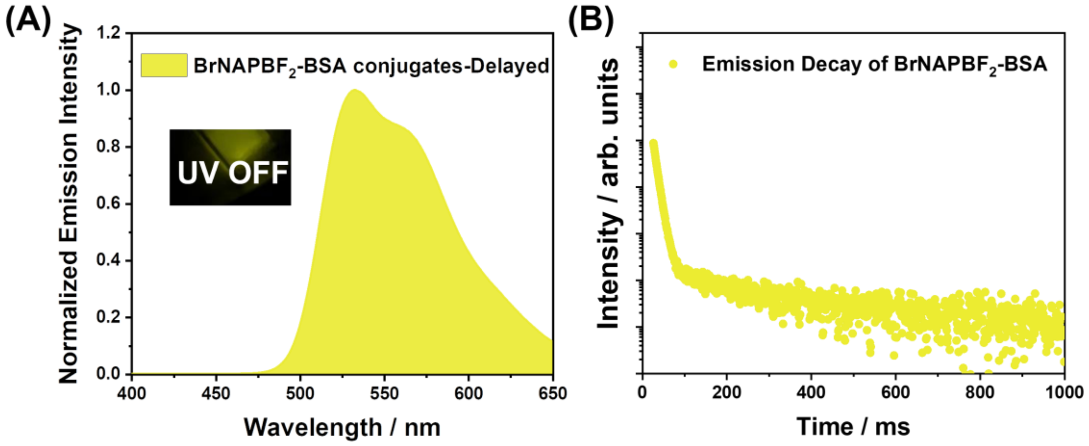

Supplementary Fig. 42. Photophysical property of **BrNAPBF2-BSA** conjugates. (A) Delayed emission spectra of the frozen **BrNAPBF2-BSA** conjugates and (B) the corresponding emission decay curve under 365 nm excitation.

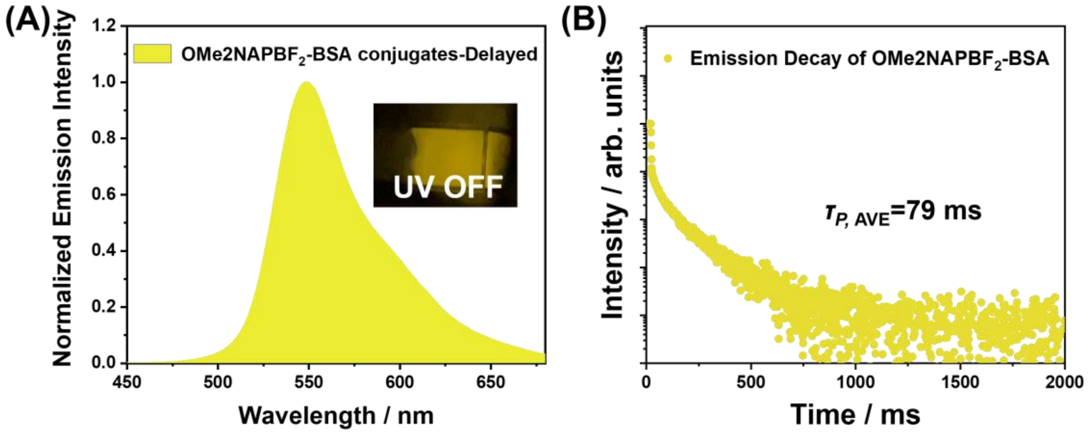

Supplementary Fig. 43. Photophysical property of **OMe2NAPBF2-BSA** conjugates. (A) Delayed emission spectra of the frozen **OMe2NAPBF2-BSA** conjugates and (B) the corresponding emission decay curve under 365 nm excitation.

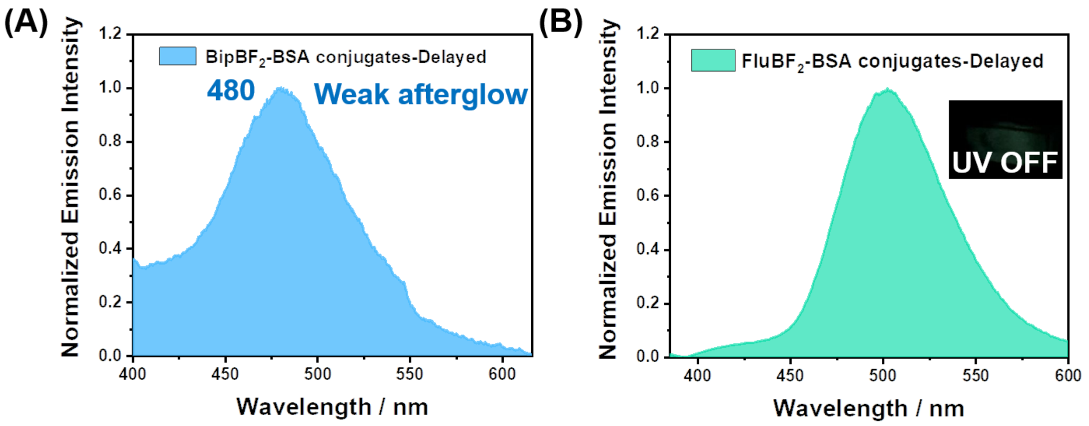

Supplementary Fig. 44. Photophysical property of **BipBF2-BSA** conjugates. (A) Delayed emission spectra of the frozen **BipBF2-BSA** conjugates (A) and the frozen **FluBF2-BSA** conjugates (B) under 365 nm excitation.

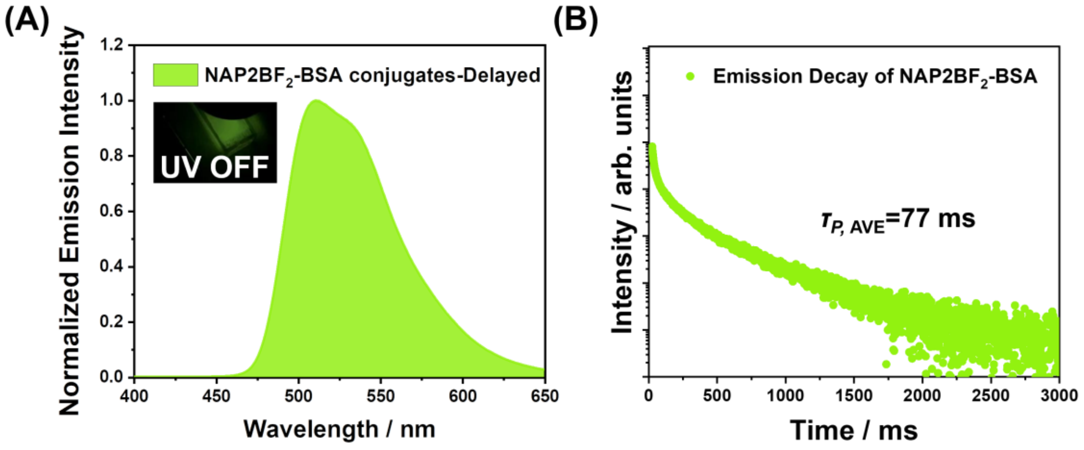

Supplementary Fig. 45. Photophysical property of **NAP2BF2-BSA** conjugates. (A) Delayed emission spectra of the frozen **NAP2BF2-BSA** conjugates and (B) the corresponding emission decay curve under 365 nm excitation.

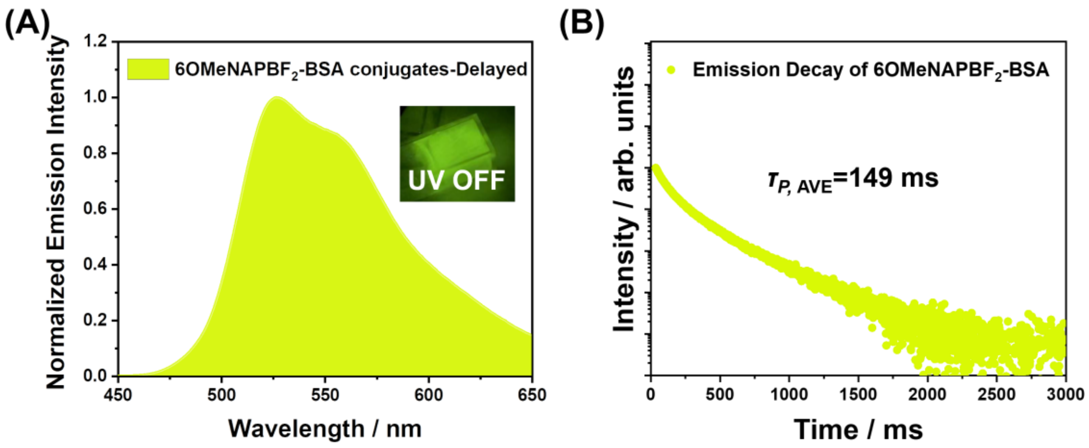

Supplementary Fig. 46. Photophysical property of **6OMeNAPBF2-BSA** conjugates. (A) Delayed emission spectra of the frozen **6OMeNAPBF2-BSA** conjugates and (B) the corresponding emission decay curve under 365 nm excitation.

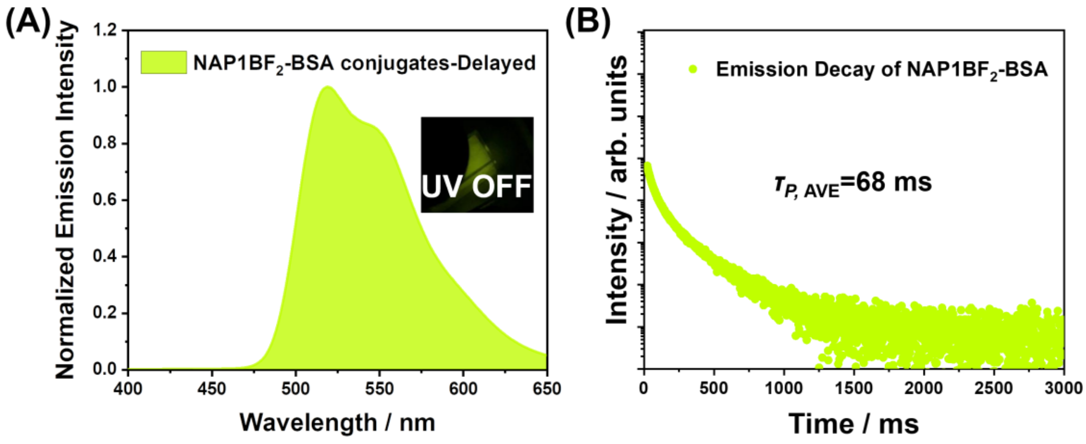

Supplementary Fig. 47. Photophysical property of **NAP1BF$_2$-BSA** conjugates. (A) Delayed emission spectra of the frozen **NAP1BF$_2$-BSA** conjugates and (B) the corresponding emission decay curve under 365 nm excitation.

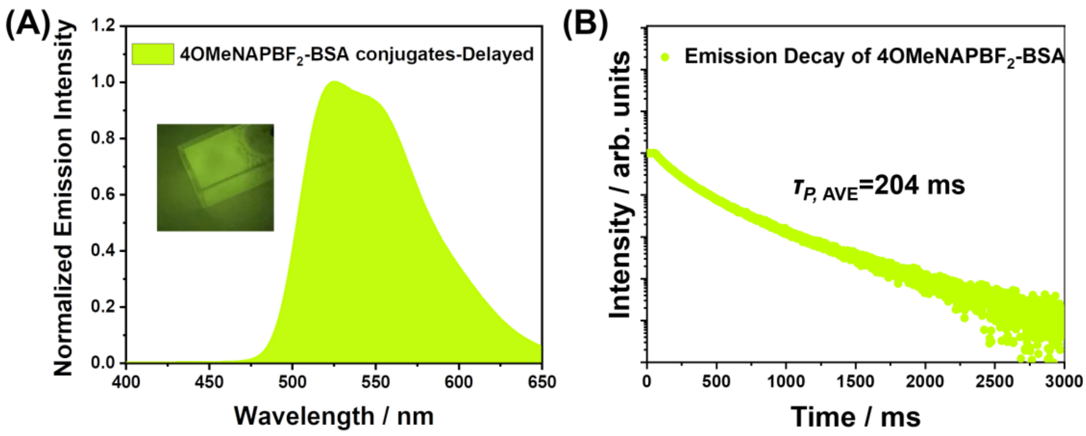

Supplementary Fig. 48. Photophysical property of **4OMeNAPBF$_2$-BSA** conjugates. (A) Delayed emission spectra of the frozen **4OMeNAPBF$_2$-BSA** conjugates and (B) corresponding emission decay curve under 365 nm excitation.

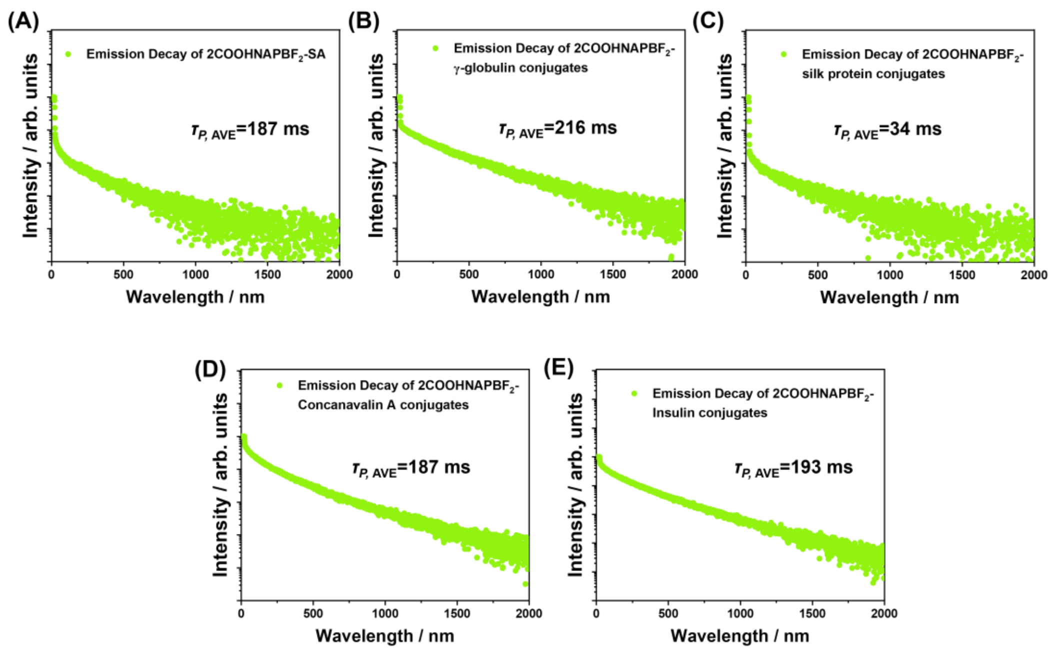

Supplementary Fig. 49. Photophysical property of **2COOHNAPBF2**-protein conjugates. Emission decay curve of the frozen **2COOHNAPBF2-SA** (A), the **2COOHNAPBF2-γ-globulin** (B), **2COOHNAPBF2-silk protein** (C), **2COOHNAPBF2-concanavalin A** (D), **2COOHNAPBF2-insulin** (E) conjugates upon 365 nm excitation.

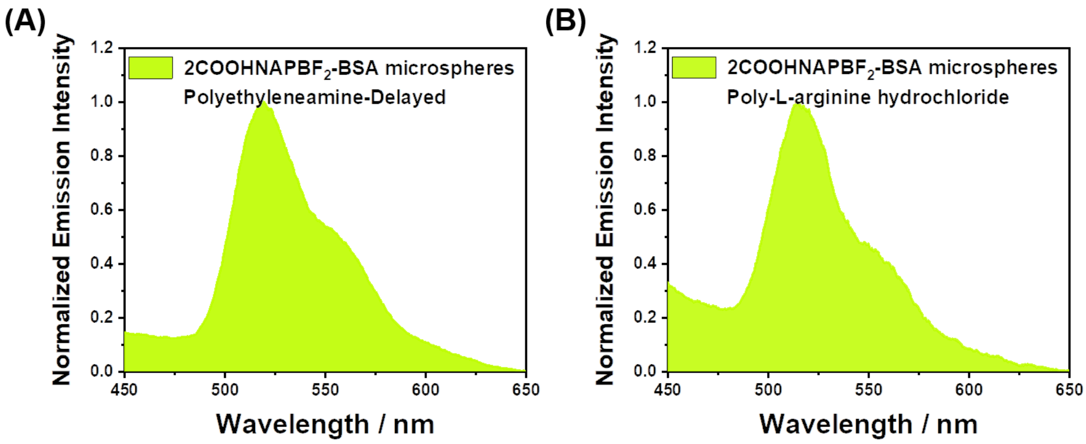

Supplementary Fig. 50. Photophysical property of protein-decorated microspheres. Delayed emission spectra of the frozen organic afterglow **2COOHNAPBF2-BSA**-decorated microspheres prepared using (A) polyethyleneimine and (B) poly-L-arginine as electrostatic binder.

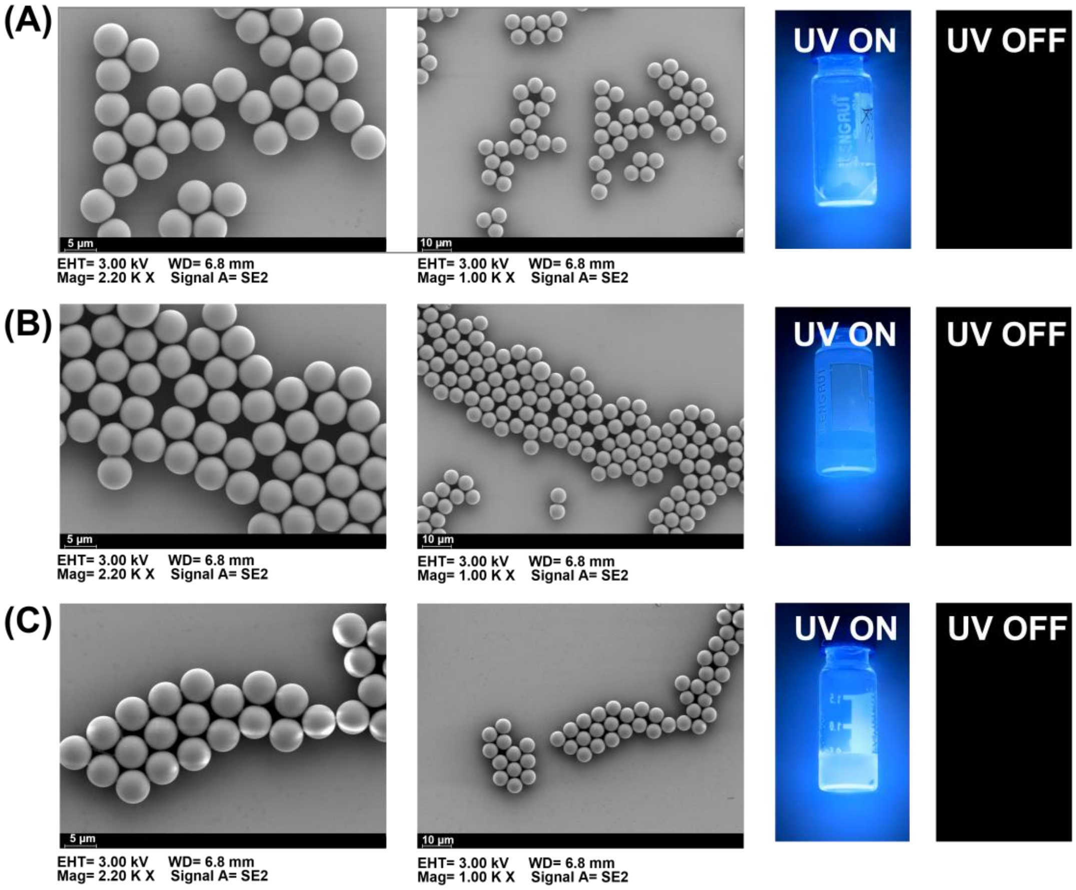

Supplementary Fig. 51. SEM images and UV-ON/OFF photographs of the samples in control experiment. (A) pristine BSA was used in the place of **2COOHNAPBF$_2$-BSA** (polyethyleneamine, electrostatic binder), (B) pristine BSA was used in the place of **2COOHNAPBF$_2$-BSA** (poly-L-arginine, electrostatic binder), and (C) the mixture of BSA and microspheres (without polyelectrolyte binder).

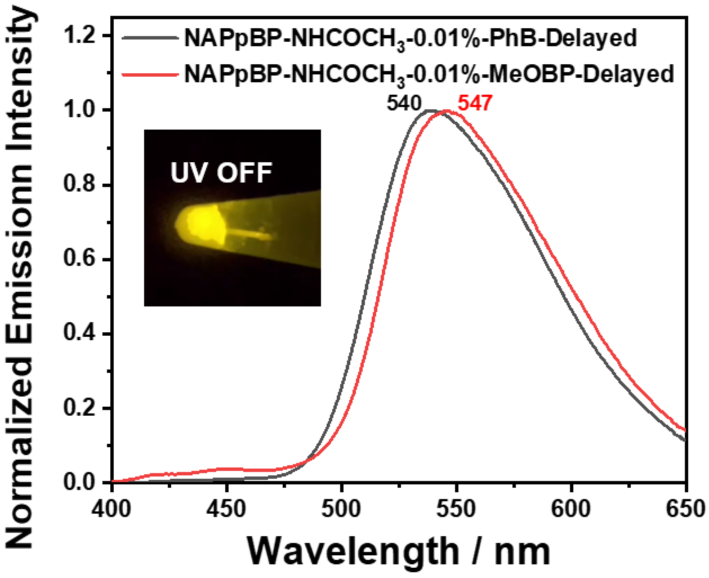

Supplementary Fig. 52. Photophysical property of **NAPpBP-NHCOCH3**-matrix-0.01% samples. Delayed emission spectra of **NAPpBP-NHCOCH3**-PhB-0.01% (A) and **NAPpBP-NHCOCH3**-MeOBP-0.01% melt-cast samples (B) under 365 nm excitation. PhB and MeOBP represent phenyl benzoate and 4-methoxybenzophenone matrices, respectively.

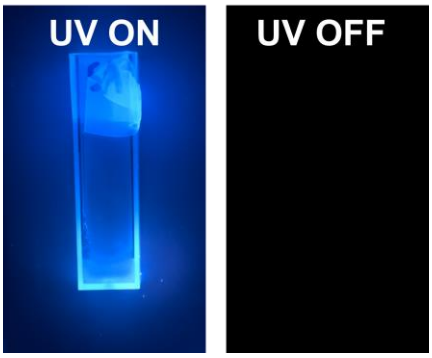

Supplementary Fig. 53. Photographs of the frozen SA solution following identical experimental protocols under UV and after switching off UV excitation.

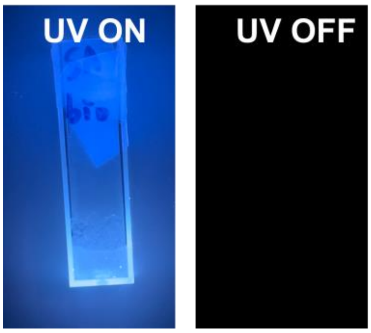

Supplementary Fig. 54. Photographs of the frozen solution of Biotin-SA complex under UV and after switching off UV excitation.

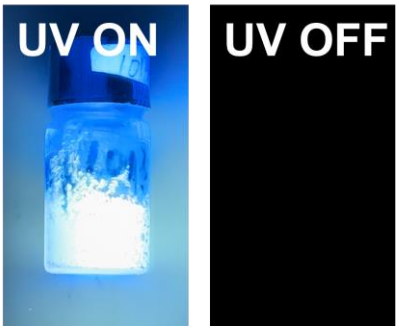

Supplementary Fig. 55. Photographs of the frozen **NAPpBP-PEG** solution at 10 mg/mL under UV and after switching off UV excitation.

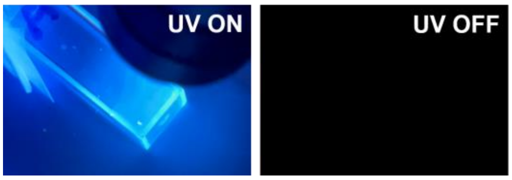

Supplementary Fig. 56. Photographs of the frozen solution of **NAPpBP-NHCOCH$_3$** and SA under UV and after switching off UV excitation.

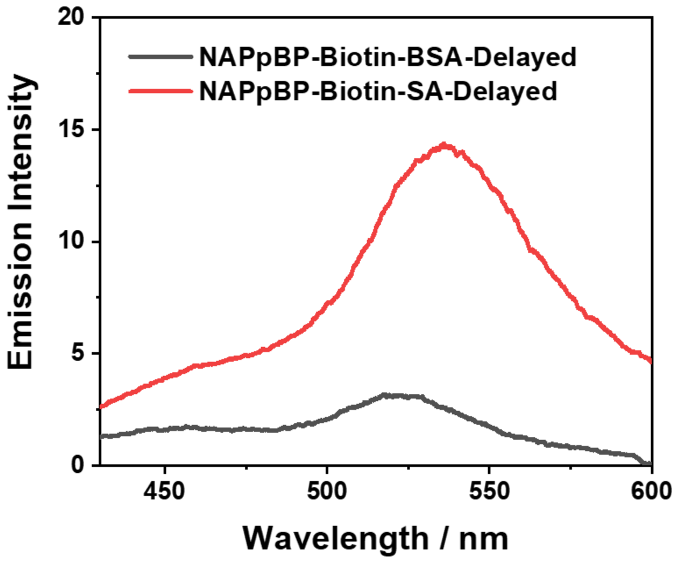

Supplementary Fig. 57. Delayed emission spectra of the frozen solution of **NAPpBP-Biotin** and **BSA** (without specific recognition) and the frozen solution of **NAPpBP-Biotin-SA** complex under 365 nm excitation.

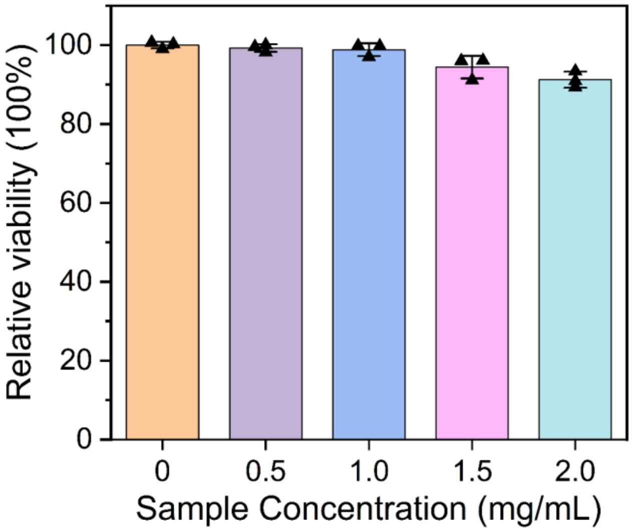

Supplementary Fig. 58. Cell viability assay of **NAPpBP-Biotin-SA** complex. No data were excluded from the analyses.

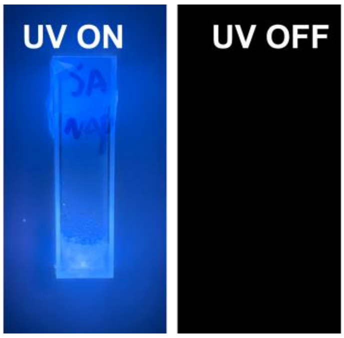

Supplementary Fig. 59. Photographs of the frozen solution of **1NAP-Biotin-SA** complex under UV and after switching off UV excitation.

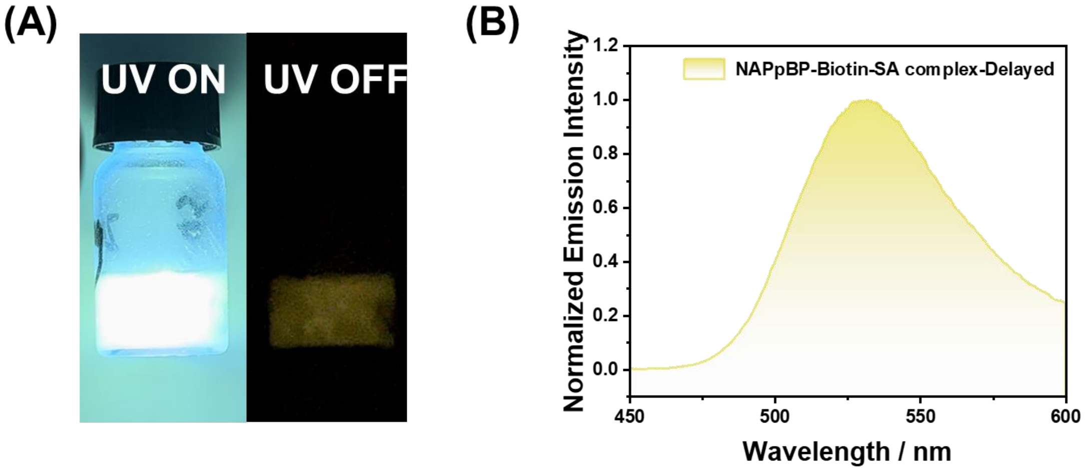

Supplementary Fig. 60. Photophysical property of **NAPpBP-Biotin-SA** complex. (A) Photograph of the 2 mg/mL frozen **NAPpBP-Biotin-SA** complex under UV and after switching off UV excitation. (B) Delayed emission spectra of the frozen **NAPpBP-Biotin-SA** complex. The complex can be obtained by directly adding powders of **NAPpBP-Biotin** into aqueous solution of streptavidin, followed by 8 h stirring. This protocol avoids the use of organic solvent and eliminate separation procedures.

Structural characterization results and sample purity information

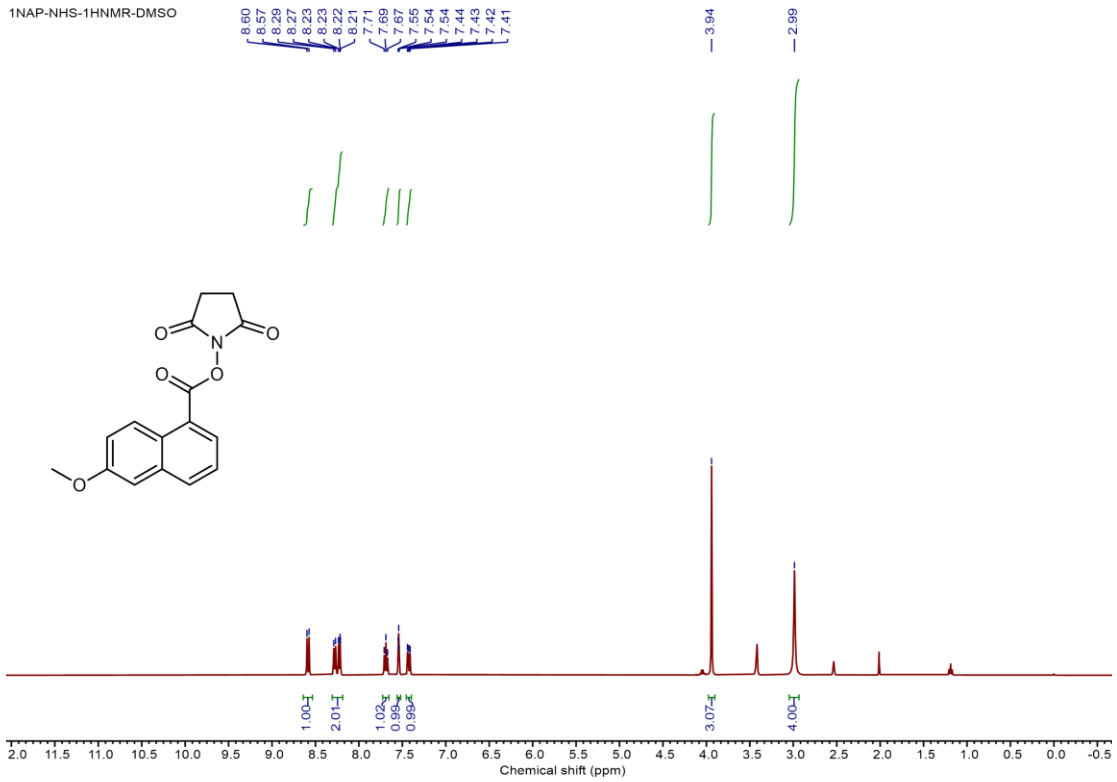

Supplementary Fig. 61. ^{1}H NMR spectra (400 MHz, DMSO-d_6) of compound **1NAP-NHS.**

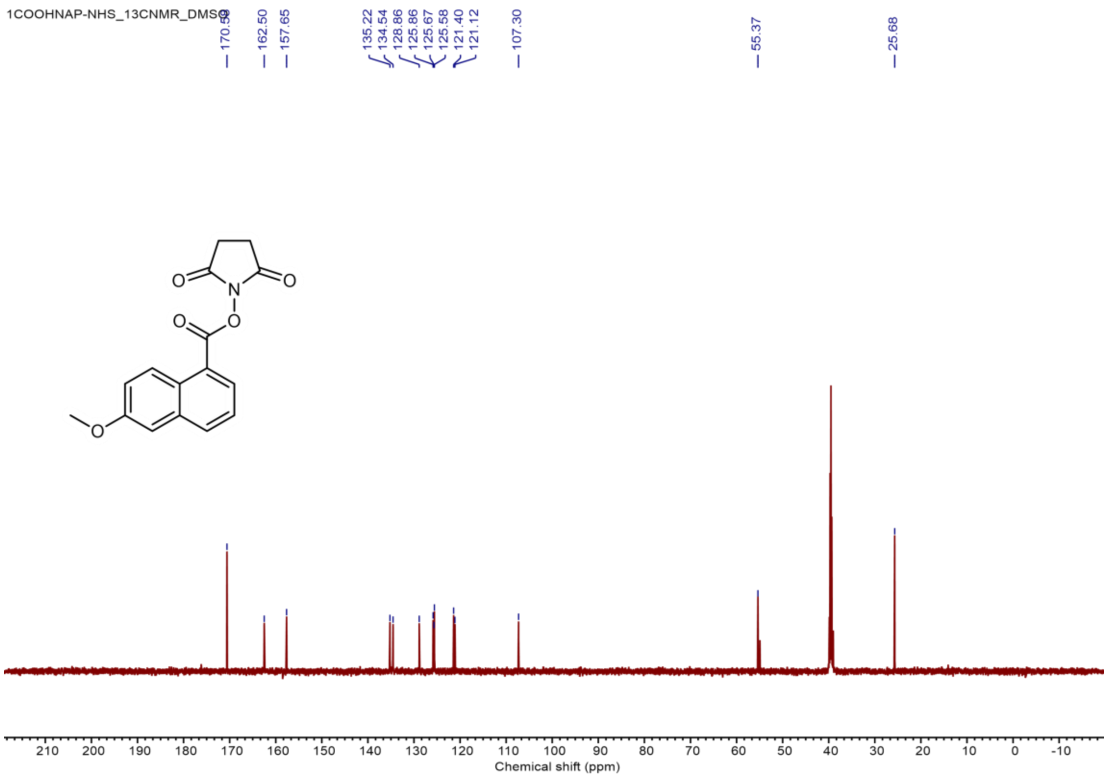

Supplementary Fig. 62. ^{13}C NMR spectra (126 MHz, DMSO-d_6) of compound **1NAP-NHS.**

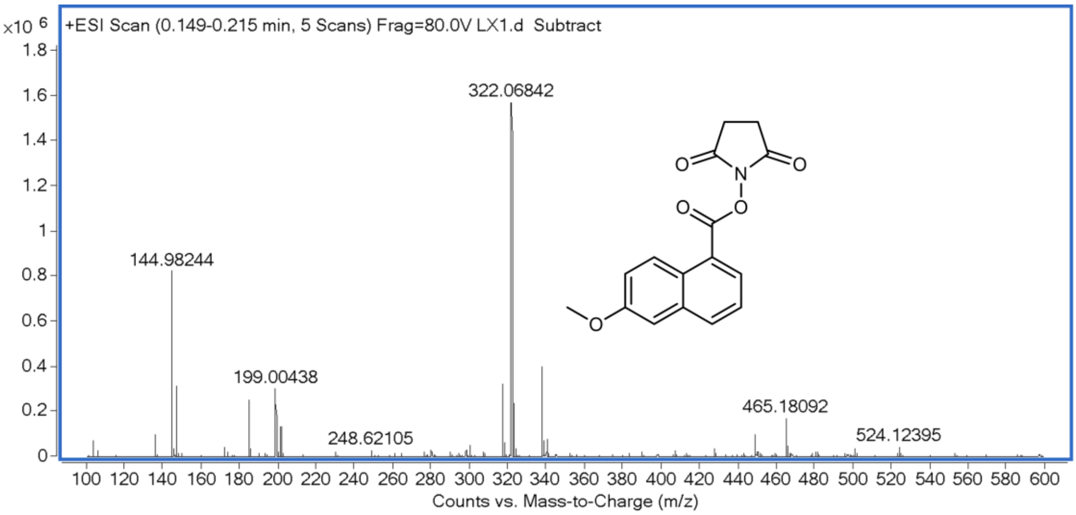

Supplementary Fig. 63. HRMS spectra of compound **1NAP-NHS.**

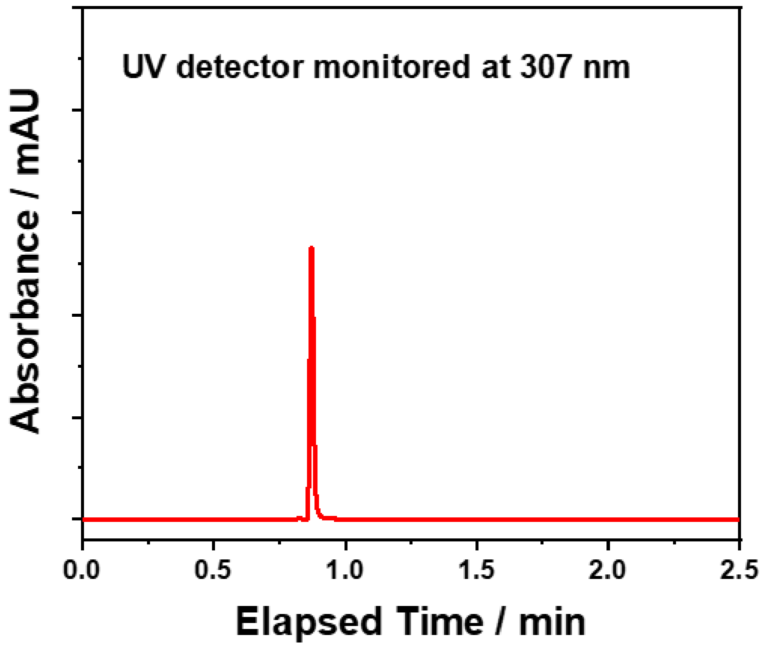

Supplementary Fig. 64. HPLC of compound **1NAP-NHS.**

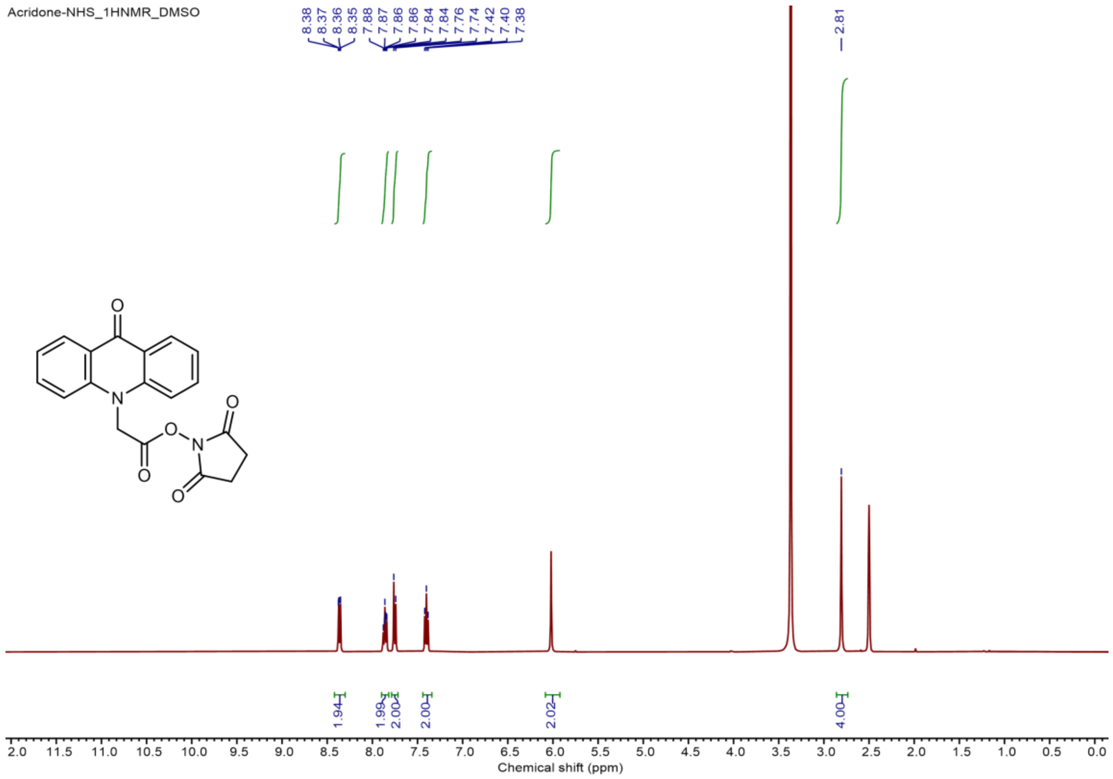

Supplementary Fig. 65. ^{1}H NMR spectra (400 MHz, DMSO-d_6) of compound **Acridone-NHS.**

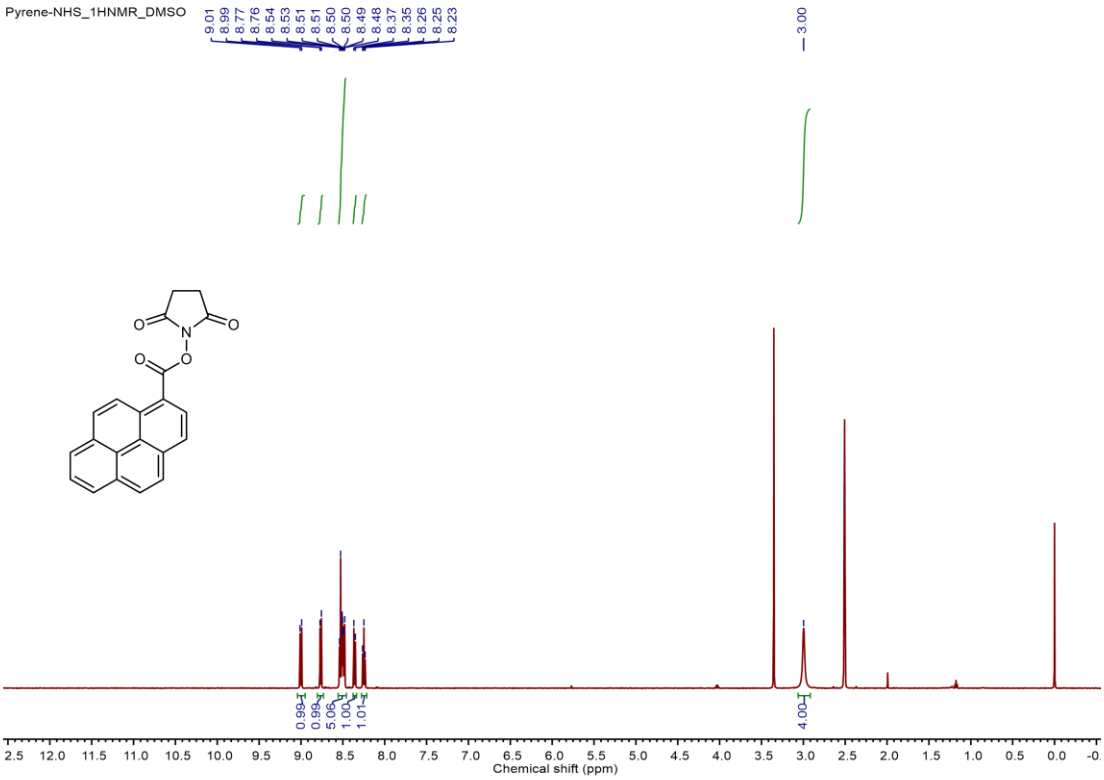

Supplementary Fig. 66. ^{1}H NMR spectra (500 MHz, DMSO-d_6) of compound **Pyrene-NHS.**

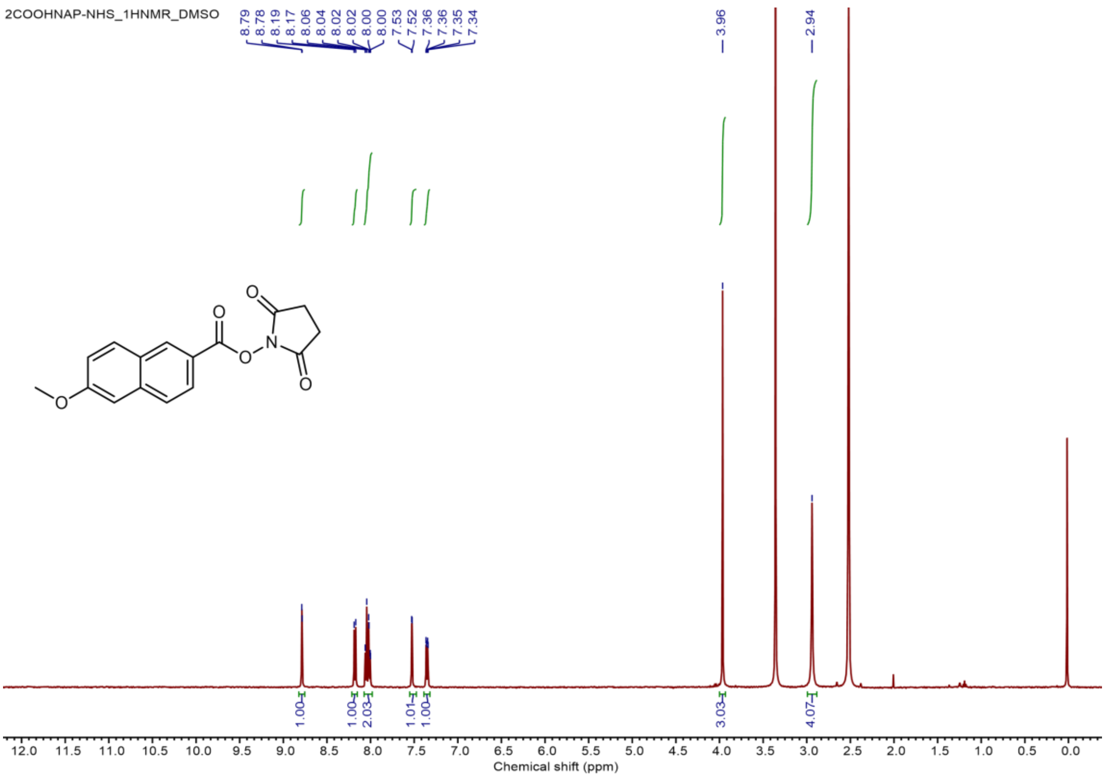

Supplementary Fig. 67. ^{1}H NMR spectra (500 MHz, DMSO-d_6) of compound **2NAP-NHS.**

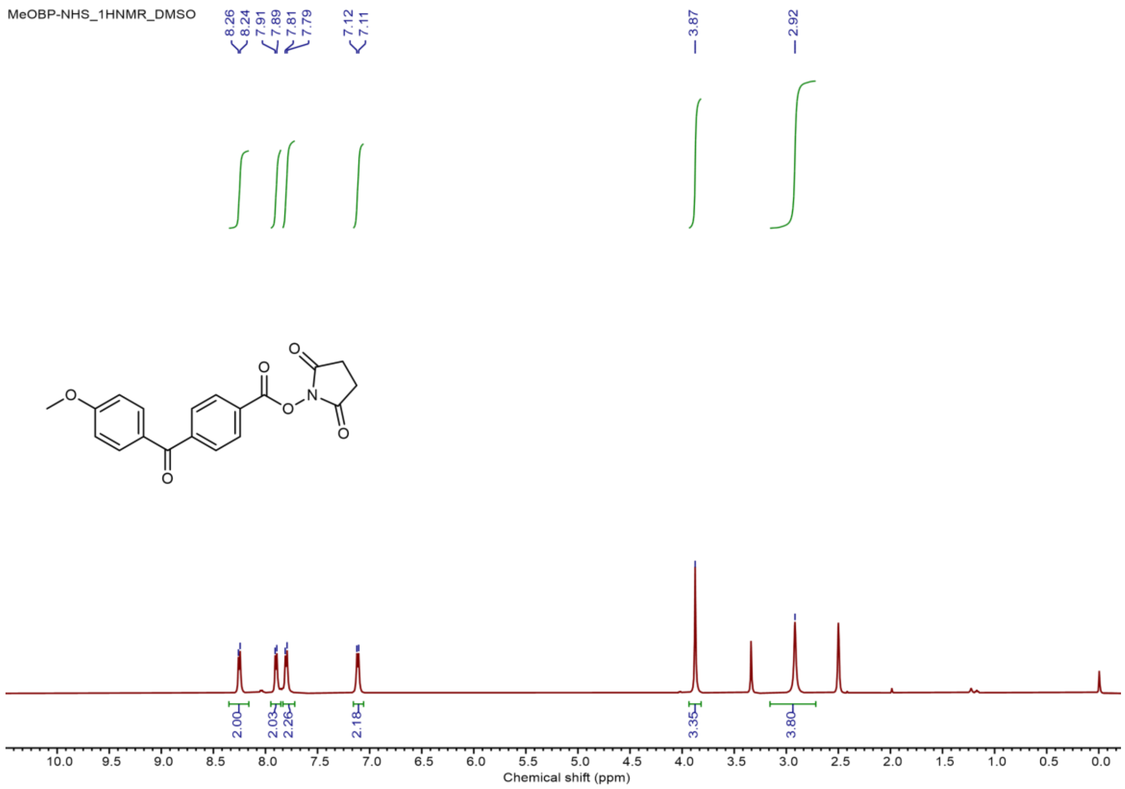

Supplementary Fig. 68. ^{1}H NMR spectra (500 MHz, DMSO-d_6) of compound **MeOBP-NHS.**

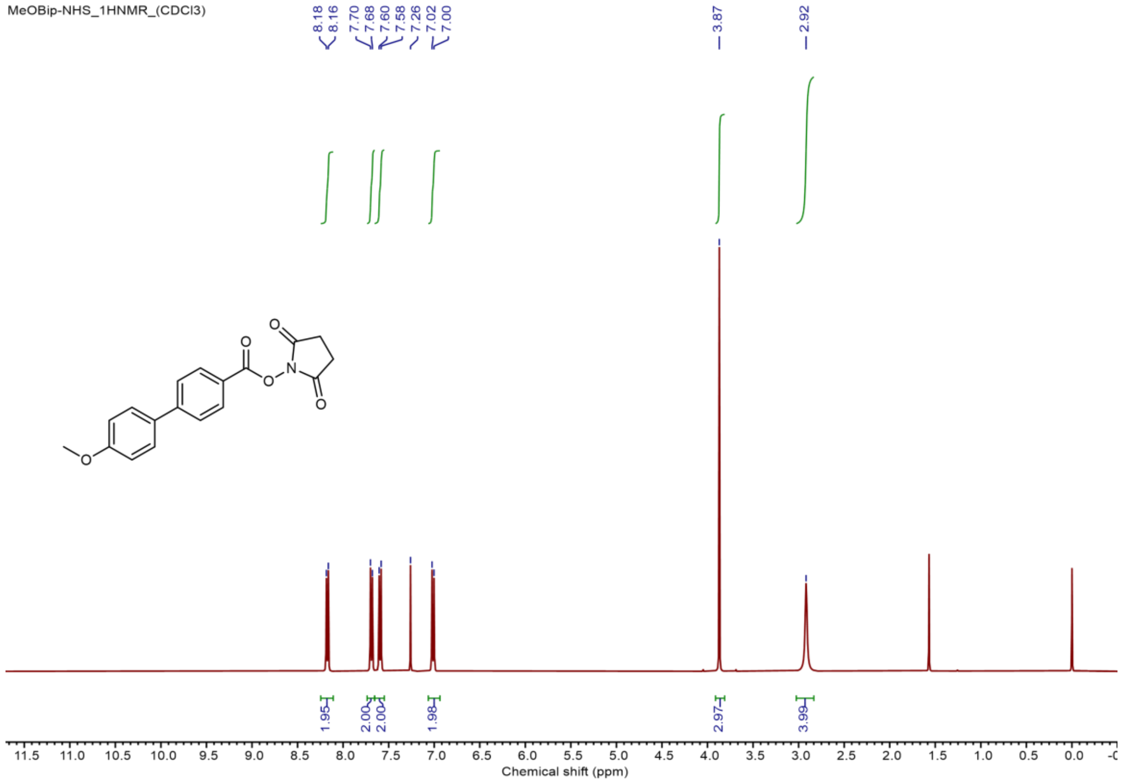

Supplementary Fig. 69. ^{1}H NMR spectra (400 MHz, Chloroform-d) of compound **MeOBip-NHS.**

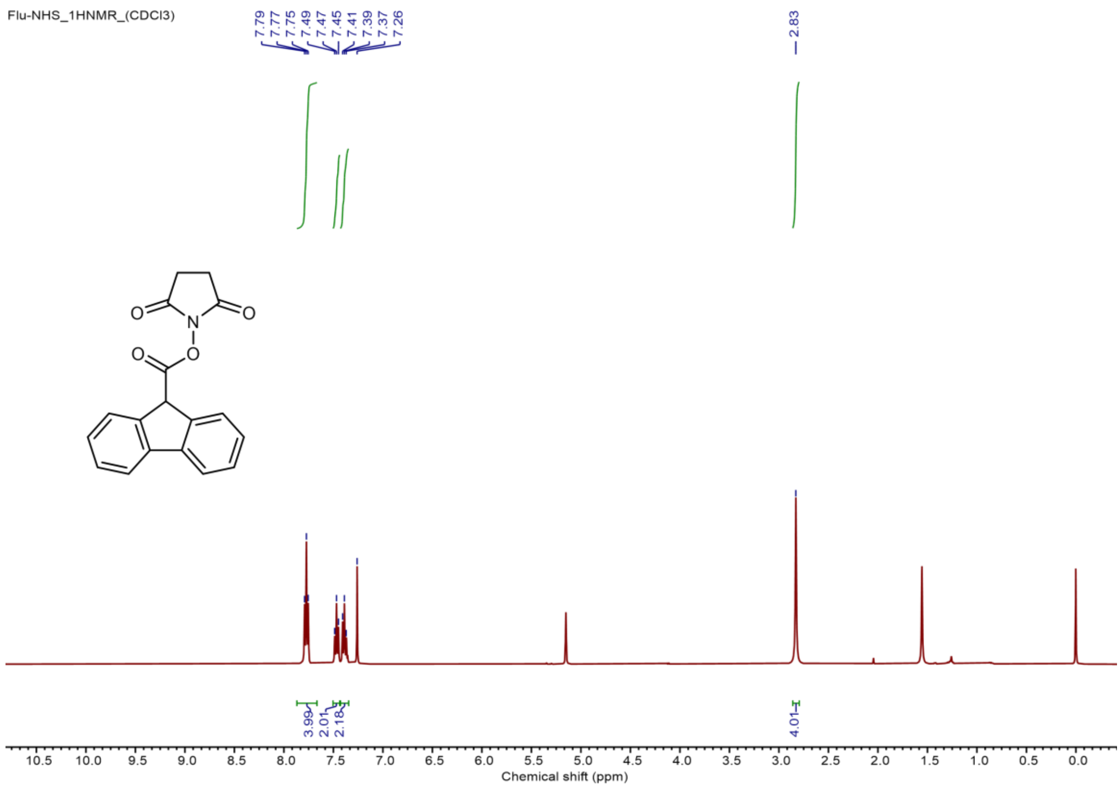

Supplementary Fig. 70. ^{1}H NMR spectra (400 MHz, Chloroform-*d*) of compound **Fluorene-NHS.**

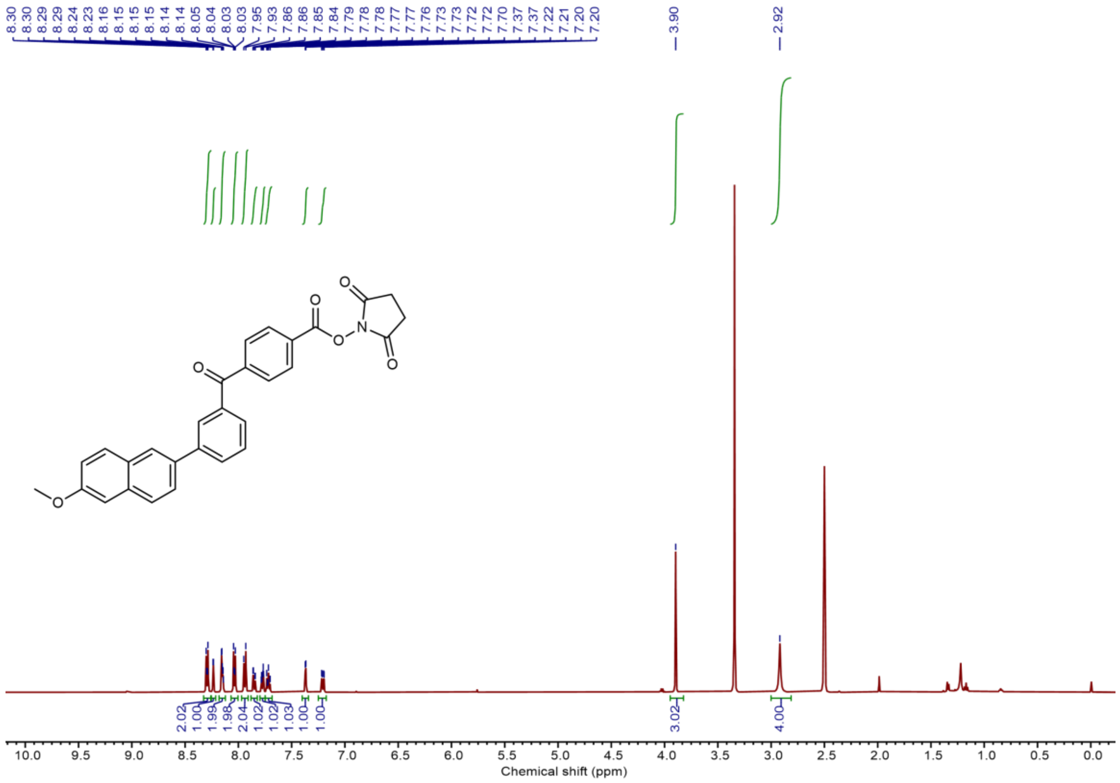

Supplementary Fig. 71. ^{1}H NMR spectra (500 MHz, DMSO-d_6) of compound **NAPBP-NHS.**

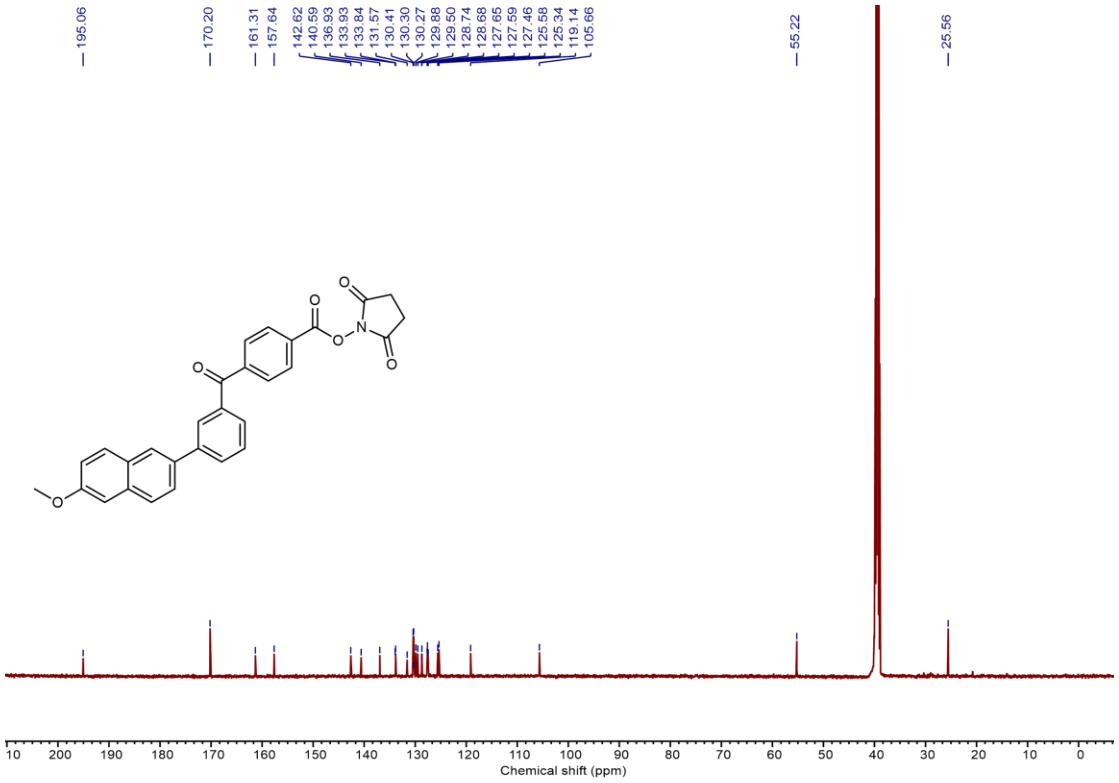

Supplementary Fig. 72. ^{13}C NMR spectra (126 MHz, DMSO-d_6) of compound **NAPBP-NHS.**

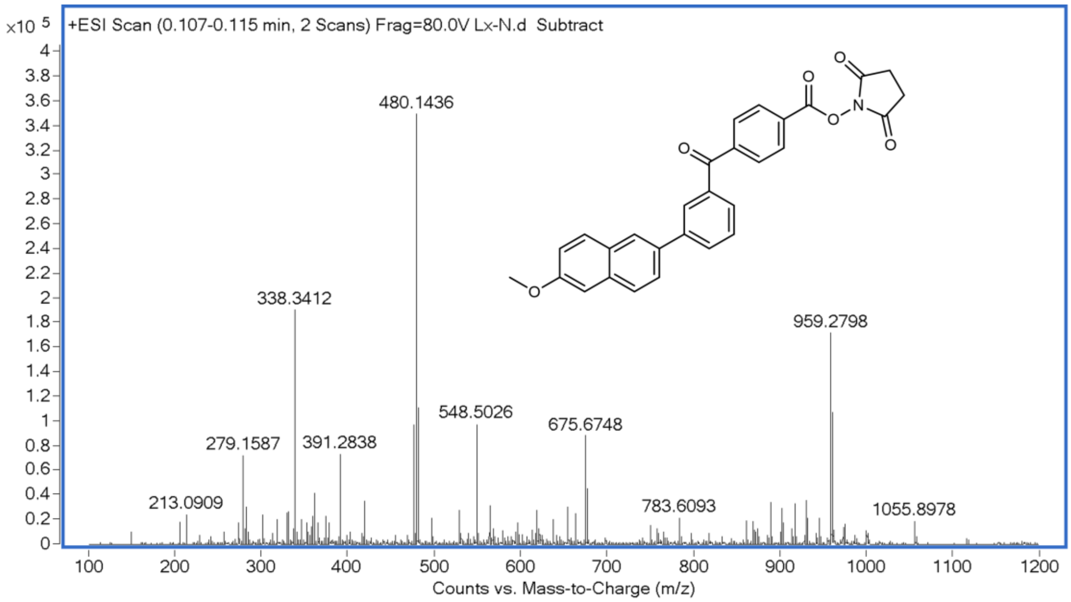

Supplementary Fig. 73. HRMS spectra of compound **NAPBP-NHS.**

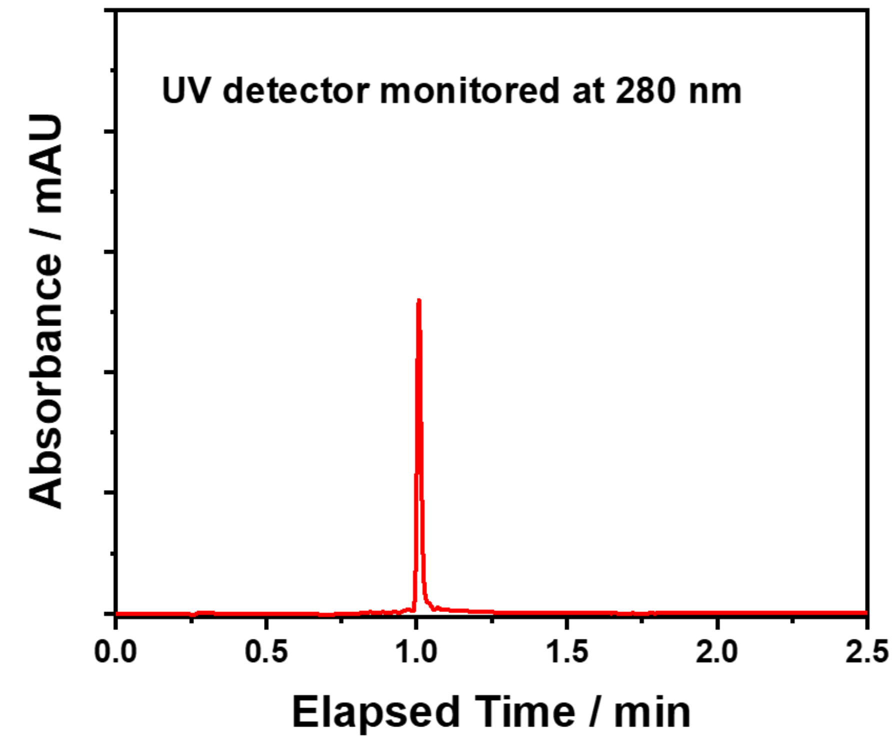

Supplementary Fig. 74. HPLC of compound **NAPBP-NHS.**

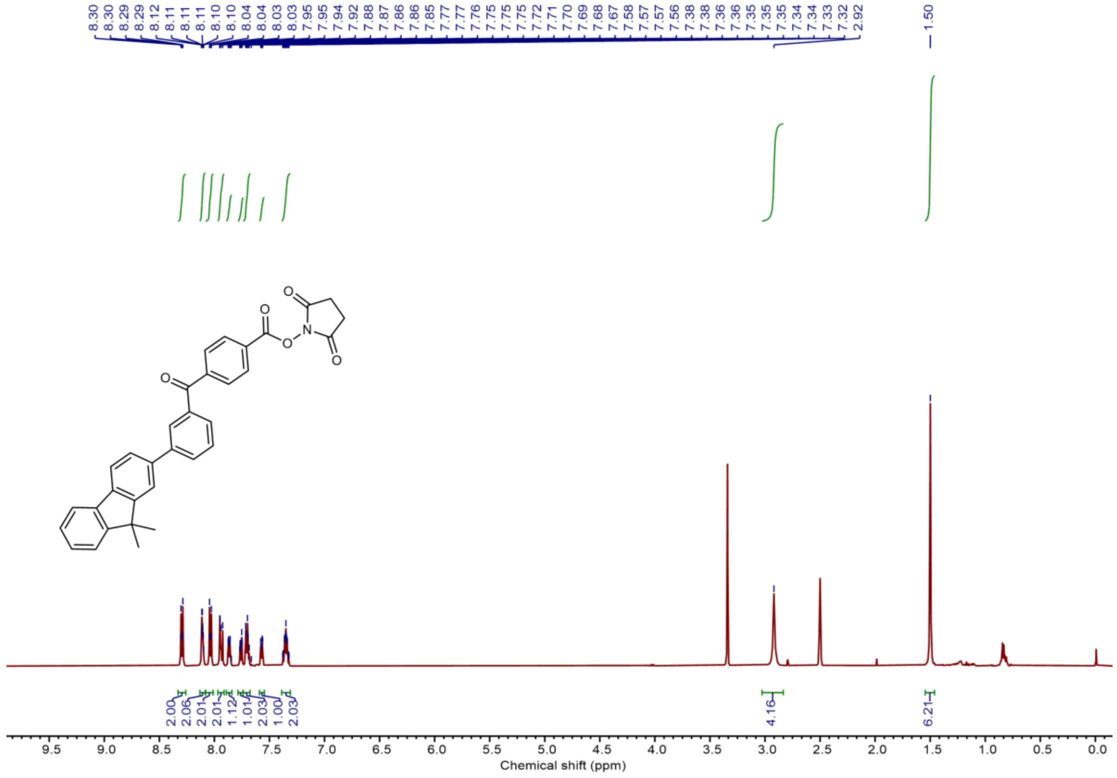

Supplementary Fig. 75. ^{1}H NMR spectra (500 MHz, DMSO-d_6) of compound **FluoreneBP-NHS.**

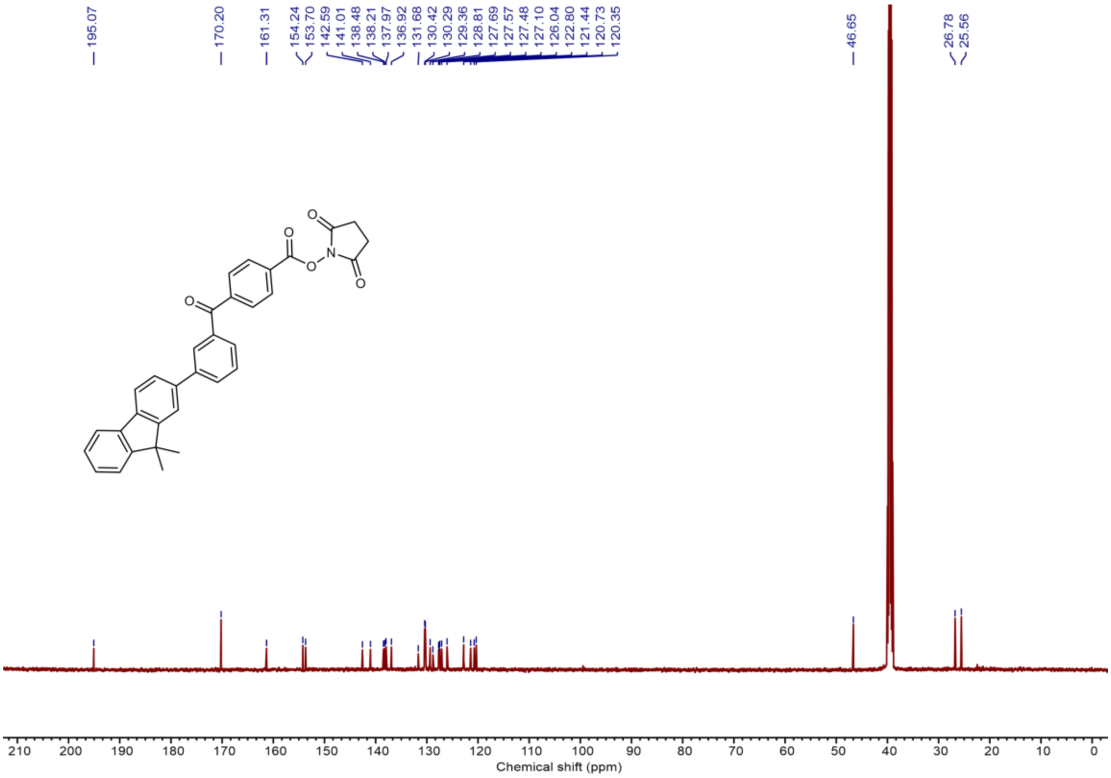

Supplementary Fig. 76. ^{13}C NMR spectra (126 MHz, DMSO-d_6) of compound **FluoreneBP-NHS.**

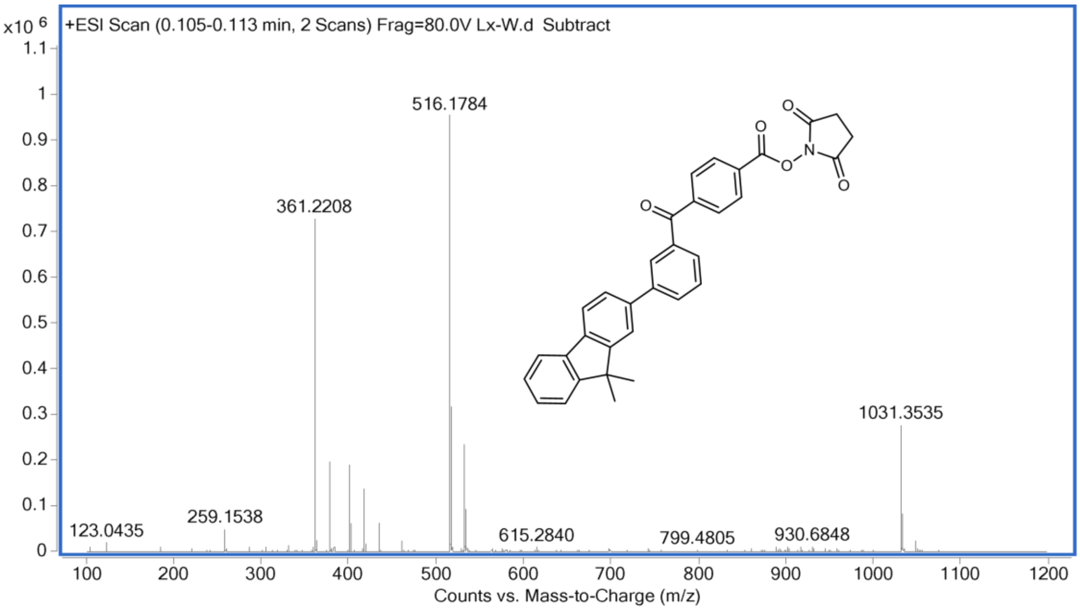

Supplementary Fig. 77. HRMS spectra of compound **FluoreneBP-NHS.**

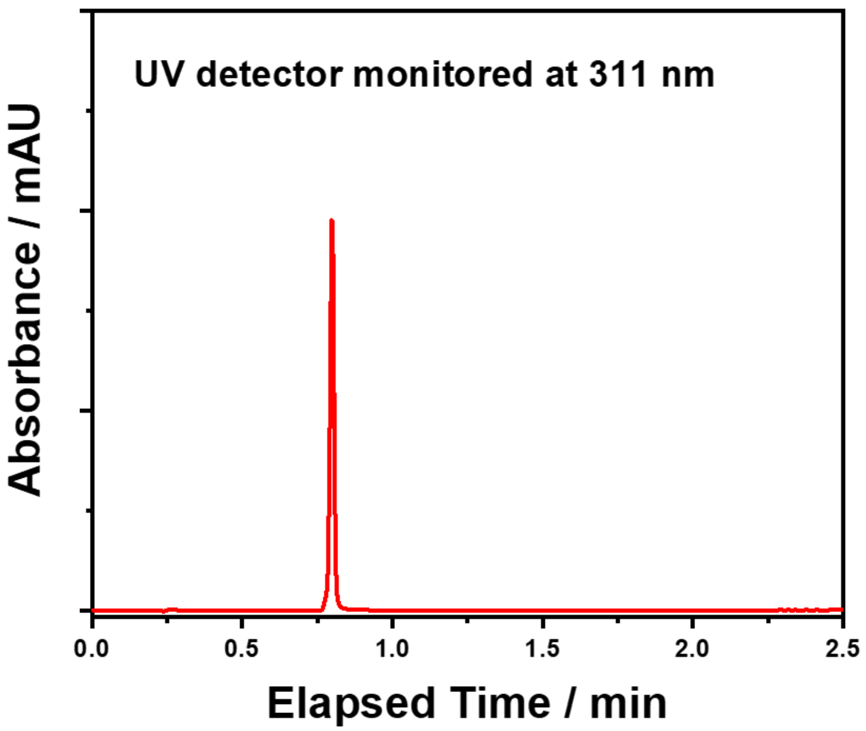

Supplementary Fig. 78. HPLC of compound **FluoreneBP-NHS.**

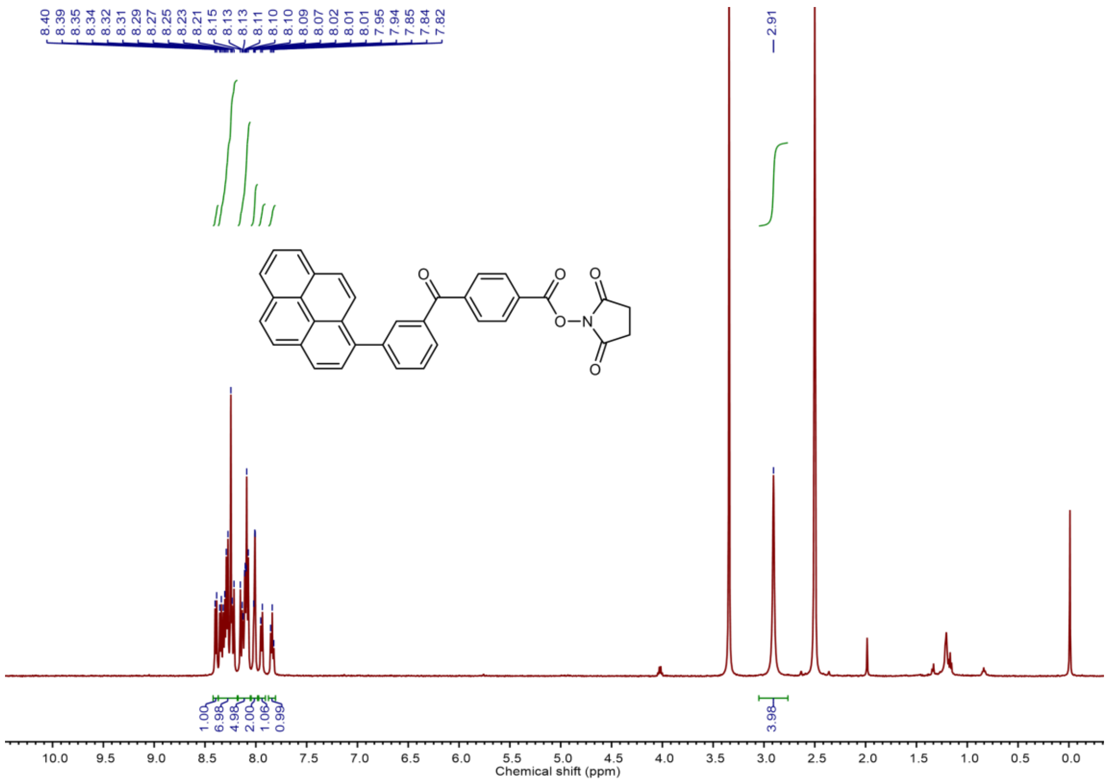

Supplementary Fig. 79. ^{1}H NMR spectra (500 MHz, DMSO-d_6) of compound **PyreneBP-NHS.**

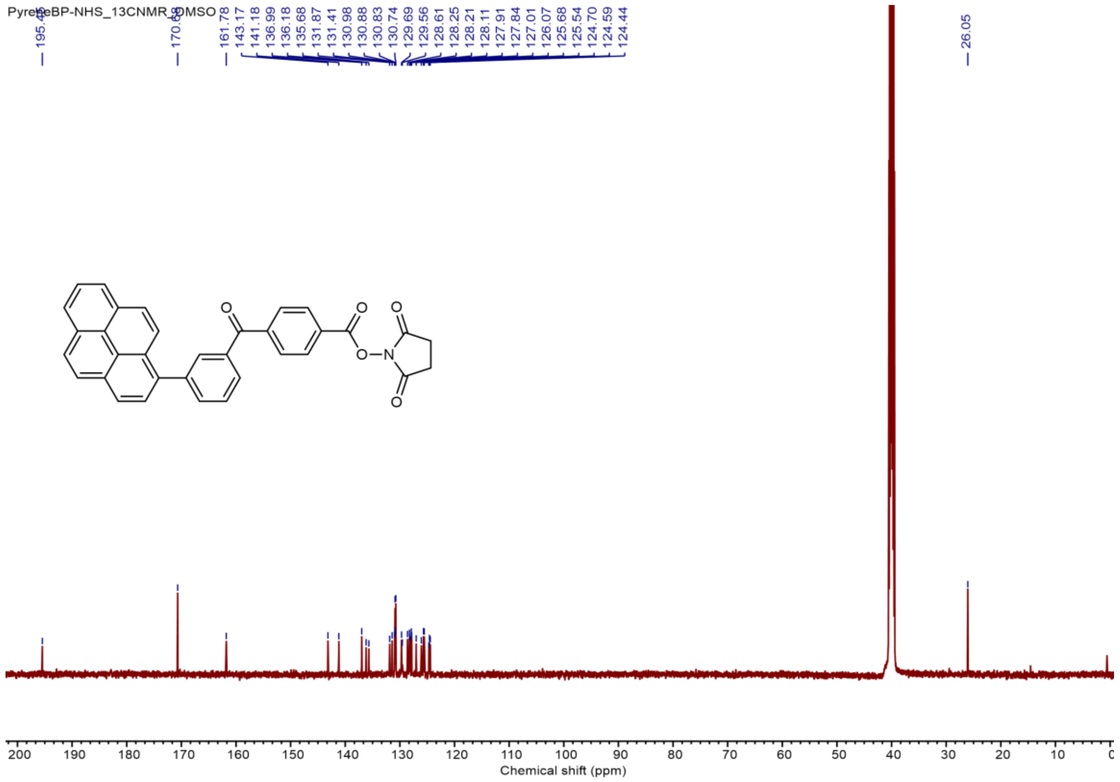

Supplementary Fig. 80. ^{13}C NMR spectra (126 MHz, DMSO-d_6) of compound **PyreneBP-NHS.**

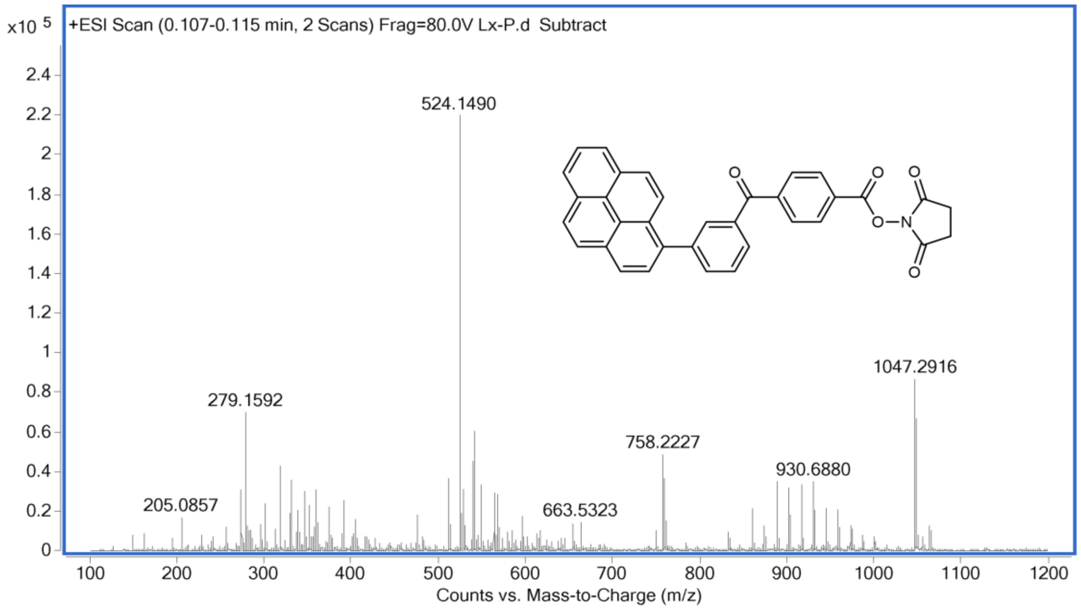

Supplementary Fig. 81. HRMS spectra of compound **PyreneBP-NHS.**

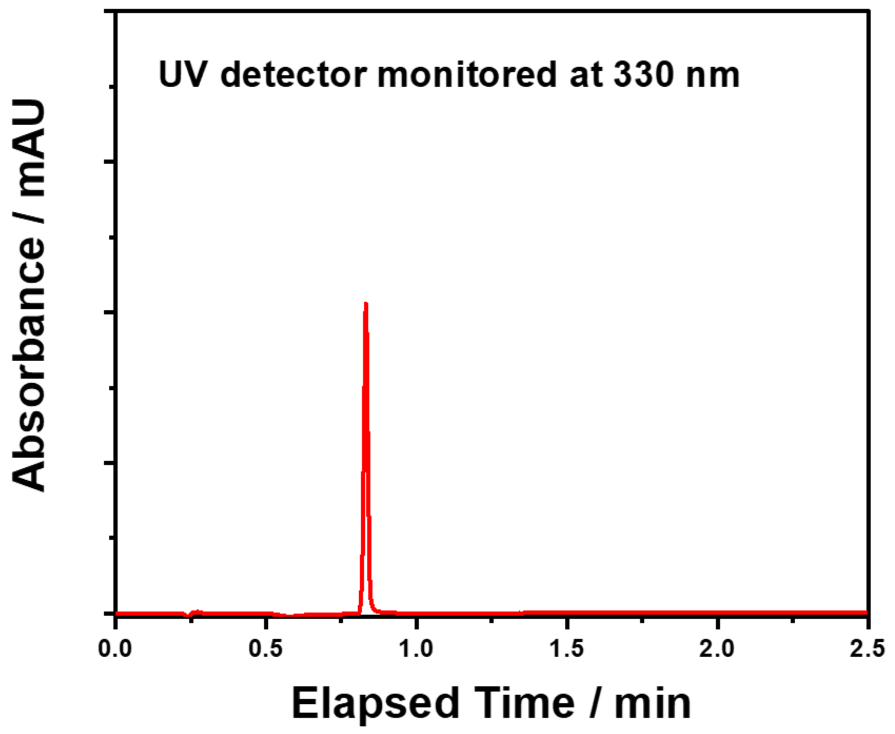

Supplementary Fig. 82. HPLC of compound **PyreneBP-NHS.**

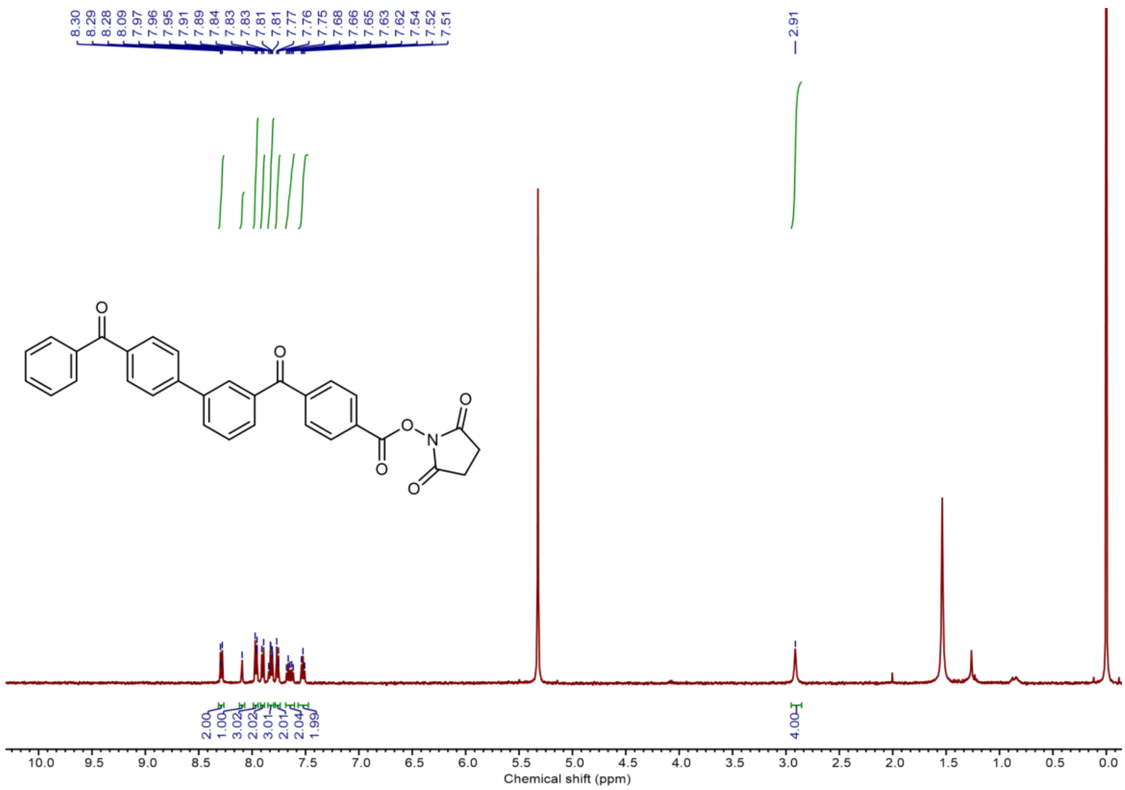

Supplementary Fig. 83. ^{1}H NMR spectra (500 MHz, Methylene Chloride-d_2) of compound **BPBP-NHS.**

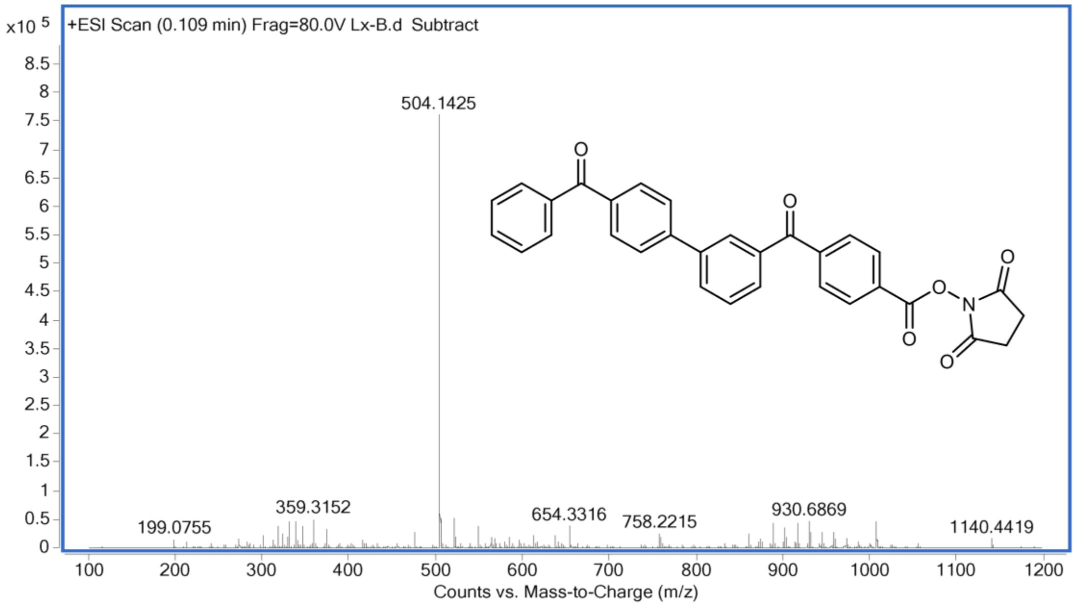

Supplementary Fig. 84. HRMS spectra of compound **BPBP-NHS.**

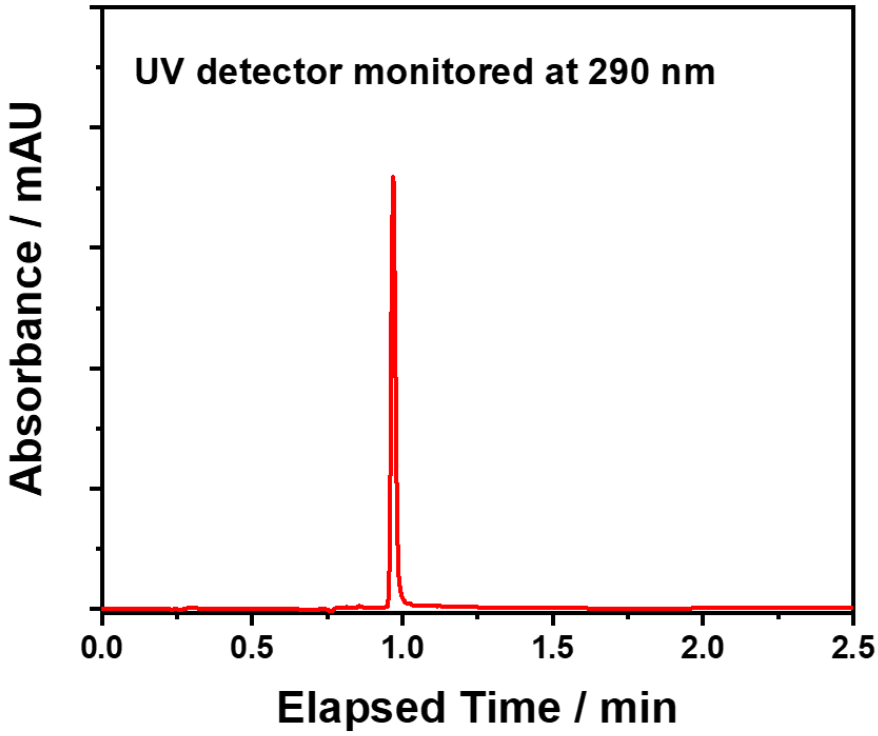

Supplementary Fig. 85. HPLC of compound **BPBP-NHS.**

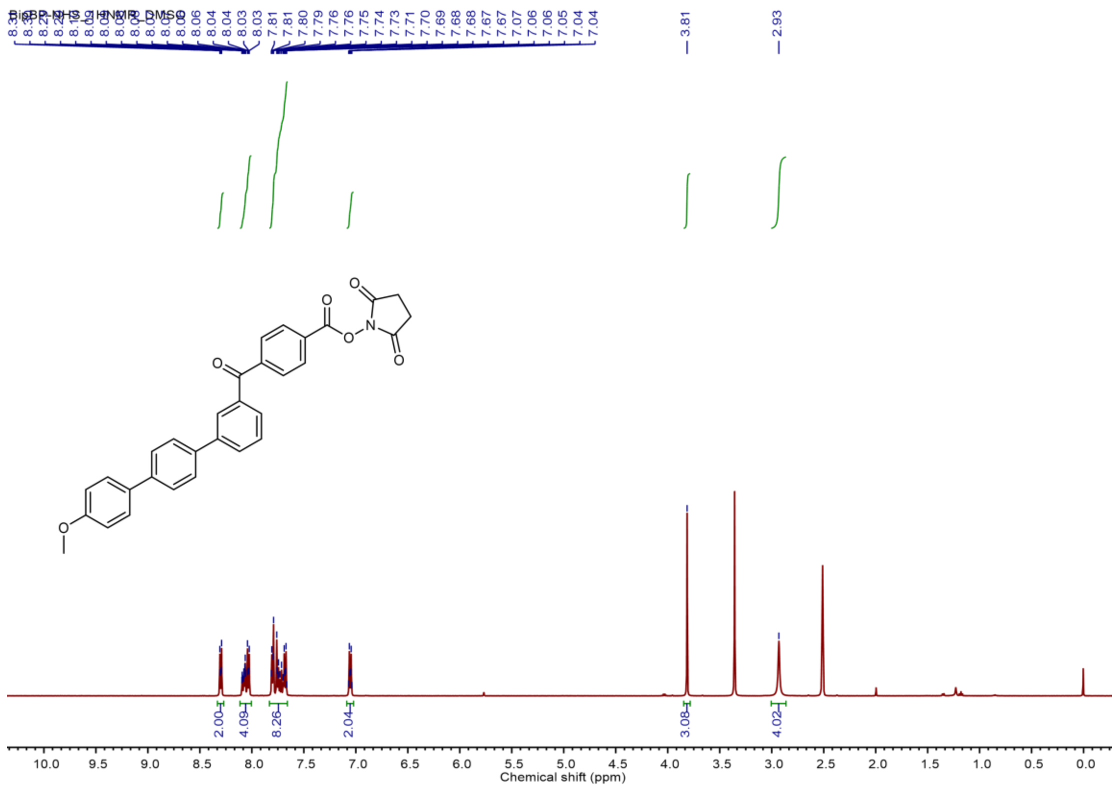

Supplementary Fig. 86. ^{1}H NMR spectra (500 MHz, DMSO-d_6) of compound **MeOBipBP-NHS.**

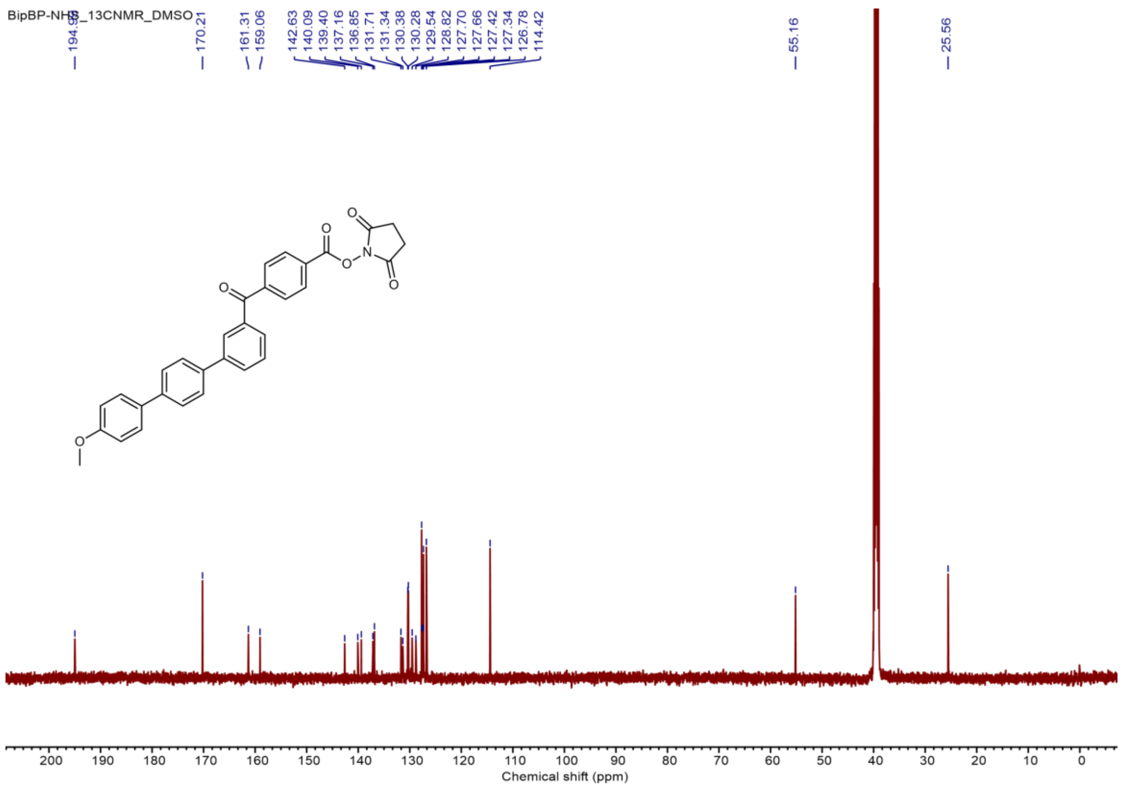

Supplementary Fig. 87. ^{13}C NMR spectra (126 MHz, DMSO-d_6) of compound **MeOBipBP-NHS.**

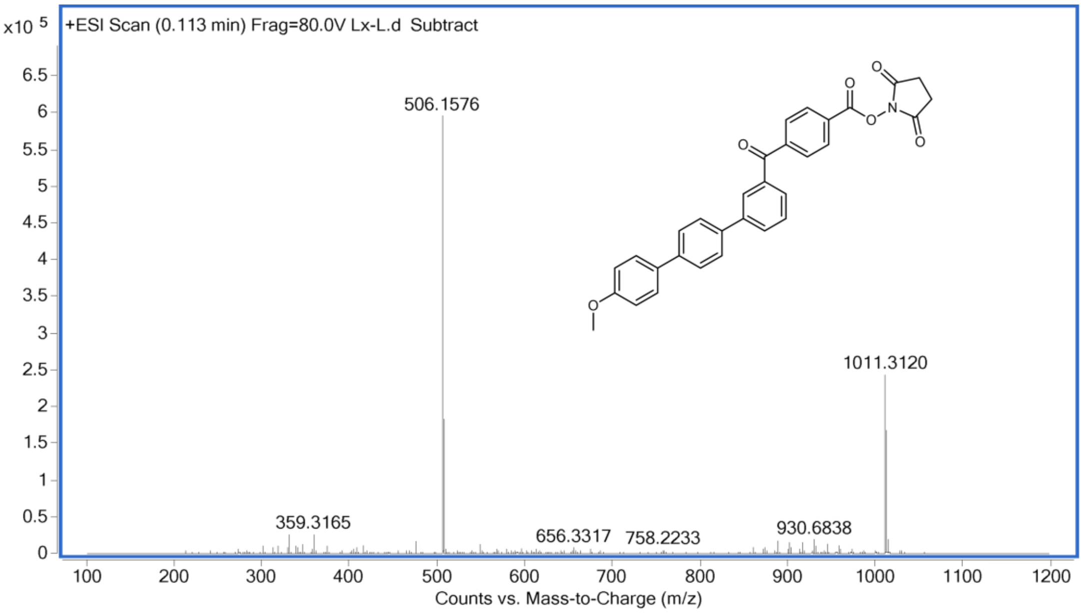

Supplementary Fig. 88. HRMS spectra of compound **MeOBipBP-NHS.**

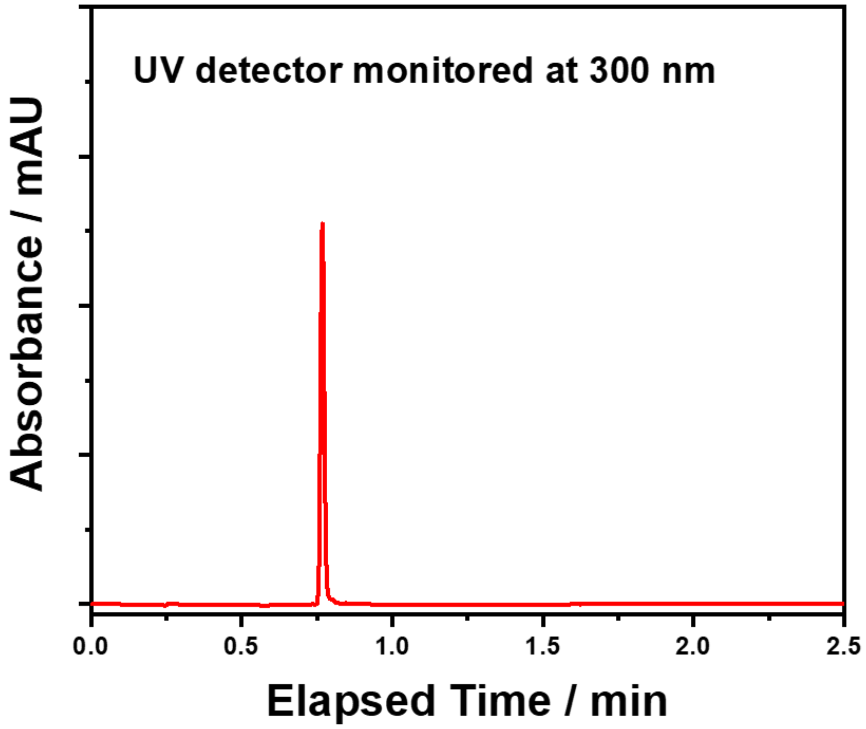

Supplementary Fig. 89. HPLC compound **MeOBipBP-NHS.**

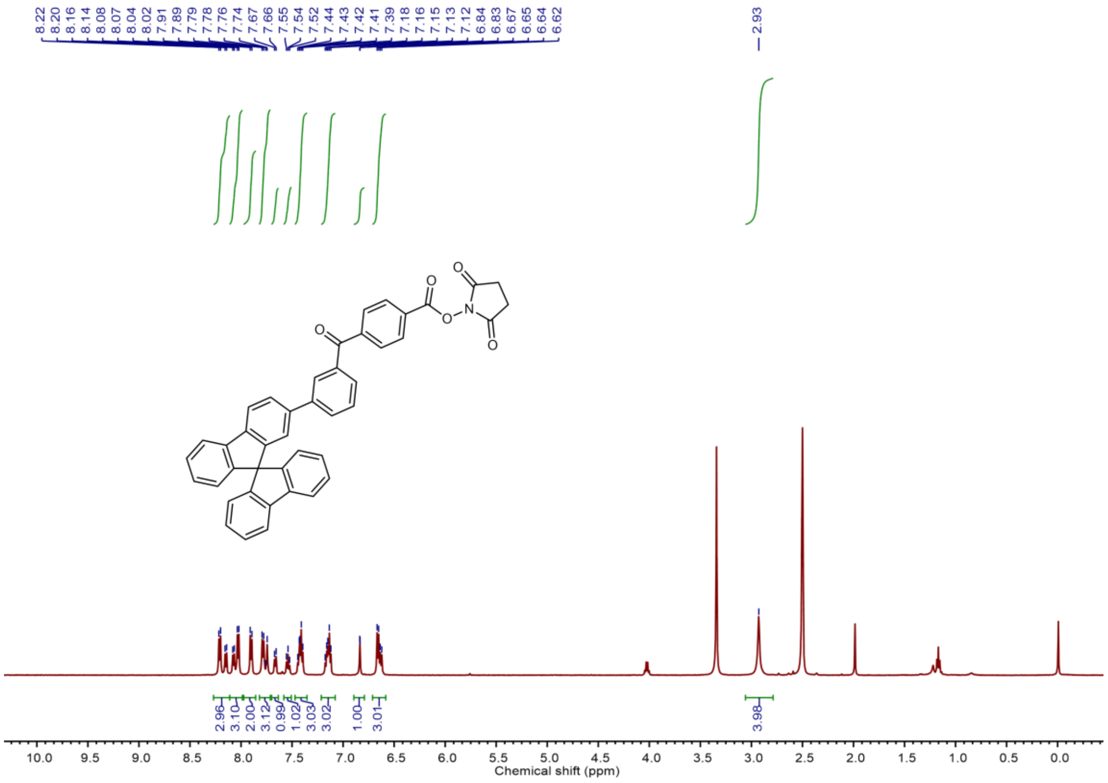

Supplementary Fig. 90. ^{1}H NMR spectra (500 MHz, DMSO-d_6) of compound **SpiroBP-NHS.**

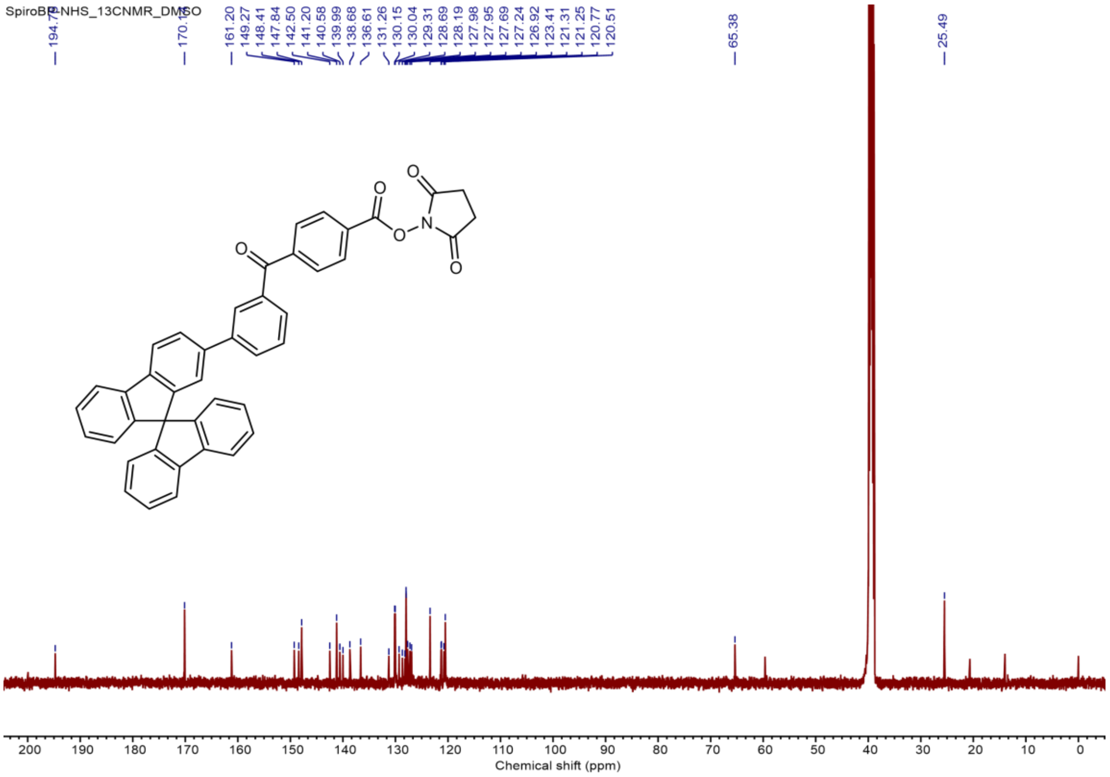

Supplementary Fig. 91. ^{13}C NMR spectra (126 MHz, DMSO-d_6) of compound **SpiroBP-NHS.**

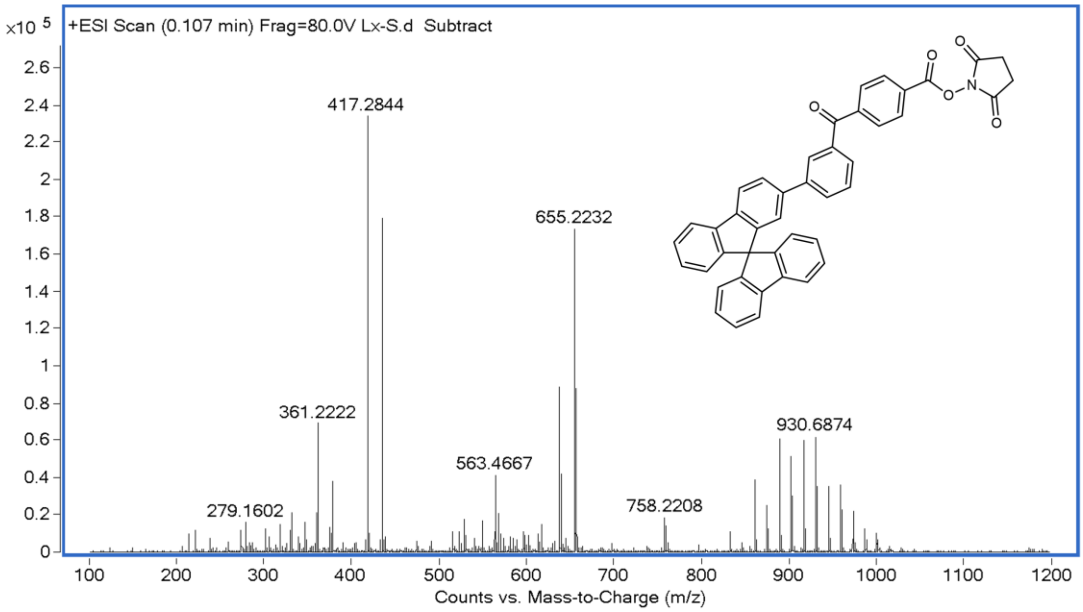

Supplementary Fig. 92. HRMS spectra of compound **SpiroBP-NHS.**

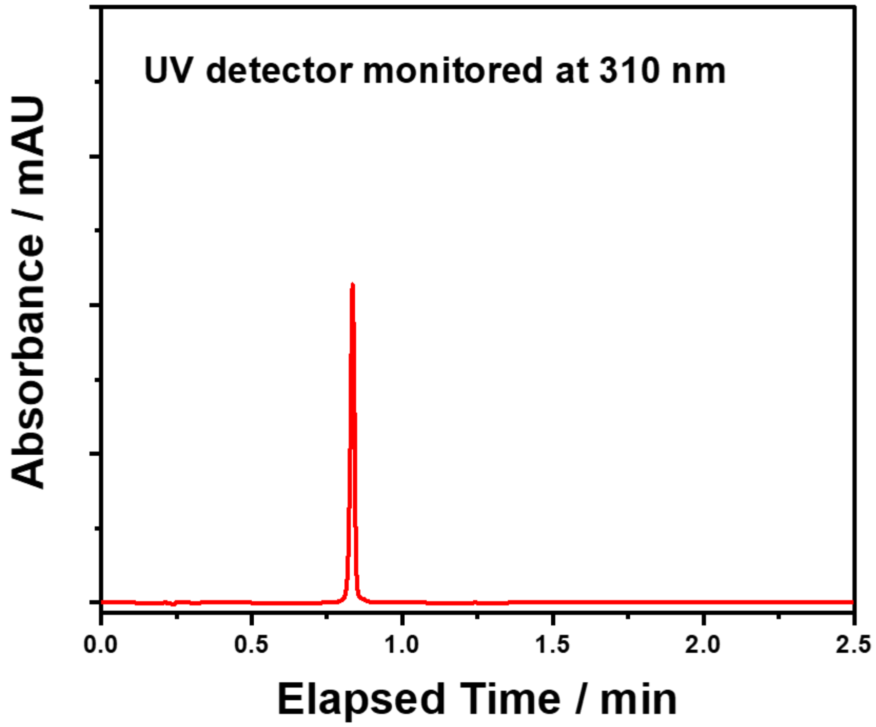

Supplementary Fig. 93. HPLC of compound **SpiroBP-NHS.**

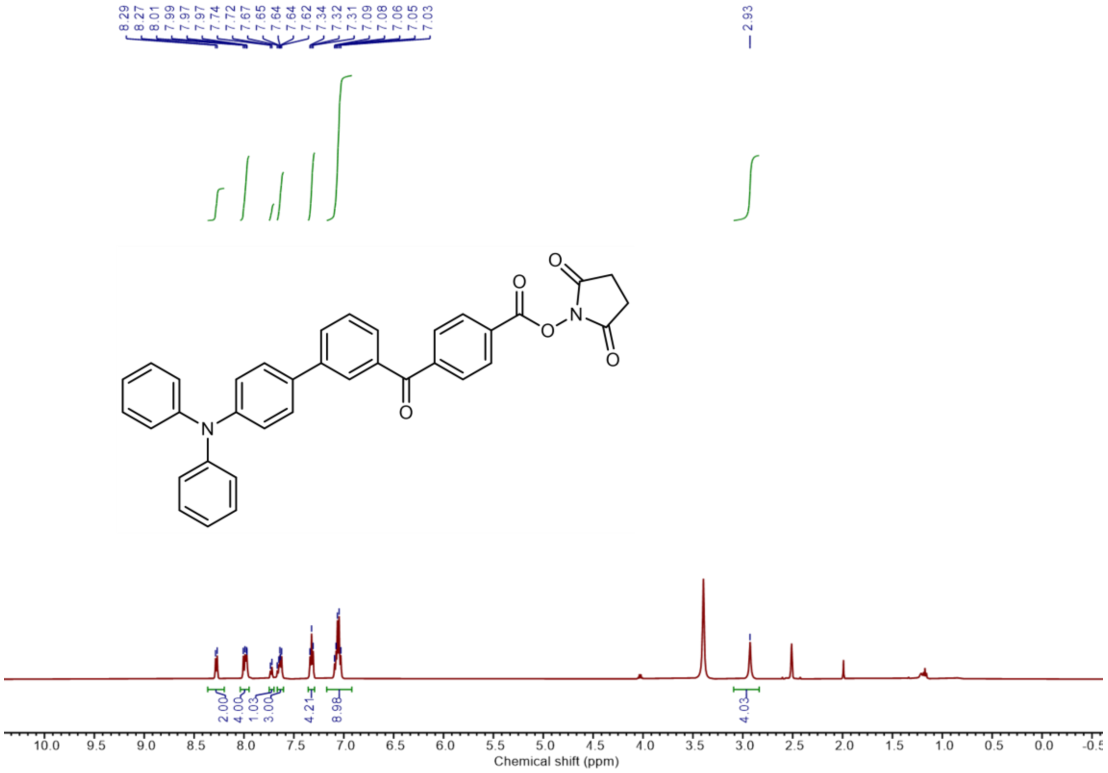

Supplementary Fig. 94. ^{1}H NMR spectra (500 MHz, DMSO-d_6) of compound **TPABP-NHS.**

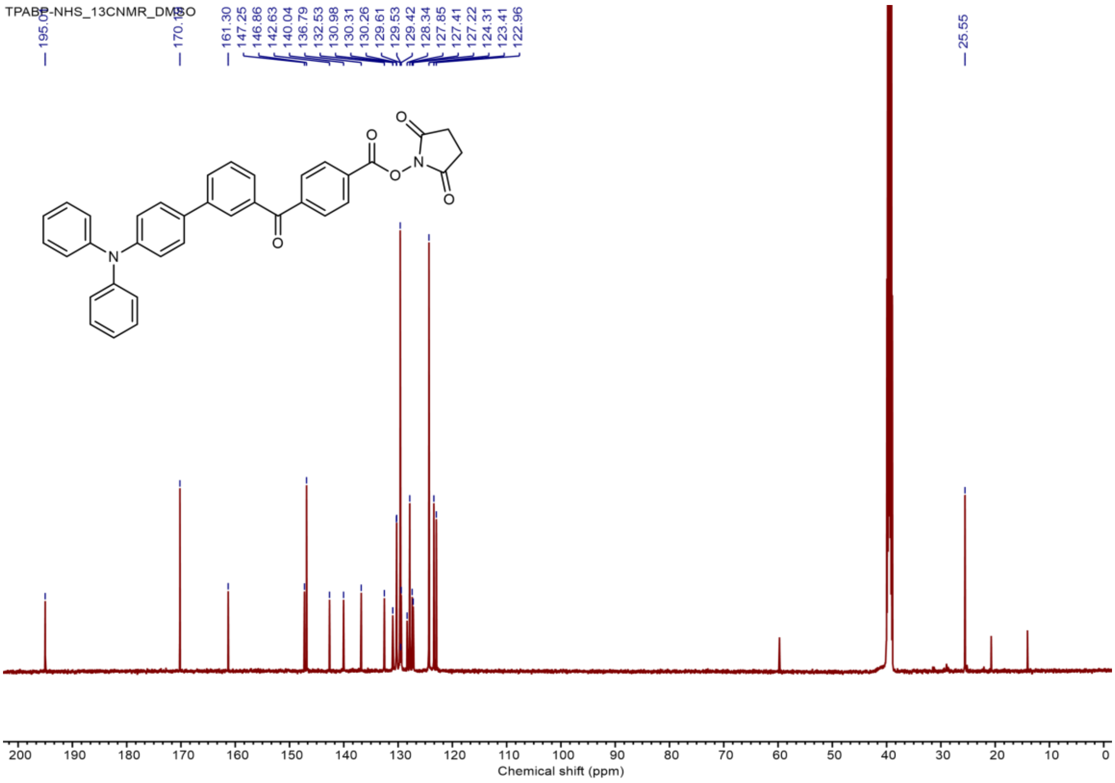

Supplementary Fig. 95. ^{13}C NMR spectra (126 MHz, DMSO-d_6) of compound **TPABP-NHS.**

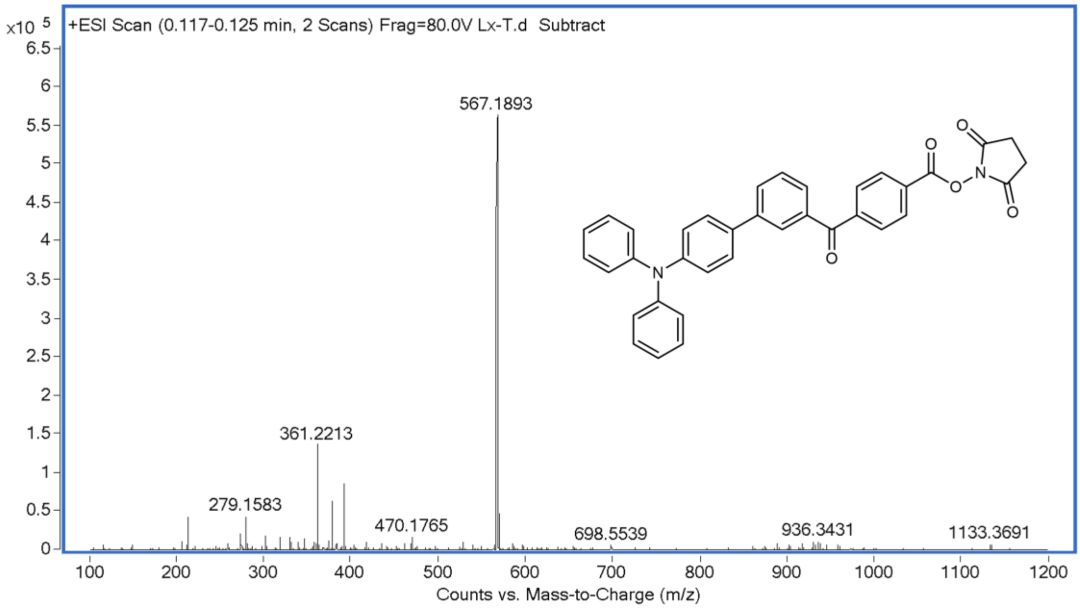

Supplementary Fig. 96. HRMS spectra of compound **TPABP-NHS.**

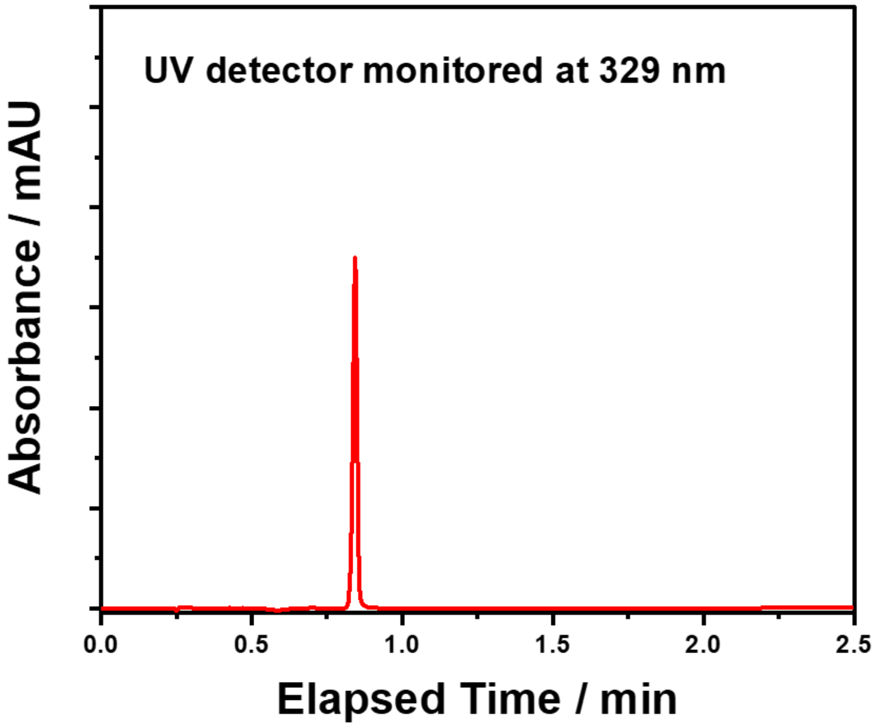

Supplementary Fig. 97. HPLC of compound **TPABP-NHS.**

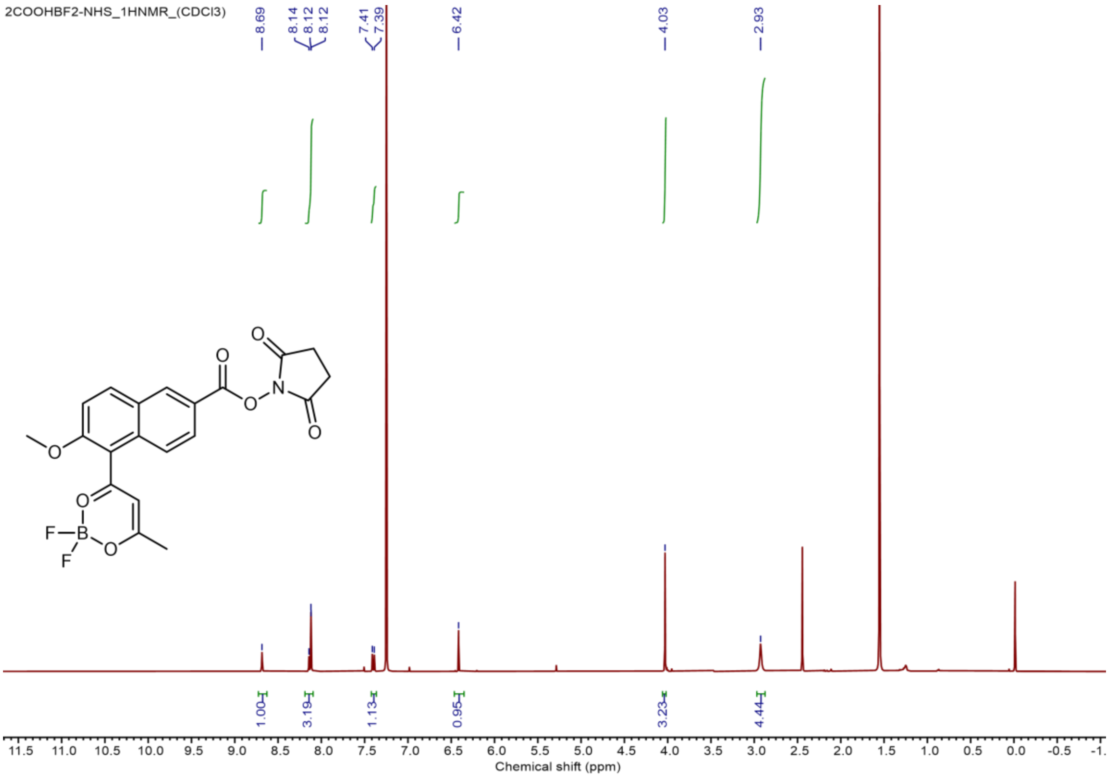

Supplementary Fig. 98. ^{1}H NMR spectra (400 MHz, Chloroform-*d*) of compound **2COOHNAPBF₂-NHS.**

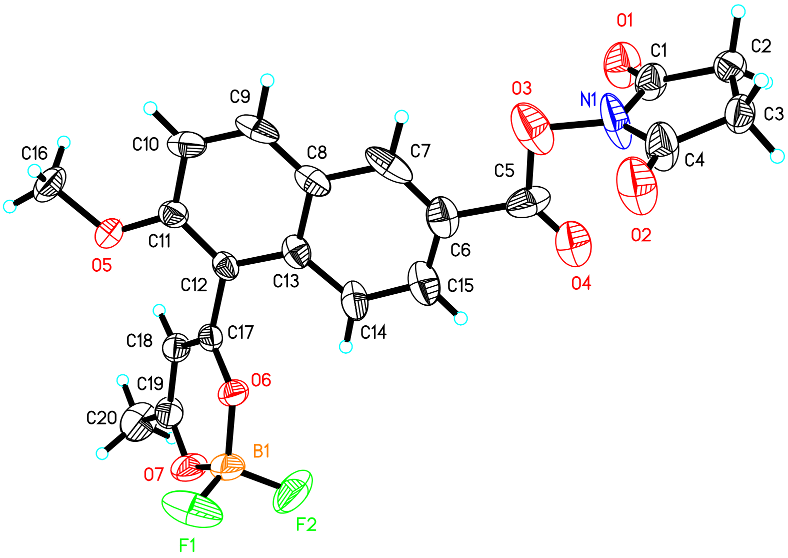

Supplementary Fig. 99. Single crystal structure of compound **2COOHNAPBF$_2$-NHS** (CCDC 2505526).

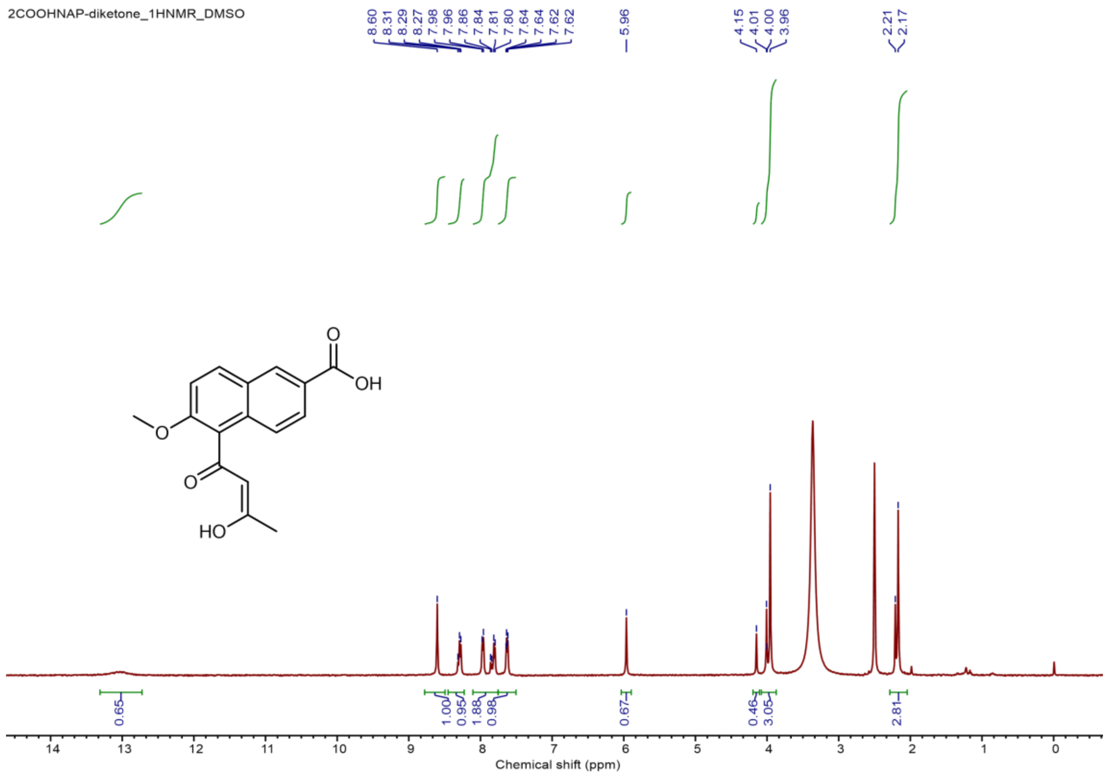

Supplementary Fig. 100. ^{1}H NMR spectra (500 MHz, DMSO-d_6) of compound **2COOHNAP-diketone**.

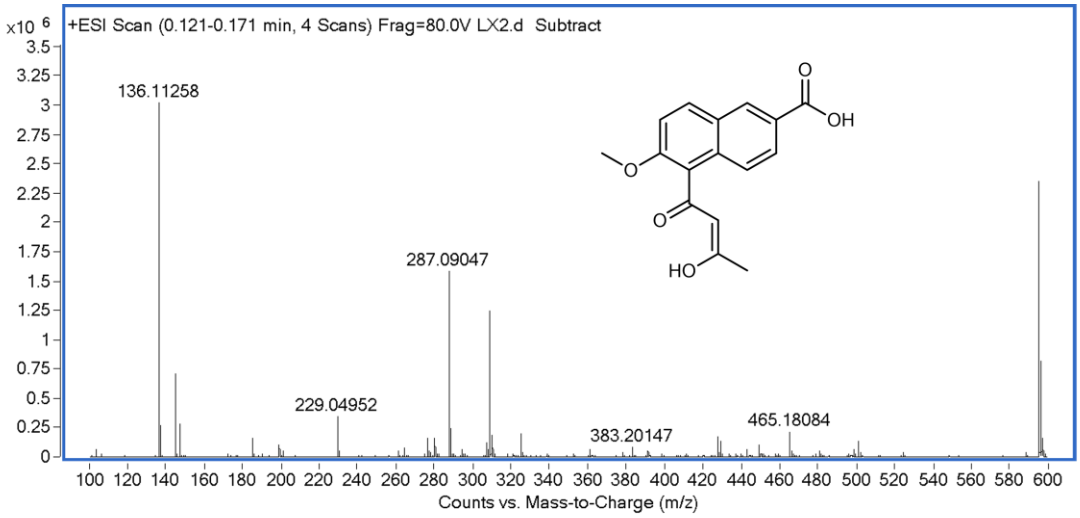

Supplementary Fig. 101. HRMS spectra of compound **2COOHNAP-diketone.**

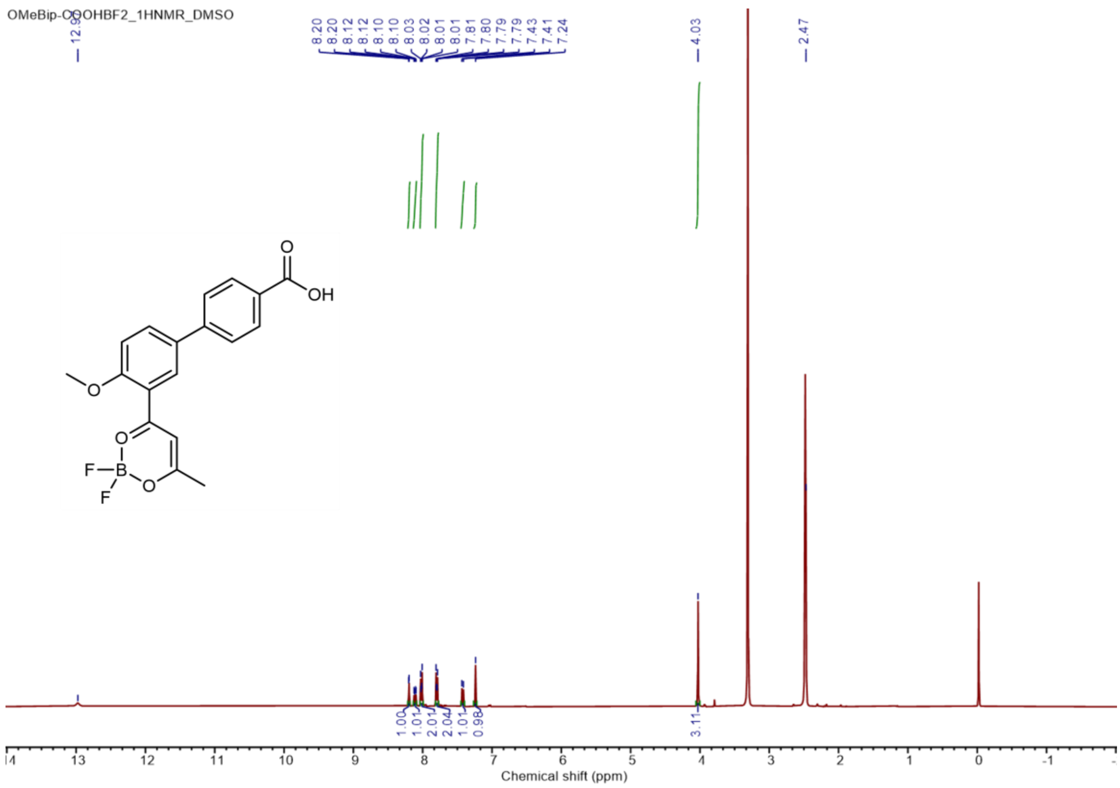

Supplementary Fig. 102. ^{1}H NMR spectra (400 MHz, DMSO-d_6) of compound **BipCOOHBF₂.**

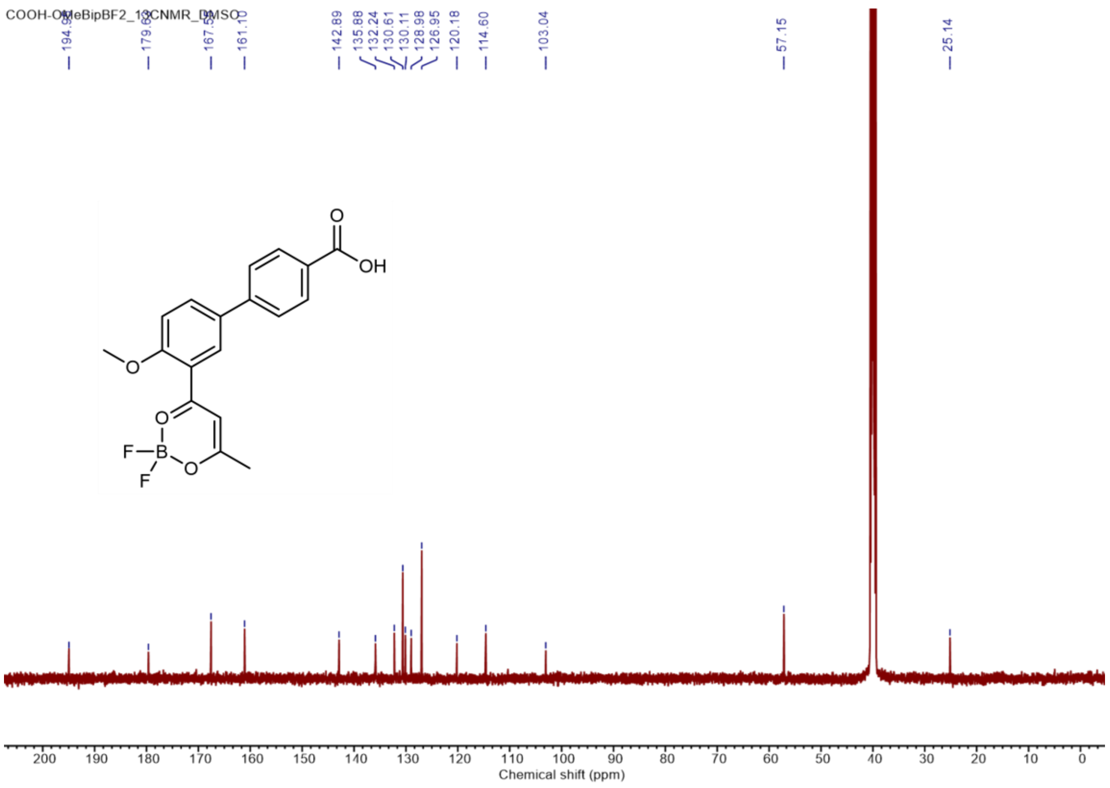

Supplementary Fig. 103. ^{13}C NMR spectra (126 MHz, DMSO-d_6) of compound **BipCOOHBF₂.**

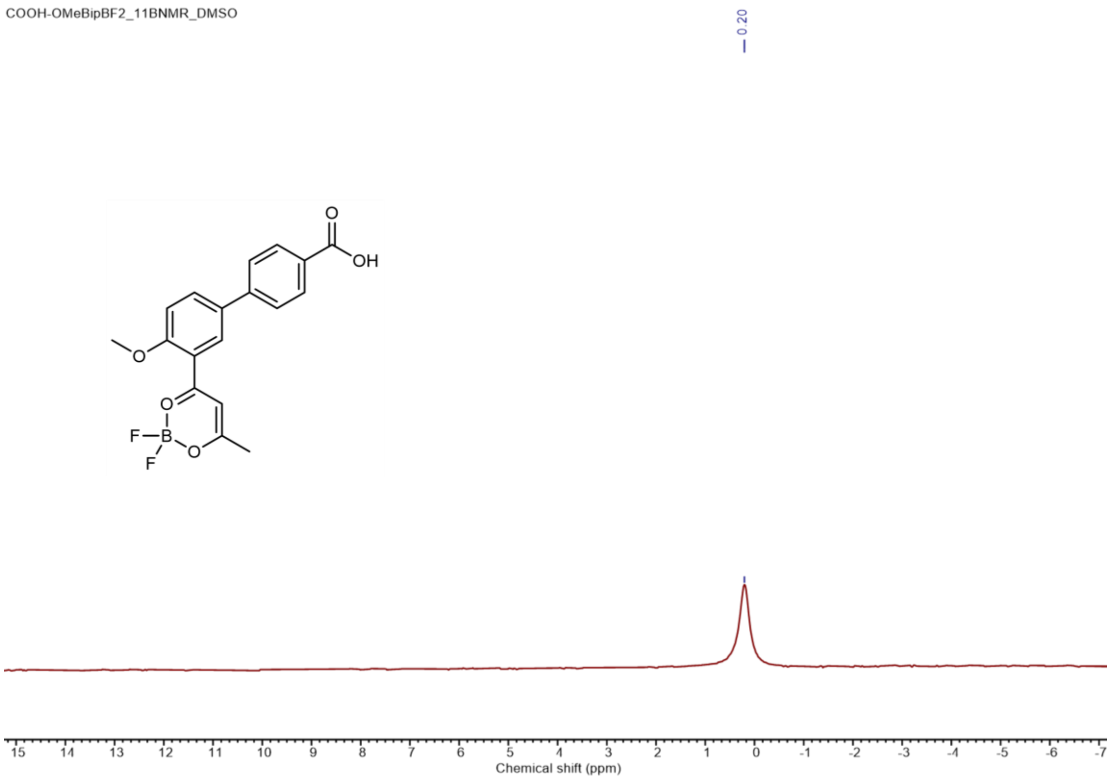

Supplementary Fig. 104. ^{11}B NMR spectra (128 MHz, DMSO-d_6) of compound **BipCOOHBF$_2$.**

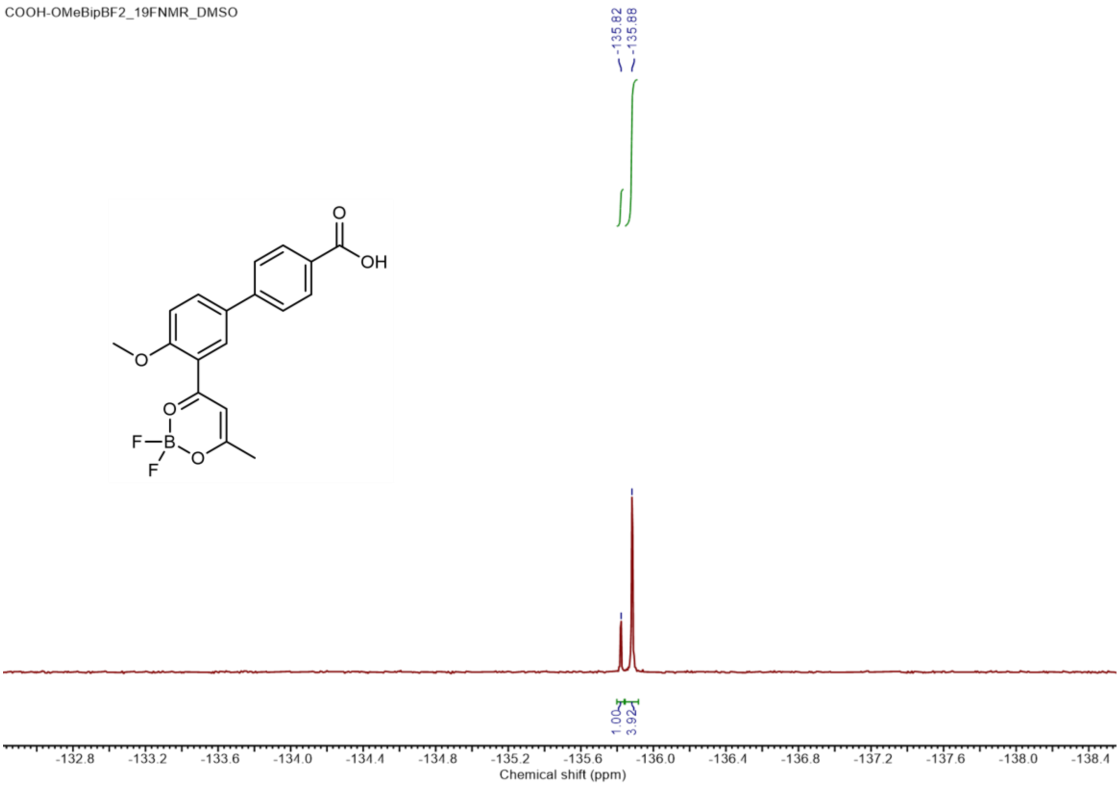

Supplementary Fig. 105. ^{19}F NMR spectra (376 MHz, DMSO-d_6) of compound **BipCOOHBF$_2$**.

Qualitative Analysis Report

**Data Filename**	2019115-LX-1.d	**Sample Name**	LX-1
**Sample Type**	Sample	**Position**	Vial 61
**Instrument Name**	Instrument 1	**User Name**	
**Acq Method**	IDJ4-75V.m	**Acquired Time**	10/18/2023 3:48:17 PM
**IRM Calibration Status**	Success	**DA Method**	FGFUS-C18.m
**Comment**			

Sample Group **Info.**
Acquisition SW 6200 series TOF/6500 series
Version Q-TOF B.05.01 (B5125.3)

User Spectra

Fragmentor Voltage	Collision Energy	Ionization Mode
175	0	ESI

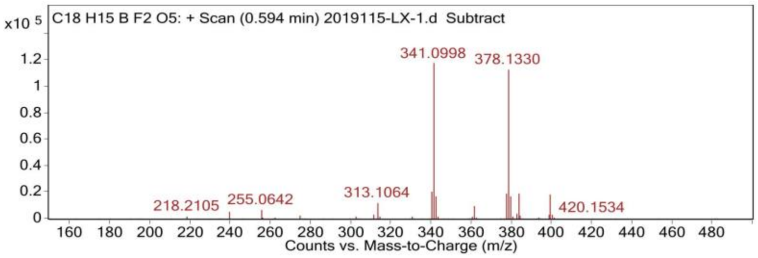

Peak List

m/z	z	Abund	Formula	Ion
377.1351	1	19523.9	C18 H15 B F2 O5	(M+NH4)+
378.1330	1	113189.34	C18 H15 B F2 O5	(M+NH4)+
379.1351	1	17176.12	C18 H15 B F2 O5	(M+NH4)+
380.1386	1	1947.63	C18 H15 B F2 O5	(M+NH4)+
382.0914	1	4072.79	C18 H15 B F2 O5	(M+Na)+
383.0869	1	19222.62	C18 H15 B F2 O5	(M+Na)+
384.0909	1	2943.98	C18 H15 B F2 O5	(M+Na)+

Formula Calculator Element Limits

Element	Min	Max
C	3	70
H	0	120
O	1	5
F	1	3
B	1	1

Formula Calculator Results

Ion Formula	m/z	m/z (Calc)	DBE	Diff (ppm)	Score (MFG)
C21 H18 B F N O4	377.1351	377.1344	15	-2.02	98.47
C18 H19 B F2 N O5	377.1351	377.1355	11	1.16	99.49
C18 H15 B F2 Na O5	382.0914	382.0909	11	-1.36	99.3

--- End Of Report ---

Agilent Technologies Page 1 of 1 Printed at: 4:39 PM on: 10/18/2023

Supplementary Fig. 106. HRMS spectra of compound **BipCOOHBF$_2$**.

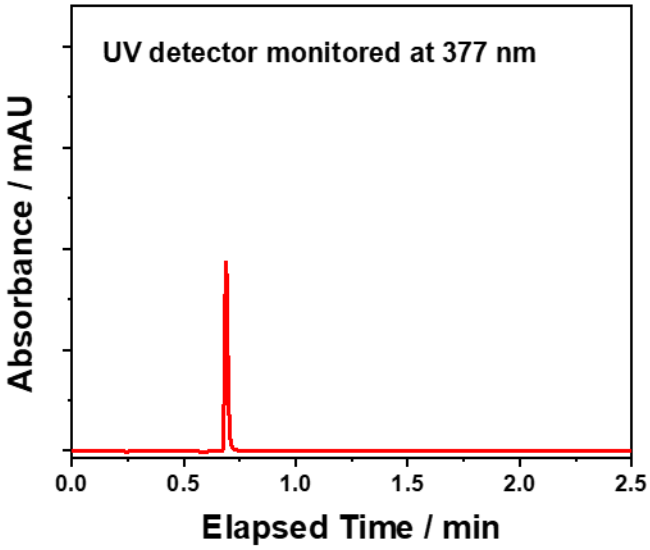

Supplementary Fig. 107. HPLC of compound **BipCOOHBF$_2$**.

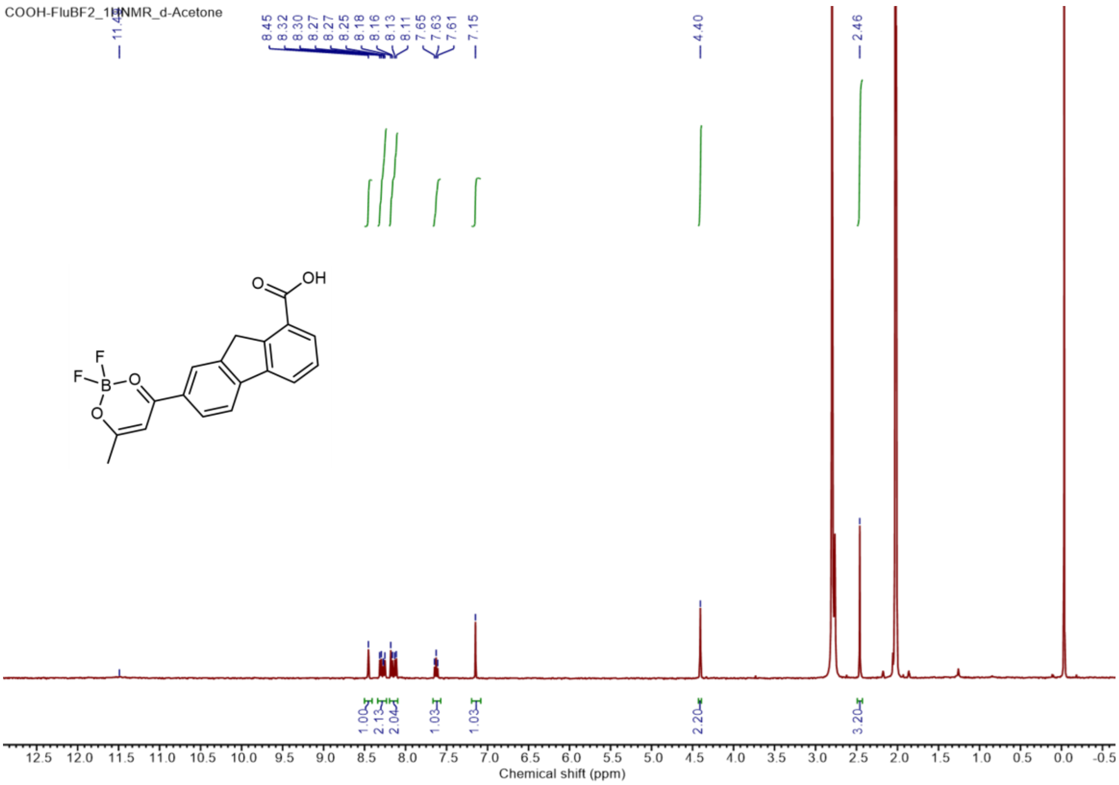

Supplementary Fig. 108. ^{1}H NMR spectra (400 MHz, Acetone-d_6) of compound **FluCOOHBF$_2$**.

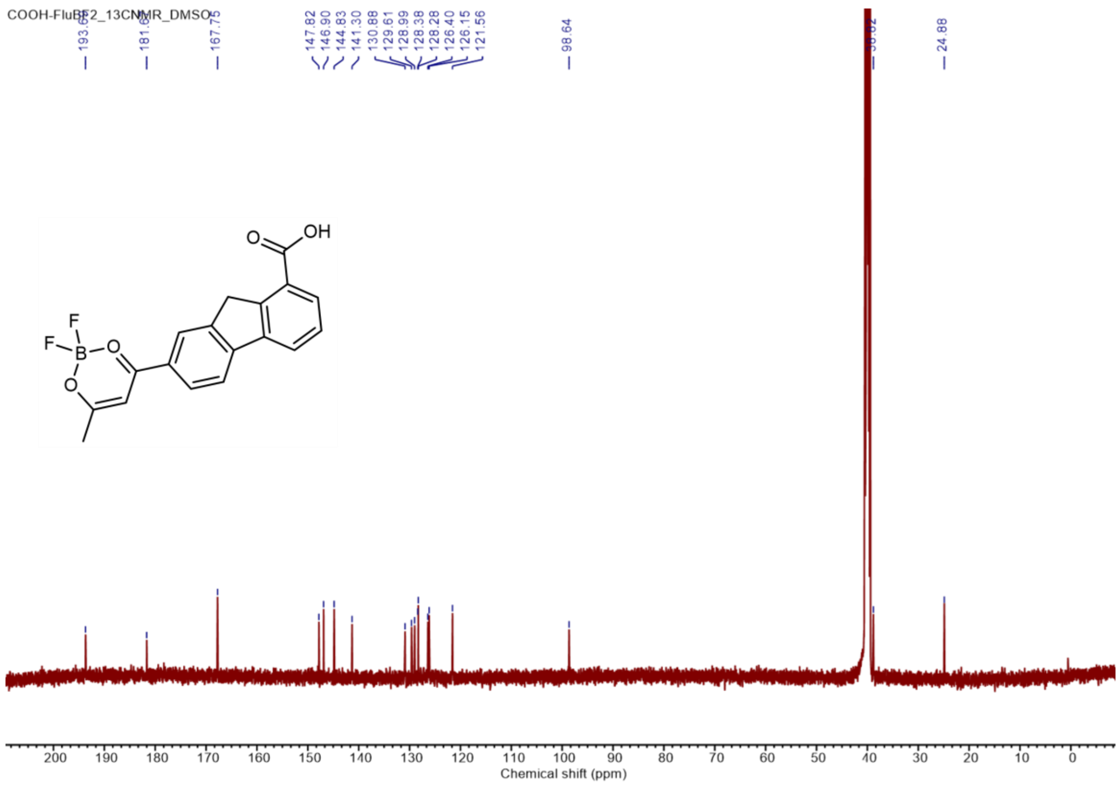

Supplementary Fig. 109. ^{13}C NMR spectra (126 MHz, DMSO-d_6) of compound **FluCOOHBF2**.

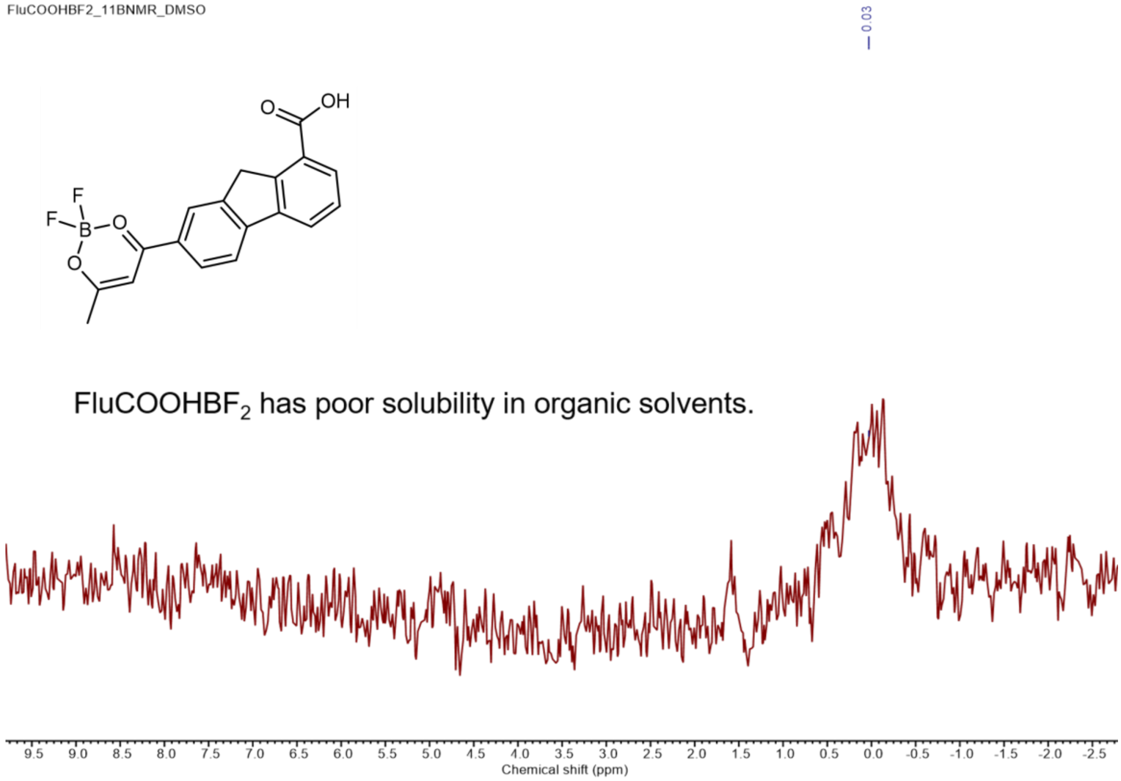

Supplementary Fig. 110. ^{11}B NMR spectra (128 MHz, DMSO-d_6) of compound **FluCOOHBF2**.

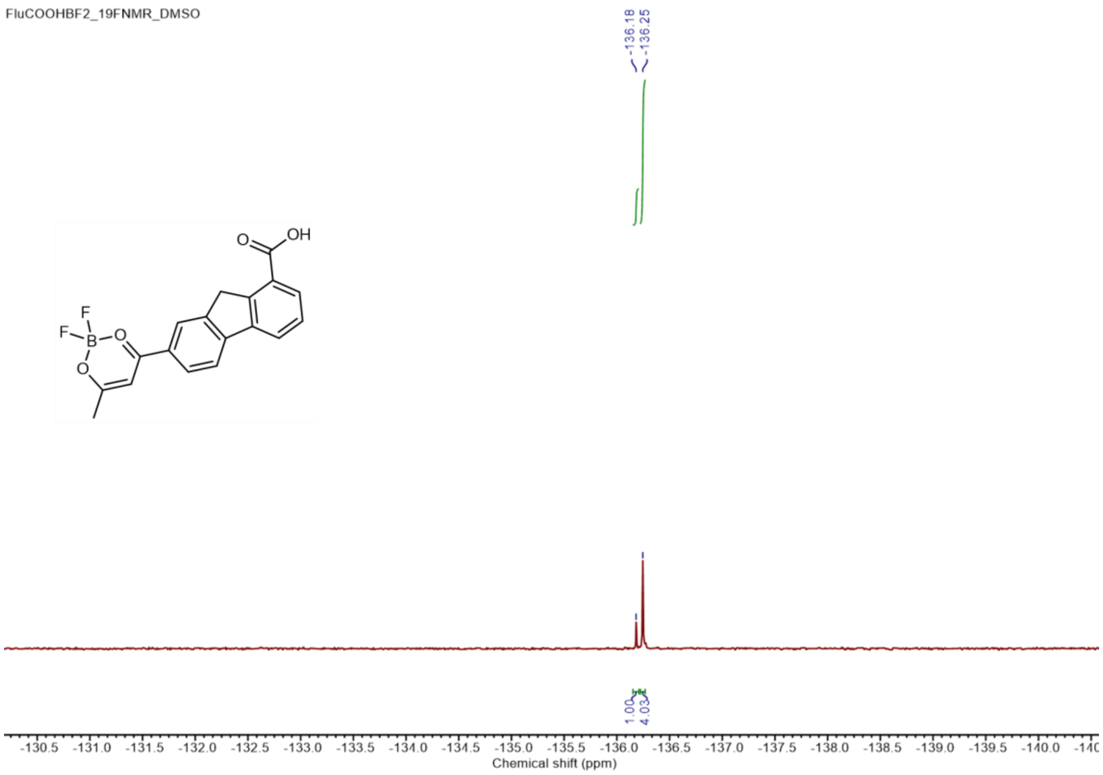

Supplementary Fig. 111. ^{19}F NMR spectra (376 MHz, DMSO-d_6) of compound **FluCOOHBF$_2$**.

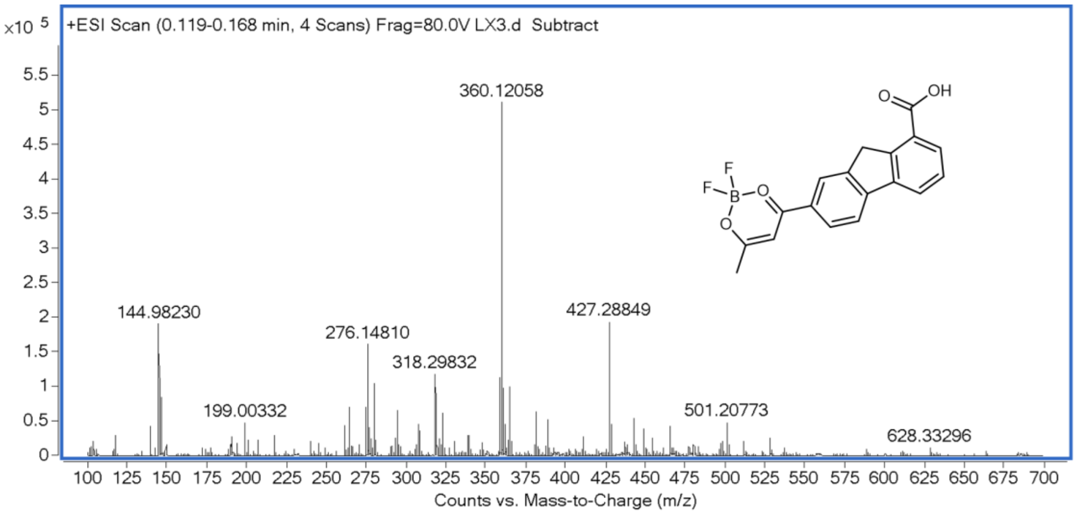

Supplementary Fig. 112. HRMS spectra of compound **FluCOOHBF$_2$**.

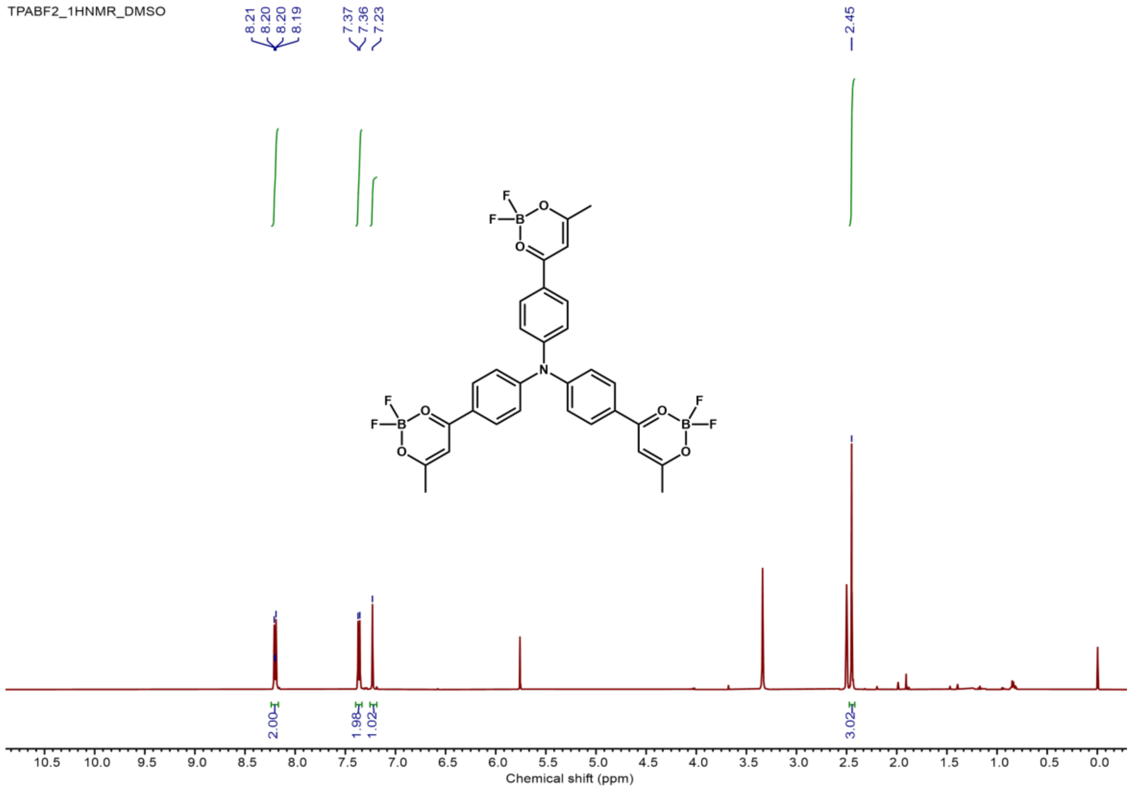

Supplementary Fig. 113. ^{1}H NMR spectra (500 MHz, DMSO-d_6) of compound **TPABF$_2$**.

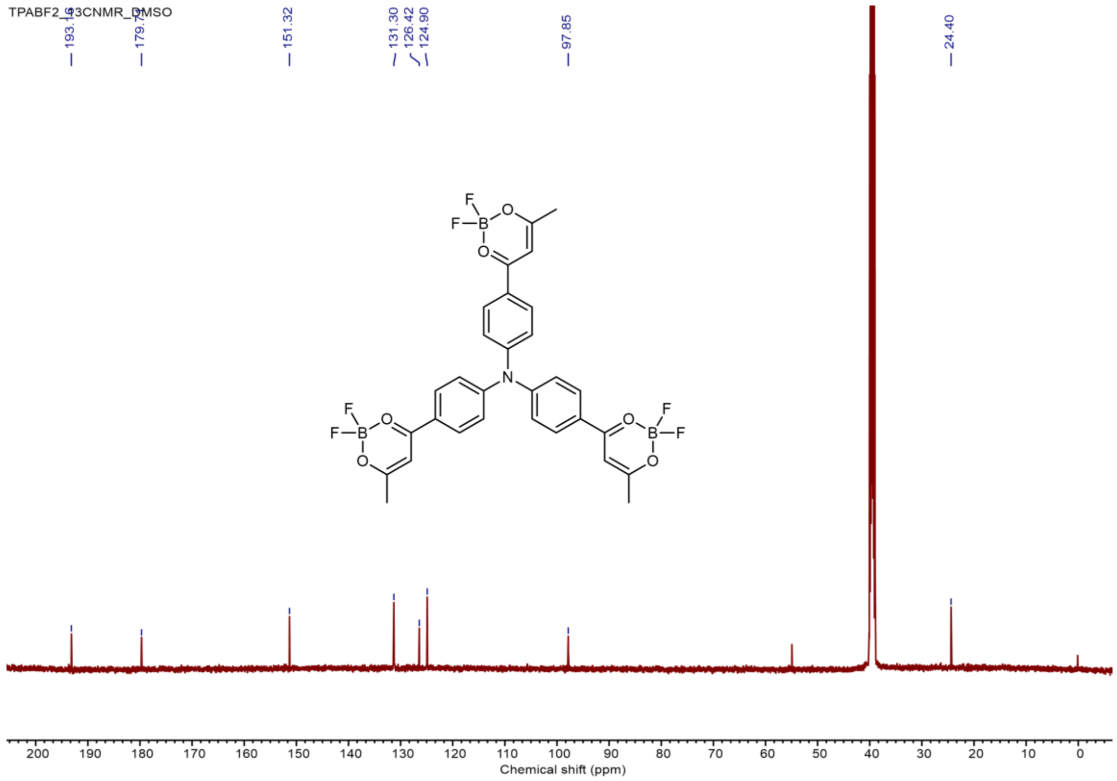

Supplementary Fig. 114. ^{13}C NMR spectra (126 MHz, DMSO-d_6) of compound **TPABF$_2$**.

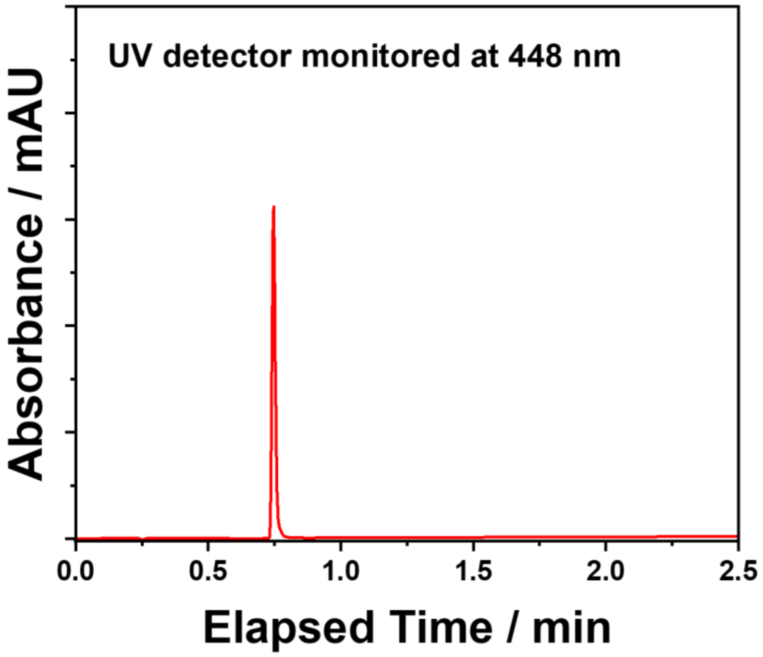

Supplementary Fig. 115. HPLC of compound **$TPABF_2$**.

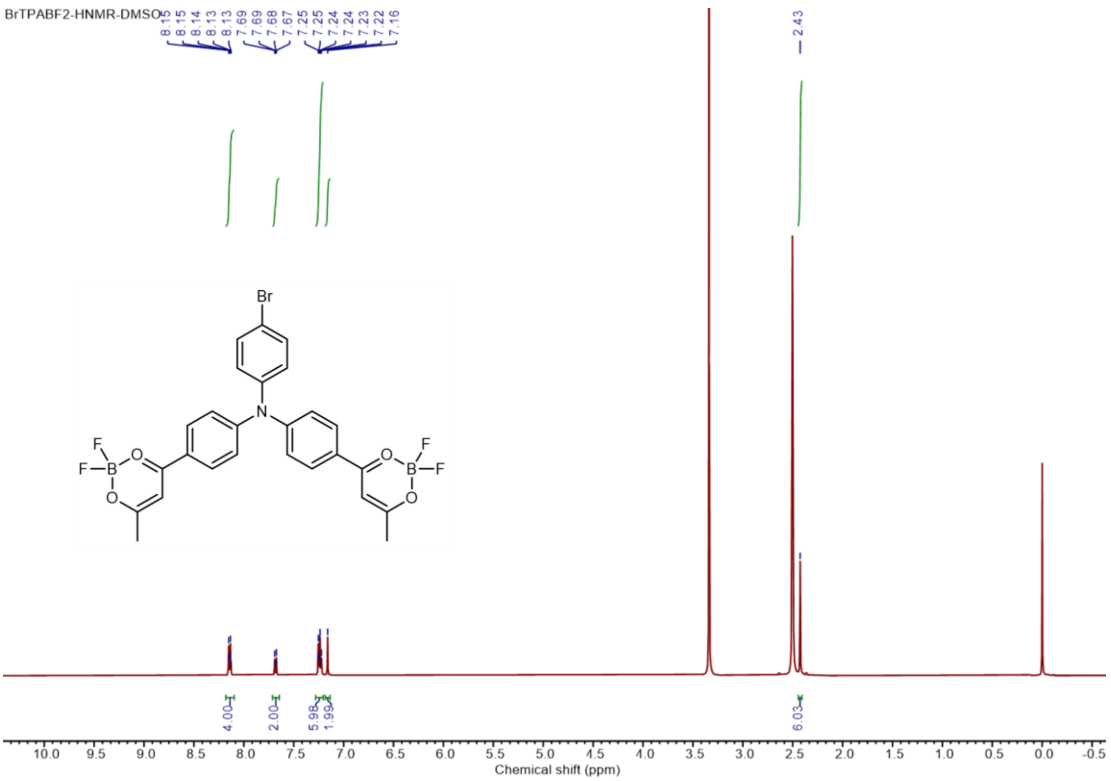

Supplementary Fig. 116. ^{1}H NMR spectra (500 MHz, DMSO-d_6) of compound **$BrTPABF_2$**.

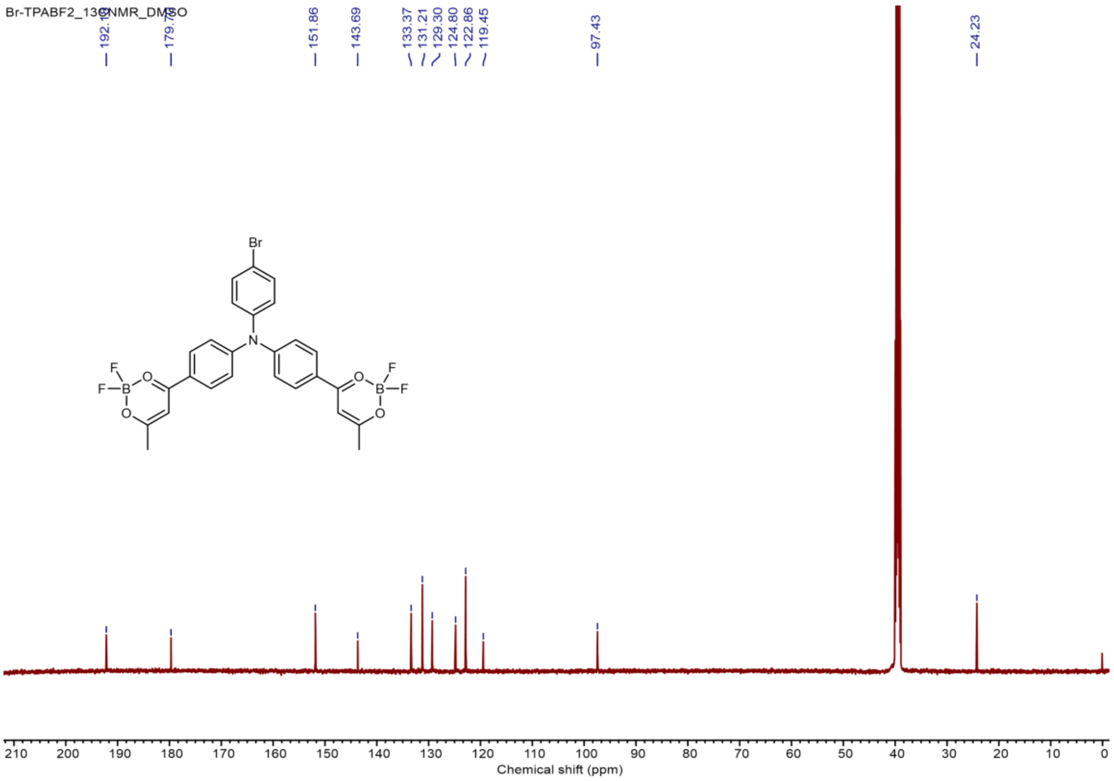

Supplementary Fig. 117. ^{11}C NMR spectra (126 MHz, DMSO-d_6) of compound **BrTPABF$_2$**.

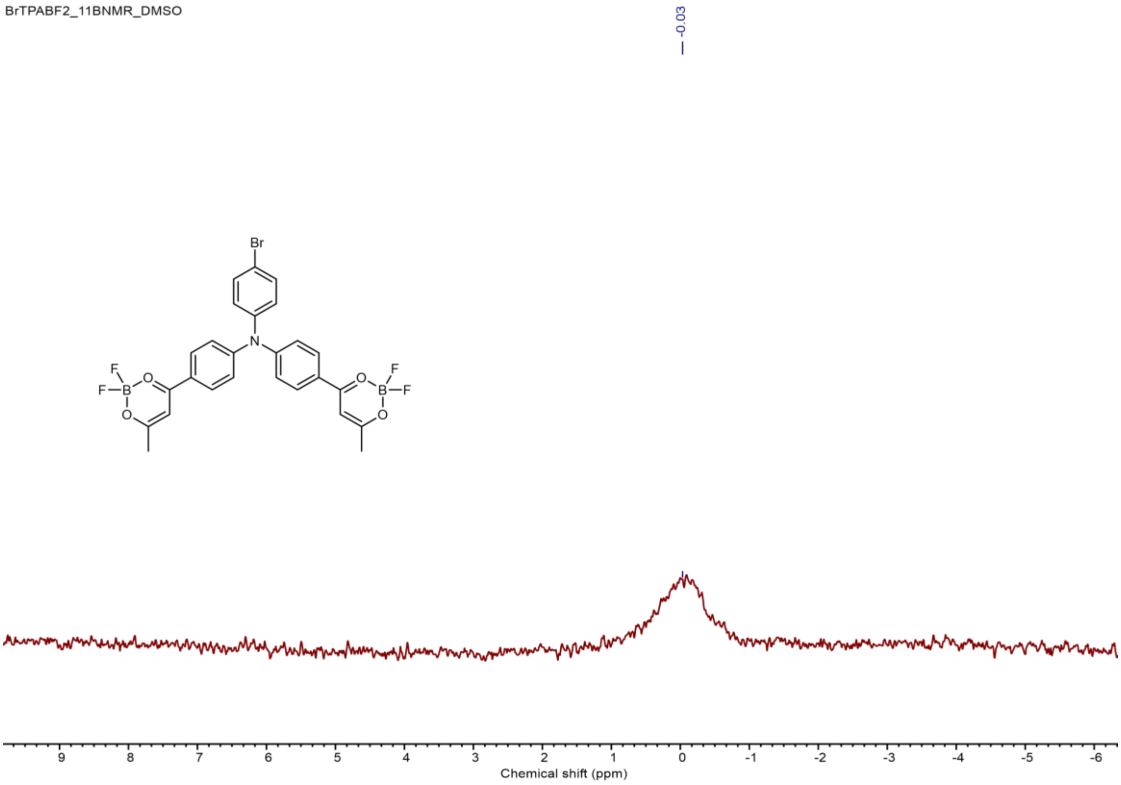

Supplementary Fig. 118. ^{11}B NMR spectra (128 MHz, DMSO-d_6) of compound **BrTPABF$_2$**.

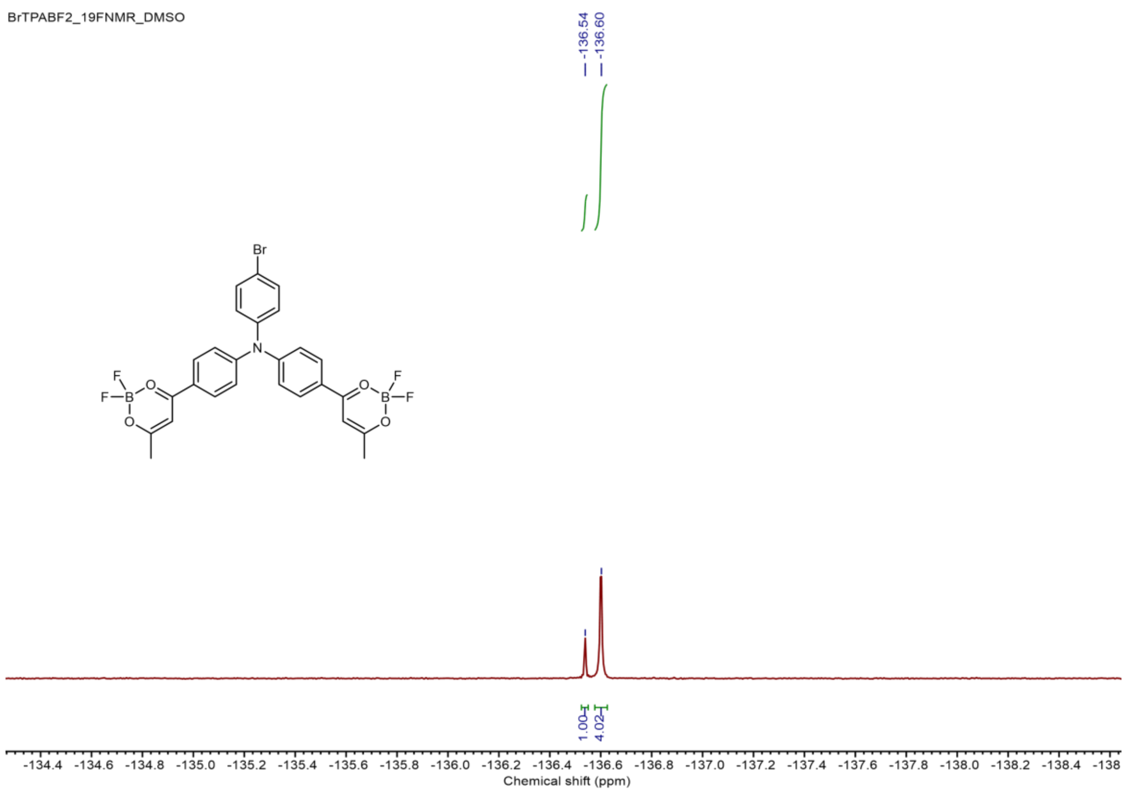

Supplementary Fig. 119. ^{19}F NMR spectra (376 MHz, DMSO-d_6) of compound **BrTPABF$_2$**.

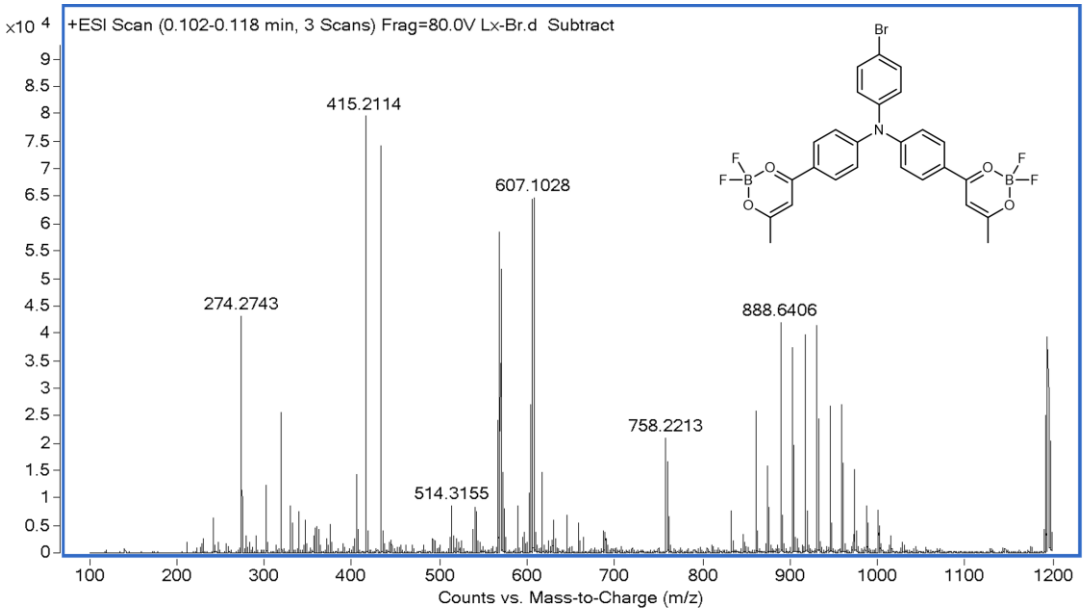

Supplementary Fig. 120. HRMS spectra of compound **BrTPABF$_2$**.

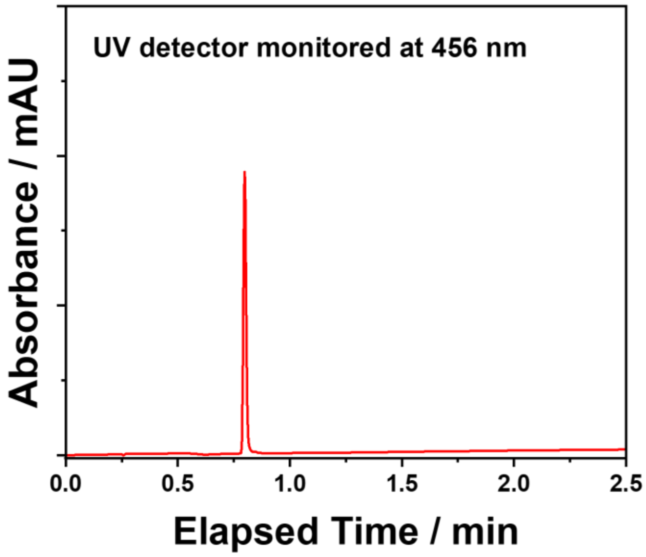

Supplementary Fig. 121. HPLC of compound **BrTPABF$_2$**.

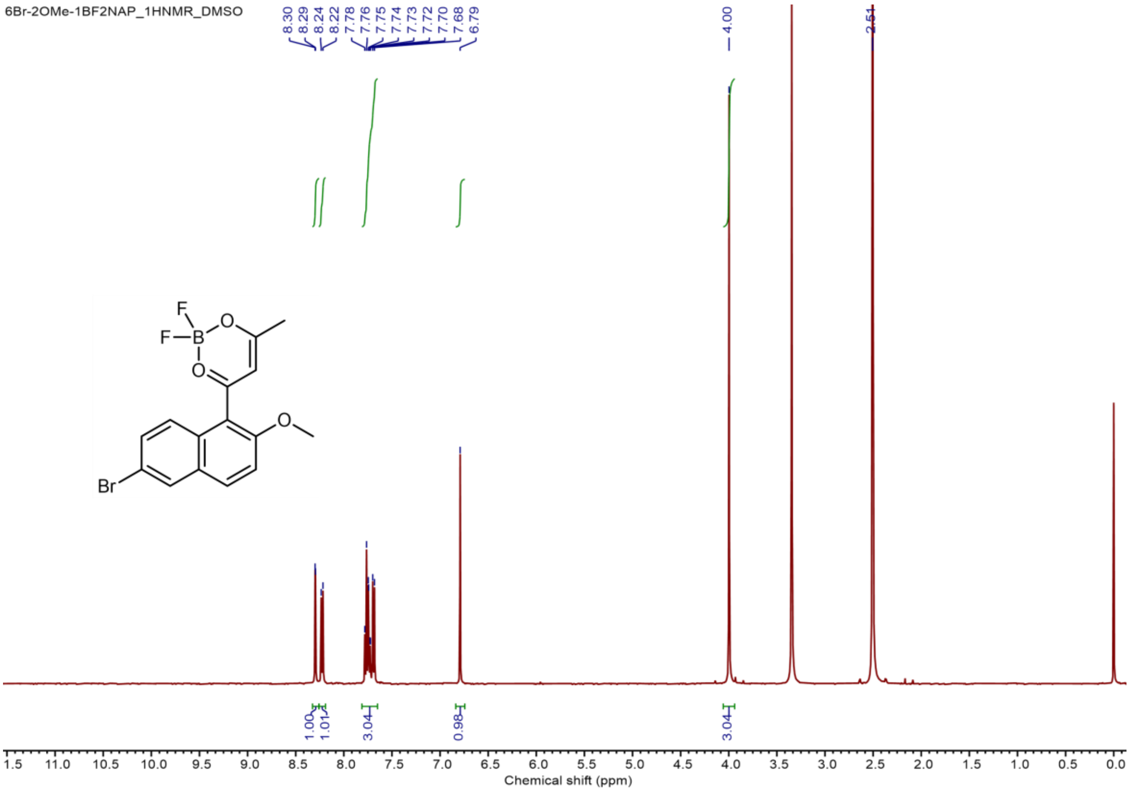

Supplementary Fig. 122. ^{1}H NMR spectra (500 MHz, DMSO-d_6) of compound **BrNAPBF$_2$**.

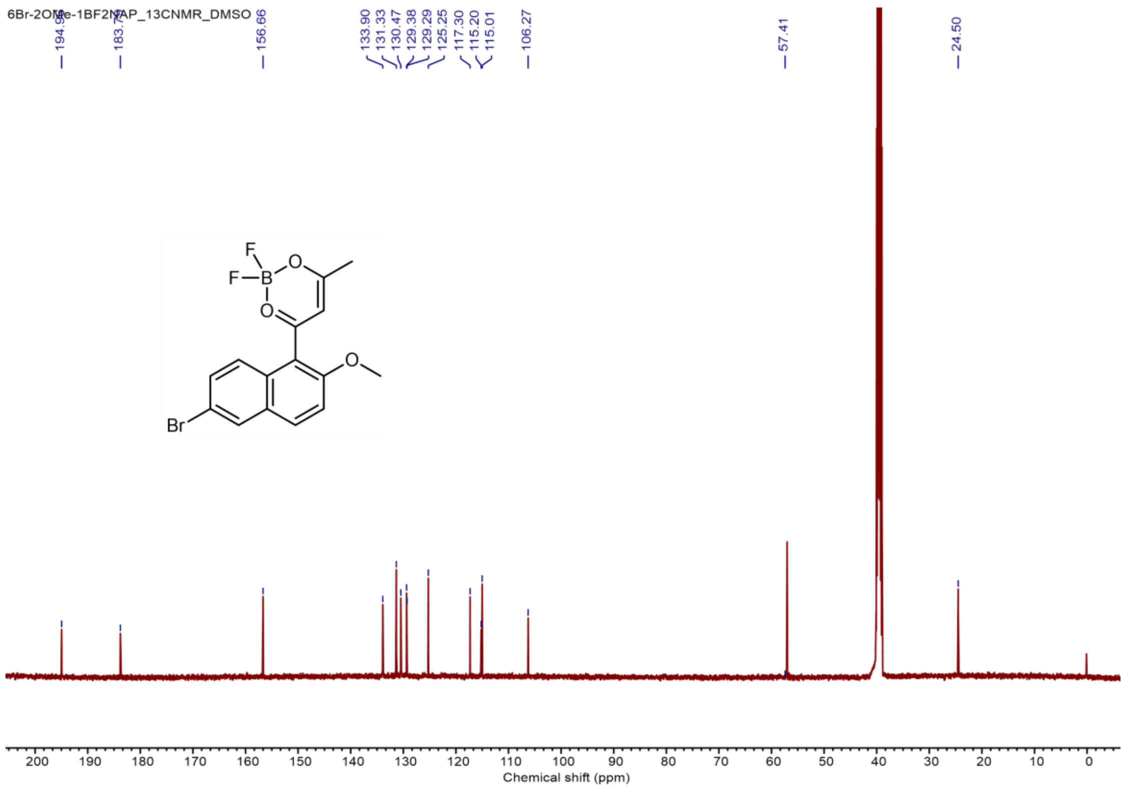

Supplementary Fig. 123. ^{13}C NMR spectra (126 MHz, DMSO-d_6) of compound **BrNAPBF$_2$**.

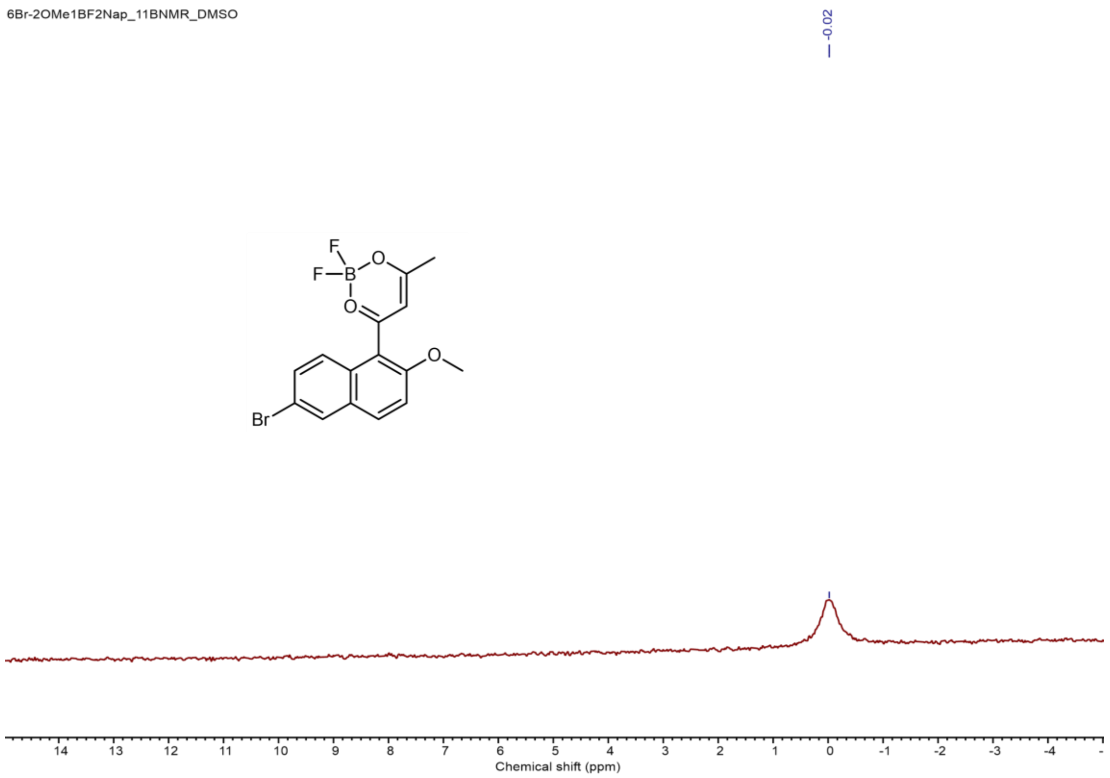

Supplementary Fig. 124. ^{11}B NMR spectra (128 MHz, DMSO-d_6) of compound **BrNAPBF$_2$**.

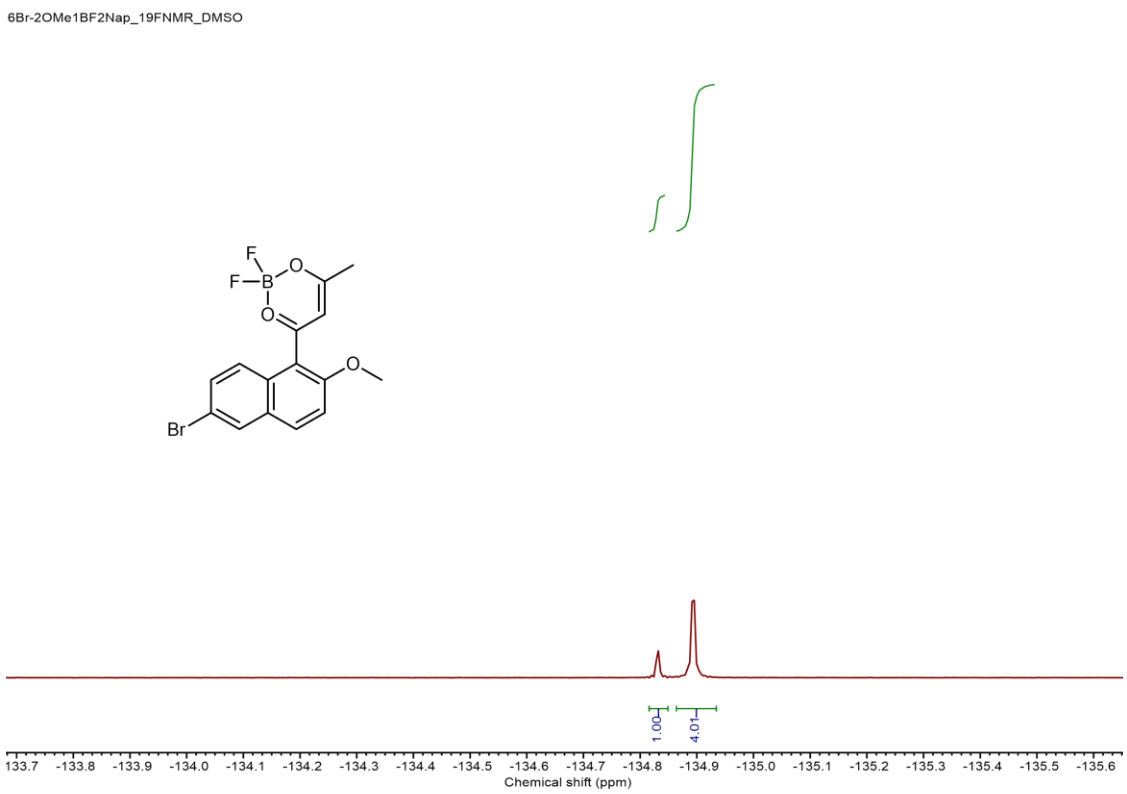

Supplementary Fig. 125. ^{19}F NMR spectra (376 MHz, DMSO-d_6) of compound **BrNAPBF$_2$**.

Qualitative Analysis Report

**Data Filename**	2019115-LX-7.d	**Sample Name**	LX-7
**Sample Type**	Sample	**Position**	Vial 67
**Instrument Name**	Instrument 1	**User Name**	
**Acq Method**	IDJ4-75V.m	**Acquired Time**	10/18/2023 3:31:52 PM
**IRM Calibration Status**	Success	**DA Method**	FGFUS-C18.m
**Comment**			

**Sample Group**		**Info.**
**Acquisition SW Version**	6200 series TOF/6500 series Q-TOF B.05.01 (B5125.3)	

User Spectra

Fragmentor Voltage	Collision Energy	Ionization Mode
175	0	ESI

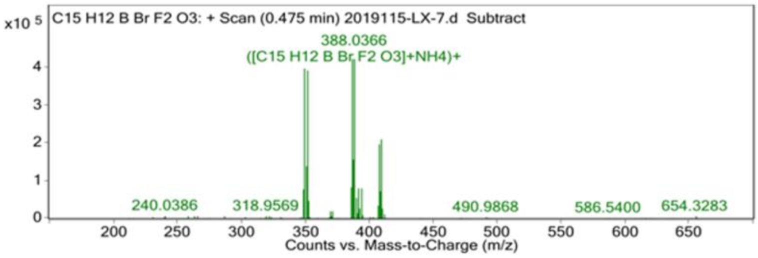

Peak List

m/z	z	Abund	Formula	Ion
385.0406	1	83146.62	C15 H12 B Br F2 O3	(M+NH4)+
386.0384	1	423143.38	C15 H12 B Br F2 O3	(M+NH4)+
387.0398	1	157114.25	C15 H12 B Br F2 O3	(M+NH4)+
388.0366	1	424479.25	C15 H12 B Br F2 O3	(M+NH4)+
389.0384	1	55670.05	C15 H12 B Br F2 O3	(M+NH4)+

Formula Calculator Element Limits

Element	Min	Max
C	3	70
H	0	120
O	1	5
F	1	3
B	1	1
Br	1	1

Formula Calculator Results

Ion Formula	m/z	m/z (Calc)	DBE	Diff (ppm)	Score (MFG)
C18 H15 B Br F N O2	385.0406	385.0394	13	-3.25	96.06
C15 H16 B Br F2 N O3	385.0406	385.0406	9	-0.13	99.99
C12 H17 B Br F3 N O4	385.0406	385.0417	5	2.98	96.66

--- End Of Report ---

Agilent Technologies Page 1 of 1 Printed at: 5:02 PM on: 10/18/2023

Supplementary Fig. 126. HRMS spectra of compound **BrNAPBF$_2$**.

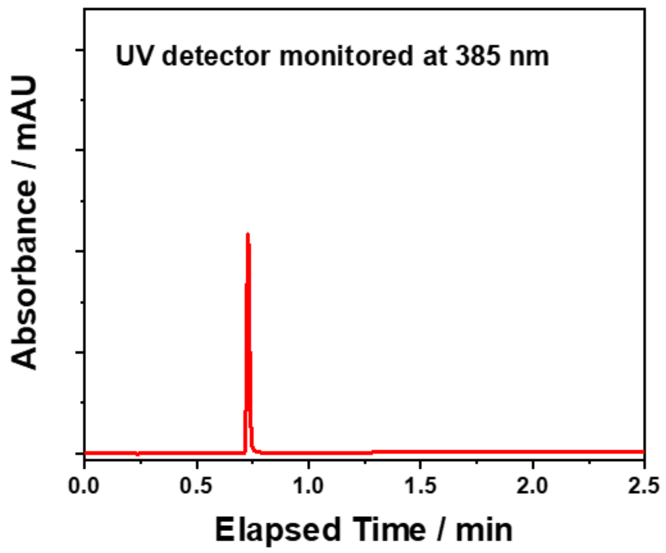

Supplementary Fig. 127. HLPC of compound **BrNAPBF$_2$**.

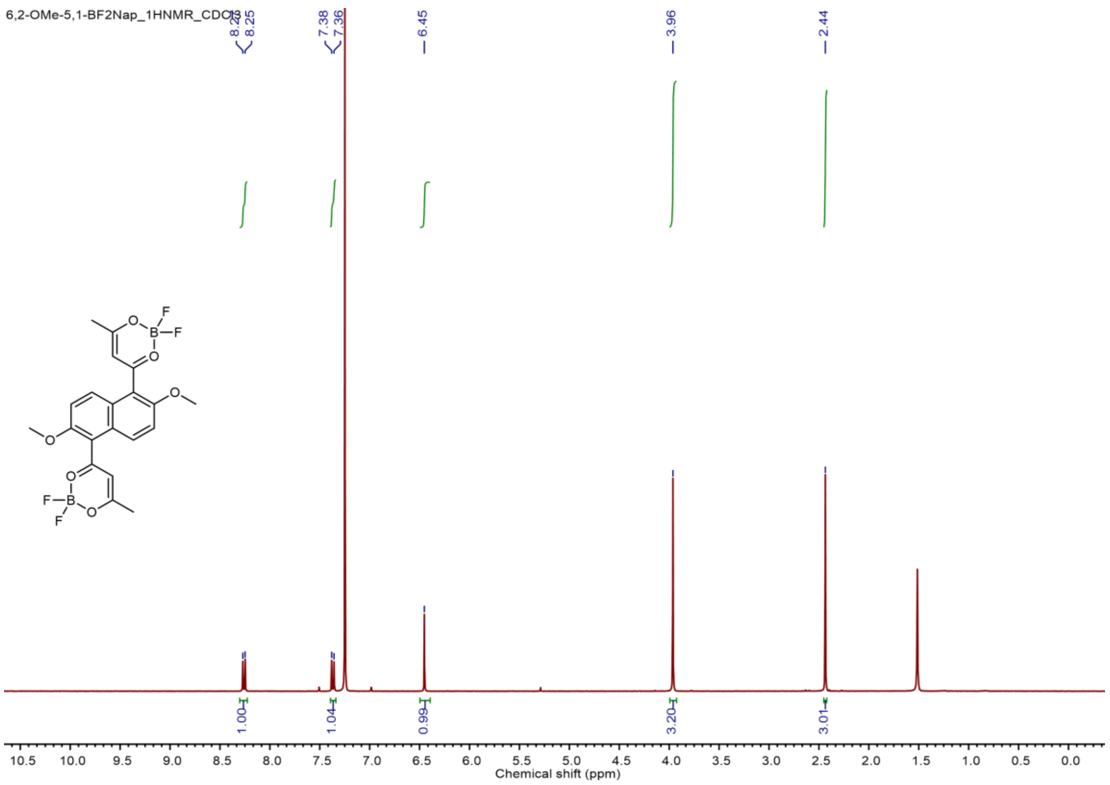

Supplementary Fig. 128. ^{1}H NMR spectra (400 MHz, Chloroform-*d*) of compound **OMe2NAPBF$_2$**.

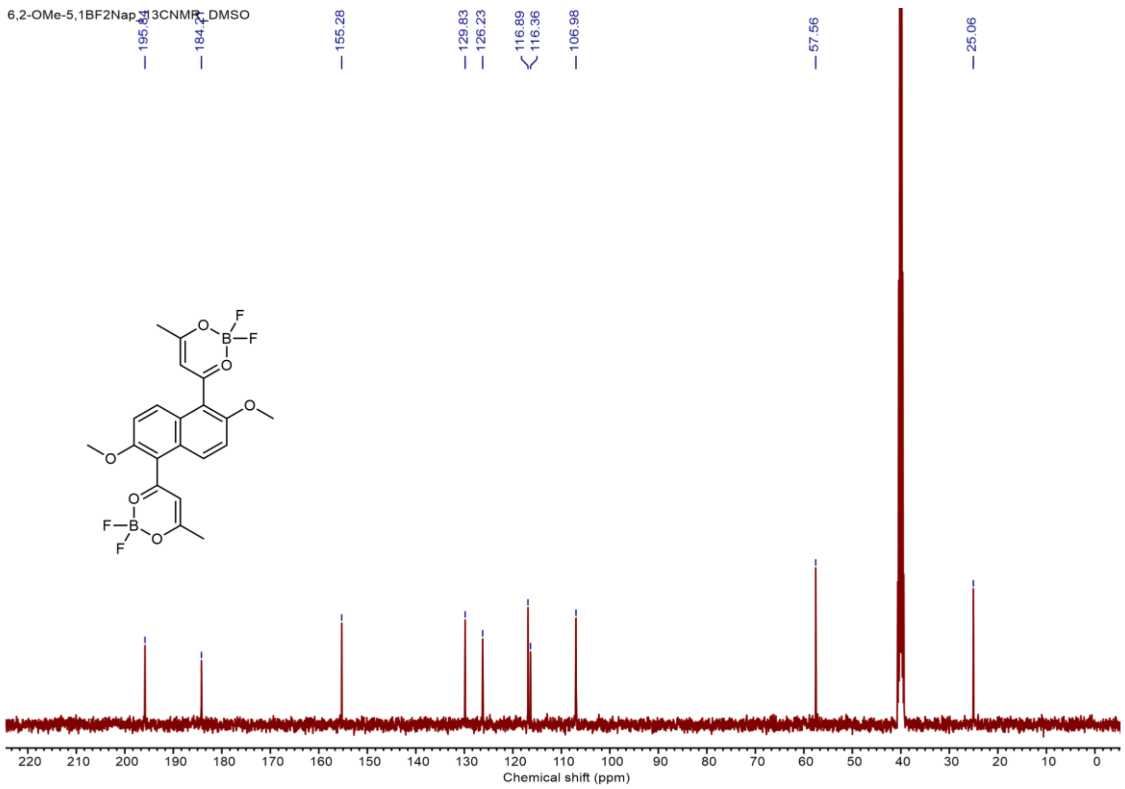

Supplementary Fig. 129. ^{13}C NMR spectra (101 MHz, DMSO-d_6) of compound **OMe2NAPBF$_2$**.

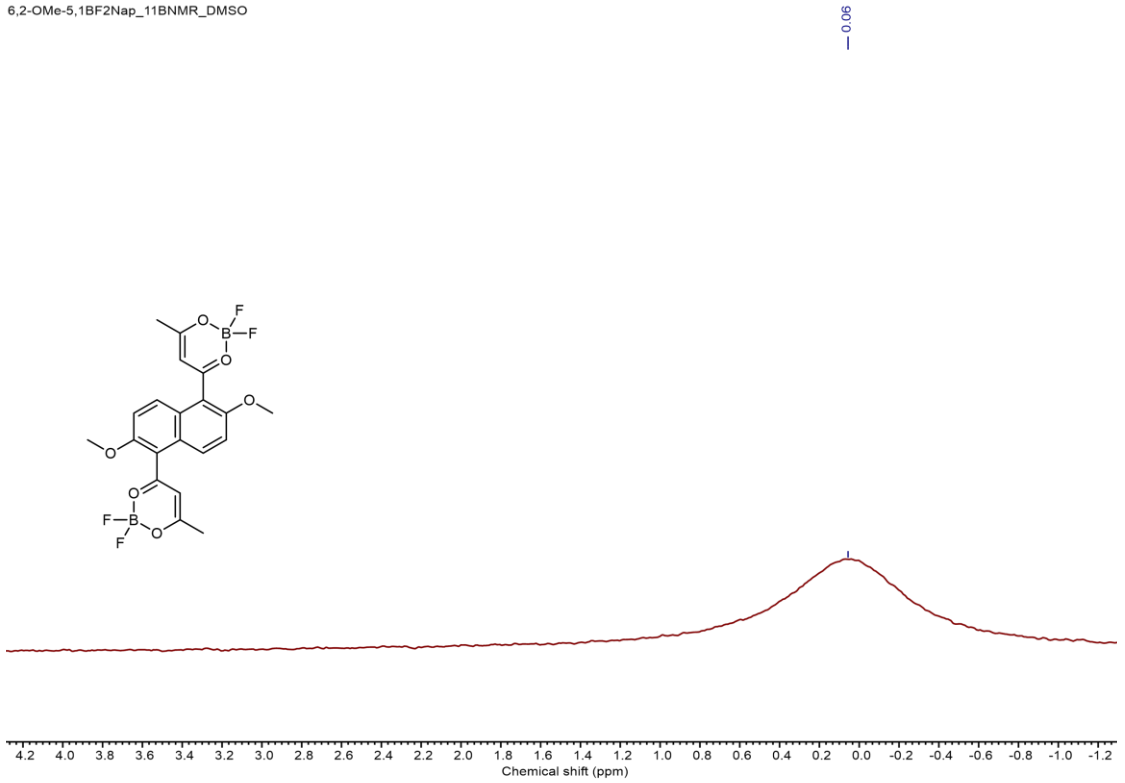

Supplementary Fig. 130. ^{11}B NMR spectra (128 MHz, DMSO-d_6) of compound **OMe2NAPBF$_2$**.

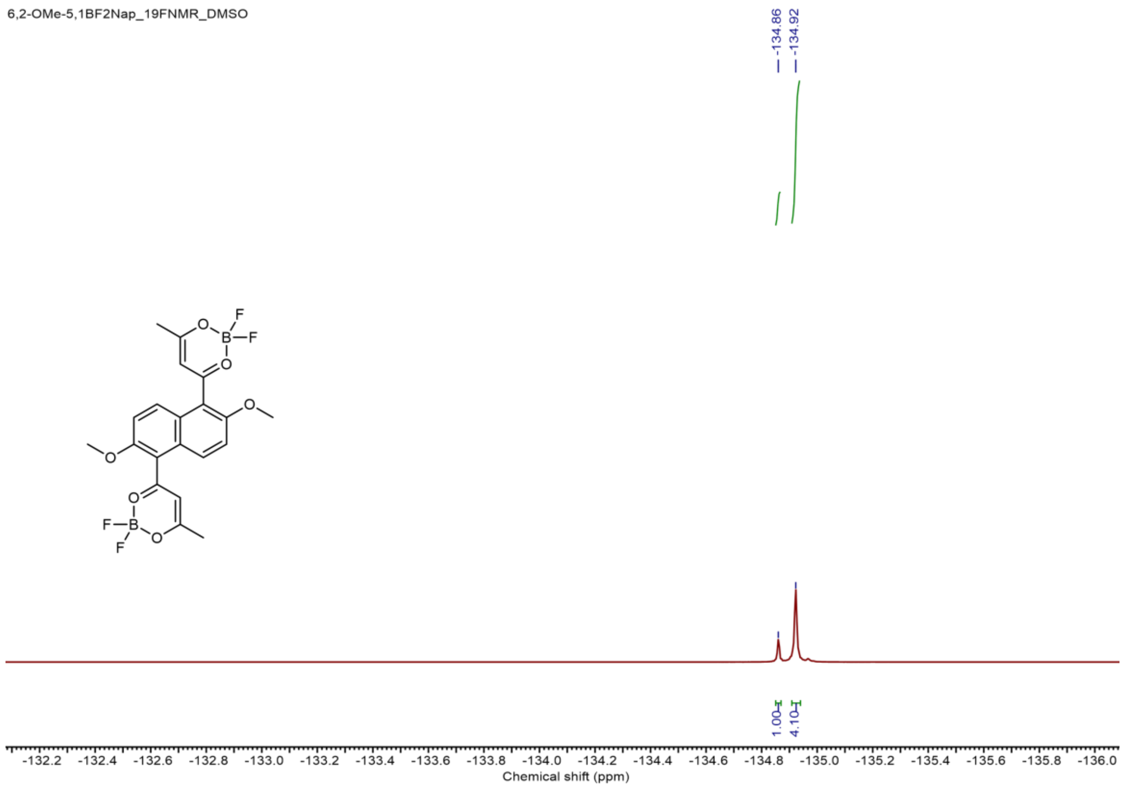

Supplementary Fig. 131. ^{19}F NMR spectra (376 MHz, DMSO-d_6) of compound **OMe2NAPBF$_2$**.

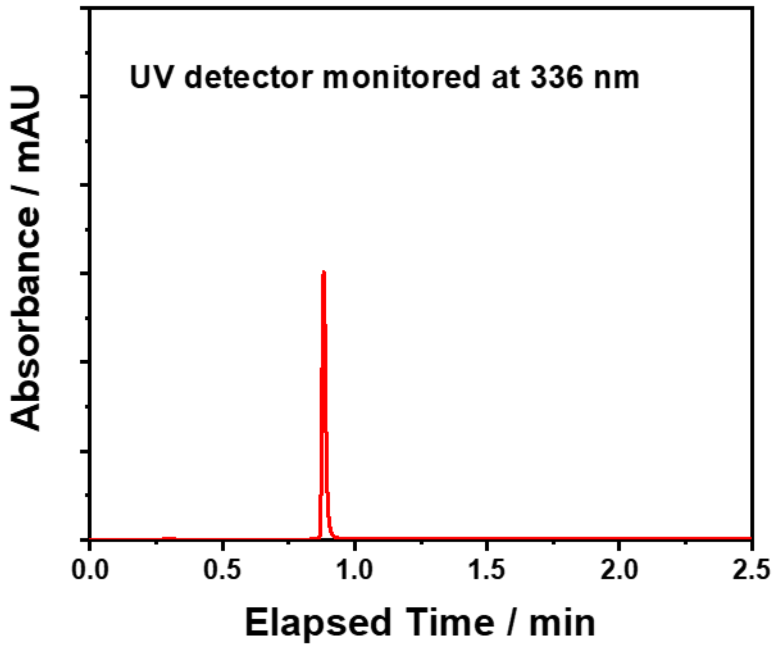

Supplementary Fig. 132. HPLC of compound **OMe2NAPBF$_2$**.

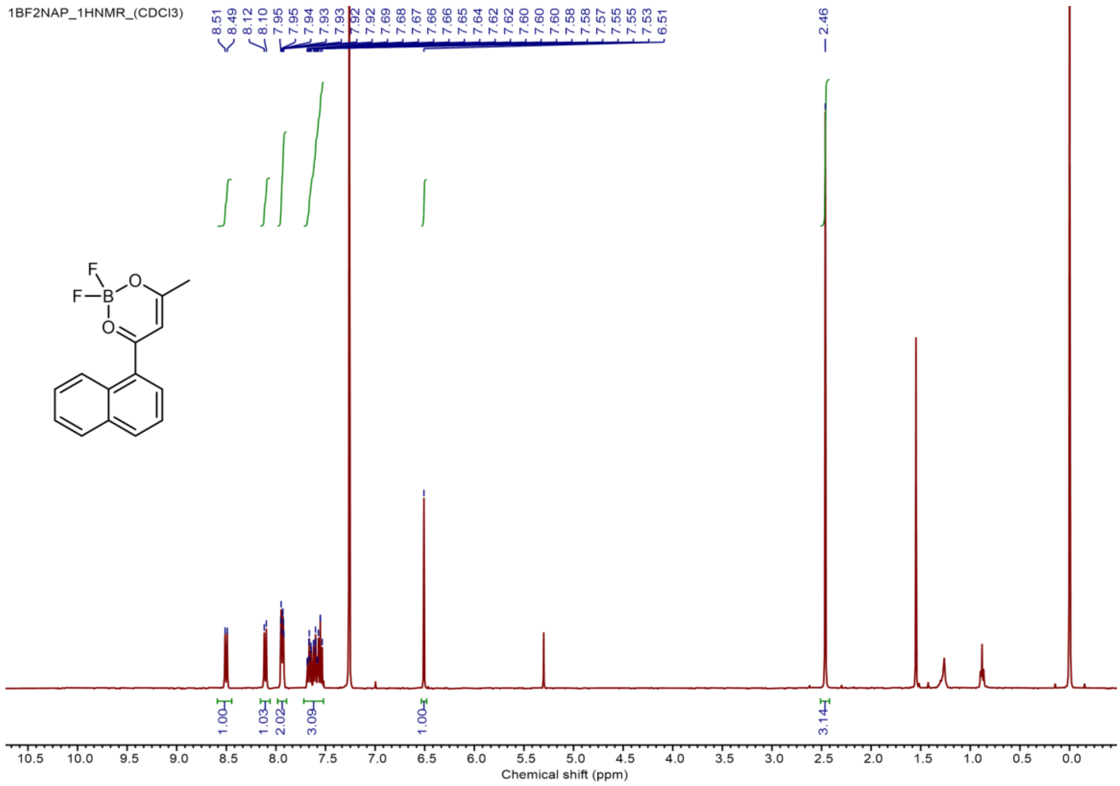

Supplementary Fig. 133. ^{1}H NMR spectra (400 MHz, Chloroform-*d*) of compound **NAP1BF$_2$**.

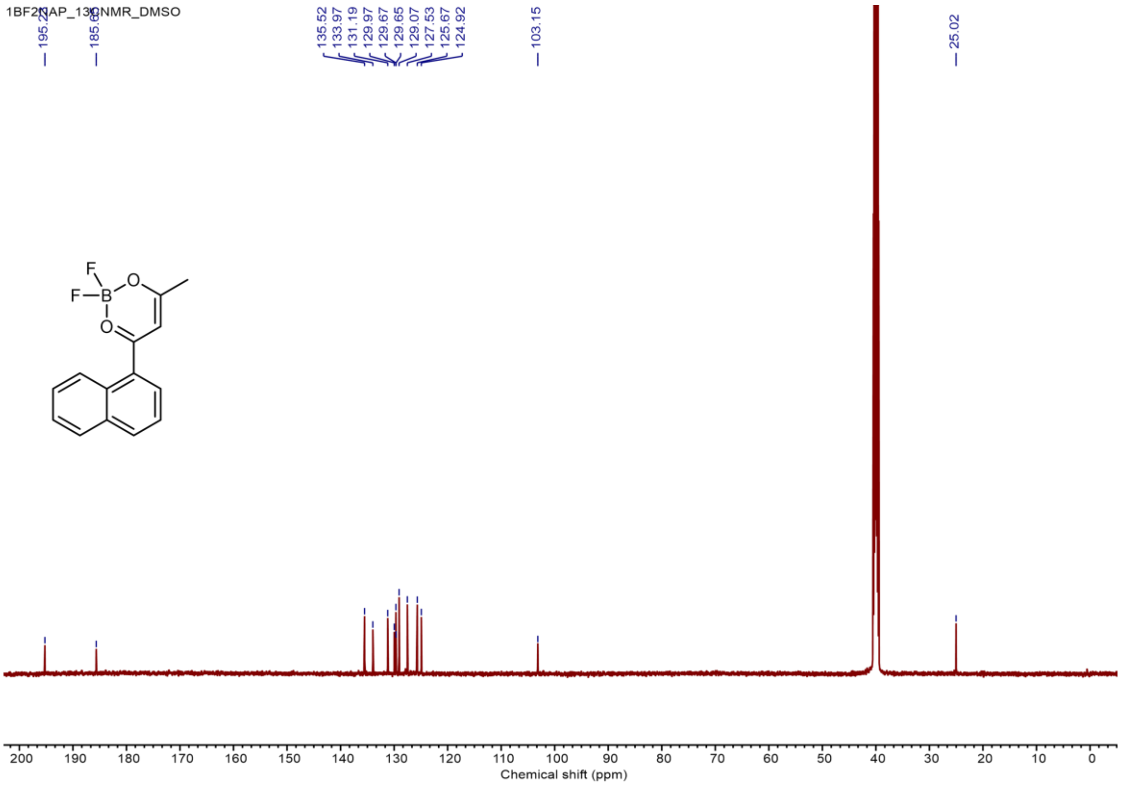

Supplementary Fig. 134. ^{13}C NMR spectra (126 MHz, DMSO-d_6) of compound **NAP1BF$_2$**.

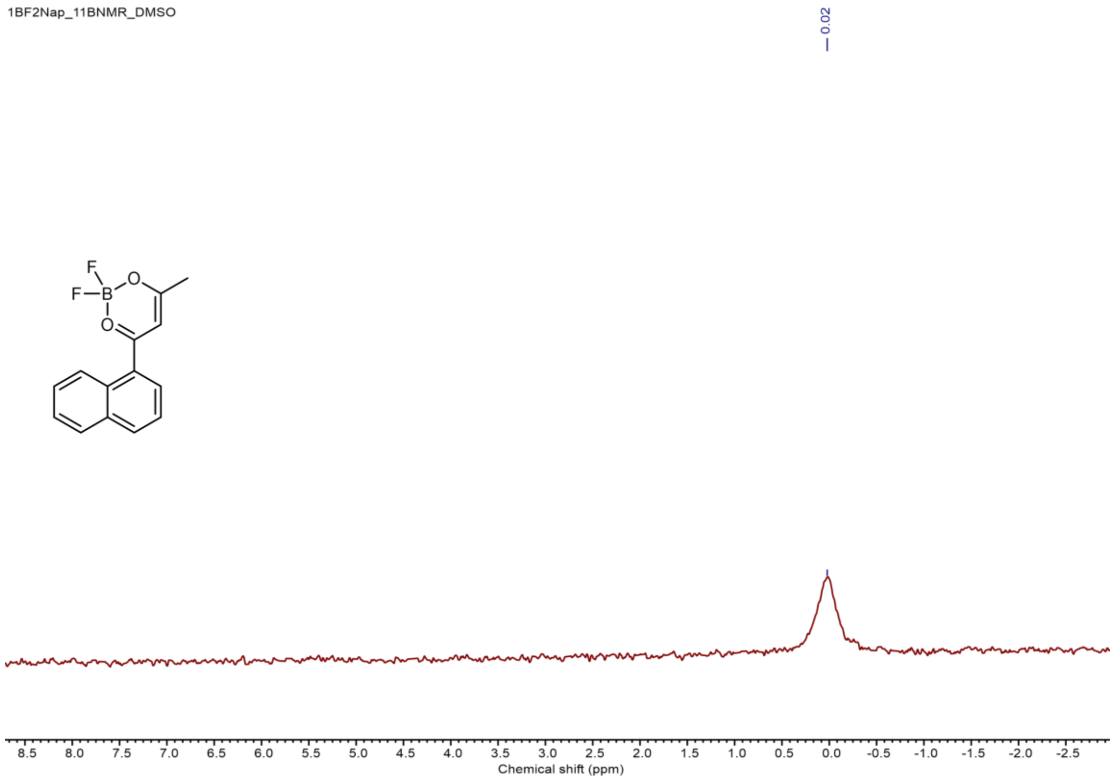

Supplementary Fig. 135. ^{11}B NMR spectra (128 MHz, DMSO-d_6) of compound **NAP1BF$_2$**.

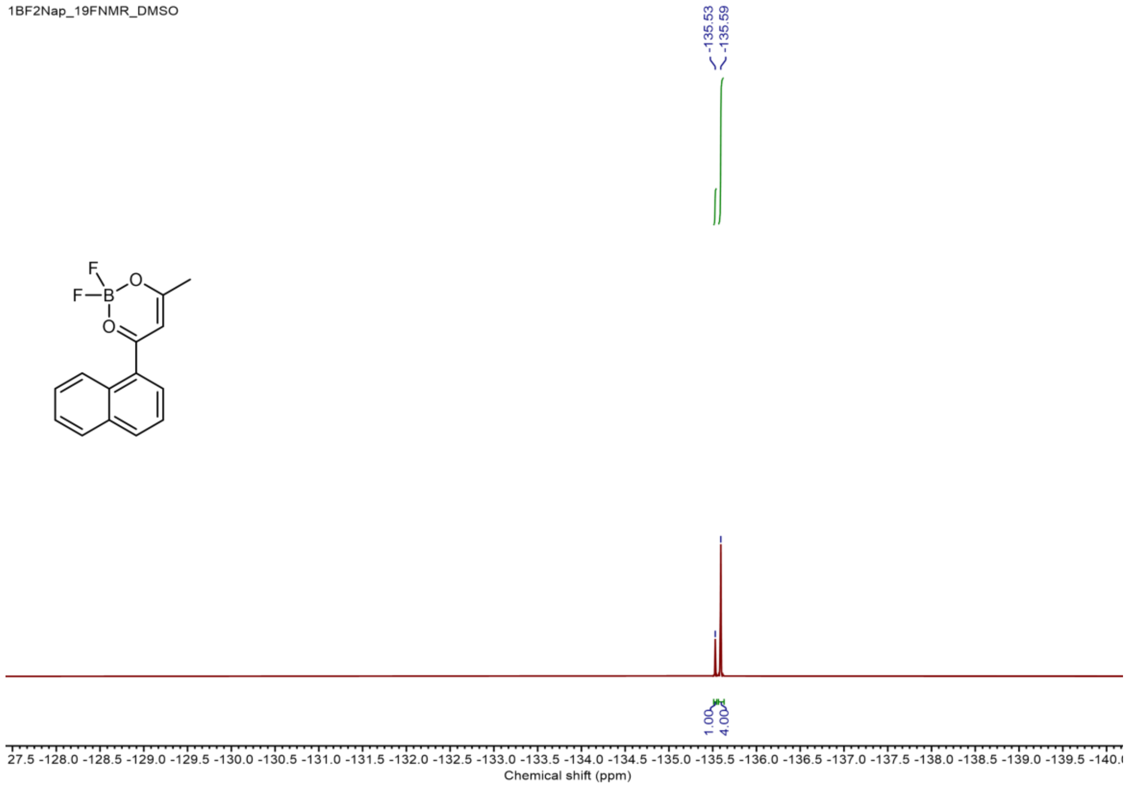

Supplementary Fig. 136. ^{19}F NMR spectra (376 MHz, DMSO-d_6) of compound **NAP1BF$_2$**.

Qualitative Analysis Report

**Data Filename**	2019115-LX-8.d	**Sample Name**	LX-8
**Sample Type**	Sample	**Position**	Vial 68
**Instrument Name**	Instrument 1	**User Name**	
**Acq Method**	IDJ4-75V.m	**Acquired Time**	10/18/2023 3:37:21 PM
**IRM Calibration Status**	Success	**DA Method**	FGFUS-C18.m
**Comment**			

Sample Group **Info.**
Acquisition SW 6200 series TOF/6500 series
Version Q-TOF B.05.01 (B5125.3)

User Spectra

Fragmentor Voltage	Collision Energy	Ionization Mode
175	0	ESI

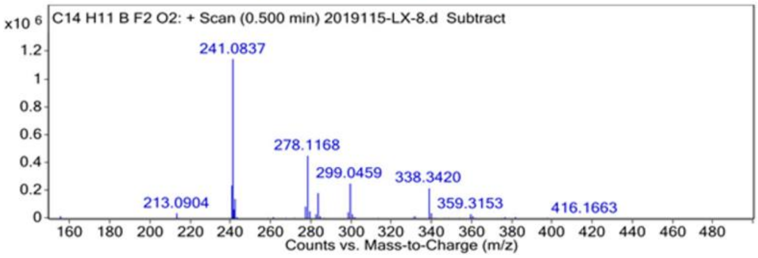

Peak List

m/z	z	Abund	Formula	Ion
277.1194	1	91421.05	C14 H11 B F2 O2	(M+NH4)+
278.1168	1	455276.5	C14 H11 B F2 O2	(M+NH4)+
279.1189	1	58326.85	C14 H11 B F2 O2	(M+NH4)+
282.0743	1	33972.89	C14 H11 B F2 O2	(M+Na)+
283.0719	1	183068.7	C14 H11 B F2 O2	(M+Na)+
284.0741	1	20845.13	C14 H11 B F2 O2	(M+Na)+

Formula Calculator Element Limits

Element	Min	Max
C	3	70
H	0	120
O	1	5
F	1	3
B	1	1

Formula Calculator Results

Ion Formula	m/z	m/z (Calc)	DBE	Diff (ppm)	Score (MFG)
C14 H15 B F2 N O2	277.1194	277.1195	9	0.28	99.98
C17 H10 B F Na O	282.0743	282.0737	13	-2.21	98.7
C14 H11 B F2 Na O2	282.0743	282.0749	9	2.2	98.72

--- End Of Report ---

Agilent Technologies Page 1 of 1 Printed at: 5:00 PM on: 10/18/2023

Supplementary Fig. 137. HRMS spectra of compound **NAP1BF$_2$**.

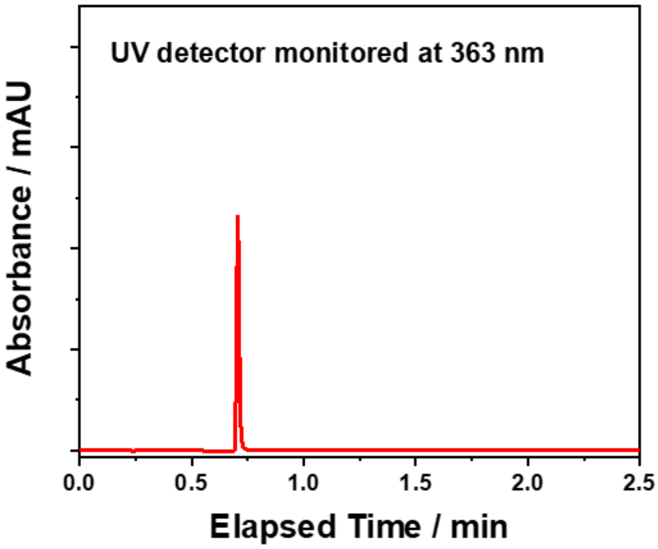

Supplementary Fig. 138. HPLC of compound **NAP1BF$_2$**.

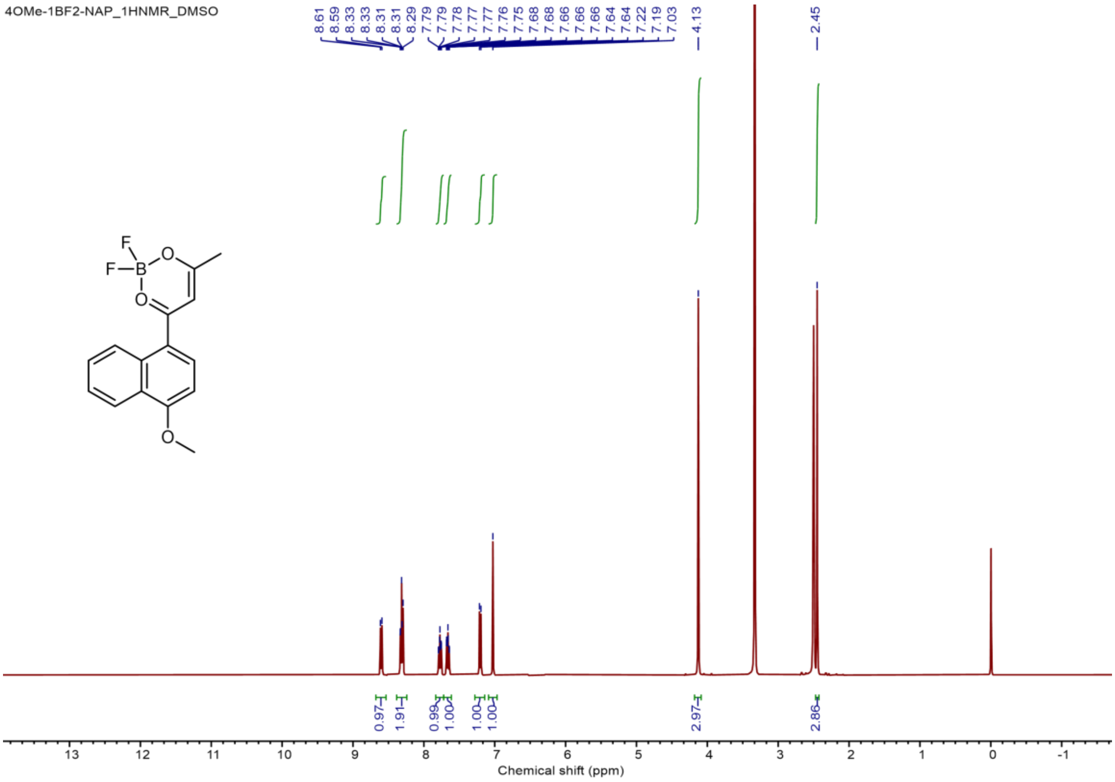

Supplementary Fig. 139. ^{1}H NMR spectra (400 MHz, DMSO-d_6) of compound **4OMeNAPBF$_2$**.

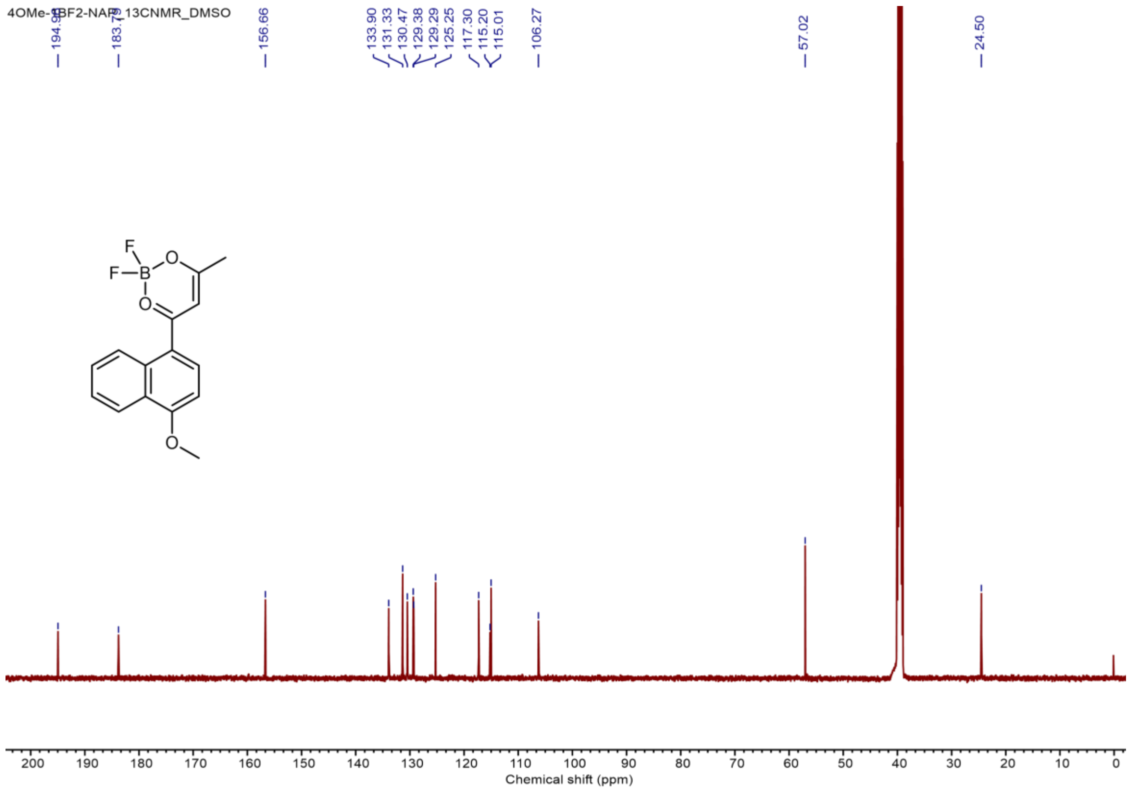

Supplementary Fig. 140. ^{13}C NMR spectra (126 MHz, DMSO-d_6) of compound **4OMeNAPBF$_2$**.

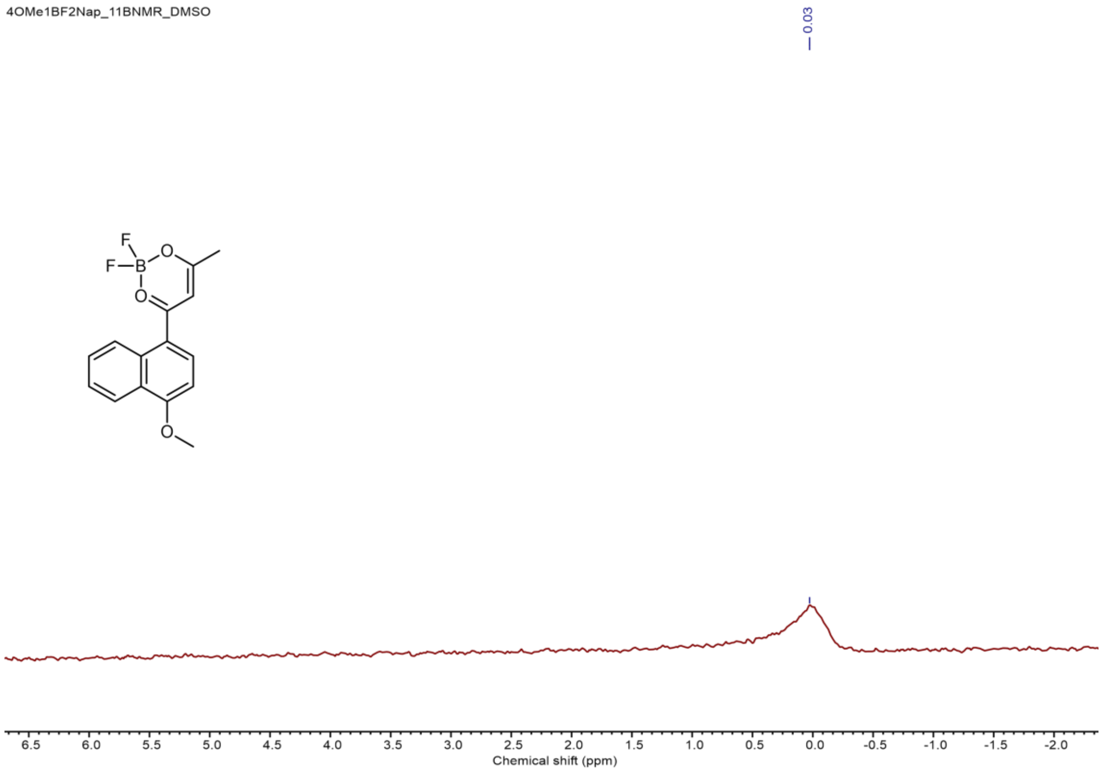

Supplementary Fig. 141. ^{11}B NMR spectra (128 MHz, DMSO-d_6) of compound **4OMeNAPBF$_2$**.

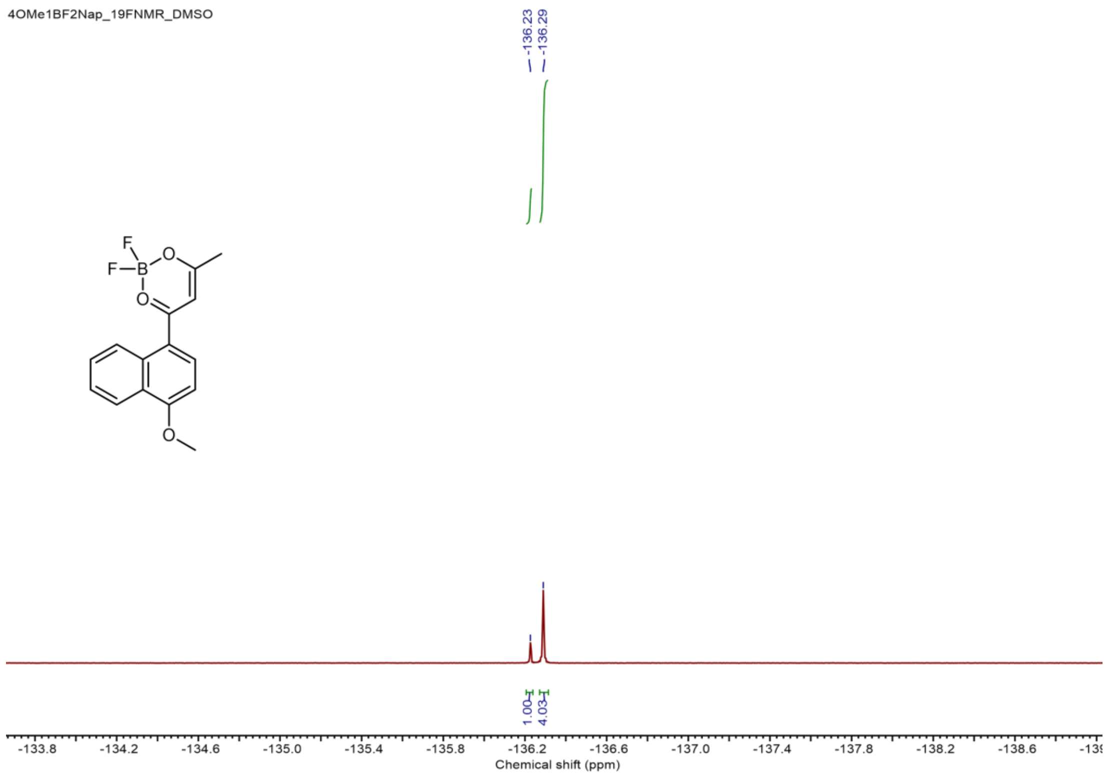

Supplementary Fig. 142. ^{19}F NMR spectra (376 MHz, DMSO-d_6) of compound **4OMeNAPBF$_2$**.

Qualitative Analysis Report

**Data Filename**	2019115-LX-5.d	**Sample Name**	LX-5
**Sample Type**	Sample	**Position**	Vial 65
**Instrument Name**	Instrument 1	**User Name**	
**Acq Method**	IDJ4-75V.m	**Acquired Time**	10/18/2023 3:20:55 PM
**IRM Calibration Status**	Success	**DA Method**	FGFUS-C18.m
**Comment**			

Sample Group **Info.**
Acquisition SW 6200 series TOF/6500 series
Version Q-TOF B.05.01 (B5125.3)

User Spectra

Fragmentor Voltage	Collision Energy	Ionization Mode
175	0	ESI

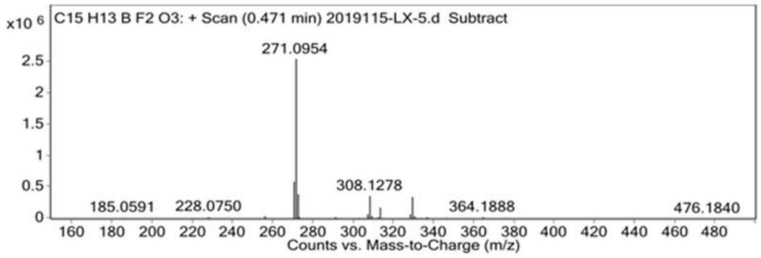

Peak List

m/z	z	Abund	Formula	Ion
307.1301	1	72035.45	C15 H13 B F2 O3	(M+NH4)+
308.1278	1	365860.59	C15 H13 B F2 O3	(M+NH4)+
309.1297	1	49344.18	C15 H13 B F2 O3	(M+NH4)+
310.1318	1	4308.16	C15 H13 B F2 O3	(M+NH4)+
312.0850	1	33940.36	C15 H13 B F2 O3	(M+Na)+
313.0828	1	184540.42	C15 H13 B F2 O3	(M+Na)+
314.0848	1	21742.59	C15 H13 B F2 O3	(M+Na)+

Formula Calculator Element Limits

Element	Min	Max
C	3	70
H	0	120
O	1	6
F	1	3
B	1	1

Formula Calculator Results

Ion Formula	m/z	m/z (Calc)	DBE	Diff (ppm)	Score (MFG)
C15 H17 B F2 N O3	307.1301	307.1300	9	-0.21	99.99
C12 H18 B F3 N O4	307.1301	307.1312	5	3.74	95.93
C18 H12 B F Na O2	312.0850	312.0843	13	-2.45	98.21
C15 H13 B F2 Na O3	312.0850	312.0854	9	1.5	99.32

--- End Of Report ---

Agilent Technologies Page 1 of 1 Printed at: 4:53 PM on: 10/18/2023

Supplementary Fig. 143. HRMS spectra of compound **4OMeNAPBF$_2$**.

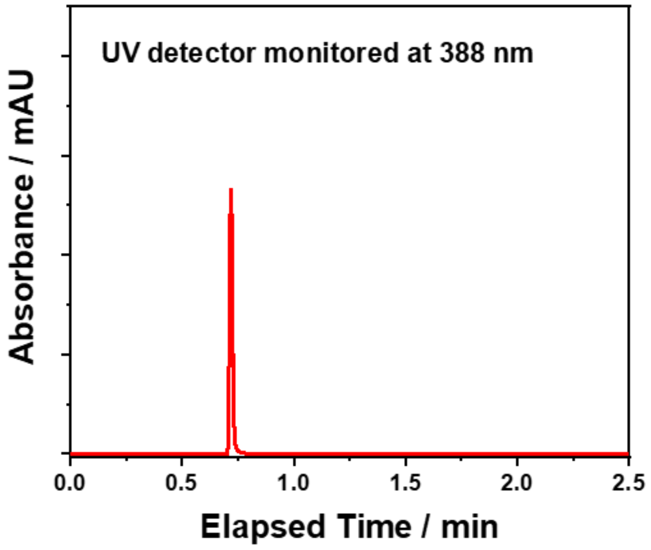

Supplementary Fig. 144. HPLC of compound **4OMeNAPBF$_2$**.

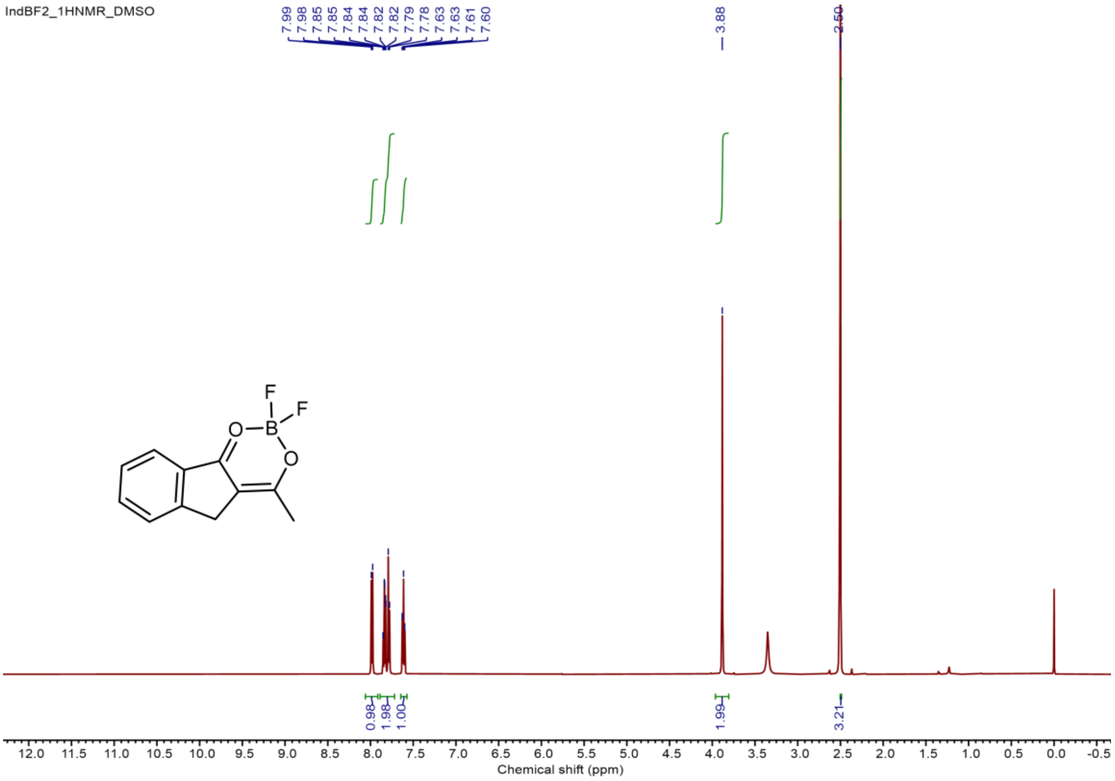

Supplementary Fig. 145. ^{1}H NMR spectra (500 MHz, DMSO-d_6) of compound **IndBF$_2$**.

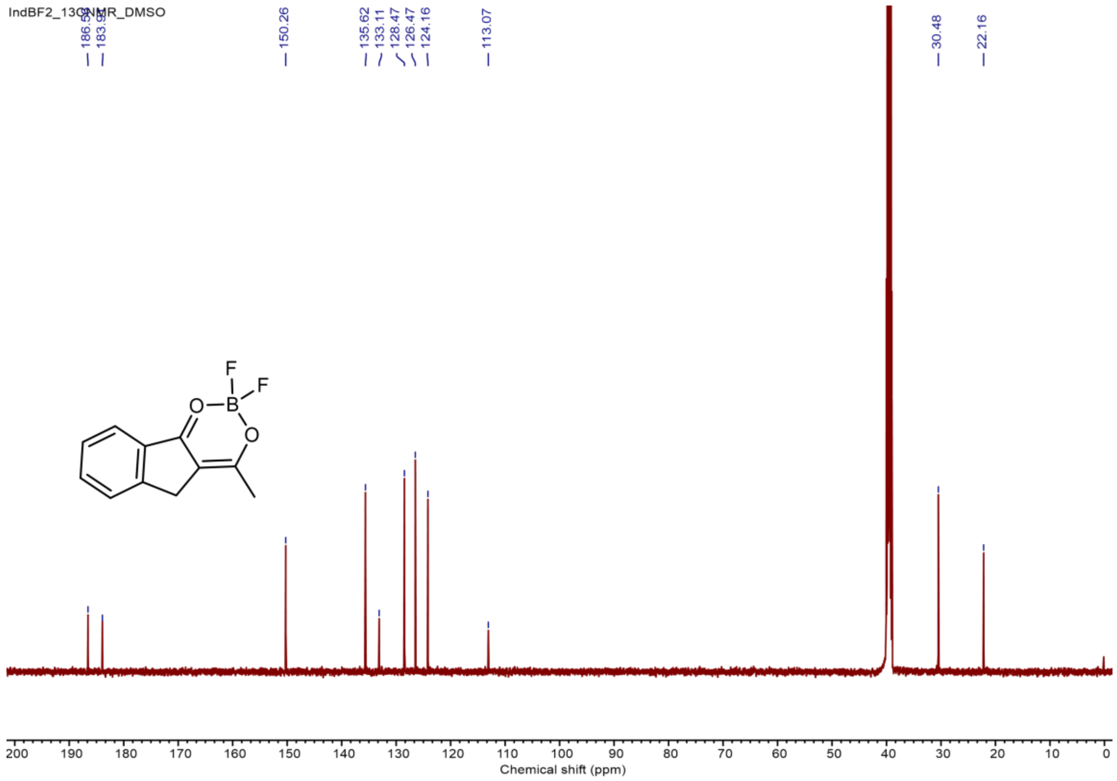

Supplementary Fig. 146. ^{13}C NMR spectra (126 MHz, DMSO-d_6) of compound **IndBF$_2$**.

Qualitative Analysis Report

**Data Filename**	2019115-LX-3.d	**Sample Name**	LX-3
**Sample Type**	Sample	**Position**	Vial 63
**Instrument Name**	Instrument 1	**User Name**	
**Acq Method**	IDJ4-75V.m	**Acquired Time**	10/18/2023 3:10:02 PM
**IRM Calibration Status**	Success	**DA Method**	FGFUS-C18.m
**Comment**			

**Sample Group**		**Info.**
**Acquisition SW**	6200 series TOF/6500 series	
**Version**	Q-TOF B.05.01 (B5125.3)	

User Spectra

Fragmentor Voltage	Collision Energy	Ionization Mode
175	0	ESI

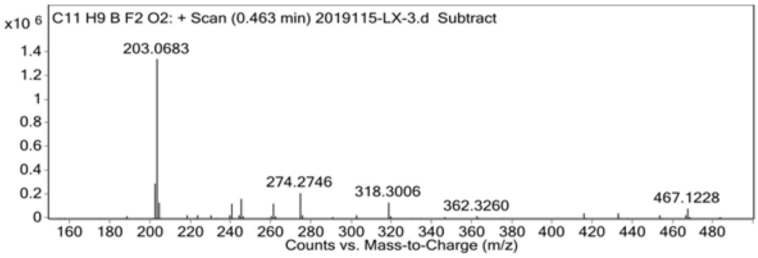

Peak List

m/z	z	Abund	Formula	Ion
239.1043	1	33484.28	C11 H9 B F2 O2	(M+NH4)+
240.1008	1	127869.13	C11 H9 B F2 O2	(M+NH4)+
241.1030	1	11755.55	C11 H9 B F2 O2	(M+NH4)+
244.0591	1	31396.69	C11 H9 B F2 O2	(M+Na)+
245.0562	1	170719.36	C11 H9 B F2 O2	(M+Na)+
246.0587	1	14955.91	C11 H9 B F2 O2	(M+Na)+

Formula Calculator Element Limits

Element	Min	Max
C	3	70
H	0	120
O	1	6
F	1	3
B	1	1

Formula Calculator Results

Ion Formula	m/z	m/z (Calc)	DBE	Diff (ppm)	Score (MFG)
C11 H13 B F2 N O2	239.1043	239.1038	7	-2.16	98.98
C11 H9 B F2 Na O2	244.0591	244.0592	7	0.54	99.94

--- End Of Report ---

Supplementary Fig. 147. HRMS spectra of compound **IndBF$_2$**.

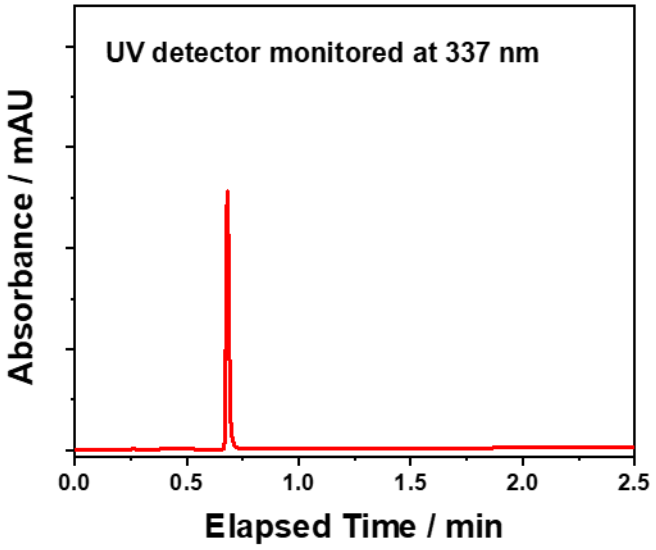

Supplementary Fig. 148. HLPC of compound **IndBF$_2$**.

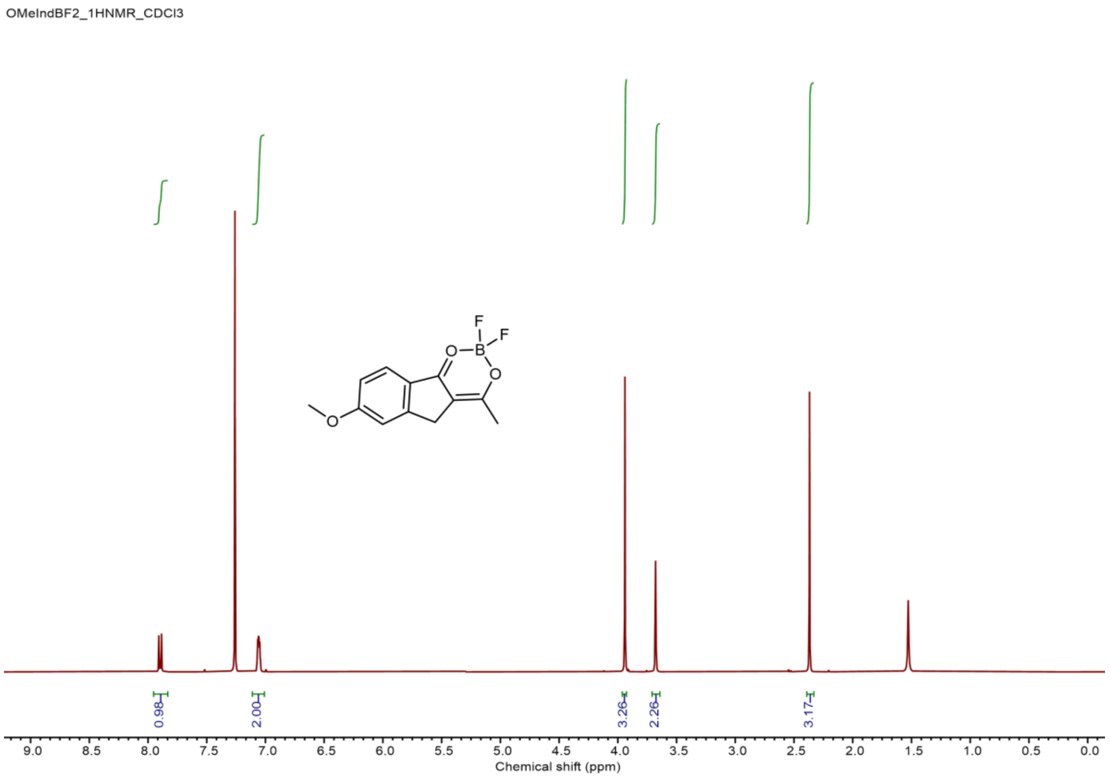

Supplementary Fig. 149. ^{1}H NMR spectra (400 MHz, Chloroform-*d*) of compound **OMeIndBF$_2$**.

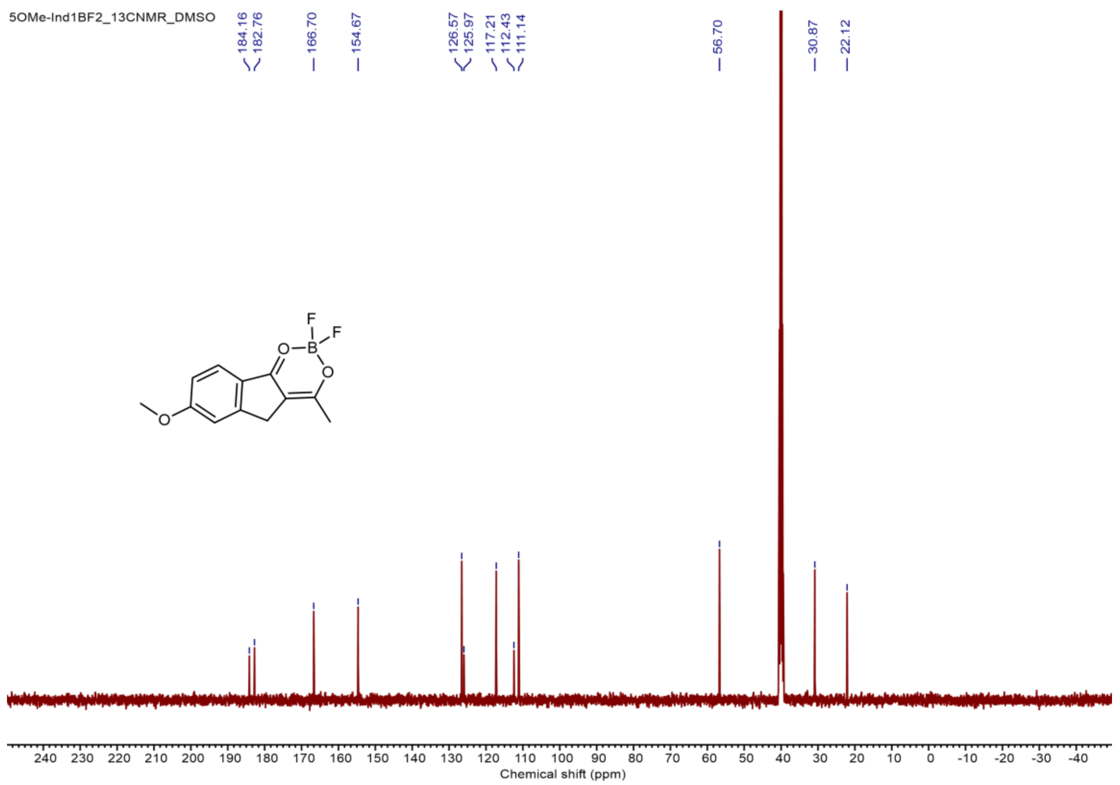

Supplementary Fig. 150. ^{13}C NMR spectra (101 MHz, DMSO-d_6) of compound **OMeIndBF$_2$**.

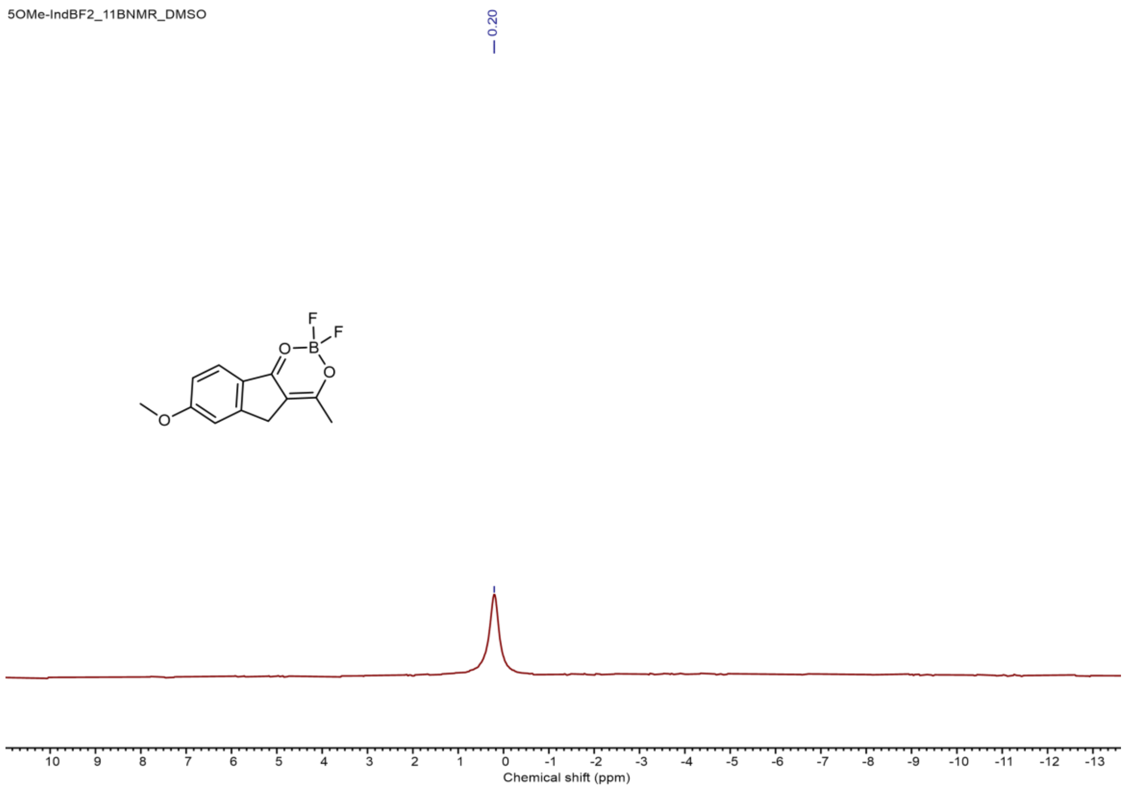

Supplementary Fig. 151. ^{11}B NMR spectra (128 MHz, DMSO-d_6) of compound **OMeIndBF$_2$**.

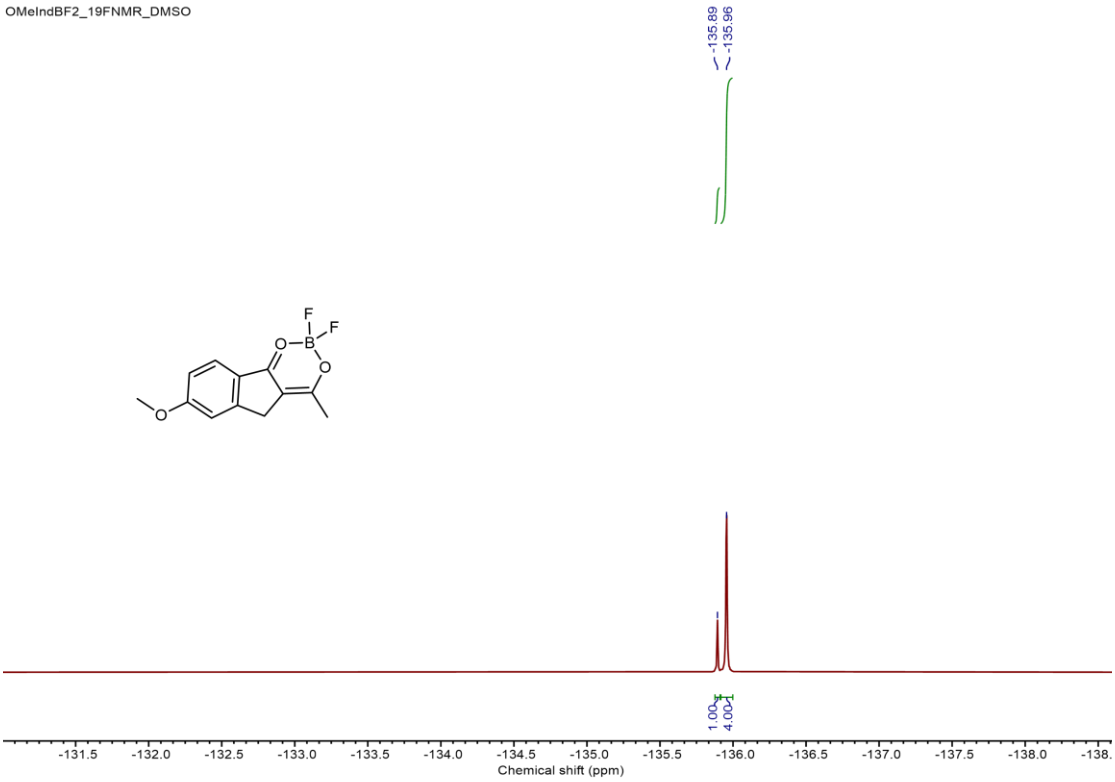

Supplementary Fig. 152. ^{19}F NMR spectra (376 MHz, DMSO-d_6) of compound **OMeIndBF$_2$**.

Qualitative Analysis Report

**Data Filename**	2019115-LX-2.d	**Sample Name**	LX-2
**Sample Type**	Sample	**Position**	Vial 62
**Instrument Name**	Instrument 1	**User Name**	
**Acq Method**	IDJ4-75V.m	**Acquired Time**	10/18/2023 3:53:48 PM
**IRM Calibration Status**	Success	**DA Method**	FGFUS-C18.m
**Comment**			

Sample Group **Info.**
Acquisition SW 6200 series TOF/6500 series
Version Q-TOF B.05.01 (B5125.3)

User Spectra

Fragmentor Voltage	Collision Energy	Ionization Mode
175	0	ESI

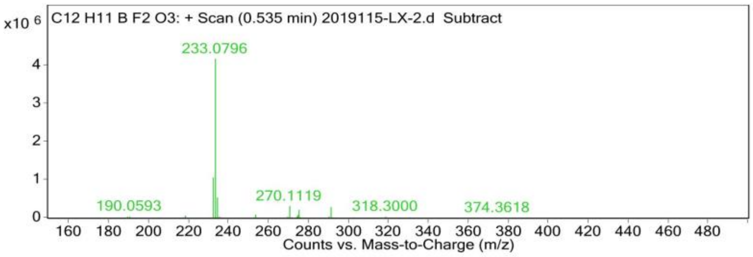

Peak List

m/z	z	Abund	Formula	Ion
269.1144	1	61502.86	C12 H11 B F2 O3	(M+NH4)+
270.1119	1	312964.66	C12 H11 B F2 O3	(M+NH4)+
271.1131	1	31543.61	C12 H11 B F2 O3	(M+NH4)+
274.0699	1	42596.86	C12 H11 B F2 O3	(M+Na)+
275.0671	1	225286.47	C12 H11 B F2 O3	(M+Na)+
276.0693	1	21998.09	C12 H11 B F2 O3	(M+Na)+

Formula Calculator Element Limits

Element	Min	Max
C	3	70
H	0	120
O	1	5
F	1	3
B	1	1

Formula Calculator Results

Ion Formula	m/z	m/z (Calc)	DBE	Diff (ppm)	Score (MFG)
C12 H15 B F2 N O3	269.1144	269.1144	7	-0.05	100
C12 H11 B F2 Na O3	274.0699	274.0698	7	-0.46	99.94

--- End Of Report ---

Agilent Technologies   Page 1 of 1   Printed at: 4:44 PM on: 10/18/2023

Supplementary Fig. 153. HRMS spectra of compound **OMeIndBF$_2$.**

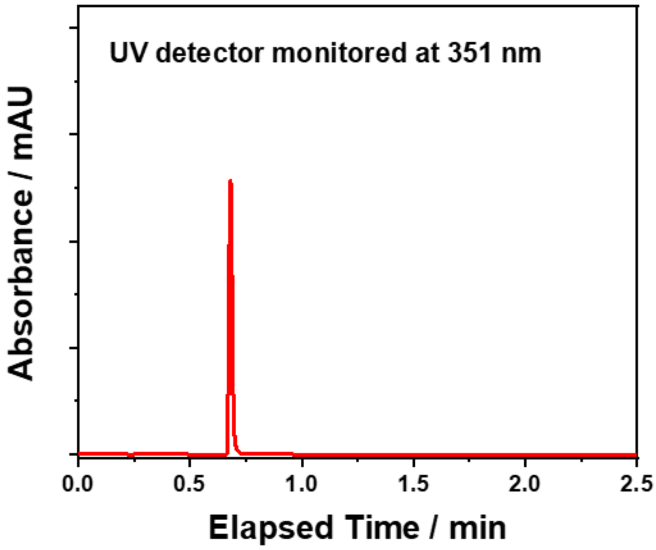

Supplementary Fig. 154. HPLC of compound **OMeIndBF$_2$.**

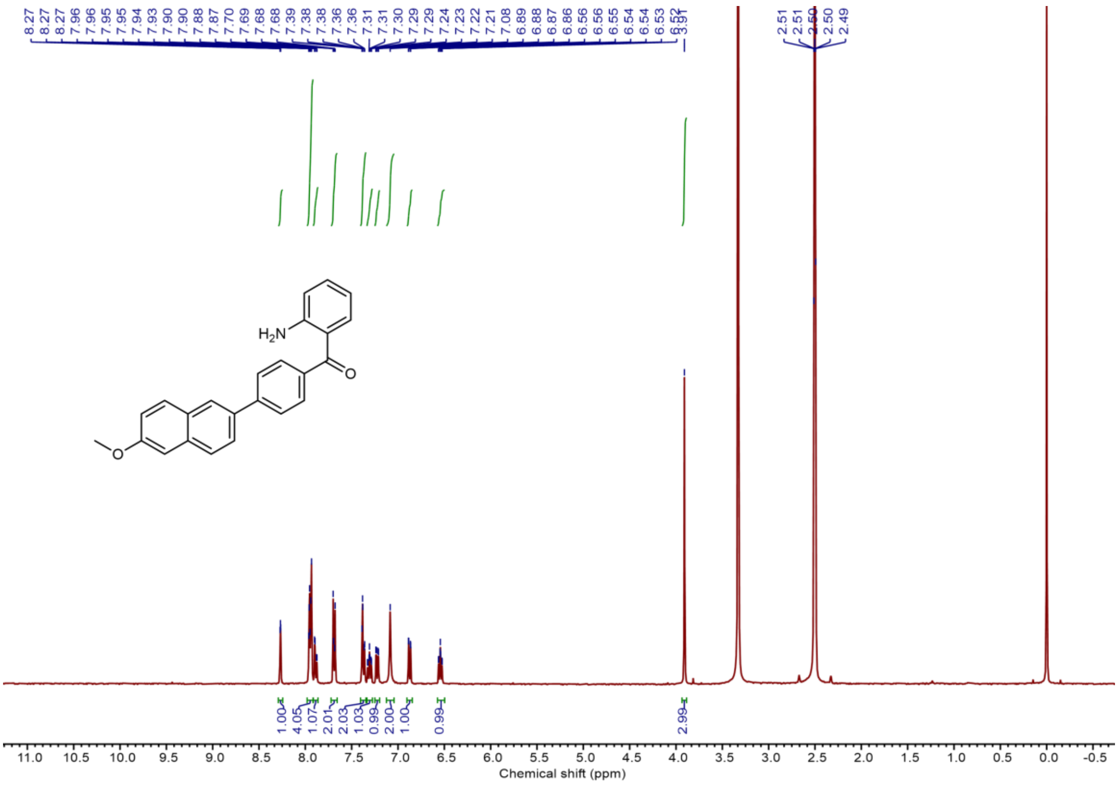

Supplementary Fig. 155. ^{1}H NMR spectra (400 MHz, DMSO-d_6) of compound **NAPpBP-NH$_2$**.

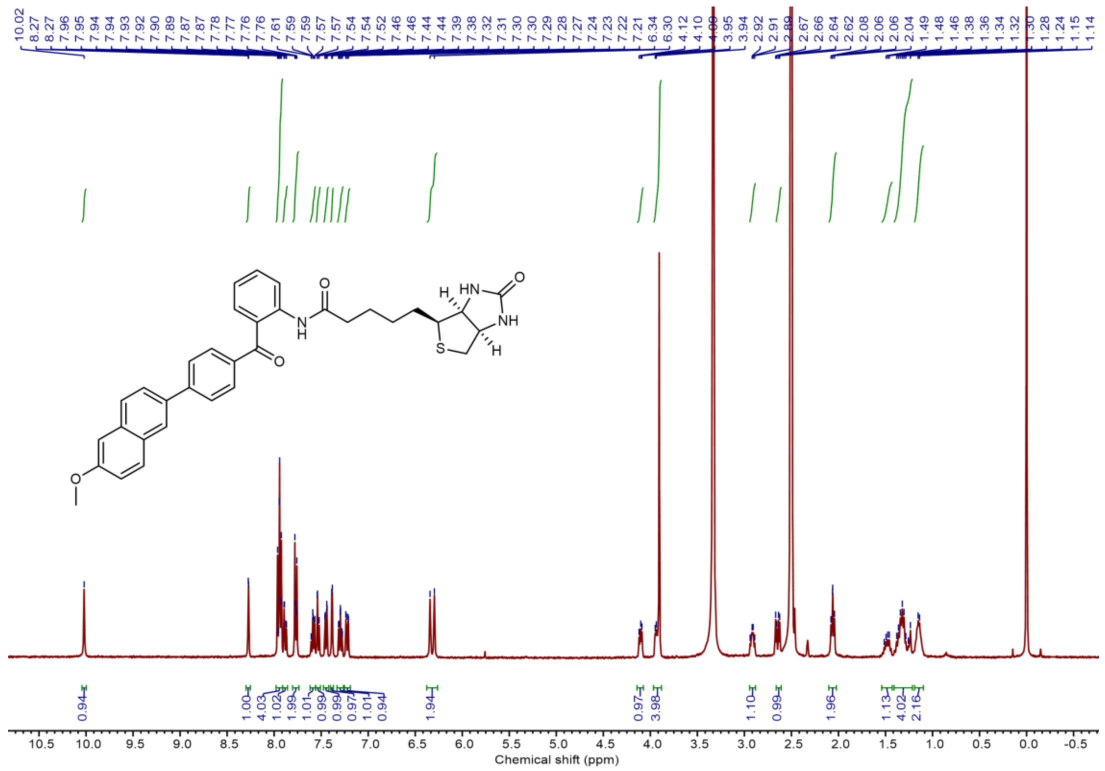

Supplementary Fig. 156. ^{1}H NMR spectra (400 MHz, DMSO-d_6) of compound **NAPpBP-Biotin**.

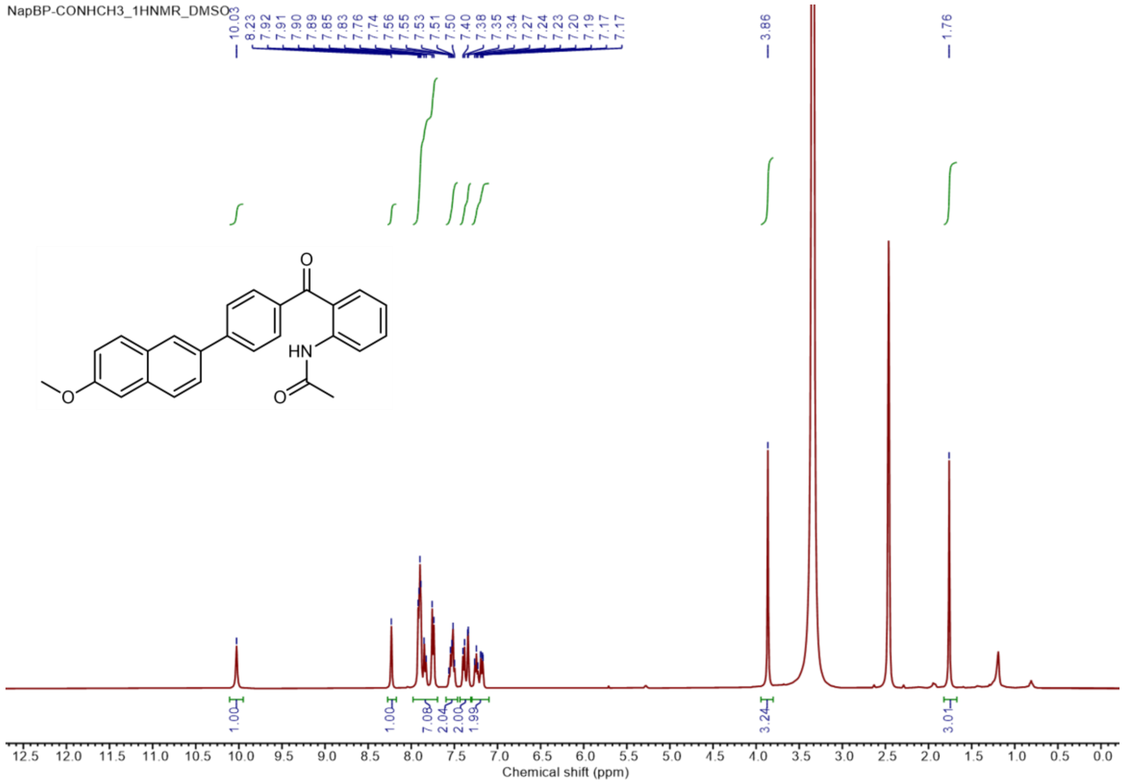

Supplementary Fig. 157. ^{1}H NMR spectra (400 MHz, DMSO-d_6) of compound **NAPpBP-NHCOCH3**.

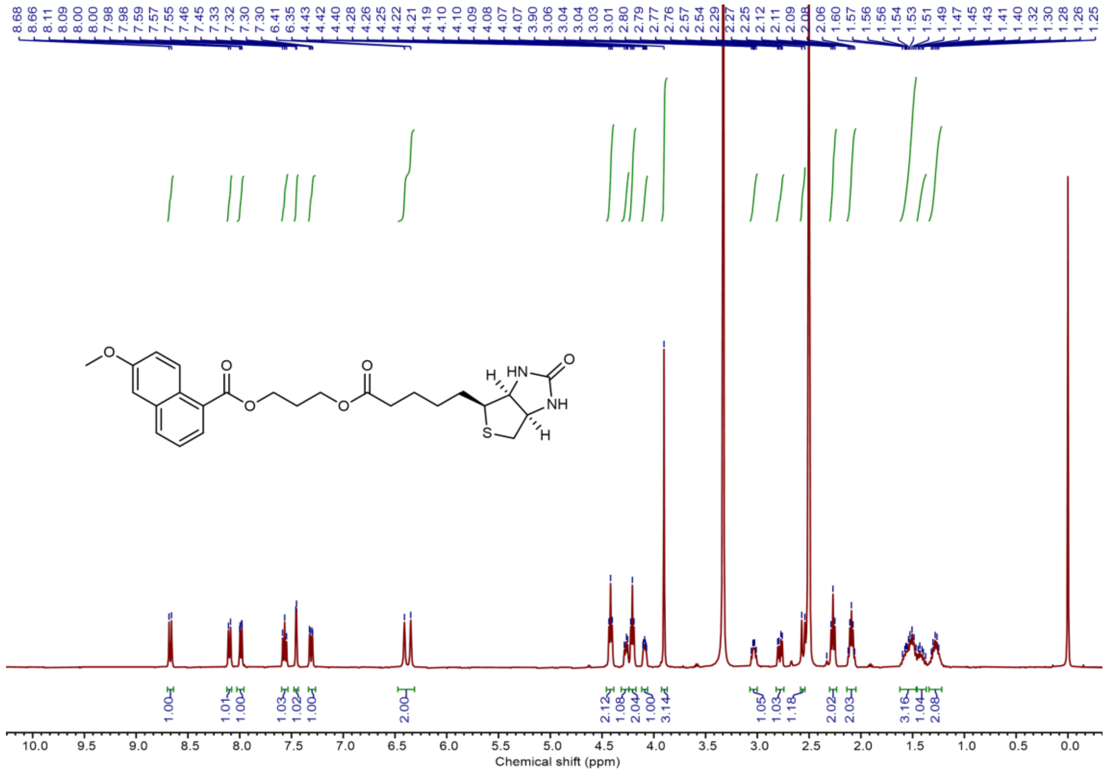

Supplementary Fig. 158. ^{1}H NMR spectra (400 MHz, DMSO-d_6) of compound **1NAP-Biotin**.

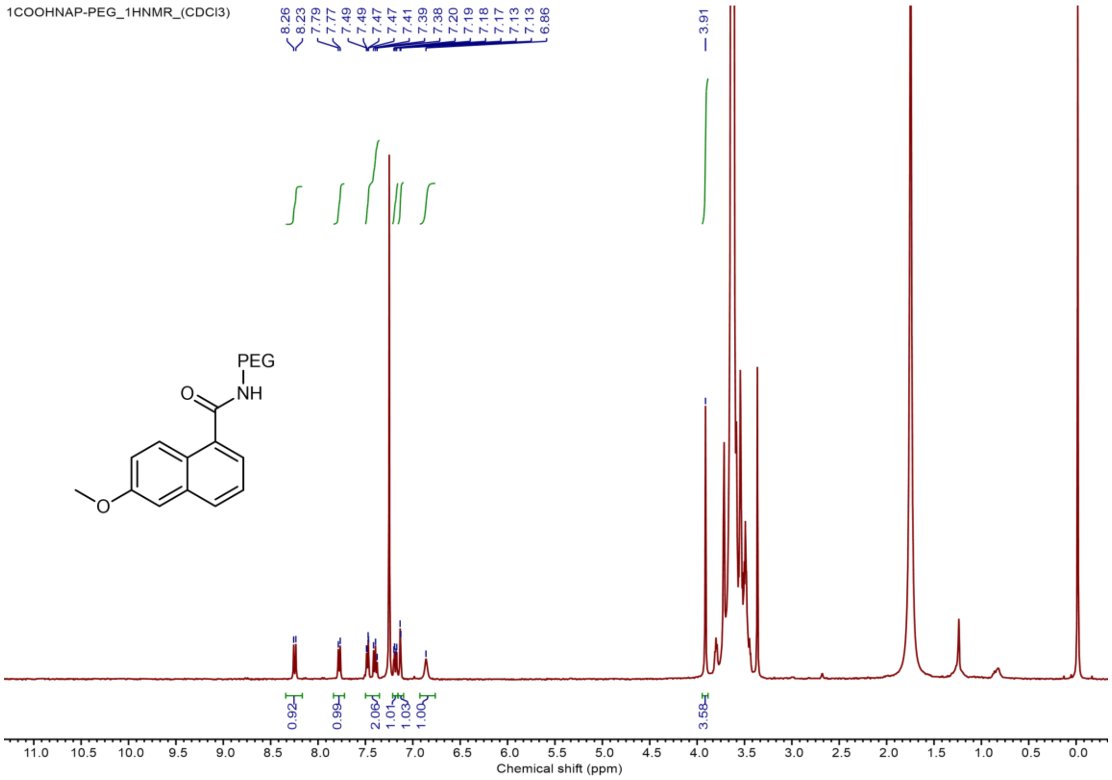

Supplementary Fig. 159. ^{1}H NMR spectra (400 MHz, Chloroform-d) of compound **1NAP-PEG**.

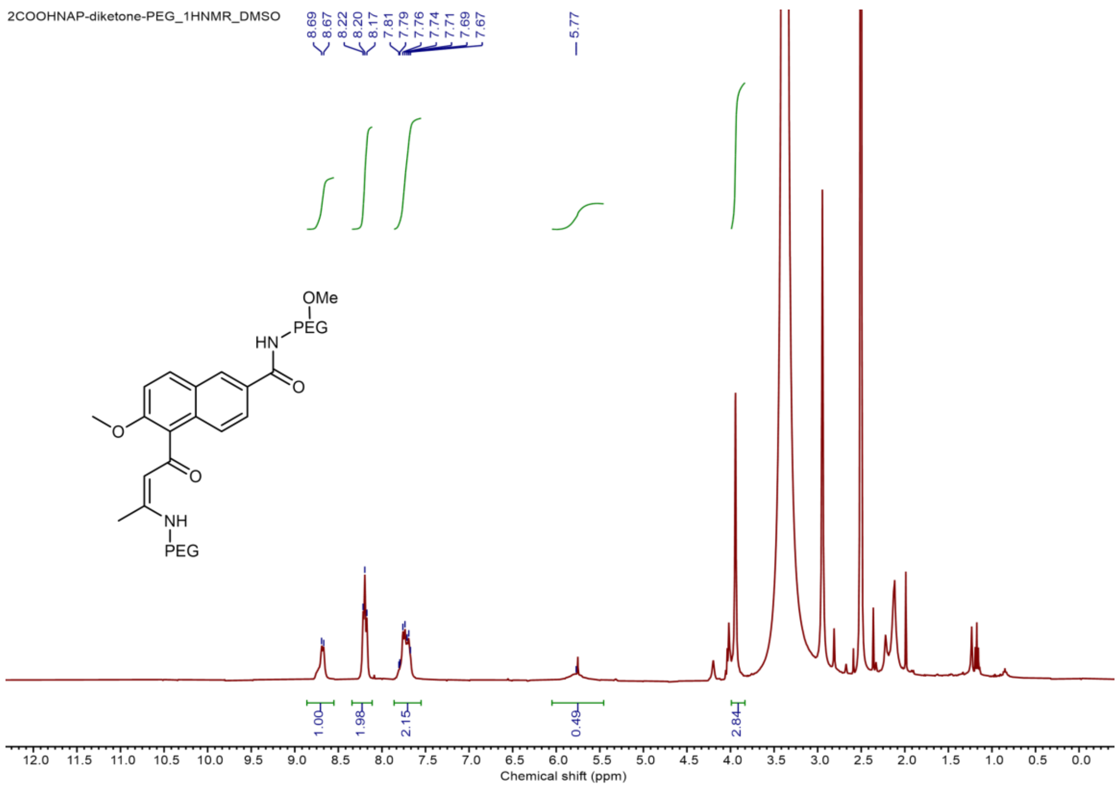

Supplementary Fig. 160. ^{1}H NMR spectra (400 MHz, DMSO-d_6) of compound **2COOHNAP-diketone-PEG**.

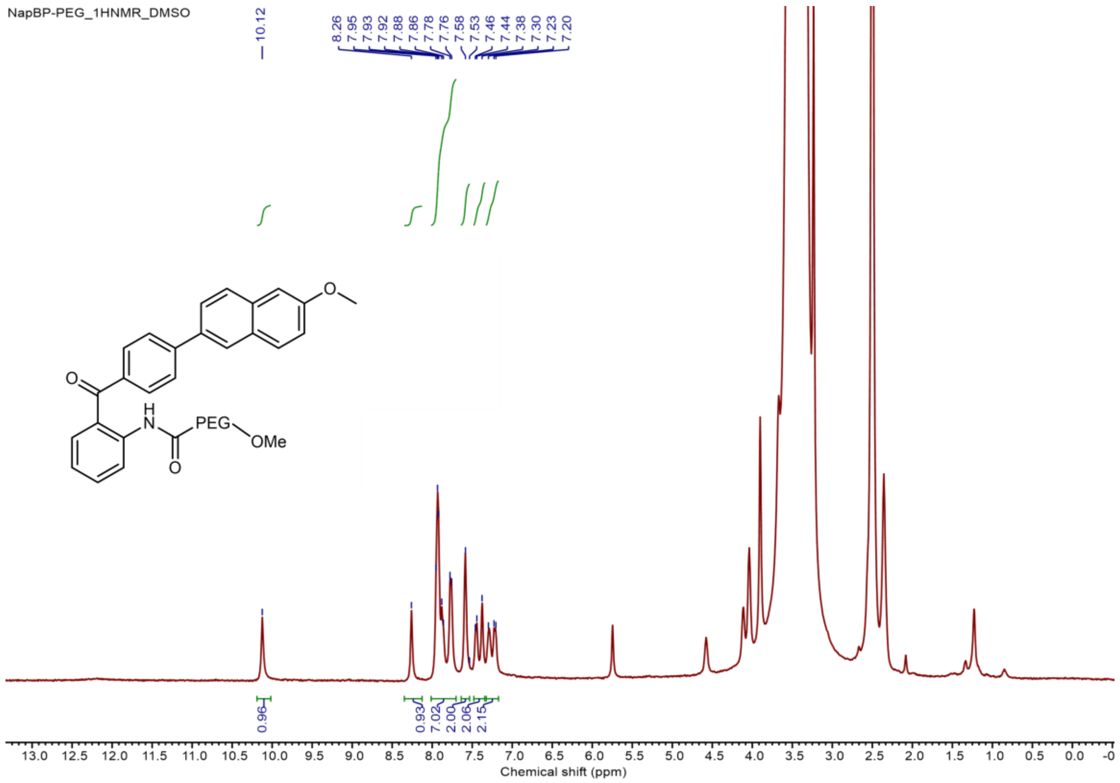

Supplementary Fig. 161. ^{1}H NMR spectra (400 MHz, DMSO-d_6) of compound **NAPpBP-PEG**.

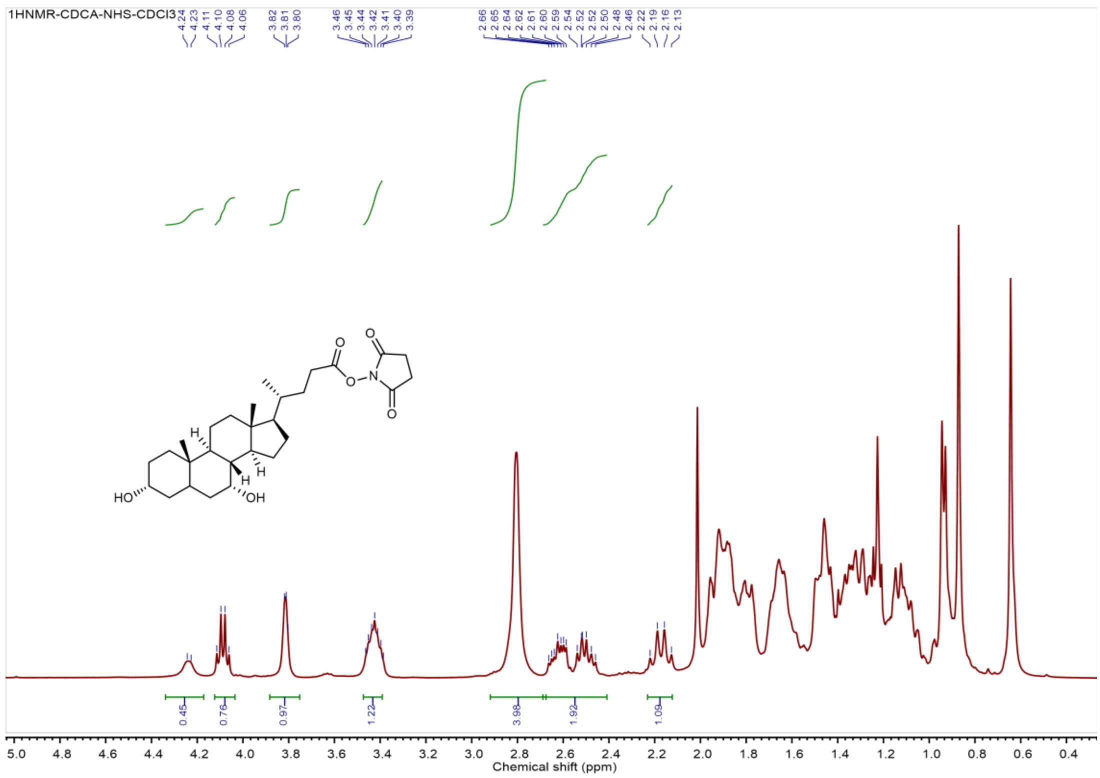

Supplementary Fig. 162. ^{1}H NMR spectra (400 MHz, Chloroform-*d*) of compound **CDCA-NHS.**

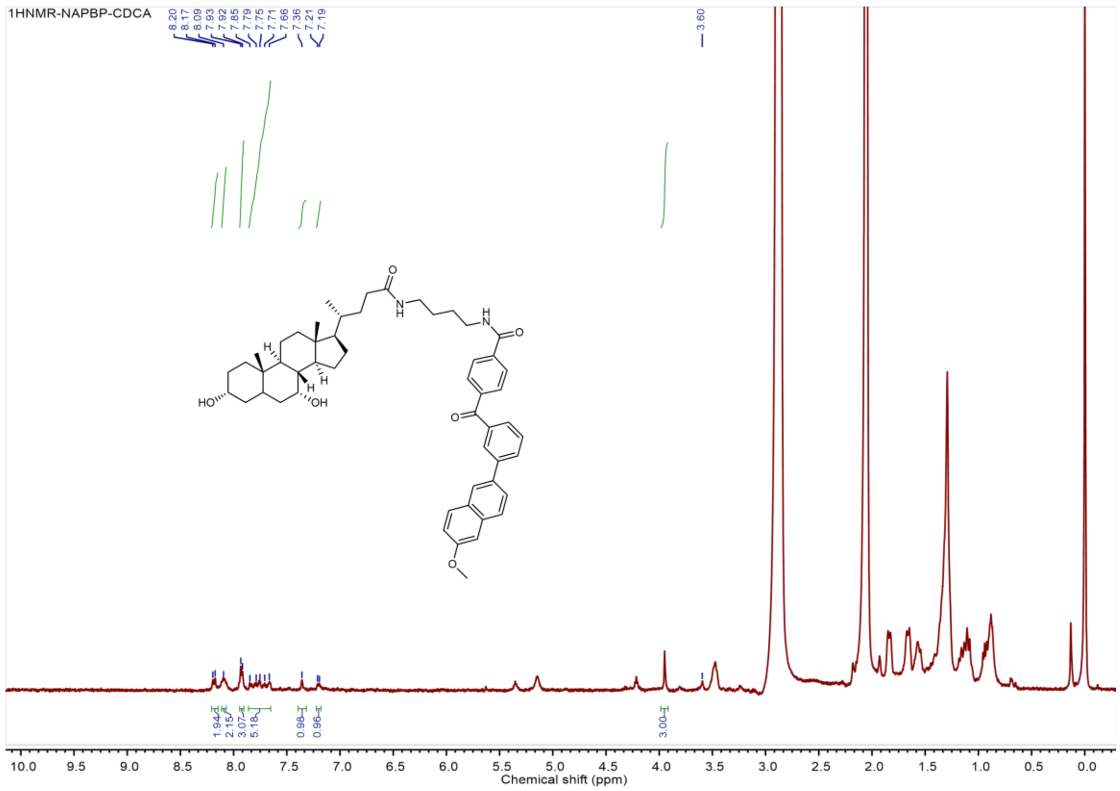

Supplementary Fig. 163. ^{1}H NMR spectra (500 MHz, Acetone-d_5) of compound **NAPBP-CDCA.**

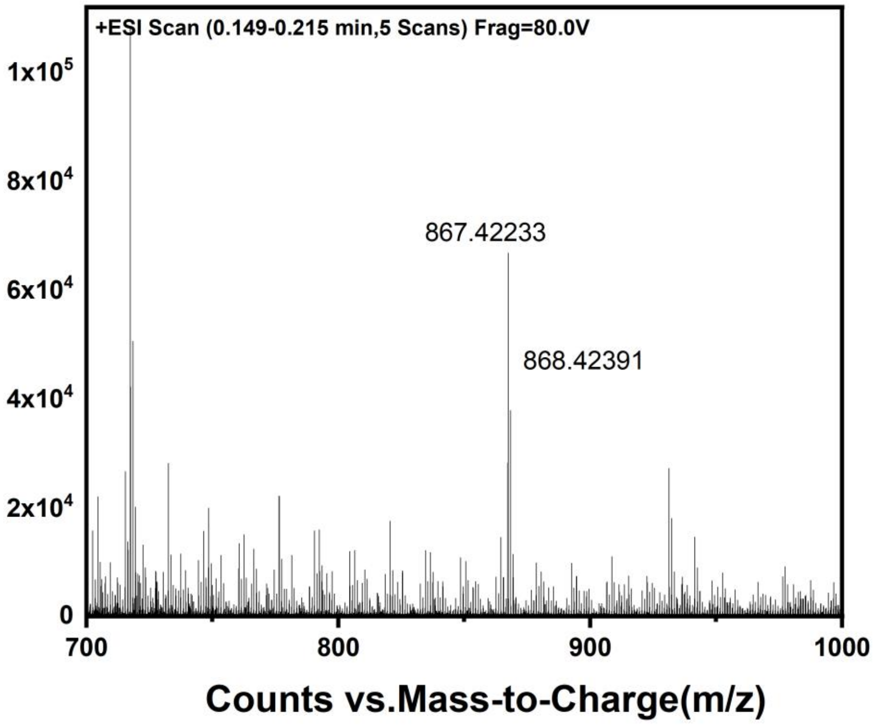

Supplementary Fig. 164. HRMS spectra of compound **NAPBP-CDCA.**

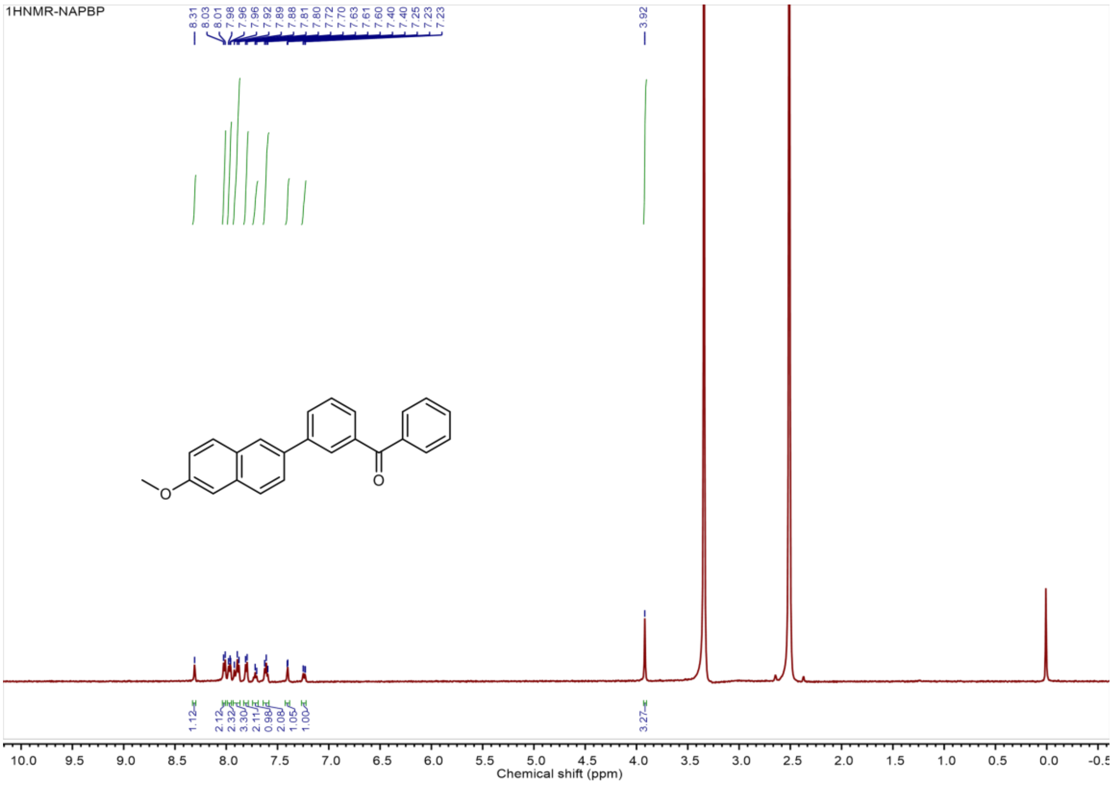

Supplementary Fig. 165. ^{1}H NMR spectra (500 MHz, DMSO-d_6) of compound **NAPBP.**

Materials

6-Methoxy-2-naphthoic acid (97%, Bidepharm), 6-methoxy-1-naphthoic acid (95%, Bidepharm), 2-methoxynaphthalene (97%, Bidepharm), 4’-methoxy-[1,1’-biphenyl]-4-carboxylic acid (RG, Adamas), N-hydroxysuccinimide (98%, Shanghai Dibo Chemicals Technology Co., Ltd.), 3-(3-dimethylaminopropyl)-1-ethylcarbodiimide hydrochloride (RG, Adamas), potassium permanganate (AG, Sinopharm Chemical Reagent), fluorene-9-carboxylic acid (RG, Adamas), 9H-fluorene-1-carboxylic acid (98%, Bidepharm), 2-(9-oxoacridin-10(9H)-yl) acetic acid (99%, Bidepharm), 1-pyrenecarboxylic acid (97%, Bidepharm), 3-bromobenzoylchloride (98%, Adamas), 6-methoxy-2-naphthaleneboronic acid (98%, Adamas), (4'-methoxy-[1,1'-biphenyl]-4-yl)boronic acid (98%, Bidepharm), (4-benzoylphenyl)boronic acid (98%, Bidepharm), 4-(diphenylamino)phenylboronic acid (98%, Bidepharm), 1-pyreneboronic acid (RG, Adamas), 9,9’-dimethylfluoren-2-boronic acid (97%, Shanghai Dibo Chemicals Technology Co.,Ltd.), 9,9’-spirobifluorene-2-boronic acid (98%, Bidepharm), 2-(6-methoxynaphthalen-2-yl)propanoic acid (98%, Bidepharm), 2,6-dimethoxynaphthalene (98%, Adamas), 6-methoxy-2-bromonaphthalene (98%, Shanghai Dibo Chemicals Technology Co.,Ltd.), triphenylamine (98%, Adamas), (4-bromophenyl) diphenylamine (98%, Bidepharm), 1-indanone (98%, Adamas), 5-methoxy-1-indanone (98%, Shanghai Saen Chemicals Technology Co.,Ltd.), 1-(4-methoxynaphthalen-1-yl) ethenone (97%, Bidepharm), 1-acetonaphthone (97%, Innochem), 2-acetonaphthone (99%, Innochem), 6-methoxy-2-acetonaphthone (98%, Innochem), coronene (97%, Aladdin), pyrene (RG, Bidepharm), 9,9'-spirobifluorene (98%, Bidepharm), 9,9’-dimethylfluorene, (99%, Innochem), 4-methoxybiphenyl (95%, Bidepharm), palladium(II) acetate (99%, Adamas), potassium carbonate (99%, Shanghai Dahe Chemicals Co., Ltd.), acetic anhydride (98.5%, Sinopharm Chemical Reagent), boron trifluoride diethyl etherate (98%, TCI), benzoic acid (99%, Bidepharm), H_2N-PEG-OMe (M_n = 2000), HOOC-PEG-OMe (M_n = 2000), PEG-NHS (M_n = 2000, with MeO- end group) and PEG-NHS (M_n = 5000, with MeO-

end group) (Chongqing Yusi Pharmaceutical Technology Co., Ltd.), polystyrene microsphere (5 μm, carboxylated, Zhichuan Intelligent Technology (Suzhou) Co., Ltd.), chenodeoxycholic acid (RG, Adamas), *N*,*N*-dicyclohexylcarbodiimide (99%, Adamas), 3-bromobenzophenone (97%, Adamas), 1,4-diaminobutane (98%, Innochem), lipase from pocine pancreas (SHIMIE Co., Ltd.), *ε*-polylysine (EPL) (≥95%, Aladdin).

Molecular synthesis

Synthesis of compound 1NAP-NHS

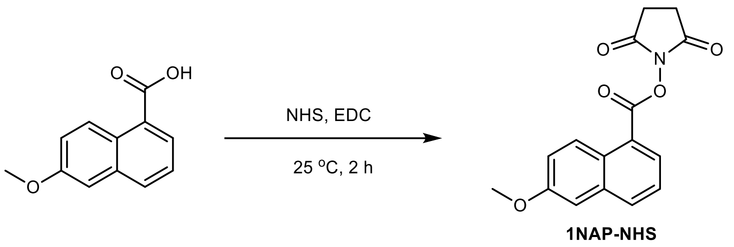

To a round-bottom flask, 6-methoxy-1-naphthoic acid (202.0 mg, 1.0 mmol), N-hydroxysuccinimide (345.0 mg, 3.0 mmol), 3-(3-dimethylaminopropyl)-1-ethylcarbodiimide hydrochloride (479.0 mg, 2.5 mmol), and dichloromethane (3.0 mL) were added. The reaction mixture was stirred at 25 ℃ for 2 hours. Upon completion, the reaction was purified via silica gel column chromatography, utilizing dichloromethane/ethyl acetate (50:1) as the eluent, which resulted in the formation of a white solid, **1NAP-NHS** (226.0 mg), with an isolated yield of 75.6%. ^{1}H NMR (400 MHz, DMSO-d_6) δ 8.59 (d, J = 9.5 Hz, 1H), 8.31 – 8.19 (m, 2H), 7.69 (t, J = 7.8 Hz, 1H), 7.54 (t, J = 2.4 Hz, 1H), 7.43 (dd, J = 9.4, 2.7 Hz, 1H), 3.94 (s, 3H), 2.99 (s, 4H). ^{13}C NMR (126 MHz, DMSO-d_6) δ 170.58, 162.50, 157.65, 135.22, 134.54, 128.86, 125.86, 125.67, 125.58, 121.40, 121.12, 107.30, 55.37, 25.68. HRMS m/z found (calcd for $C_{16}H_{13}NO_5Na^+$): 322.06842 (322.0691).

Synthesis of compound Acridone-NHS

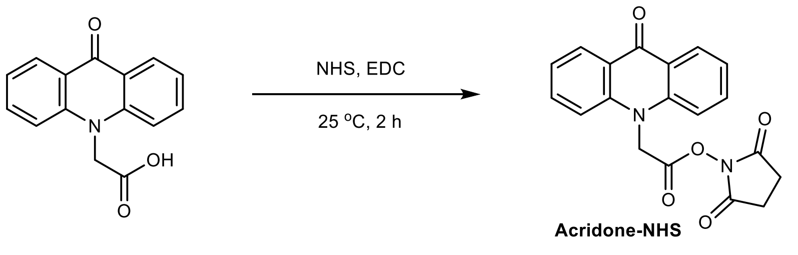

To a round-bottom flask, 2-(9-oxoacridin-10(9H)-yl) acetic acid (253.0 mg, 1.0 mmol), N-hydroxysuccinimide (345.0 mg, 3.0 mmol), 3-(3-dimethylaminopropyl)-1-ethylcarbodiimide hydrochloride (479.0 mg, 2.5 mmol), and dichloromethane (2.0 mL) were added. The reaction mixture was stirred at 25 ℃ for 2 hours. Upon completion,

the reaction was purified via silica gel column chromatography, utilizing dichloromethane/ethyl acetate (50:1) as the eluent, resulting in the formation of a white compound, **Acridone-NHS**. The compound was further purified by recrystallization using spectroscopic-grade dichloromethane/hexane. ^{1}H NMR (400 MHz, DMSO-*d6*) δ 8.36 (dd, J = 7.9, 1.7 Hz, 2H), 7.90–7.82 (m, 2H), 7.75 (d, J = 8.7 Hz, 2H), 7.40 (t, J = 7.5 Hz, 2H), 6.02 (s, 2H), 2.81 (s, 4H).

Synthesis of compound Pyrene-NHS

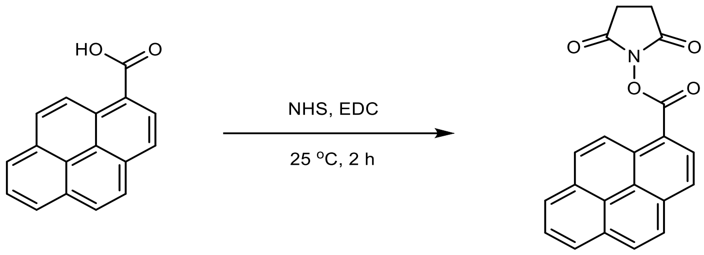

Pyrene-NHS

To a round-bottom flask, 1-pyrenecarboxylic acid (123.0 mg, 0.5 mmol), N-hydroxysuccinimide (173.0 mg, 1.5 mmol), 3-(3-dimethylaminopropyl)-1-ethylcarbodiimide hydrochloride (239.0 mg, 1.25 mmol), and dichloromethane (2.0 mL) were added. The reaction mixture was stirred at 25 ℃ for 2 hours. Upon completion, the reaction was purified via silica gel column chromatography, utilizing dichloromethane/ethyl acetate (50:1) as the eluent, resulting in the formation of a yellow compound, **Pyrene-NHS** (123.1 mg), with an isolated yield of 71.8%. ^{1}H NMR (500 MHz, DMSO-d_6) δ 9.00 (d, *J* = 9.5 Hz, 1H), 8.76 (d, *J* = 8.2 Hz, 1H), 8.56 – 8.46 (m, 5H), 8.36 (d, *J* = 9.0 Hz, 1H), 8.25 (t, *J* = 7.6 Hz, 1H), 3.00 (s, 4H).

Synthesis of compound 2NAP-NHS

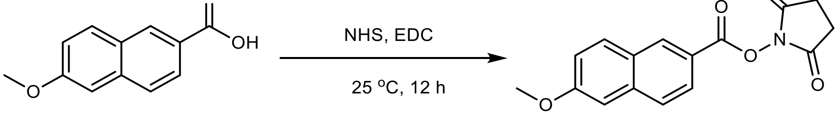

2NAP-NHS

To a round-bottom flask, 6-methoxy-2-naphthoic acid (202.0 mg, 1.0 mmol), N-hydroxysuccinimide (150.0 mg, 1.3 mmol), 3-(3-dimethylaminopropyl)-1-ethylcarbodiimide hydrochloride (249.0 mg, 1.3 mmol), and *N,N*-dimethylformamide

(1.0 mL) were added. The reaction mixture was stirred at 25 ℃ for 12 hours. Upon completion, the solvent was removed by rotary evaporation, yielding an oily solution. To this, 5.0 mL of deionized water was added, precipitating a large amount of white flocculent material. The precipitate was collected by vacuum filtration, dissolved in dichloromethane, and the organic phase was washed three times with saturated sodium chloride solution, dried over anhydrous sodium sulfate, and concentrated by rotary evaporation to afford a white solid, **2NAP-NHS**. The product was dried under vacuum, yielding 264.0 mg with an isolated yield of 88.4%. ^{1}H NMR (500 MHz, DMSO-d_6) δ 8.79 (d, *J* = 2.0 Hz, 1H), 8.18 (d, *J* = 9.1 Hz, 1H), 8.07 – 7.98 (m, 2H), 7.53 (d, *J* = 2.7 Hz, 1H), 7.35 (dd, *J* = 9.1, 2.6 Hz, 1H), 3.96 (s, 3H), 2.94 (s, 4H).

Synthesis of compound MeOBP-NHS

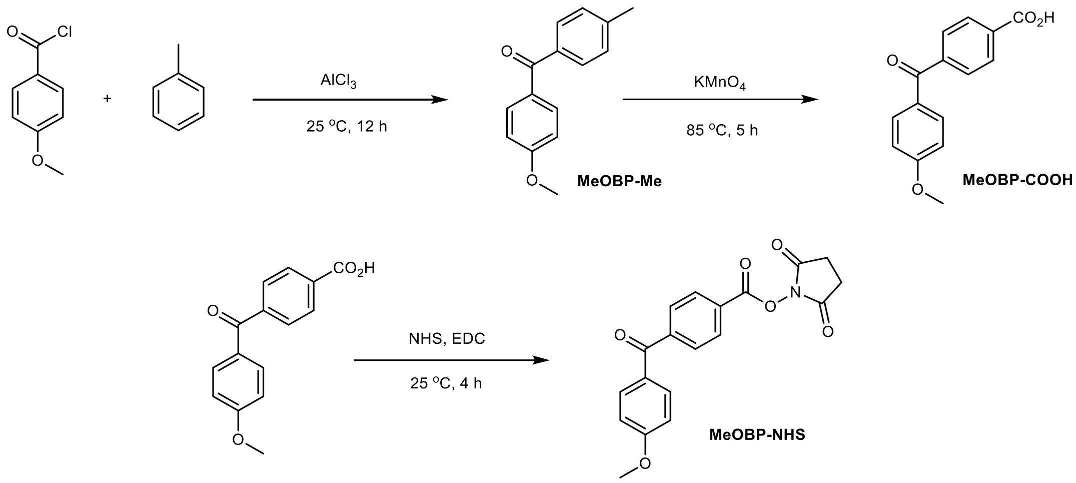

To a round-bottom flask, 4-methoxybenzoyl chloride (1.0 mL, 7.4 mmol) and toluene (2.0 mL, 18.9 mmol) were added, followed by the slow addition of $AlCl_3$ (1.6 g, 12.0 mmol). The reaction mixture was stirred at 25 ℃ for 12 hours. After the reaction was complete, dichloromethane was added to dilute the mixture, and the reaction mixture was slowly dripped into an ice-cold dilute hydrochloric acid solution to quench the reaction. The resulting mixture was extracted with dichloromethane, dried over anhydrous sodium sulfate, and concentrated via rotary evaporation. The crude product was loaded onto a wet silica gel column and purified using a gradient elution with dichloromethane/petroleum ether (ratios: 1:10, 1:3, 1:1, 2:1), yielding 864.6 mg of the white solid **MeOBP-Me**, with a yield of 54.7%.

Next, **MeOBP-Me** (226.3 mg, 1.0 mmol), potassium permanganate (790.0 mg, 5.0 mmol), tert-butanol (1.4 mL), and water (2.1 mL) were combined as a mixed solvent in a round-bottom flask and stirred at 85 ℃ for 5 hours. Upon completion, the reaction mixture was slowly dripped into an ice-cold dilute hydrochloric acid solution to quench the reaction. The resulting mixture was extracted with dichloromethane, dried over anhydrous sodium sulfate, and concentrated by rotary evaporation, yielding 209.0 mg of the white solid **MeOBP-COOH**, with a yield of 81.5%.

Finally, **MeOBP-COOH** (76.9 mg, 0.3 mmol), N-hydroxysuccinimide (173.0 mg, 0.9 mmol), 3-(3-dimethylaminopropyl)-1-ethylcarbodiimide hydrochloride (110.0 mg, 0.9 mmol), and dichloromethane (3.0 mL) were added to a round-bottom flask. The reaction mixture was stirred at 25 ℃ for 4 hours. After completion, the reaction was purified via silica gel column chromatography using dichloromethane/ethyl acetate (30:1) as the eluent, resulting in 49.6 mg white compound **MeOBP-NHS**, with a yield of 47.0%. ^{1}H NMR (500 MHz, DMSO-d_6) δ 8.25 (d, J = 8.4 Hz, 2H), 7.90 (d, J = 8.2 Hz, 2H), 7.80 (d, J = 8.8 Hz, 2H), 7.11 (d, J = 8.8 Hz, 2H), 3.87 (s, 3H), 2.92 (s, 4H).

Synthesis of compound MeOBip-NHS

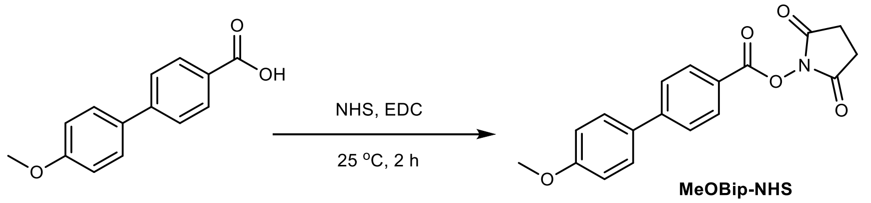

To a round-bottom flask, 4'-methoxy-[1,1'-biphenyl]-4-carboxylic acid (228.0 mg, 1.0 mmol), N-hydroxysuccinimide (345.0 mg, 3.0 mmol), 3-(3-dimethylaminopropyl)-1-ethylcarbodiimide hydrochloride (479.0 mg, 2.5 mmol), and dichloromethane (2.0 mL) were added. The reaction mixture was stirred at 25 ℃ for 2 hours. Upon completion, the reaction was purified via silica gel column chromatography, utilizing dichloromethane/ethyl acetate (50:1) as the eluent, resulting in a white compound, **MeOBip-NHS**. The compound was further purified by recrystallization. ^{1}H NMR (400 MHz, Chloroform-d) δ 8.17 (d, J = 8.6 Hz, 2H), 7.69 (d, J = 8.6 Hz, 2H), 7.59 (d, J = 8.9 Hz, 2H), 7.01 (d, J = 8.7 Hz, 2H), 3.87 (s, 3H), 2.92 (s, 4H).

Synthesis of compound Fluorene-NHS

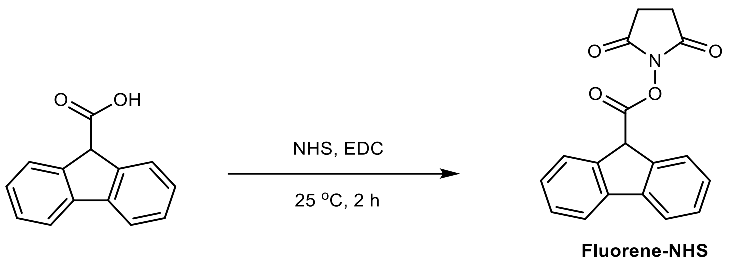

To a round-bottom flask, fluorene-9-carboxylic acid (210.0 mg, 1.0 mmol), N-hydroxysuccinimide (345.0 mg, 3.0 mmol), 3-(3-dimethylaminopropyl)-1-ethylcarbodiimide hydrochloride (479.0 mg, 2.5 mmol), and dichloromethane (2.0 mL) were added. The reaction mixture was stirred at 25 ℃ for 2 hours. Upon completion, the reaction was purified via silica gel column chromatography, utilizing dichloromethane/ethyl acetate (50:1) as the eluent, resulting in the formation of a white compound, **Fluorene-NHS**. The compound was further purified by recrystallization using spectroscopic-grade dichloromethane/hexane. ^{1}H NMR (400 MHz, Chloroform-*d*) δ 7.77 (t, *J* = 7.3 Hz, 4H), 7.47 (t, *J* = 7.6 Hz, 2H), 7.39 (t, *J* = 7.3 Hz, 2H), 2.83 (s, 4H).

Synthesis of compound NAPBP-NHS

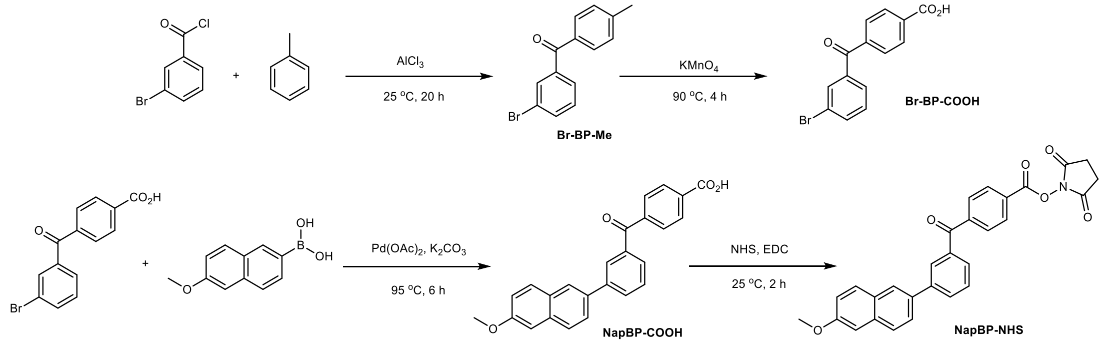

To a round-bottom flask, 3-bromobenzoylchloride (1.0 mL, 7.57 mmol) and toluene (1.0 mL, 9.48 mmol) were added, followed by the slow addition of $AlCl_3$ (912.0 mg, 6.86 mmol) under an ice-water bath. After stirring for 30 minutes, the reaction mixture was allowed to warm to 25 ℃ and reacted for 20 hours. The yellow reaction mixture was then slowly quenched by adding it dropwise into an ice-cold dilute hydrochloric acid solution, followed by stirring for an additional 30 minutes. The

desired product was extracted with dichloromethane, washed with deionized water, dried over anhydrous magnesium sulfate, and concentrated by rotary evaporation to obtain the white target product **Br-BP-Me** (1.825 g, 6.63 mmol) with a yield of 87.9%. ^{1}H NMR (400 MHz, Chloroform-*d*) δ 7.91 (s, 1H), 7.70 (t, *J* = 6.3 Hz, 4H), 7.32 (dd, *J* = 18.5, 7.9 Hz, 3H), 2.45 (s, 3H).

To a round-bottom flask, **Br-BP-Me** (1.825 g, 6.63 mmol), potassium permanganate (5.0 g, 31.6 mmol), tert-butanol (5.0 mL), and deionized water (10.0 mL) were added. The reaction mixture was heated to 90 ℃ and stirred for 4 hours. After cooling to room temperature, the reaction mixture was poured into ice-cold dilute hydrochloric acid. The target product was then extracted with a dichloromethane/acetone mixture and washed with deionized water. The organic layer was dried over anhydrous sodium sulfate and concentrated by rotary evaporation to yield the white target product **Br-BP-COOH** (1.656 g, 5.43 mmol) with a yield of 81.9%.

To a round-bottom flask, 6-methoxy-2-naphthylboronic acid (131.3 mg, 0.65 mmol), **Br-BP-COOH** (152.5 mg, 0.5 mmol), potassium carbonate (173.8 mg, 1.25 mmol), palladium acetate (5.6 mg, 0.025 mmol), ethanol (1.5 mL), and deionized water (0.75 mL) were added. The reaction mixture was heated to 95 ℃ and stirred for 6 hours. After cooling to room temperature, the reaction mixture was poured into dilute hydrochloric acid and stirred for 10 minutes. The crude product was extracted with a large amount of a dichloromethane/acetone solvent mixture and washed with deionized water. The organic layer was dried over anhydrous sodium sulfate and concentrated by rotary evaporation to obtain the crude product **NapBP-COOH.** Due to the poor solubility of **NapBP-COOH**, a purification method involving washing with a large volume of acetone and dichloromethane yielded the pure **NapBP-COOH** (129.4 mg, 0.34 mmol) with a yield of 67.6%. ^{1}H NMR (400 MHz, Acetone-d_6) δ 8.25 – 8.14 (m, 4H), 8.09 (d, *J* = 7.6 Hz, 1H), 7.97 – 7.87 (m, 4H), 7.85 – 7.76 (m, 2H), 7.69 (t, *J* = 7.6 Hz, 1H), 7.33 (d, *J* = 3.1 Hz, 1H), 7.18 (dd, *J* = 8.8, 2.7 Hz, 1H), 3.92 (s, 3H).

To a round-bottom flask, **NapBP-COOH** (38.2 mg, 0.1 mmol), N-hydroxysuccinimide (34.5 mg, 0.3 mmol), 3-(3-dimethylaminopropyl)-1-

ethylcarbodiimide hydrochloride (48.0 mg, 0.25 mmol), and dichloromethane (2.0 mL) were added. The reaction mixture was stirred at 25 ℃ for 2 hours. Upon completion, the reaction was purified via silica gel column chromatography, utilizing dichloromethane/ethyl acetate (50:1) as the eluent, yielding the target compound **NAPBP-NHS** (19.7 mg, 0.04 mmol) with an isolated yield of 41.1%. ^{1}H NMR (500 MHz, DMSO-d_6) δ 8.33 – 8.25 (m, 2H), 8.24 (d, J = 2.0 Hz, 1H), 8.18 – 8.12 (m, 2H), 8.07 – 8.00 (m, 2H), 7.94 (d, J = 9.3 Hz, 2H), 7.85 (dd, J = 8.6, 2.0 Hz, 1H), 7.77 (dt, J = 7.7, 1.6 Hz, 1H), 7.72 (t, J = 8.0 Hz, 1H), 7.37 (d, J = 2.7 Hz, 1H), 7.21 (dd, J = 8.9, 2.6 Hz, 1H), 3.90 (s, 3H), 2.92 (s, 4H). ^{13}C NMR (126 MHz, DMSO-d_6) δ 195.06, 170.20, 161.31, 157.64, 142.62, 140.59, 136.93, 133.93, 133.84, 131.57, 130.41, 130.30, 129.88, 129.50, 128.74, 128.68, 127.65, 127.59, 127.46, 125.58, 125.34, 119.14, 105.66, 55.22, 25.56. HRMS m/z found (calcd for $C_{33}H_{26}NO_5^+$): 516.1811 (516.1784).

Synthesis of compound FluoreneBP-NHS

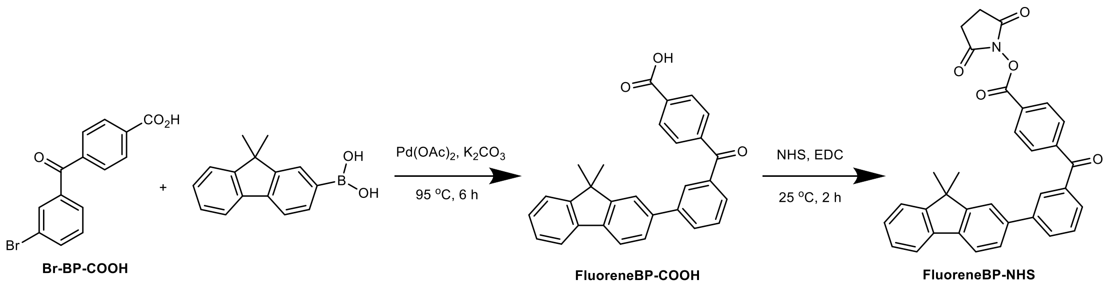

Synthesized the compound **Br-BP-COOH** via a synthetic route identical to aforementioned **NAPBP-NHS**. Then to a round-bottom flask, 9,9-dimethylfluoren-2-boronic acid (155.4 mg, 0.65 mmol), Br-BP-COOH (152.5 mg, 0.5 mmol), potassium carbonate (173.8 mg, 1.25 mmol), palladium acetate (5.6 mg, 0.025 mmol), ethanol (1.5 mL), and deionized water (0.75 mL) were added. The reaction mixture was heated to 95 ℃ and stirred for 6 hours. After cooling to room temperature, the reaction mixture was poured into dilute hydrochloric acid and stirred for 10 minutes. The crude product was extracted with a large volume of a dichloromethane/acetone solvent mixture and washed with deionized water. The organic phase was dried over anhydrous sodium sulfate, concentrated by rotary evaporation, and purified via silica gel column

chromatography using dichloromethane/ethyl acetate (30:1) as the eluent, yielding the white compound **FluoreneBP-COOH**.

To a round-bottom flask, **FluoreneBP-COOH** (83.7 mg, 0.2 mmol), N-hydroxysuccinimide (69.0 mg, 0.6 mmol), 3-(3-dimethylaminopropyl)-1-ethylcarbodiimide hydrochloride (97.5 mg, 0.5 mmol), and dichloromethane (2.0 mL) were added. The reaction mixture was stirred at 25 ℃ for 2 hours. Upon completion, the reaction was purified via silica gel column chromatography, utilizing dichloromethane/ethyl acetate (50:1) as the eluent, yielding the target compound **FluoreneBP-NHS**. ^{1}H NMR (500 MHz, DMSO-d_6) δ 8.33 – 8.26 (m, 2H), 8.11 (dt, *J* = 4.6, 2.1 Hz, 2H), 8.08 – 8.01 (m, 2H), 7.97 – 7.91 (m, 2H), 7.87 (dd, *J* = 6.5, 2.4 Hz, 1H), 7.76 (dt, *J* = 7.7, 1.6 Hz, 1H), 7.74–7.68 (m, 2H), 7.57 (dd, *J* = 6.3, 2.4 Hz, 1H), 7.35 (tt, *J* = 7.4, 5.8 Hz, 2H), 2.92 (s, 4H), 1.50 (s, 6H). ^{13}C NMR (126 MHz, DMSO-d_6) δ 195.07, 170.20, 161.31, 154.24, 153.70, 142.59, 141.01, 138.48, 138.21, 137.97, 136.92, 131.68, 130.42, 130.29, 129.36, 128.81, 127.69, 127.57, 127.48, 127.10, 126.04, 122.80, 121.44, 120.73, 120.35, 46.65, 26.78, 25.56. HRMS m/z found (calcd for $C_{33}H_{26}NO_5^+$): 516.1784 (516.1811).

Synthesis of compound PyreneBP-NHS

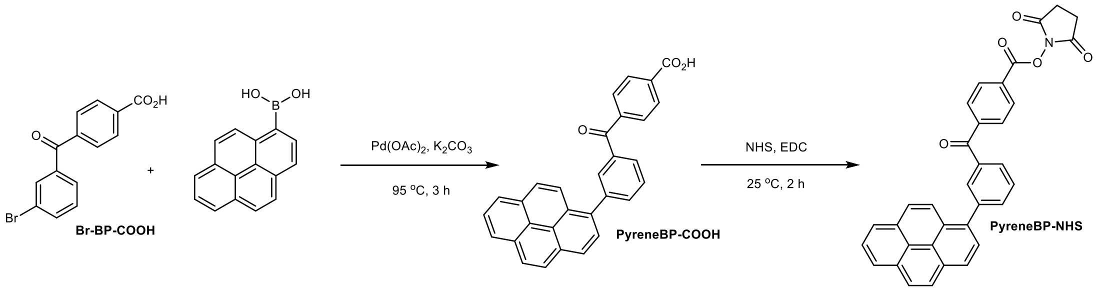

Synthesized the compound **Br-BP-COOH** via a synthetic route identical to aforementioned **NAPBP-NHS**. Then to a round-bottom flask, 1-pyreneboronic acid (159.9 mg, 0.65 mmol), **Br-BP-COOH** (152.5 mg, 0.5 mmol), potassium carbonate (173.8 mg, 1.25 mmol), palladium acetate (5.6 mg, 0.025 mmol), ethanol (1.5 mL), and deionized water (0.75 mL) were added. The reaction mixture was heated to 95 ℃ and stirred for 3 hours. After cooling to room temperature, the reaction mixture was poured into dilute hydrochloric acid and stirred for 10 minutes. The crude product was

extracted with a large volume of a dichloromethane/acetone solvent mixture and washed with deionized water. The solution was dried over anhydrous sodium sulfate and concentrated by rotary evaporation to obtain the crude product **PyreneBP-COOH**. Due to the poor solubility of **PyreneBP-COOH**, a purification method involving ultrasonic washing with a large volume of acetone and dichloromethane yielded 96.3 mg of the pure yellow solid **PyreneBP-COOH**, with a yield of 45.0%.

To a round-bottom flask, **PyreneBP-COOH** (39.0 mg, 0.09 mmol), N-hydroxysuccinimide (31.5 mg, 0.27 mmol), 3-(3-dimethylaminopropyl)-1-ethylcarbodiimide hydrochloride (44.0 mg, 0.23 mmol), and dichloromethane (2.0 mL) were added. The reaction mixture was stirred at 25 ℃ for 2 hours. Upon completion, the reaction was purified via silica gel column chromatography, utilizing dichloromethane/ethyl acetate (50:1) as the eluent, yielding 19.7 mg of the yellow solid **PyreneBP-NHS**, with a yield of 41.1%. ^{1}H NMR (500 MHz, DMSO-d_6) δ 8.39 (d, J = 7.9 Hz, 1H), 8.37–8.18 (m, 7H), 8.17 – 8.05 (m, 5H), 8.05–7.98 (m, 2H), 7.94 (d, J = 7.9 Hz, 1H), 7.84 (t, J = 7.9 Hz, 1H), 2.91 (s, 4H). ^{13}C NMR (126 MHz, DMSO-d_6) δ 195.45, 170.68, 161.78, 143.17, 141.18, 136.99, 136.18, 135.68, 131.87, 131.41, 130.98, 130.88, 130.83, 130.74, 129.69, 129.56, 128.61, 128.25, 128.21, 128.11, 127.91, 127.84, 127.01, 126.07, 125.68, 125.54, 124.70, 124.59, 124.44, 26.05. HRMS m/z found (calcd for $C_{34}H_{22}NO_5^+$): 524.1490 (524.1498).

Synthesis of compound BPBP-NHS

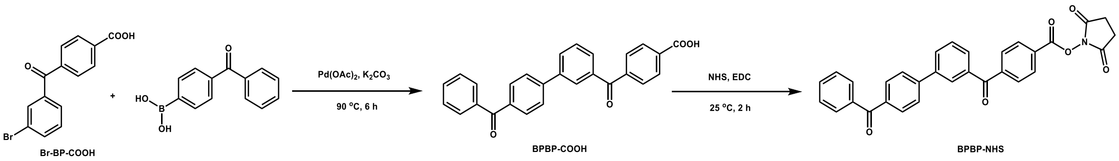

Synthesized the compound **Br-BP-COOH** via a synthetic route identical to aforementioned **NAPBP-NHS**. Then to a round-bottom flask, (4-benzoylphenyl)-boronic acid (146.9 mg, 0.65 mmol), **Br-BP-COOH** (152.5 mg, 0.5 mmol), potassium carbonate (173.8 mg, 1.25 mmol), palladium acetate (5.6 mg, 0.025 mmol), ethanol (1.5 mL), and deionized water (0.75 mL) were added. The reaction mixture was heated to 90 ℃ and stirred for 6 hours. After cooling to room temperature, the reaction mixture

was poured into dilute hydrochloric acid and stirred for 10 minutes. The crude product was extracted with a large volume of a dichloromethane/acetone solvent mixture and washed with deionized water. The solution was dried over anhydrous sodium sulfate and concentrated by rotary evaporation to obtain the crude product **BPBP-COOH**. Due to the poor solubility of **BPBP-COOH**, a purification method involving ultrasonic washing with a large volume of acetone and dichloromethane yielded 88.0 mg of the white solid **BPBP-COOH**, with a yield of 43.3%.

To a round-bottom flask, **BPBP-COOH** (88.0 mg, 0.22 mmol), N-hydroxysuccinimide (75.0 mg, 0.65 mmol), 3-(3-dimethylaminopropyl)-1-ethylcarbodiimide hydrochloride (104.0 mg, 0.54 mmol), and dichloromethane (2.0 mL) were added. The reaction mixture was stirred at 25 ℃ for 2 hours. Upon completion, the reaction was purified via silica gel column chromatography, utilizing dichloromethane/ethyl acetate (50:1) as the eluent, yielding 45.9 mg of the white solid **BPBP-NHS**, with a yield of 42.2%. ^{1}H NMR (500 MHz, Methylene Chloride-d_2) δ 8.29 (d, J = 8.5 Hz, 2H), 8.09 (s, 1H), 7.99 – 7.94 (m, 3H), 7.90 (d, J = 8.5 Hz, 2H), 7.85 – 7.80 (m, 3H), 7.78 – 7.74 (m, 2H), 7.69 – 7.60 (m, 2H), 7.52 (t, J = 7.6 Hz, 2H), 2.91 (s, 4H). HRMS m/z found (calcd for $C_{29}H_{23}O_3BF_2Na^+$): 504.1425 (504.1447).

Synthesis of compound MeOBipBP-NHS

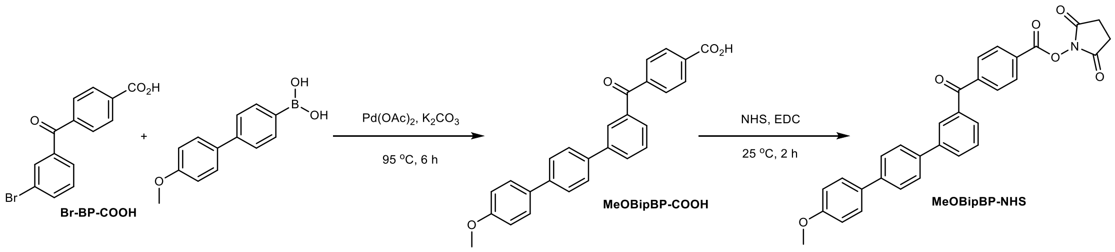

Synthesized the compound **Br-BP-COOH** via a synthetic route identical to aforementioned **NAPBP-NHS**. Then to a round-bottom flask, (4'-methoxy-[1,1'-biphenyl]-4-yl) boronic acid (148.2 mg, 0.65 mmol), **Br-BP-COOH** (152.5 mg, 0.5 mmol), potassium carbonate (173.8 mg, 1.25 mmol), palladium acetate (5.6 mg, 0.025 mmol), ethanol (1.5 mL), and deionized water (0.75 mL) were added. The reaction mixture was heated to 95 ℃ and stirred for 6 hours. After cooling to room temperature,

the reaction mixture was poured into dilute hydrochloric acid and stirred for 10 minutes. The crude product was extracted with a large volume of a dichloromethane/acetone solvent mixture and washed with deionized water. The organic phase was dried over anhydrous sodium sulfate, concentrated by rotary evaporation, and purified via silica gel column chromatography using dichloromethane/ethyl acetate (30:1) as the eluent, yielding 139.2 mg of the white compound **BipBP-COOH** with a yield of 68.2%.

To a round-bottom flask, **BipBP-COOH** (100.0 mg, 0.25 mmol), N-hydroxysuccinimide (84.5 mg, 0.73 mmol), 3-(3-dimethylaminopropyl)-1-ethylcarbodiimide hydrochloride (120.0 mg, 0.63 mmol), and dichloromethane (3.0 mL) were added. The reaction mixture was stirred at 25 ℃ for 2 hours. Upon completion, the reaction was purified via silica gel column chromatography, utilizing dichloromethane/ethyl acetate (50:1) as the eluent, yielding 81.4 mg of the white solid target compound **BipBP-NHS** with a yield of 65.8%. ^{1}H NMR (500 MHz, DMSO-d_6) δ 8.33–8.27 (m, 2H), 8.11–8.00 (m, 4H), 7.83–7.66 (m, 8H), 7.09–7.02 (m, 2H), 3.81 (s, 3H), 2.93 (s, 4H). ^{13}C NMR (126 MHz, DMSO-d_6) δ 194.99, 170.21, 161.31, 159.06, 142.63, 140.09, 139.40, 137.16, 136.85, 131.71, 131.34, 130.38, 130.28, 129.54, 128.82, 127.70, 127.66, 127.42, 127.34, 126.78, 114.42, 55.16, 25.56. HRMS m/z found (calcd for $C_{31}H_{24}NO_6^{+}$): 506.1576 (506.1604).

Synthesis of compound SpiroBP-NHS

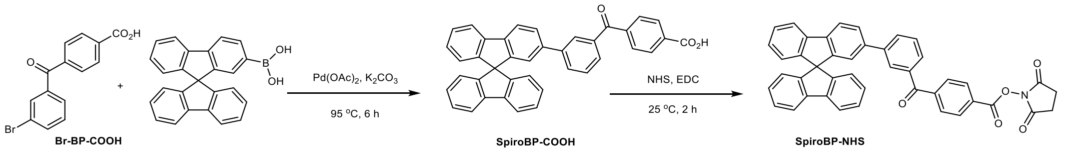

Synthesized the compound **Br-BP-COOH** via a synthetic route identical to aforementioned **NAPBP-NHS**. Then to a round-bottom flask, 9,9’-spirobifluorene-2-boronic acid (234.1 mg, 0.65 mmol), **Br-BP-COOH** (152.5 mg, 0.5 mmol), potassium carbonate (173.8 mg, 1.25 mmol), palladium acetate (5.6 mg, 0.025 mmol), ethanol (1.5 mL), and deionized water (0.75 mL) were added. The reaction mixture was heated to 95 ℃ and stirred for 6 hours. After cooling to room temperature, the reaction mixture was poured into dilute hydrochloric acid and stirred for 10 minutes. The crude product

was extracted with a large volume of a dichloromethane/acetone solvent mixture and washed with deionized water. The organic phase was dried over anhydrous sodium sulfate, concentrated by rotary evaporation, and purified via silica gel column chromatography using dichloromethane/ethyl acetate (30:1) as the eluent, yielding 141.9 mg of the white compound **SpiroBP-COOH** with a yield of 52.5%.

To a round-bottom flask, **SpiroBP-COOH** (115.0 mg, 0.19 mmol), N-hydroxysuccinimide (68.0 mg, 0.55 mmol), 3-(3-dimethylaminopropyl)-1-ethylcarbodiimide hydrochloride (88.0 mg, 0.46 mmol), and dichloromethane (3.0 mL) were added. The reaction mixture was stirred at 25 ℃ for 2 hours. Upon completion, the reaction was purified via silica gel column chromatography, utilizing dichloromethane/ethyl acetate (50:1) as the eluent, yielding 89.0 mg of the target compound **SpiroBP-NHS**, with a yield of 75.4%. ^{1}H NMR (500 MHz, DMSO-d_6) δ 8.18 (dd, J = 30.0, 8.1 Hz, 3H), 8.05 (dd, J = 22.6, 7.6 Hz, 3H), 7.90 (d, J = 8.4 Hz, 2H), 7.82–7.71 (m, 3H), 7.66 (d, J = 8.0 Hz, 1H), 7.54 (t, J = 7.8 Hz, 1H), 7.47–7.35 (m, 3H), 7.15 (dt, J = 15.0, 7.5 Hz, 3H), 6.89–6.79 (m, 1H), 6.64 (dd, J = 15.2, 7.6 Hz, 3H), 2.93 (s, 4H). ^{13}C NMR (126 MHz, DMSO-d_6) δ 194.76, 170.14, 161.20, 149.27, 148.41, 147.84, 142.50, 141.20, 140.58, 139.99, 138.68, 136.61, 131.26, 130.15, 130.04, 129.31, 128.69, 128.19, 127.98, 127.95, 127.69, 127.24, 126.92, 123.41, 121.31, 121.25, 120.77, 120.51, 65.38, 25.49. HRMS m/z found (calcd for $C_{43}H_{31}N_2O_5^+$): 655.2232 (655.2233).

Synthesis of compound TPABP-NHS

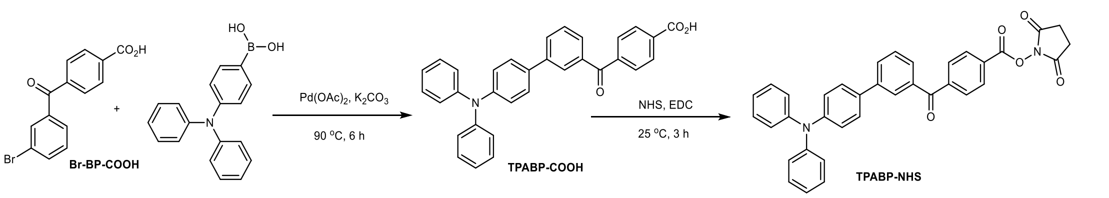

Synthesized the compound **Br-BP-COOH** via a synthetic route identical to aforementioned **NAPBP-NHS**. Then to a round-bottom flask, 4-(diphenylamino) phenylboronic acid (162.0 mg, 0.56 mmol), **Br-BP-COOH** (131.7 mg, 0.43 mmol), potassium carbonate (149.0 mg, 1.08 mmol), palladium acetate (7.0 mg, 0.03 mmol),

ethanol (1.5 mL), and deionized water (0.75 mL) were added. The reaction mixture was heated to 95 ℃ and stirred for 6 hours. After cooling to room temperature, the reaction mixture was poured into dilute hydrochloric acid and stirred for 10 minutes. The crude product was extracted with a large volume of a dichloromethane/acetone solvent mixture and washed with deionized water. The organic phase was dried over anhydrous sodium sulfate, concentrated by rotary evaporation, and purified via silica gel column chromatography using dichloromethane/ethyl acetate (30:1) as the eluent, yielding 76.0 mg of the yellow compound **TPABP-COOH** with a yield of 37.6%.

To a round-bottom flask, **TPABP-COOH** (43.4 mg, 0.09 mmol), N-hydroxysuccinimide (27.7 mg, 0.28 mmol), 3-(3-dimethylaminopropyl)-1-ethylcarbodiimide hydrochloride (44.4 mg, 0.23 mmol), and dichloromethane (3.0 mL) were added. The reaction mixture was stirred at 25 ℃ for 3 hours. Upon completion, the reaction was purified via silica gel column chromatography, utilizing dichloromethane/ethyl acetate (50:1) as the eluent, yielding 24.6 mg of the target compound **TPABP-NHS** (19.7 mg, 0.04 mmol), with a yield of 47.0%. ^{1}H NMR (500 MHz, DMSO-d_6) δ 8.28 (d, J = 8.4 Hz, 2H), 7.99 (dd, J = 13.0, 5.3 Hz, 4H), 7.75–7.59 (m, 4H), 7.32 (t, J = 7.8 Hz, 4H), 7.05 (q, J = 8.8, 8.0 Hz, 9H), 2.93 (s, 4H). ^{13}C NMR (126 MHz, DMSO-d_6) δ 195.01, 170.19, 161.30, 147.25, 146.86, 142.63, 140.04, 136.79, 132.53, 130.98, 130.31, 130.26, 129.61, 129.42, 128.34, 127.85, 127.41, 127.22, 124.31, 123.41, 122.96, 25.55. HRMS m/z found (calcd for $C_{36}H_{27}N_2O_5^+$): 567.1893 (567.1920).

Synthesis of compound 2COOHNAPBF$_2$

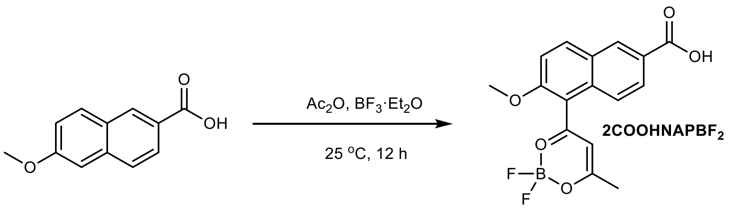

Into a round bottom flask were added 2-methoxy-6-naphthoic acid (202 mg, 1.00 mmol), acetic anhydride (2.00 mL, 21.2 mmol) and boron trifluoride diethyl etherate (0.65 mL, 5.24 mmol). The reaction mixture was kept at 25 ℃ and stirred for 12 hours.

The crude product was purified by column chromatography over silica gel using petroleum ether/dichloromethane (1:1), dichloromethane and dichloromethane/ethyl acetate (10:1) as gradient eluent to give green solids. The **$2COOHNAPBF_2$** was further purified by three cycles of recrystallization in spectroscopic grade acetone/hexane. Melting point of **$2COOHNAPBF_2$** was measured by DSC to be 260 ℃. ^{1}H NMR (400 MHz, acetone-*d6*) δ 11.41 (s, 1H), 8.68 (d, *J* = 1.8 Hz, 1H), 8.40 (d, *J* = 9.2 Hz, 1H), 8.13 (dd, *J* = 9.0, 1.8 Hz, 1H), 8.06 (d, *J* = 9.0 Hz, 1H), 7.71 (d, *J* = 9.2 Hz, 1H), 6.75 (s, 1H), 4.11 (s, 3H), 2.53 (s, 2H), 2.52 – 2.49 (m, 1H). ^{13}C NMR (101 MHz, acetone-*d6*) δ 206.26, 195.44, 185.93, 167.37, 159.34, 136.98, 134.66, 132.51, 128.75, 128.62, 127.38, 124.76, 117.05, 115.12, 107.01, 57.47, 24.83. ^{19}F NMR (376 MHz, acetone-*d6*) δ -137.66 (19.8%), -137.72 (80.2%). ^{11}B NMR (128 MHz, acetone-*d6*) δ 0.16. FT-IR (KBr, cm^{-1}): ν 2981, 2557, 1682, 1624, 1599,1538, 1484, 1465, 1443, 1419, 1378, 1366, 1314, 1291, 1251,1215, 1180, 1082, 1060, 1029, 978, 937, 923, 851, 823, 811, 785, 761, 713, 667, 621, 602, 580, 560, 508, 480, 421. LRMS, m/z 357.1. HRMS (positive DART) m/z found (calcd for $C_{16}H_{13}O_5BF_2Na^+$): 356.0750 (356.0753). The synthetic procedures are the same as our previous study (*ACS Appl. Mater. Interfaces* **2022**, *14*, 1587-1600).

Synthesis of compound $2COOHNAPBF_2$-NHS

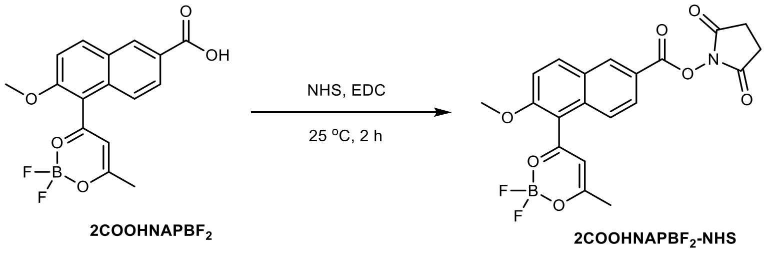

To a round-bottom flask, **$2COOHNAPBF_2$** (81.0 mg, 0.24 mmol), N-hydroxysuccinimide (84.0 mg, 0.74 mmol), 3-(3-dimethylaminopropyl)-1-ethylcarbodiimide hydrochloride (117.0 mg, 0.6 mmol), and acetone (12.0 mL) were added. The reaction mixture was stirred at 25 ℃ for 2 hours. Upon completion, the reaction mixture was concentrated by rotary evaporation, and the crude product was purified via silica gel column chromatography using dichloromethane/ethyl acetate

(50:1) as the eluent. The target compound **2COOHNAPBF$_2$-NHS** was further purified by recrystallization from spectroscopic grade dichloromethane/petroleum ether, yielding 46.0 mg of green crystals with a yield of 45.5%. ^{1}H NMR (400 MHz, Chloroform-*d*) δ 8.69 (s, 1H), 8.19 – 8.10 (m, 3H), 7.40 (d, *J* = 9.2 Hz, 1H), 6.42 (s, 1H), 4.03 (s, 3H), 2.93 (s, 4H). The single crystal structure of **2COOHNAPBF$_2$-NHS** can be found in Supplementary Fig. 99.

Synthesis of compound 0COOHNAPBF$_2$

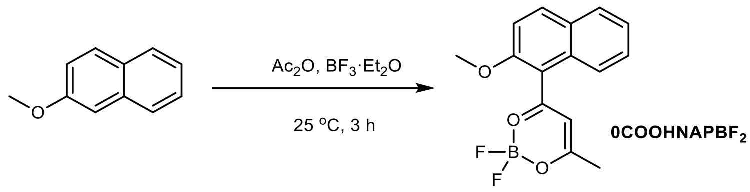

0COOHNAPBF$_2$

Into a round bottom flask were added 2-methoxynaphthalene (158 mg, 1.00 mmol), acetic anhydride (2.00 mL, 21.2 mmol) and boron trifluoride diethyl etherate (0.65 mL, 5.24 mmol). The reaction mixture was kept at 25 °C and stirred for 3 hours. Then the reaction was quenched by dropwisely adding the reaction mixture into cold water. The precipitates were washed by deionized water for three times and dried under vacuum. The crude product was purified by column chromatography over silica gel using petroleum ether/dichloromethane (1:1) as eluent to give yellow solids. The **0COOHNAPBF$_2$** was further purified by three cycles of recrystallization in spectroscopic grade dichloromethane/hexane. ^{1}H NMR (400 MHz, acetone-*d6*) δ 8.19 (d, *J* = 9.2 Hz, 1H), 8.00 (d, *J* = 8.6 Hz, 1H), 7.95 (d, *J* = 8.2 Hz, 1H), 7.64 - 7.55 (m, 2H), 7.46 (m, 1H), 6.72 (s, 1H), 4.05 (s, 3H), 2.51 (s, 3H). ^{13}C NMR (101 MHz, acetone-*d6*) δ 206.26, 194.72, 186.72, 157.72, 135.64, 132.41, 129.74, 129.56, 129.33, 125.45, 124.51, 116.99, 114.29, 106.95, 57.36, 24.72. ^{19}F NMR (376 MHz, chloroform-*d*) δ -132.56 (19.5%), -132.62 (80.5%). ^{11}B NMR (128 MHz, chloroform-*d*) δ 5.37. FT-IR (KBr, cm^{-1}): ν 3147, 2988, 2954, 2853, 1618, 1598, 1537, 1472, 1445, 1433, 1379, 1360, 1305, 1274, 1254, 1200, 1160, 1081, 1058, 1026, 978, 956, 918, 875, 821, 808, 786, 754, 734, 708, 667, 638, 598, 575, 534, 505, 496, 479, 412. LRMS, m/z 313.1. HRMS (positive ESI) m/z found (calcd for $C_{15}H_{13}O_3BF_2Na^+$): 312.0852 (312.0854).

The synthetic procedures are the same as our previous study (*ACS Appl. Mater. Interfaces* **2022**, *14*, 1587-1600).

Synthesis of compound 2COOHNAP-diketone

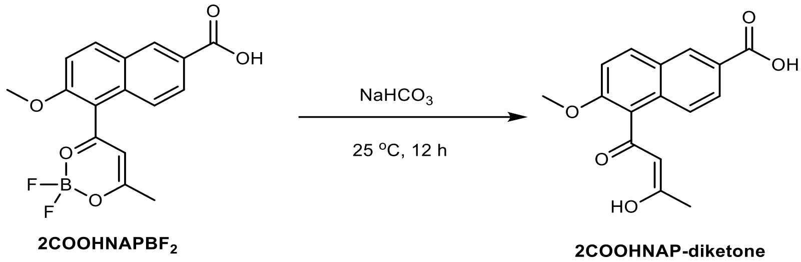

To a round-bottom flask, **2COOHNAPBF₂** (40.0 mg, 0.12 mmol), sodium bicarbonate (212.0 mg, 2.38 mmol), and deionized water (40.0 mL) were added. The reaction mixture was stirred at 25 °C for 12 hours. Afterward, the product was extracted with dichloromethane, dried over anhydrous sodium sulfate, and concentrated by rotary evaporation. The crude product was purified via silica gel column chromatography using dichloromethane/ethyl acetate (5:1) as the eluent, yielding 12.0 mg of white solid **2COOHNAP-diketone** with a yield of 34.8 %. ^{1}H NMR (500 MHz, DMSO-d_6) δ 13.03 (s, 1H), 8.60 (s, 1H), 8.29 (t, J = 10.3 Hz, 1H), 8.11 – 7.75 (m, 2H), 7.63 (dd, J = 9.3, 3.5 Hz, 1H), 5.96 (s, 1H), 3.98 (d, J = 25.9 Hz, 3H), 2.19 (d, J = 20.2 Hz, 3H). HRMS m/z found (calcd for $C_{33}H_{26}NO_5^+$): 287.0905 (287.0919).

Synthesis of compound BipCOOHBF₂

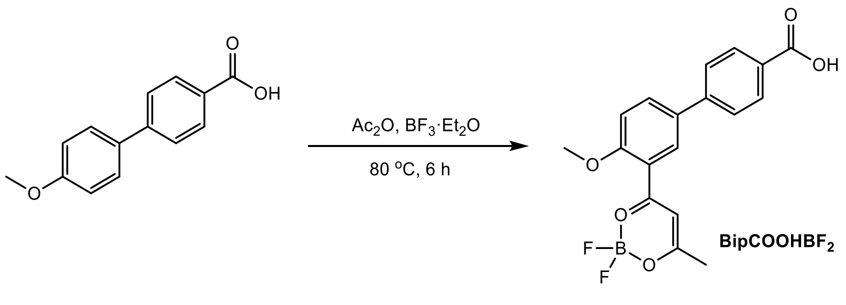

To a round-bottom flask, 4’-methoxy-[1,1’-biphenyl]-4-carboxylic acid (428.0 mg, 1.88 mmol), acetic anhydride (3.50 mL, 37.1 mmol), and boron trifluoride diethyl etherate (1.0 mL, 8.06 mmol) were added. The reaction mixture was stirred at 80 ℃ for 6 hours. After cooling to room temperature, the reaction mixture was quenched by

adding it dropwise into cold water. The crude product was then extracted with dichloromethane and washed with deionized water. The organic phase was dried over anhydrous sodium sulfate, concentrated by rotary evaporation, and directly subjected to wet loading onto a silica gel column. The target compound was purified using column chromatography with ethyl acetate/dichloromethane (1:30) as eluent, yielding 87.4 mg of a yellow-green solid, **BipCOOHBF$_2$**, with a yield of 12.9%. The compound was further purified by three recrystallizations from spectroscopic grade acetone/hexane. ^{1}H NMR (400 MHz, DMSO-d_6) δ 8.20 (d, J = 2.5 Hz, 1H), 8.11 (dd, J = 8.8, 2.5 Hz, 1H), 8.04 – 7.99 (m, 2H), 7.82 – 7.77 (m, 2H), 7.42 (d, J = 9.0 Hz, 1H), 7.24 (s, 1H), 4.03 (s, 3H). ^{13}C NMR (126 MHz, DMSO-d_6) δ 194.95, 179.63, 167.55, 161.10, 142.89, 135.88, 132.24, 130.61, 130.11, 128.98, 126.95, 120.18, 114.60, 103.04, 57.15, 25.14. ^{19}F NMR (376 MHz, DMSO-d_6) δ -135.82, -135.88. ^{11}B NMR (128 MHz, DMSO-d_6) δ 0.20. HRMS m/z found (calcd for $C_{18}H_{19}BF_2NO_5^+$): 377.1351 (377.1355).

Synthesis of compound FluCOOHBF$_2$

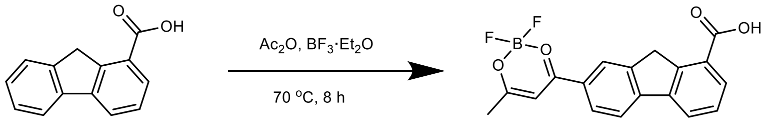

FluCOOHBF$_2$

To a round-bottom flask, 9H-fluorene-1-carboxylic acid (149.0 mg, 0.71 mmol), acetic anhydride (1.6 mL, 17.0 mmol), and boron trifluoride diethyl etherate (0.65 mL, 5.24 mmol) were added. The reaction mixture was stirred at 70 ℃ for 8 hours. After cooling to room temperature, the reaction mixture was directly subjected to wet loading onto a silica gel column. The target compound was purified using silica gel column chromatography with ethyl acetate/dichloromethane (1:30) as the eluent, yielding a yellow solid, **FluCOOHBF$_2$**. Due to the poor solubility of **FluCOOHBF$_2$**, further purification was achieved by washing with a large volume of acetone and dichloromethane, yielding 53 mg of a pale-yellow solid with a yield of 15.5%.^{1}H NMR (400 MHz, Acetone-d_6) δ 8.45 (s, 1H), 8.28 (dd, J = 17.5, 7.6 Hz, 2H), 8.15 (dd, J = 18.9, 7.9 Hz, 2H), 7.63 (t, J = 7.7 Hz, 1H), 7.15 (s, 1H), 4.40 (s, 2H), 2.46 (s, 3H). ^{13}C NMR (126 MHz, DMSO-d_6) δ 193.69, 181.67, 167.75, 147.82, 146.90, 144.83, 141.30,

130.88, 129.61, 128.99, 128.38, 128.28, 126.40, 126.15, 121.56, 98.64, 38.82, 24.88. ^{11}B NMR (128 MHz, DMSO-d_6) δ 0.03. ^{19}F NMR (376 MHz, DMSO-d_6) δ -136.18, -136.25. HRMS m/z found (calcd for $C_{18}H_{17}BF_2NO_4^+$): 360.1219 (360.1206).

Synthesis of compound 1COOHNAPBF$_2$

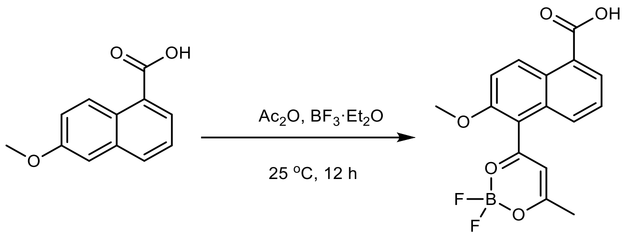

1COOHNAPBF$_2$

Into a round bottom flask were added 6-methoxy-1-naphthoic acid (202 mg, 1.00 mmol), acetic anhydride (2.00 mL, 21.2 mmol) and boron trifluoride diethyl etherate (0.65 mL, 5.24 mmol). The reaction mixture was kept at 25 °C and stirred for 12 hours. The crude product was purified by column chromatography over silica gel using petroleum ether/dichloromethane (1:1), dichloromethane and dichloromethane/ethyl acetate (10:1) as gradient eluent to give white solids. The **1COOHNAPBF$_2$** was further purified by three cycles of recrystallization in spectroscopic grade acetone/hexane. ^{1}H NMR (400 MHz, acetone-*d6*) δ 11.56 (s, 1H), 9.28 (d, J = 9.8 Hz, 1H), 8.21 (dd, J = 7.2, 1.2 Hz, 1H), 8.15 (d, J = 8.7 Hz, 1H), 7.74 – 7.65 (m, 2H), 6.73 (s, 1H), 5.63 (s, 1H), 4.08 (s, 3H), 2.53 (s, 2H), 2.53 – 2.51 (m, 1H). ^{13}C NMR (101 MHz, acetone-*d6*) δ 206.26, 195.40, 186.23, 168.59, 156.91, 132.94, 132.87, 129.40, 129.13, 128.75, 127.94, 127.18, 117.35, 115.43, 107.10, 57.18, 24.66. ^{19}F NMR (376 MHz, acetone-*d6*) δ -137.73 (19.8%), -137.80 (80.2%). ^{11}B NMR (128 MHz, acetone-*d6*) δ 0.17. FT-IR (KBr, cm^{-1}): ν 2972, 2641, 1687, 1615, 1594, 1546, 1470, 1412, 1363, 1324, 1286, 1266, 1232, 1214, 1153, 1106, 1074,1054, 978, 937, 897, 840, 802, 755, 727, 673, 591, 511, 480, 415. LRMS, m/z 357.1. HRMS (positive ESI) m/z found (calcd for $C_{16}H_{13}O_5BF_2Na^+$): 356.0752 (356.0753). The synthetic procedures are the same as our previous study (*ACS Appl. Mater. Interfaces* **2022**, *14,* 1587-1600).

Synthesis of compound $TPABF_2$

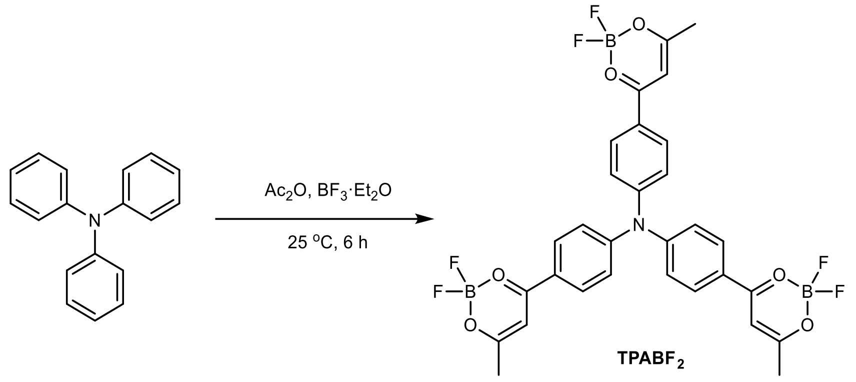

To a round-bottom flask, triphenylamine (245.3 mg, 1.00 mmol), acetic anhydride (2.0 mL, 21.6 mmol), and boron trifluoride diethyl etherate (0.65 mL, 5.24 mmol) were added. The reaction mixture was stirred at 25 ℃ for 6 hours. Upon completion, the reaction mixture was slowly added dropwise into cold water to quench the reaction. The resulting mixture was washed three times with deionized water. The organic phase was dried over anhydrous sodium sulfate, and the filtrate was collected and concentrated. The crude product was directly subjected to wet loading onto a silica gel column. The target compound was purified using silica gel column chromatography with petroleum ether/dichloromethane (2:1) as the eluent, yielding 87.3 mg yellow solid, **$TPABF_2$**, with a yield of 12.9%. Further purification was achieved by recrystallization from spectroscopic grade dichloromethane/hexane. ^1H NMR (500 MHz, DMSO-d_6) δ 8.24 – 8.17 (m, 2H), 7.36 (d, J = 8.8 Hz, 2H), 7.23 (s, 1H), 2.45 (s, 3H). ^{13}C NMR (126 MHz, DMSO-d_6) δ 193.16, 179.71, 151.32, 131.30, 126.42, 124.90, 97.85, 24.40.

Synthesis of compound $BrTPABF_2$

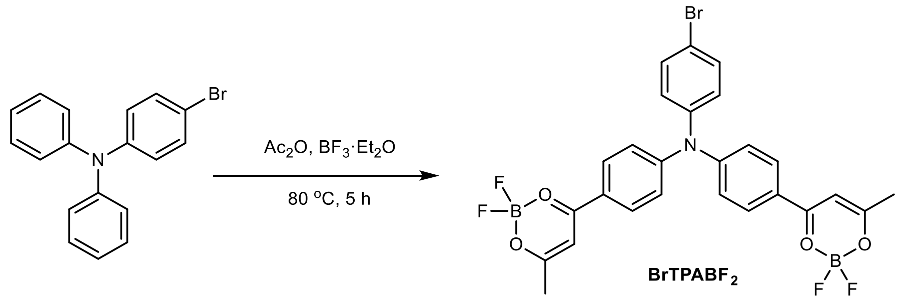

To a round-bottom flask, (4-bromophenyl) diphenylamine (324.0 mg, 1.00 mmol), acetic anhydride (2.0 mL, 21.6 mmol), and boron trifluoride diethyl etherate (0.65 mL,

5.24 mmol) were added. The reaction mixture was stirred at 80 ℃ for 5 hours. After cooling to room temperature, the reaction mixture was quenched by adding it dropwise into cold water. The crude product was then extracted with dichloromethane and washed with deionized water. The organic phase was dried over anhydrous sodium sulfate, concentrated by rotary evaporation, and directly subjected to wet loading onto a silica gel column. The target compound was purified using silica gel column chromatography with petroleum ether/dichloromethane (1:3) as the eluent, yielding a yellow solid, **BrTPABF$_2$**. Further purification was achieved by recrystallization from spectroscopic grade dichloromethane/hexane, affording 78.4 mg of orange-yellow crystalline blocks with a yield of 17.2%. ^{1}H NMR (500 MHz, DMSO-d_6) δ 8.18 – 8.11 (m, 2H), 7.72 – 7.66 (m, 1H), 7.24 (dd, J = 8.9, 7.0 Hz, 3H), 7.16 (s, 1H), 2.43 (s, 3H). ^{13}C NMR (126 MHz, DMSO-d_6) δ 192.18, 179.70, 151.86, 143.69, 133.37, 131.21, 129.30, 124.80, 122.86, 119.45, 97.43, 24.23. ^{19}F NMR (376 MHz, DMSO-d_6) δ -136.54, -136.60. HRMS m/z found (calcd for $C_{26}H_{24}B_2BrF_4N_2O_4^+$): 607.1021 (607.1028).

Synthesis of compound BrNAPBF$_2$

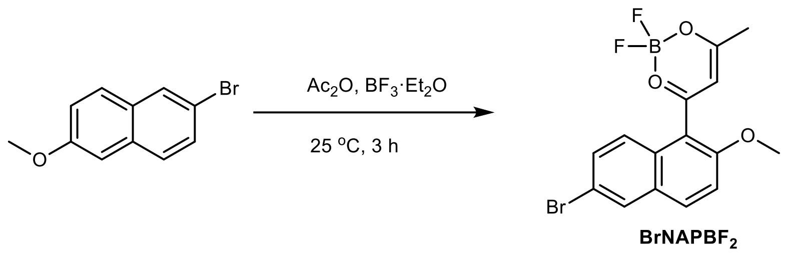

To a round-bottom flask, 6-methoxy-2-bromonaphthalene (237.0 mg, 1.0 mmol), acetic anhydride (2.0 mL, 21.6 mmol), and boron trifluoride diethyl etherate (0.65 mL, 5.24 mmol) were added. The reaction mixture was stirred at 25 ℃ for 3 hours. After completion, the reaction mixture was quenched by adding it dropwise into cold water. The crude product was then extracted with dichloromethane and washed with deionized water. The organic phase was dried over anhydrous sodium sulfate, concentrated by rotary evaporation, and directly subjected to wet loading onto a silica gel column. The target compound was purified using silica gel column chromatography with petroleum

ether/dichloromethane (2:1) as the eluent, yielding a yellow solid, **BrNAPBF$_2$**. The compound was further purified by recrystallization from spectroscopic grade acetone/hexane, repeated twice. ^{1}H NMR (500 MHz, DMSO-*d*$_6$) δ 8.30 (d, *J* = 2.2 Hz, 1H), 8.23 (d, *J* = 9.3 Hz, 1H), 7.81 – 7.65 (m, 3H), 6.79 (s, 1H), 4.00 (s, 3H). ^{13}C NMR (126 MHz, DMSO-*d*$_6$) δ 194.96, 183.79, 156.66, 133.90, 131.33, 130.47, 129.38, 129.29, 125.25, 117.30, 115.20, 115.01, 106.27, 24.50. ^{11}B NMR (128 MHz, DMSO-*d*$_6$) δ -0.02. ^{19}F NMR (376 MHz, DMSO-*d*$_6$) δ -134.83, -134.90. HRMS m/z found (calcd for $C_{15}H_{16}BBrF_2NO_3^+$): 385.0406 (385.0406).

Synthesis of compound OMe2NAPBF$_2$

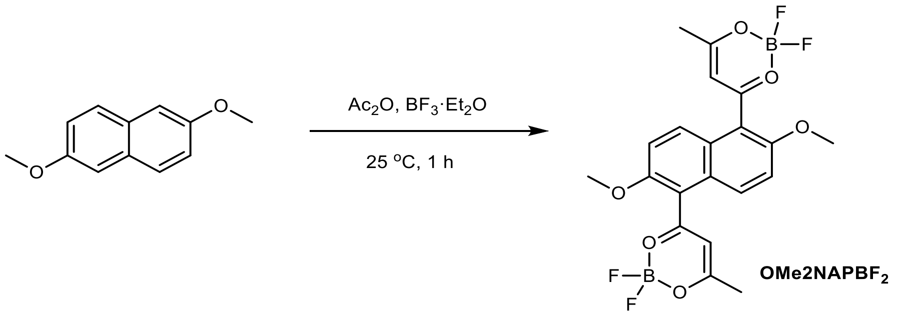

To a round-bottom flask, 2,6-dimethoxynaphthalene (188.2 mg, 1 mmol), acetic anhydride (2.0 mL, 21.6 mmol), and boron trifluoride diethyl etherate (0.65 mL, 5.24 mmol) were added. The reaction mixture was stirred at 25 ℃ for 1 hour. Upon completion, the reaction mixture was slowly added dropwise into cold water to quench the reaction. The resulting mixture was washed three times with deionized water. The organic phase was dried over anhydrous sodium sulfate, and the filtrate was collected and concentrated. The crude product was then purified by column chromatography using petroleum ether/dichloromethane (1:1) as the eluent, yielding a yellow solid, **OMe2NAPBF$_2$**. ^{1}H NMR (400 MHz, Chloroform-*d*) δ 8.26 (d, *J* = 9.3 Hz, 1H), 7.37 (d, *J* = 9.5 Hz, 91H), 6.45 (s, 1H), 3.96 (s, 3H), 2.44 (s, 3H). ^{13}C NMR (101 MHz, DMSO-*d*$_6$) δ 195.84, 184.21, 155.28, 129.83, 126.23, 116.89, 116.36, 106.98, 57.56, 25.06. ^{11}B NMR (128 MHz, DMSO-*d*$_6$) δ 0.06. ^{19}F NMR (376 MHz, DMSO-*d*$_6$) δ -134.86, -134.92.

Synthesis of compound BipBF$_2$

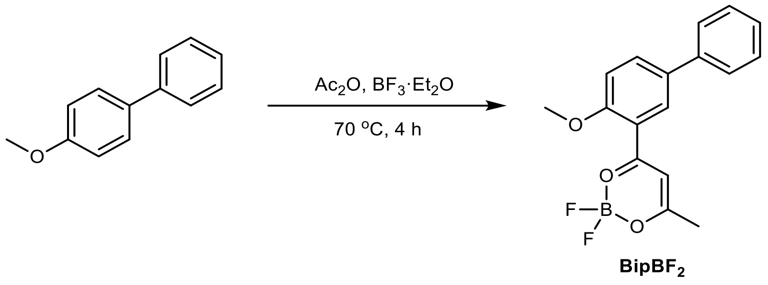

Into a round bottom flask were 4-methoxybiphenyl (300.0 mg, 1.63 mmol), acetic anhydride (4.0 mL, 69.9 mmol) and boron trifluoride diethyl etherate (0.60 mL, 4.75 mmol). The reaction mixture was kept at 70 °C and stirred for 4 h. Then the reaction was quenched by dropwisely adding the reaction mixture into cold water. Subsequently, the crude product was extracted with dichloromethane and washed with deionized water. Dry the solution on anhydrous sodium sulfate and purify the target product by column chromatography over silica gel using ethyl petroleum ether/ ethyl acetate (1:3) as eluent to give yellow solid **BipBF$_2$**. The **BipBF$_2$** was further purified by three cycles of recrystallization in spectroscopic grade acetone/hexane. ^{1}H NMR (400 MHz, DMSO-*d*$_6$) δ 8.20 (d, *J* = 2.5 Hz, 1H), 8.11 (dd, *J* = 8.8, 2.5 Hz, 1H), 8.04 – 7.99 (m, 2H), 7.82 – 7.77 (m, 2H), 7.42 (d, *J* = 9.0 Hz, 1H), 7.24 (s, 1H), 4.03 (s, 3H). ^{13}C NMR (101 MHz, Chloroform-d) δ 190.42, 182.03, 162.84, 136.72, 132.16, 131.87, 131.15, 129.51, 128.37, 127.98, 123.49, 111.50, 96.59, 56.21, 24.66. ^{19}F NMR (376 MHz, Chloroform-d, relative to $CFCl_3$ /ppm) δ -138.50, -138.55. ^{11}B NMR (128 MHz, Chloroform-*d*, relative to $BF_3 \cdot Et_2O$ /ppm) δ -0.01. FT-IR (KBr, cm^{-1}): ν 3156.7, 3009.9, 2957.1, 1614.4, 1582.7, 1536.7, 1485.0, 1474.4, 1452.5, 1436.8, 1411.1,1360.9, 1338.9, 1297.5, 1265.7, 1256.3, 1213.5, 1166.4, 1102.3, 1078.6, 1050.5, 1001.6, 977.7, 952.4, 916.6, 900.7, 849.0, 824.6, 813.2, 765.0, 710.7, 703.8, 655.7, 616.8, 606.3, 567.1, 532.1, 492.3. LRMS, m/z 339.1. HRMS (positive ESI) m/z found (calcd for $C_{17}H_{15}O_3BF_2Na^+$): 338.1009 (338.1011). The synthetic procedures are the same as our previous study (*Angew. Chem. Int. Ed.* **2021**, *60*, 17138–17147).

Synthesis of compound FluBF2

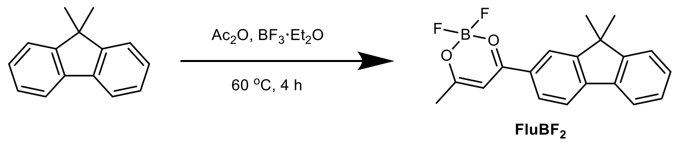

Into a round bottom flask were added 9,9-dimethyl-9*H*-fluorene (300 mg, 0.9 mmol), acetic anhydride (5 mL), and boron trifluoride diethyl etherate (0.4 mL). The reaction mixture was heated to 60 °C and stirred for 4 h. Then the reaction was quenched by adding the reaction mixture dropwise into cold water. The precipitates were washed by water for three times and dried under vacuum. The crude product was purified by column chromatography over silica gel using petroleum ether/dichloromethane (1:1) as eluent to give yellow solids with an isolation yield of 50%. The **FluBF2** was further purified by three cycles of recrystallization in spectroscopic grade dichloromethane/hexane. Melting point of **FluBF2** was measured by DSC to be 230 °C. ^{1}H NMR (400 MHz, Chloroform-*d*, relative to Me_4Si /ppm) *d* 8.17 (d, *J* = 1.7 Hz, 1H), 8.04 (dd, *J* = 8.1, 1.7 Hz, 1H), 7.88–7.75 (m, 2H), 7.53–7.36 (m, 3H), 6.63 (s, 1H), 2.43 (s, 3H), and 1.54 (s, 6H). ^{13}C NMR (100 MHz, Chloroform-*d*) *d* 191.28, 182.90, 155.35, 154.61, 147.23, 137.41, 129.77, 129.07, 127.66, 123.63, 123.16, 121.63, 120.55, 97.40, 47.35, 26.95, and 24.83. ^{19}F NMR (376 MHz, 298K, Chloroform-*d*, relative to $CFCl_3$ /ppm) *d* −139.08 (20%) and −139.14 (80%). ^{11}B NMR (128 MHz, Chloroform-*d*, 298 K, relative to $BF_3{\cdot}Et_2O$ /ppm) *d* 0.10. FT-IR (KBr, cm^{-1}): ν 2966, 2926, 2863, 1610, 1534, 1473, 1440, 1428, 1357, 1336, 1210, 1158, 1109, 1088, 1055, 1007, 978, 912, 828, 792, 776, 761, 736, 639, 585, 568, 488, and 427. LRMS, m/z 348.1. HRMS (positive ESI) m/z found (calcd for $C_{19}H_{17}O_2BF_2Na^+$): 348.1216 (348.1218). The synthetic procedures are the same as our previous study (*Adv. Opt. Mater.* **2021**, *9*, 2100353).

Synthesis of compound PyreneBF₂

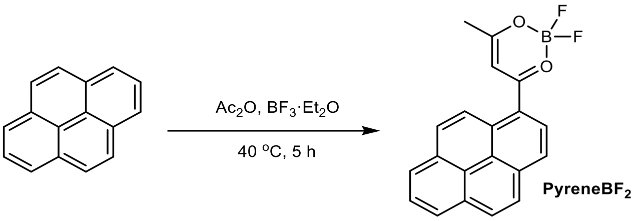

Into a round bottom flask were added pyrene (200 mg, 1.0 mmol), acetic anhydride (2 mL), and boron trifluoride diethyl etherate (0.6 mL). The reaction mixture was heated to 40 °C and stirred for 2 h. Then the reaction was quenched by adding the reaction mixture dropwise into cold water. The precipitates were washed by water for three times and dried under vacuum. The crude product was purified by column chromatography over silica gel using petroleum ether/dichloromethane (2:1) as eluent to give orange solids with an isolation yield of 48%. The **PyreneBF$_2$** was further purified by three cycles of recrystallization in spectroscopic grade dichloromethane/hexane. Melting point of **PyreneBF$_2$** was measured by DSC to be 237 °C. ^{1}H NMR (400 MHz, Chloroform-*d*, relative to Me_4Si/ppm) *d* 8.87 (d, J = 9.3 Hz, 1H), 8.33(t, J = 8.4 Hz, 2H), 8.31 (d, J = 4.8 Hz, 1H), 8.29 (d, J = 9.6 Hz, 1H), 8.25 (d, J = 8.9 Hz, 1H), 8.19 (d, J = 8.2 Hz, 1H), 8.11 (t, J = 7.6 Hz, 1H), 8.10 (d, J = 8.8 Hz, 1H), 6.67 (s, 1H), and 2.50 (s, 3H). ^{13}C NMR (100 MHz, Chloroform-*d*) *d* 191.61, 187.15, 135.75, 131.14, 131.04, 130.74, 130.52, 127.85, 127.42, 127.27, 127.24, 127.02, 126.09, 125.11, 124.59, 124.35, 124.16, 102.63, and 24.96. ^{19}F NMR (376 MHz, Chloroform-*d*, 298 K, relative to $CFCl_3$ /ppm) *d* −138.20 (20.20%) and −138.27 (79.80%). ^{11}B NMR (128 MHz, Chloroform-*d*, 298 K, relative to $BF_3·Et_2O$ /ppm) *d* 0.26. FT-IR (KBr, cm^{-1}): ν 1538, 1436, 1413, 1391, 1378, 1340, 1303, 1247, 1223, 1167, 1138, 1060, 1044, 1029, 980, 903, 848, 826, 751, 733, 713, 558, and 495. LRMS, m/z 351.1. HRMS (positive DART) m/z found (calcd for $C_{20}H_{17}NO_2BF_2^+$): 351.1351 (351.1351). The synthetic procedures are the same as our previous study (*Adv. Opt. Mater.* **2021**, *9*, 2100353).

Synthesis of compound $CorBF_2$

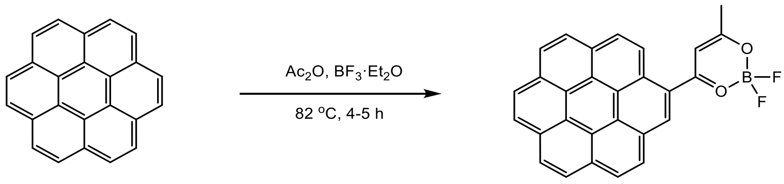

$CorBF_2$

Into a round bottom flask were added coronene (300 mg, 1.0 mmol), acetic anhydride (4 mL) and boron trifluoride diethyl etherate (0.7 mL). The reaction mixture was heated to 82 °C and stirred for 4-5 h. Then the reaction was quenched by adding the reaction mixture dropwise into cold water. The precipitates were washed by water for three times and dried under vacuum. The crude product was purified by column chromatography over silica gel using petroleum ether/dichloromethane (2:1-1:2) as eluent to give orange solids with an isolation yield of 10%. The **$CorBF_2$** was further purified by three cycles of recrystallization in spectroscopic grade dichloromethane/hexane. ^{1}H NMR (400 MHz, DMSO-d6) δ 9.53 (s, 1H), 9.37 (d, J = 9.0 Hz, 1H), 9.12-8.95 (m, 9H), 7.52 (s, 1H), 2.72 (s, 3H). ^{19}F NMR (376 MHz, DMSO-d6), δ-135.23, -135.29, relative to $CFCl_3$ /ppm. FT-IR (KBr, cm-1): ν 3020, 1908, 1609, 1457, 1433, 1391, 1338, 1304, 1162, 1066, 1046, 1026, 980, 910, 848, 816, 760, 688, 605, 578, 547, 489, 473. LRMS, m/z 432. HRMS (negative ESI) m/z found (calcd for $C_{28}H_{14}O_2{}^{10}BF_2$) 430.1097 (430.1098). The synthetic procedures are the same as our previous study (*J. Mater. Chem. C* ***2023***, *11*, 2291–2301).

Synthesis of compound $DCorBF_2$

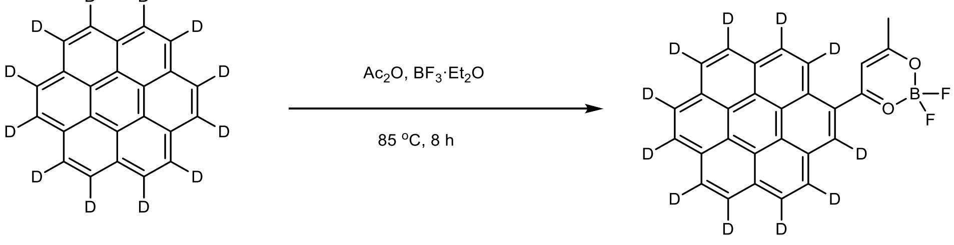

DCor, deuteration yield of 84.3%

$DCorBF_2$

The deuterated-coronene (**DCor**) utilized in this study was reported by our group previously, and ^{1}H NMR characterization revealed a deuteration yield of 84.3% (*J. Phys. Chem. C* **2021**, *125*, 26986–26998).

Into a round bottom flask were added deuteration-coronene (60 mg, 0.2 mmol), acetic anhydride (0.4 mL) and boron trifluoride diethyl etherate (0.14 mL). The reaction mixture was heated to 85 °C and stirred for 8 h. Then the reaction was quenched by adding the reaction mixture dropwise into cold water. The precipitates were washed by water for three times and dried under vacuum. The crude product was purified by column chromatography over silica gel using petroleum ether/dichloromethane (2:1-1:2) as eluent to give orange solids with an isolation yield of 2.3%. The synthetic procedures are the same as our previous study (*J. Mater. Chem. C* **2023**, *11*, 2291–2301).

Synthesis of compound NAP2BF$_2$

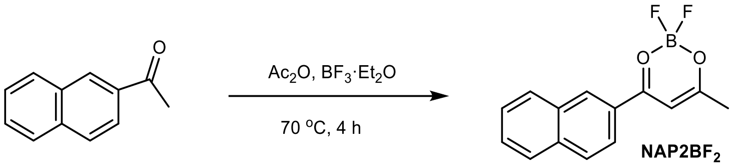

To a round-bottom flask, 2-acetonaphthone (170.0 mg, 1.0 mmol), acetic anhydride (2.0 mL, 21.6 mmol), and boron trifluoride diethyl etherate (0.65 mL, 5.24 mmol) were added. The reaction mixture was stirred at 70 ℃ for 4 hours. After cooling to room temperature, the reaction mixture was quenched by adding it dropwise into cold water. The crude product was then extracted with dichloromethane and washed with deionized water. The organic phase was dried over anhydrous sodium sulfate, concentrated by rotary evaporation, and directly subjected to wet loading onto a silica gel column. The target compound was purified using silica gel column chromatography with petroleum ether/dichloromethane (2:1) as the eluent, yielding the target crystal **NAP2BF$_2$**. Further purification was achieved by recrystallization from spectroscopic grade dichloromethane/hexane. ^{1}H NMR (400 MHz, Chloroform-*d*) δ 8.70 (s, 1H), 7.99 (dd, *J* = 8.7, 1.7 Hz, 2H), 7.94 (d, *J* = 8.7 Hz, 1H), 7.90 (d, *J* = 8.1 Hz, 1H), 7.71 – 7.65

(m, 1H), 7.63 – 7.58 (m, 1H), 6.72 (s, 1H), 2.46 (s, 3H).^{19}F NMR (376 MHz, Chloroform-*d*) δ -138.74, -138.80. ^{13}C NMR (101 MHz, DMSO-d_6) δ 194.09, 181.10, 136.15, 132.19, 131.79, 130.13, 130.08, 129.19, 127.94, 127.90, 127.63, 123.50, 98.62, 24.54. ^{11}B NMR (128 MHz, DMSO-d_6) δ 0.03. HRMS m/z found (calcd for $C_{14}H_{11}BF_2O_2Na^+$): 283.0718 (283.0715).

Synthesis of compound 6OMeNAPBF₂

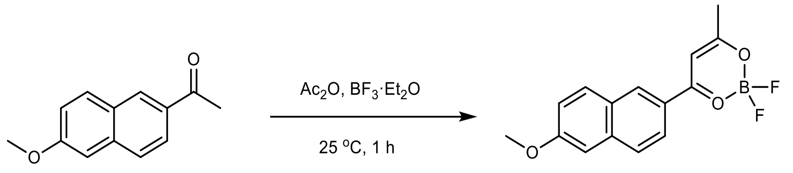

6OMeNAPBF$_2$

To a round-bottom flask, 6-methoxy-2-acetylnaphthalene (200.0 mg, 1.0 mmol), acetic anhydride (2.0 mL, 21.6 mmol), and boron trifluoride diethyl etherate (0.65 mL, 5.24 mmol) were added. The reaction mixture was stirred at 25 ℃ for 1 hour. Upon completion, the reaction mixture was quenched by adding it dropwise into cold water. The crude product was then extracted with dichloromethane and washed with deionized water. The organic phase was dried over anhydrous sodium sulfate, concentrated by rotary evaporation, and directly subjected to wet loading onto a silica gel column. The target compound was purified using silica gel column chromatography with petroleum ether/dichloromethane (2:1) as the eluent, yielding the target compound **6OMeNAPBF$_2$**. Further purification was achieved by recrystallization from spectroscopic grade dichloromethane/hexane, affording 63.0 mg of the yellow solid with a yield of 21.7%. ^{1}H NMR (400 MHz, DMSO-d_6) δ 8.88 (d, *J* = 2.1 Hz, 1H), 8.19 – 8.06 (m, 2H), 8.00 (d, *J* = 8.8 Hz, 1H), 7.49 (d, *J* = 2.6 Hz, 1H), 7.33 (d, *J* = 8.8 Hz, 2H), 3.95 (s, 3H), 2.48 (s, 3H). ^{13}C NMR (126 MHz, DMSO-d_6) δ 193.10, 181.61, 161.17, 138.88, 132.38, 132.27, 128.32, 128.05, 125.83, 124.71, 120.74, 106.82, 98.43, 56.12, 24.81. ^{11}B NMR (128 MHz, DMSO-d_6) δ 0.05. ^{19}F NMR (376 MHz, DMSO-d_6) δ -123.00, -123.06. HRMS m/z found (calcd for $C_{15}H_{17}BF_2NO_3^+$): 307.1300 (307.1300).

Synthesis of compound $NAP1BF_2$

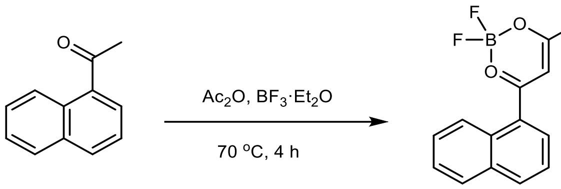

$NAP1BF_2$

To a round-bottom flask, 1-acetonaphthone (170.0 mg, 1.0 mmol), acetic anhydride (2.0 mL, 21.6 mmol), and boron trifluoride diethyl etherate (0.65 mL, 5.24 mmol) were added. The reaction mixture was stirred at 70 ℃ for 4 hours. After cooling to room temperature, the reaction mixture was quenched by adding it dropwise into cold water. The crude product was then extracted with dichloromethane and washed with deionized water. The organic phase was dried over anhydrous sodium sulfate, concentrated by rotary evaporation, and directly subjected to wet loading onto a silica gel column. The target compound was purified using silica gel column chromatography with petroleum ether/dichloromethane (2:1) as the eluent. Further purification was achieved by recrystallization from spectroscopic grade dichloromethane/hexane, yielding 160.8 mg of the target crystal, **$NAP1BF_2$**, with a yield of 61.8%. ^{1}H NMR (400 MHz, Chloroform-*d*) δ 8.50 (d, J = 8.7 Hz, 1H), 8.11 (d, J = 8.3 Hz, 1H), 7.98 – 7.89 (m, 2H), 7.72 – 7.52 (m, 3H), 6.51 (s, 1H), 2.46 (s, 3H). ^{13}C NMR (126 MHz, DMSO-d_6) δ 195.23, 185.65, 135.52, 133.97, 131.19, 129.97, 129.67, 129.65, 129.07, 127.53, 125.67, 124.92, 103.15, 25.02. ^{11}B NMR (128 MHz, DMSO-d_6) δ 0.02. ^{19}F NMR (376 MHz, DMSO-d_6) δ -135.53, -135.59. HRMS m/z found (calcd for $C_{14}H_{15}BF_2NO_2^+$): 277.1194 (277.1195).

Synthesis of compound $4OMeNAPBF_2$

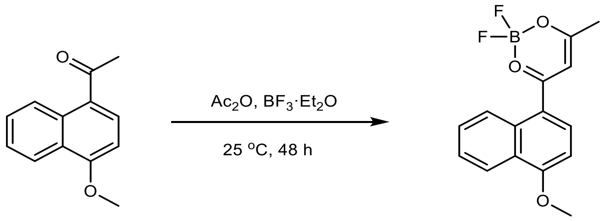

$4OMeNAPBF_2$

To a round-bottom flask, 1-(4-methoxynaphthalen-1-yl)ethanone (200.0 mg, 1.0 mmol), acetic anhydride (2.0 mL, 21.6 mmol), and boron trifluoride diethyl etherate (0.65 mL, 5.24 mmol) were added. The reaction mixture was stirred at 25 ℃ for 48

hours. After cooling to room temperature, the reaction mixture was quenched by adding it dropwise into cold water. The crude product was then extracted with dichloromethane and washed with deionized water. The organic phase was dried over anhydrous sodium sulfate, concentrated by rotary evaporation, and directly subjected to wet loading onto a silica gel column. The target compound was purified using silica gel column chromatography with petroleum ether/dichloromethane (2:1) as the eluent, yielding the target compound **4OMeNAPBF2**. Further purification was achieved by recrystallization from spectroscopic grade dichloromethane/hexane, affording 170.5 mg of the target crystal with a yield of 51.0%. ^{1}H NMR (400 MHz, DMSO-d_6) δ 8.60 (d, J = 8.6 Hz, 1H), 8.38 – 8.24 (m, 2H), 7.77 (ddd, J = 8.6, 6.8, 1.6 Hz, 1H), 7.66 (ddd, J = 8.2, 6.8, 1.2 Hz, 1H), 7.21 (d, J = 8.5 Hz, 1H), 7.03 (s, 1H), 4.13 (s, 3H), 2.45 (s, 3H). ^{13}C NMR (126 MHz, DMSO-d_6) δ 194.96, 183.79, 156.66, 133.90, 131.33, 130.47, 129.38, 129.29, 125.25, 117.30, 115.20, 115.01, 106.27, 57.02, 24.50. ^{11}B NMR (128 MHz, DMSO-d_6) δ 0.03. ^{19}F NMR (376 MHz, DMSO-d_6) δ -136.23, -136.29. HRMS m/z found (calcd for $C_{15}H_{17}BF_2NO_3^+$): 307.1301 (307.1300).

Synthesis of compound IndBF2

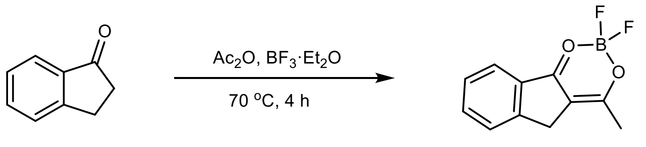

To a round-bottom flask, 1-indanone (260.0 mg, 2 mmol), acetic anhydride (6.0 mL, 63.6 mmol), boron trifluoride diethyl etherate (1.3 mL, 10.48 mmol), and chloroform (3.0 mL) were added. The reaction mixture was stirred at 70 ℃ for 4 hours. After completion, the reaction mixture was quenched by adding it dropwise into cold water. The crude product was then extracted with dichloromethane and washed with deionized water. The organic phase was dried over anhydrous sodium sulfate, concentrated by rotary evaporation, and directly subjected to wet loading onto a silica gel column. The target compound was purified using silica gel column chromatography with petroleum ether/dichloromethane (1:1) as the eluent, yielding a pale yellow solid, **IndBF2**. Further purification was achieved by recrystallization from spectroscopic

grade dichloromethane/hexane, affording 207.1 mg of the target crystal with a yield of 46.6%. ^{1}H NMR (500 MHz, DMSO-d_6) δ 7.98 (d, J = 7.7 Hz, 1H), 7.89 – 7.72 (m, 2H), 7.65 – 7.57 (m, 1H), 3.88 (s, 2H), 2.50 (s, 3H). ^{13}C NMR (126 MHz, DMSO-d_6) δ 186.56, 183.91, 150.26, 135.62, 133.11, 128.47, 126.47, 124.16, 113.07, 30.48, 22.16. HRMS m/z found (calcd for $C_{11}H_9BF_2O_2Na^+$): 244.0591 (244.0592).

Synthesis of compound OMeIndBF$_2$

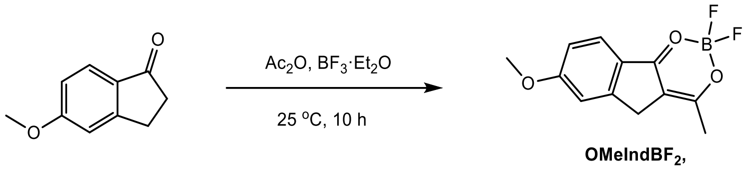

To a round-bottom flask, 5-methoxy-1-indanone (162.0 mg, 1 mmol), acetic anhydride (2.0 mL, 21.6 mmol), and boron trifluoride diethyl etherate (0.65 mL, 5.24 mmol) were added. The reaction mixture was stirred at 25 ℃ for 10 hours. Upon completion, the reaction mixture was quenched by adding it dropwise into cold water. The crude product was then extracted with dichloromethane and washed with deionized water. The organic phase was dried over anhydrous sodium sulfate, concentrated by rotary evaporation, and directly subjected to wet loading onto a silica gel column. The target compound was purified using silica gel column chromatography with petroleum ether/dichloromethane (1:1) as the eluent, yielding a pale yellow solid, **OMeIndBF$_2$**. Further purification was achieved by recrystallization from spectroscopic grade dichloromethane/hexane, affording 126.7 mg of the target crystal with a yield of 42.6%. ^{1}H NMR (400 MHz, Chloroform-d) δ 7.90 (d, J = 9.3 Hz, 1H), 7.06 (dq, J = 6.0, 2.2 Hz, 2H), 3.94 (s, 3H), 3.68 (s, 2H), 2.37 (s, 3H). ^{13}C NMR (101 MHz, DMSO-d_6) δ 184.16, 182.76, 166.70, 154.67, 126.57, 125.97, 117.21, 112.43, 111.14, 56.70, 30.87, 22.12. ^{11}B NMR (128 MHz, DMSO-d_6) δ 0.20. ^{19}F NMR (376 MHz, DMSO-d_6) δ -135.89, -135.96. HRMS m/z found (calcd for $C_{12}H_{15}BF_2NO_3^+$): 269.1144 (269.1144).

Synthesis of compound NAPpBP-NH2

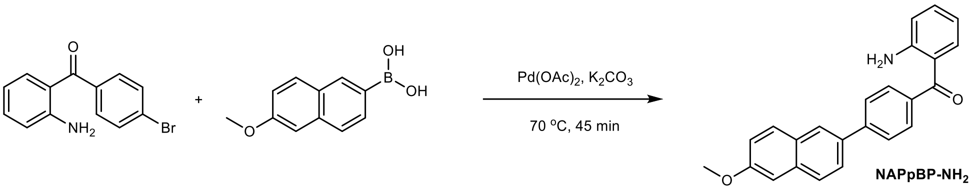

To a round-bottom flask, 6-methoxy-2-naphthylboronic acid (151.5 mg, 0.75 mmol), 2-amino-4-bromobenzophenone (138.0 mg, 0.5 mmol), potassium carbonate (152.0 mg, 1.10 mmol), palladium acetate (6.0 mg, 0.025 mmol), ethanol (2.0 mL), and deionized water (0.3 mL) were added. The reaction mixture was heated to 70 ℃ and stirred for 1 hour. After cooling to room temperature, the crude product was extracted with dichloromethane and washed with deionized water. The organic phase was dried over anhydrous sodium sulfate and concentrated via rotary evaporation. The resulting residue was purified by wet loading onto a silica gel column and eluted with dichloromethane/petroleum ether (1:1) to obtain 158.8 mg of a yellow flocculent compound, **NAPpBP-NH2**, with a yield of 90.5%. ^{1}H NMR (400 MHz, DMSO-d_6) δ 8.29 – 8.24 (m, 1H), 7.98 – 7.92 (m, 4H), 7.89 (dd, *J* = 8.6, 2.0 Hz, 1H), 7.72 – 7.66 (m, 2H), 7.40 – 7.35 (m, 2H), 7.31 (ddd, *J* = 8.6, 6.9, 1.7 Hz, 1H), 7.22 (dd, *J* = 9.0, 2.5 Hz, 1H), 7.08 (s, 2H), 6.88 (dd, *J* = 8.4, 1.2 Hz, 1H), 6.54 (ddd, *J* = 8.1, 7.0, 1.2 Hz, 1H), 3.91 (s, 3H).

Synthesis of compound NAPpBP-Biotin

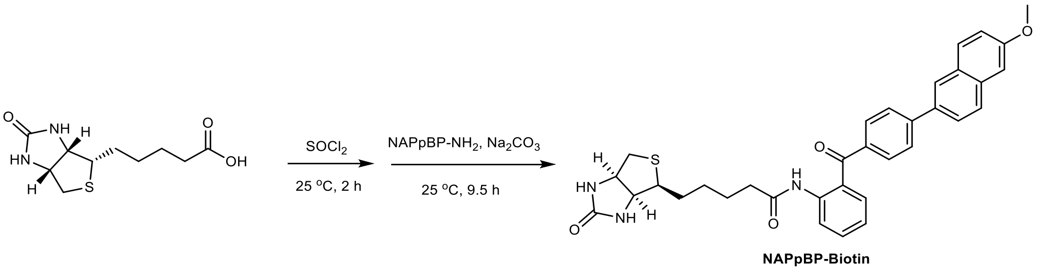

To a round-bottom flask, biotin (51.0 mg, 0.25 mmol) and thionyl chloride (0.5 mL, 0.89 mmol) were added, and the mixture was stirred at 25 ℃ for 2 hours. Following the reaction, an excess of sodium bicarbonate aqueous solution was added to the rotary evaporator flask to neutralize and remove the thionyl chloride by rotary evaporation.

This step was repeated several times with dichloromethane to ensure complete removal of thionyl chloride. Subsequently, **NAPpBP-NH2** (97.0 mg, 0.28 mmol), sodium carbonate (39.8 mg, 0.38 mmol), and dichloromethane (2.0 mL) were added to the round-bottom flask, and the reaction mixture was stirred at 25 ℃ for 9.5 hours. Upon completion of the reaction, the mixture was washed multiple times with water to remove residual sodium carbonate. The organic phase was extracted with dichloromethane, dried over anhydrous sodium sulfate, and the solvent was removed by rotary evaporation to yield the crude product **NAPpBP-Biotin**. Due to the poor solubility of **NAPpBP-Biotin**, purification was achieved by washing with a large amount of acetone and dichloromethane, resulting in the pure **NAPpBP-Biotin** (76.0 mg, 0.13 mmol) with a yield of 52.5%. ^{1}H NMR (400 MHz, DMSO-d_6) δ 10.02 (s, 1H), 8.30 – 8.25 (m, 1H), 7.98 – 7.91 (m, 4H), 7.88 (dd, *J* = 8.6, 1.9 Hz, 1H), 7.80 – 7.74 (m, 2H), 7.62 – 7.56 (m, 1H), 7.56 – 7.51 (m, 1H), 7.45 (dd, *J* = 7.8, 1.7 Hz, 1H), 7.38 (d, *J* = 2.7 Hz, 1H), 7.30 (ddd, *J* = 8.4, 7.4, 1.4 Hz, 1H), 7.22 (dd, *J* = 8.9, 2.4 Hz, 1H), 6.32 (d, *J* = 18.6 Hz, 2H), 4.14 – 4.07 (m, 1H), 3.94 (d, *J* = 4.7 Hz, 4H), 3.31 (s, 1H), 2.94 – 2.88 (m, 1H), 2.66 – 2.61 (m, 1H), 2.10 – 2.02 (m, 2H), 1.49 (dd, *J* = 14.5, 7.4 Hz, 1H), 1.33 (dq, *J* = 14.6, 7.5 Hz, 3H), 1.15 (d, *J* = 6.1 Hz, 2H).

Synthesis of compound NAPpBP-NHCOCH3

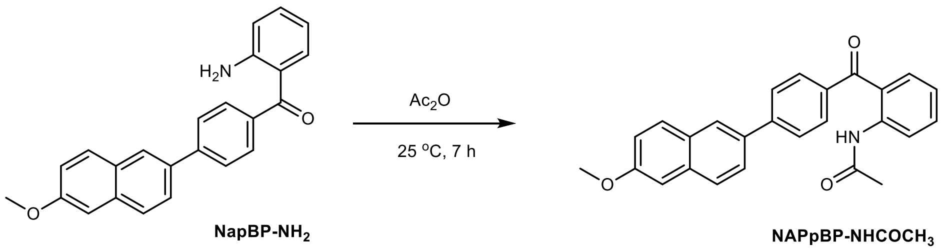

To a round-bottom flask, **NAPpBP-NH2** (71.0 mg, 0.2 mmol), acetic anhydride (0.35 mL, 3.73 mmol), and dichloromethane (5.0 mL) were added. The reaction mixture was stirred at 25 ℃ for 7 hours. Upon completion, the reaction mixture was directly subjected to wet loading onto a silica gel column and purified using petroleum ether/dichloromethane (1:4) as the eluent. This process yielded a yellow solid,

NAPpBP-NHCOCH$_3$, with a yield of 67.1%. ^{1}H NMR (400 MHz, DMSO-d_6) δ 10.03 (s, 1H), 8.23 (s, 1H), 7.98 – 7.70 (m, 7H), 7.60 – 7.46 (m, 2H), 7.43 – 7.31 (m, 2H), 7.30 – 7.10 (m, 2H), 3.86 (s, 3H), 1.76 (s, 3H).

Synthesis of compound 1NAP-Biotin

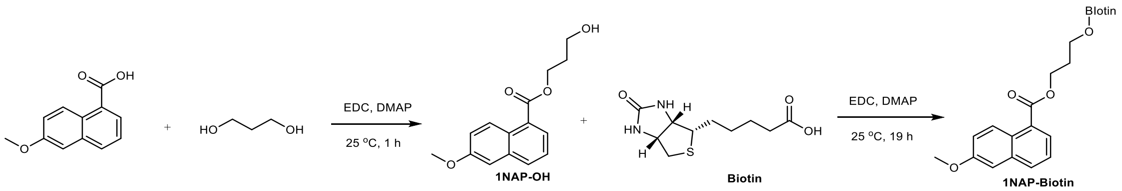

To a round-bottom flask, 6-methoxy-1-naphthoic acid (101.0 mg, 0.5 mmol), propylene glycol (228.0 mg, 3.0 mmol), 3-(3-dimethylaminopropyl)-1-ethylcarbodiimide hydrochloride (480.0 mg, 2.5 mmol), N, N-dimethylpyridine (305 mg, 2.5 mmol), and dichloromethane (5.0 mL) were added. The reaction mixture was stirred at 25 ℃ for 1 hour. Upon completion, the reaction mixture was directly subjected to wet loading onto a silica gel column and purified using ethyl acetate/dichloromethane (1:10) as the eluent, yielding 125.5 mg of a white solid, **1NAP-OH**, with a yield of 96.5%.

Subsequently, **1NAP-OH** (86.7 mg, 0.33 mmol), biotin (81.5 mg, 0.33 mmol), 3-(3-dimethylaminopropyl)-1-ethylcarbodiimide hydrochloride (3.8 g, 20.0 mmol), N,N-dimethylpyridine (2.0 g, 50.0 mmol), and dichloromethane (10.0 mL) were added to a round-bottom flask. The reaction mixture was stirred at 25 ℃ for 19 hours. Upon completion, the organic phase was washed repeatedly with aqueous hydrochloric acid until the pH reached neutrality, followed by a final wash. The product was extracted with dichloromethane, dried over anhydrous magnesium sulfate, and the solvent was removed by rotary evaporation. The product was further purified by recrystallization in dichloromethane/hexane, yielding 146.7 mg of the yellow compound, **1NAP-Biotin**, with a yield of 90.0%. ^{1}H NMR (400 MHz, DMSO-d_6) δ 8.67 (d, J = 9.5 Hz, 1H), 8.10 (d, J = 7.9 Hz, 1H), 7.99 (dd, J = 7.3, 1.4 Hz, 1H), 7.57 (t, J = 7.7 Hz, 1H), 7.45 (d, J = 2.7 Hz, 1H), 7.31 (dd, J = 9.4, 2.7 Hz, 1H), 6.38 (d, J = 24.3 Hz, 2H), 4.46 – 4.39 (m, 2H), 4.32 – 4.24 (m, 1H), 4.24 – 4.17 (m, 2H), 4.12 – 4.06 (m, 1H), 3.90 (s, 3H), 3.07

– 3.00 (m, 1H), 2.78 (dd, *J* = 12.5, 5.1 Hz, 1H), 2.56 (d, *J* = 12.1 Hz, 1H), 2.27 (t, *J* = 7.5 Hz, 2H), 2.09 (p, *J* = 6.2 Hz, 2H), 1.62 – 1.46 (m, 3H), 1.41 (dt, *J* = 13.9, 7.2 Hz, 1H), 1.28 (dt, *J* = 13.7, 6.6 Hz, 2H).

Synthesis of compound 1NAP-PEG

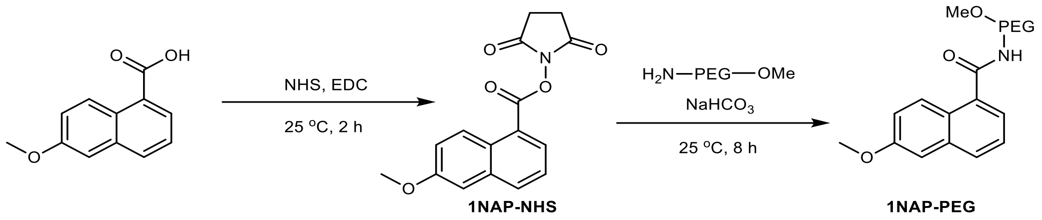

Synthesized the compound **1NAP-NHS** via a synthetic route identical to aforementioned procedures. Then to a round-bottom flask, **1NAP-NHS** (28.3 mg, 0.1 mmol), NH_2-PEG-OMe (80.0 mg, 0.04 mmol), and chloroform (3.0 mL) were added. The reaction mixture was stirred at 25 ℃ for 8 hours. Upon completion, the reaction mixture was concentrated via rotary evaporation and purified by precipitating the product with ice-cold diethyl ether, followed by rapid filtration. This process was repeated three times to yield 74.0 mg of a white solid identified as **1NAP-PEG**, with an isolated yield of 67.7%.

Synthesis of compound 2COOHNAP-diketone-PEG

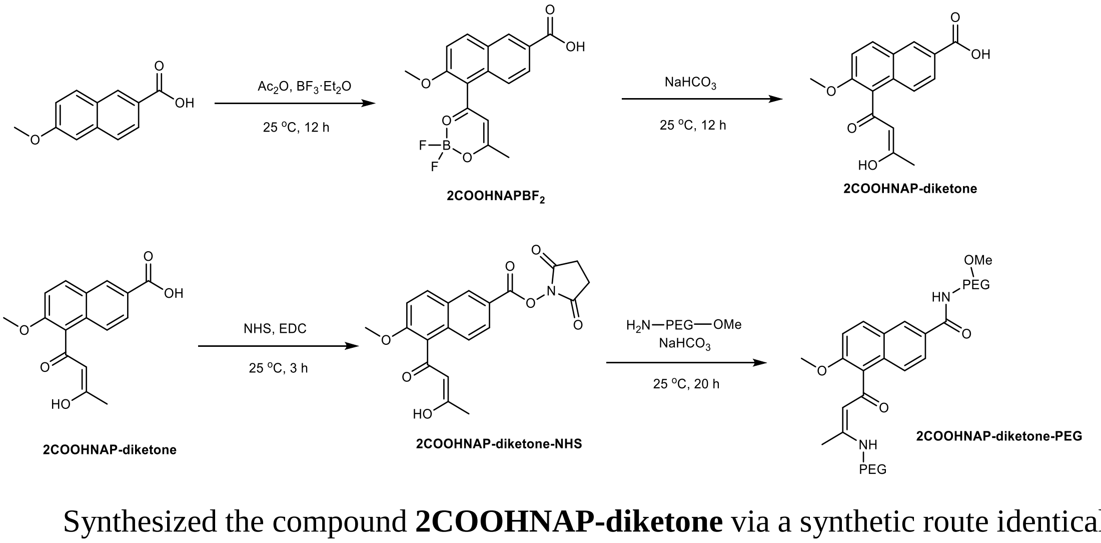

Synthesized the compound **2COOHNAP-diketone** via a synthetic route identical to aforementioned. Then to a round-bottom flask, **2COOHNAP-diketone** (70.1 mg, 0.25 mmol), N-hydroxysuccinimide (58.0 mg, 0.5 mmol), 3-(3-dimethylaminopropyl)-

1-ethylcarbodiimide hydrochloride (120.0 mg, 0.6 mmol), and dichloromethane (8.0 mL) were added. The reaction mixture was stirred at 25 ℃ for 3 hours. Upon completion, the reaction mixture was concentrated by rotary evaporation, and the crude product was purified via silica gel column chromatography using dichloromethane/ethyl acetate (4:1) as the eluent, yielding 57.0 mg of the target compound **2COOHNAP-diketone-NHS** with a yield of 60.9%.

To a round-bottom flask, **2COOHNAP-diketone-NHS** (18.4 mg, 0.05 mmol), NH_2-PEG-OMe (40.0 mg, 0.02 mmol), and chloroform (2.0 mL) were added. The reaction mixture was stirred at 25 ℃ for 20 hours. Upon completion, the reaction mixture was concentrated by rotary evaporation, and the target compound was purified by precipitation using cold diethyl ether followed by rapid filtration, repeated three times, yielding 30.5 mg **2COOHNAP-diketone-PEG** with a yield of 64.9%.

Synthesis of compound NAPpBP-PEG

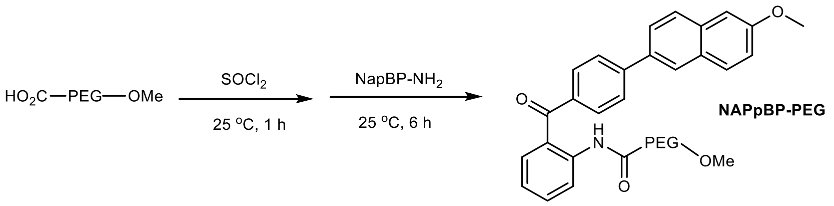

To a round-bottom flask, HOOC-PEG-OMe (100.0 mg, 0.05 mmol) and thionyl chloride (0.25 mL, 0.45 mmol) were added, and the mixture was stirred at 25 ℃ for 1 hour. Following this, the reaction was subjected to rotary evaporation with the addition of an excess of $NaHCO_3$ aqueous solution to the rotary evaporator flask to remove thionyl chloride. This step was repeated several times with dichloromethane to ensure complete removal of thionyl chloride. Next, **NAPpBP-NH$_2$** (17.5 mg, 0.05 mmol) and dichloromethane (3.0 mL) were added to the round-bottom flask, and the reaction mixture was stirred at 25 ℃ for 6 hours. Upon completion of the reaction, the resulting solution, a pale-yellow emulsion, was filtered, and the filtrate was rotary evaporated. The residue was dissolved in a suitable amount of dichloromethane and purified by precipitation with cold diethyl ether, followed by rapid filtration, repeated three times. This process yielded 76.5 mg of **NAPpBP-PEG**, with a yield of 65.8%.

Synthesis of compound NAPBP-CDCA

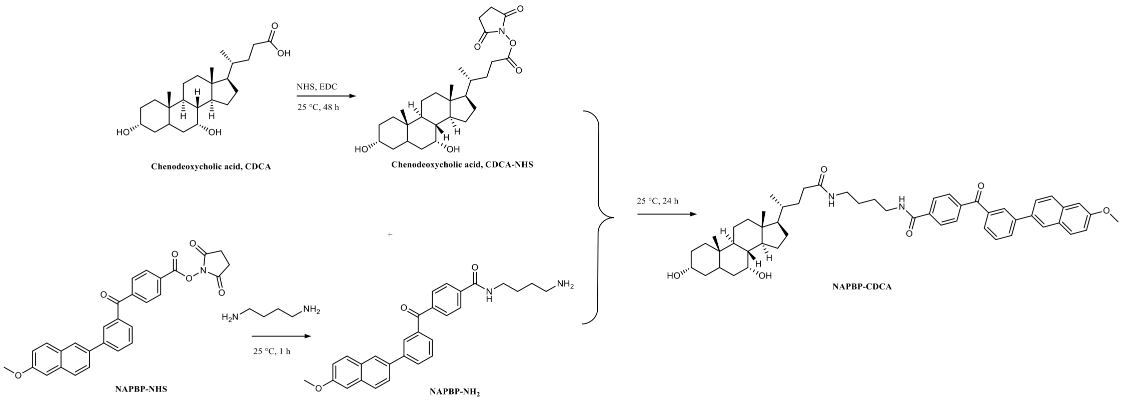

To a 250 mL round-bottom flask, chenodeoxycholie acid (3.92 g, 10 mmol), *N*-hydroxysuccinimide (3.05 g, 25 mmol), and 1,3-dicyclohexylcarbodiimide (6.18 g, 30 mmol) were added under a nitrogen atmosphere. The mixture was dissolved in a solvent mixture of 10 mL acetonitrile and 50 mL tetrahydrofuran and stirred at 25 °C for 48 hours, resulting in the formation of a significant amount of white precipitate. Following filtration, a milky-white solid was obtained and subsequently washed with ethyl acetate. The filtrate was concentrated under reduced pressure to yield 118 mg of a white solid identified as **CDCA-NHS**, corresponding to a yield of 2.3%. The synthetic procedures are the same as the reported study (*J. Org. Chem.* **2006**, *71,* 7205-7213).

In a separate reaction, **NAPBP-NHS** (48.0 mg, 0.1 mmol) and 1,4-butanediamine (88.0 mg, 0.1 mmol) were introduced into a 25 mL round-bottom flask. The compounds were dissolved in a mixed solvent of 2 mL ethanol and 0.5 mL acetone and allowed to react at 25 °C for 1 hour. Upon completion of the reaction, the mixture was diluted with dichloromethane and subjected to extensive washing with amount of deionized water (at least five times). The organic phase was dried over anhydrous sodium sulfate, concentrated, and evaporated to afford **NAPBP-NH$_2$**. Subsequently, **CDCA-NHS** (48.0 mg, 0.1 mmol) was added to this solution, which was then dissolved in 5 mL of dichloromethane and reacted at 25 °C for 24 hours. A 0.75 mL aliquot of the reaction mixture was applied to a silica gel plate, and separation was performed using

DCM/ethanol (8:1) as the developing solvent, yielding 5 mg of the target product **NAPBP-CDCA**. HRMS m/z found (calcd for $C_{53}H_{68}N_2NaO_7^+$): 867.4223 (867.4919).

Synthesis of compound NAPBP

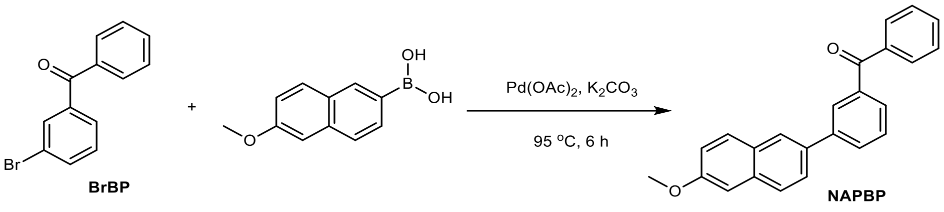

To a round-bottom flask, 6-methoxy-2-naphthylboronic acid (131.3 mg, 0.65 mmol), **Br-BP** (130.6 mg, 0.5 mmol), potassium carbonate (173.8 mg, 1.25 mmol), palladium acetate (5.6 mg, 0.025 mmol), ethanol (1.5 mL), and deionized water (0.75 mL) were added. The reaction mixture was heated to 95 ℃ and stirred for 6 hours. After cooling to room temperature, the reaction mixture was poured into dilute hydrochloric acid and stirred for 10 minutes. The crude product was extracted with a large volume of a dichloromethane/acetone solvent mixture and washed with deionized water. The organic phase was dried over anhydrous sodium sulfate, concentrated by rotary evaporation, and purified via silica gel column chromatography using dichloromethane/ethyl acetate (30:1) as the eluent, yielding the white compound **NAPBP**. The **NAPBP** was further purified by two cycles of recrystallization in spectroscopic grade dichloromethane/hexane with a yield of 53.6%. ^{1}H NMR (500 MHz, DMSO-d6) δ 8.31 (s, 1H), 8.02 (d, J = 8.1 Hz, 2H), 7.99–7.95 (m, 2H), 7.93–7.87 (m, 3H), 7.80 (d, J = 7.5 Hz, 2H), 7.71 (d, J = 7.6 Hz, 1H), 7.61 (t, J = 7.6 Hz, 2H), 7.40 (d, J = 2.5 Hz, 1H), 7.27–7.22 (m, 1H), 3.92 (s, 3H). The synthetic procedures are similar to our previous studies (*Chem. Commun.* **2023**, *59*, 1525-1528; *ACS Materials Letters*, **2024**, *6*, 1042-1049).

In vitro cytotoxicity evaluation

DMEM (Dulbecco's Modified Eagle Medium) supplemented with 10% FBS (Fetal Bovine Serum) and 1% Penicillin-Streptomycin were used in the NIH3T3 cells cultivation. Cells were maintained in a 37°C incubator with 5% CO_2 and passaged regularly to maintain exponential growth. Then, NIH3T3 cells were seeded at a density of 5000 cells per well in the 96-well plates for the MTT assay. The cells were allowed to adhere to the plate for 24 hours before sample liquid was applied. The sample liquid was processed with syringe filter (pore size 0.22 μm) for sterilization. Then the sample liquid was added in the 96 well plate with cells at a volume of 100 μL/well without further process. The cells were maintained in a 37°C incubator with 5% CO_2. At the designated time points (Day 1 and Day 2), 10 μL of MTT solution (5 mg/mL) was added to each well. The plates were then incubated for 2 hours at 37 ℃. During this incubation, viable cells reduced the MTT reagent to form insoluble formazan crystals. After incubation, the medium was removed, and 100 μL of DMSO (dimethyl sulfoxide) was added to each well to dissolve the formazan crystals. The absorbance of the resulting solution was measured at 570 nm using a microplate reader. The relative cell viability was determined by comparing the absorbance of treated wells to that of the control group.

Physical measurements and instrumentation

Nuclear magnetic resonance (NMR) spectra were recorded on a JEOL Fourier-transform NMR spectrometer (400 MHz), including ^{1}H NMR, $^{13}C\{^{1}H\}$ NMR, $^{19}F\{^{1}H\}$ NMR, $^{11}B\{^{1}H\}$ NMR. Mass spectra were performed on Agilent Q-TOF 6520 liquid chromatograph mass spectrometer. FT-IR spectra were recorded on a Nicolet AVATAR-360 FT-IR spectrophotometer with a resolution of 4 cm^{-1}. Single-crystal X-ray diffraction analysis was performed on a D8 VENTURE SC-XRD instrument. UV-Vis absorption spectra were recorded on a Techcomp UV1050, Unico UV-4802, and Shimadzu UVmini1285 UV-vis spectrophotometer. The steady-state and delayed emission spectra were collected by Hitachi FL-4700 fluorescence spectrometer equipped with chopping systems; the delayed emission spectra were obtained with a delay time of approximately 1 ms. The excited state decay profiles in millisecond to second region were collected by Hitachi FL-4700 fluorescence spectrometer equipped with chopping systems. The fluorescence decay profiles in nanosecond region were recorded by using time-correlated single photon counting technique (TCSPC) on a Edinburgh FLS1000 fluorescence spectrometer equipped with a picosecond pulsed diode laser. Photoluminescence quantum yield was measured by a Hamamatsu absolute PL quantum yield measurement system based on a standard protocol (*Adv. Mater.* 1997, 9: 230). Photographs and videos were captured by iPhone 13 and 14 cameras. Before the capture, samples were irradiated by a 365 nm UV lamp (5 W) for approximately 5 s at a distance of approximately 5 cm.

TD-DFT calculations

The ground-state geometry of compounds were optimized by a DFT calculation using B3LYP functional and 6-31G (d, p) basis set; the singlet excited states and triplet excited states were calculated on Gaussian 16 program (Revision A.03) with B3LYP functional and 6-31G (d, p) basis set; spin-orbit coupling (SOC) matrix elements between the singlet excited states and triplet excited states were calculated with spin-orbit mean-field (SOMF) methods on ORCA 4.2.1 program with B3LYP functional and def2-TZVP(-f) basis set. The obtained electronic structures were analyzed by Multiwfn software. All isosurface maps to show the electron distribution and electronic transitions were rendered by Visual Molecular Dynamics (VMD) software based on the exported files from Multiwfn (Neese F, *Wiley Interdiscip. Rev. Comput. Mol. Sci.* **2018**, *8*, 1327-1332; Becke AD, *Phys. Rev. A* 1988, **38**, 3098-3100; Lee C, Yang W, Parr RG, *Phys. Rev. B.* **1988**, *37*, 785-789; Miehlich B, Savin A, Stoll H, Preuss H, *Chem. Phys. Lett.* **1989**, *157*, 200-206; Weigend F, Ahlrichs R, *Phys. Chem. Chem. Phys.* **2005**, *7*, 3297-3305; Lu T, Chen F, *J. Comput. Chem.* **2012**, *33*, 580-592; Humphrey W, Dalke A, Schulten K, *J. Mol. Graphics* **1996,** *14*, 33-38).

Supporting Text S1

One may comment that the term “afterglow ice” may be of potential ambiguity, as it implies that the ice itself is the source of luminescence. However, we would like to clarify that this term is explicitly introduced and clearly defined in the abstract of our manuscript, where we state: “the observation of organic afterglow from aqueous solutions of luminophore-protein conjugates and complexes at freezing temperature, named as afterglow ice for abbreviation.” This phrasing makes it clear that the luminescence originates from the luminophore-protein systems embedded within the ice matrix, rather than the ice itself. Throughout the manuscript, the term “afterglow ice” is consistently used to refer to this specific phenomenon. Given that the term is clearly defined at the beginning, we believe it does not introduce any significant risk of misinterpretation. Moreover, as an interesting phenomenon, “afterglow ice” serves not only as a descriptive term but also as a concise label for the breakthrough itself—facilitating clear communication of our findings within the scientific community.
