# Supplementary material for: Afterglow ice formed by phosphorescent luminophore-protein conjugates and complexes in aqueous solution at freezing temperature
Source: Nat Commun. 2025 Dec 22;17:226. doi: 10.1038/s41467-025-67670-z (PMC12779962; doi:10.1038/s41467-025-67670-z)
Supplement: Supplementary file 2 — Description of Additional Supplementary Files [file 41467_2025_67670_MOESM2_ESM.pdf]

Description of Additional Supplementary Files

File Name: Supplementary Data 1
Description: The atomic coordinates of the optimized ground states of NAPBP-COOH.
